# Small peptide diversification through photoredox-catalyzed oxidative C-terminal modification†

**DOI:** 10.1039/d0sc06180h

**Published:** 2021-01-07

**Authors:** Eliott Le Du, Marion Garreau, Jérôme Waser

**Affiliations:** Laboratory of Catalysis and Organic Synthesis, Institut des Sciences et Ingénierie Chimique, Ecole Polytechnique Fédérale de Lausanne Lausanne CH-1015 Switzerland jerome.waser@epfl.ch

## Abstract

A photoredox-catalyzed oxidative decarboxylative coupling of small peptides is reported, giving access to a variety of *N*,*O*-acetals. They were used as intermediates for the addition of phenols and indoles, leading to novel peptide scaffolds and bioconjugates. Amino acids with nucleophilic side chains, such as serine, threonine, tyrosine and tryptophan, could also be used as partners to access tri- and tetrapeptide derivatives with non-natural cross-linking.

## Abstract 2

A photoredox approach for the generation of *N*-acyliminiums derived from peptides enabling diversification *via* Friedel–Crafts reactions.

## Graphical abstract

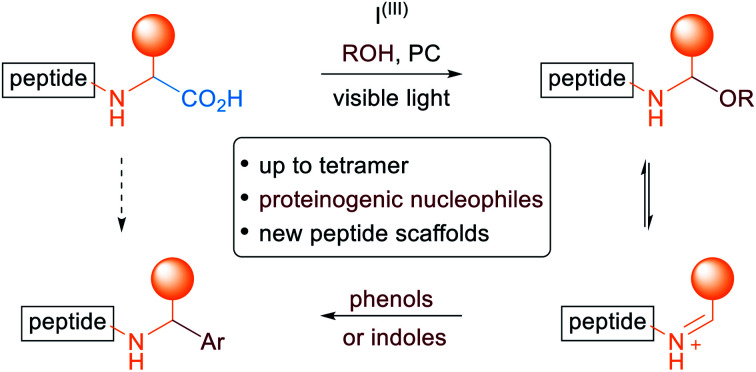

## 1. Introduction

Bioactive peptides have gained a lot of attention in the past decade as therapeutic compounds interacting with receptors or inhibiting protein–protein interactions. The biological activity and metabolic stability of native peptides is usually improved by the introduction of unnatural amino-acids (UAA) and rigidifying elements.^1^ Moreover bioconjugation between peptides and other types of synthetic or natural bioactive molecules opens access to a broader chemical space. This is particularly interesting for drug discovery as it allows combining the best of two worlds, such as the higher target affinity of peptides and the better membrane permeability of small non polar synthetic compounds.^2^ This strategy can be used to finely tune the pharmacological properties of drugs.^3^ With the ambition to develop an efficient method for the structural diversification of peptides, we were interested in the functionalization of the C-termini of peptides, which can have a fundamental influence on their bioactivity.^4^ In addition, as the C-terminus is unique and free in most peptides, such an approach would be at the same time general and selective. Most methods for the modification of native peptides rely on the specific reactivity of nucleophilic residues present in amino acids such as cysteine and lysine, and examples of functionalization of the C-terminus remain scarce.^5^

**Scheme 1:**
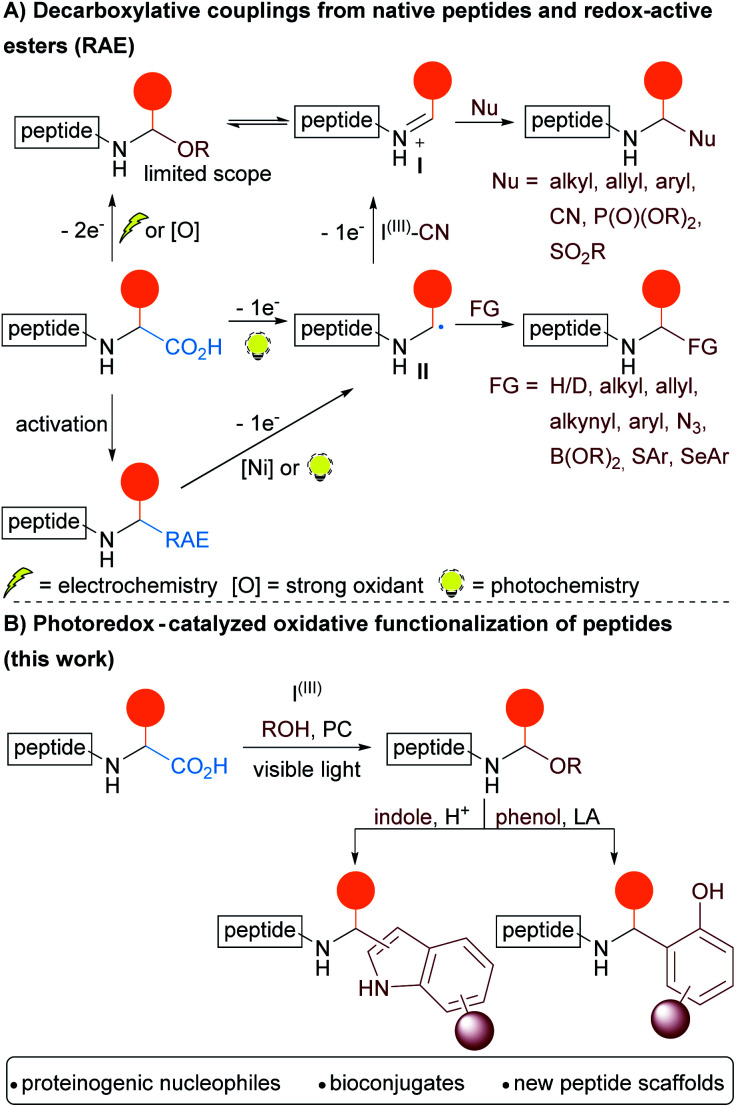
(A) Decarboxylative functionalization of peptides; (B) our photoredox strategy for the synthesis of bioconjugates.

Recently, decarboxylative coupling has emerged as a method of choice to reach this goal, either *via* one or two-electron processes (Scheme 1A). Concerning the latter, the oxidative decarboxylative conversion of carboxylic acids to alcohols under electrochemical conditions was already reported by Hofer and Moest in 1902.^6^ Seebach and coworkers later applied this method to amino acids and small peptides.^7^ In the presence of methanol or acetic acid, *N*,*O*-acetals were generated. Activation of the *N*,*O*-acetals to give versatile *N*-acyliminium intermediates **I**^8^ then allowed the addition of different nucleophiles (Grignard reagents, phosphites, allylsilanes, terminal alkyne or TMSCN). In 2020, the Malins group reported an extension of this approach to more complex peptides for the total synthesis of analogues of Biseokeaniamides A–C.^9^

The formation of *N*,*O*-acetals could also be achieved using chemical oxidants, in particular hypervalent iodine reagents. The Suárez group investigated the decarboxylative hydroxylation of proline derivatives under visible light using phenyliodine(iii) diacetate (PIDA) as oxidant.^10^ The strategy was later used by Kita and coworkers on non-cyclic amino-acids, but using heating instead of light to initiate the oxidation step.^11^ This method, often coupled with *in situ* functionalization of the acetals, was then applied to amino acids and small peptides.^12^

The advent of photoredox catalysis contributed to the development of new strategies for bioconjugation *via* one–electron processes (Scheme 1A).^13^ Starting from native peptides, C-terminal decarboxylative arylation,^14^ reduction,^15^ allylation,^16^ alkynylation,^17^ cyanation,^18^ azidation^19^ and Giese coupling^20^ have been described. Decarboxylative coupling reactions can alternatively be performed by activation of the carboxylic acid *via* the formation of a redox-active ester (RAE). Single Electron Transfer (SET) from a metal complex or from a photocatalyst then induces the decarboxylation.^13*d*^ Using nickel complexes, the Baran group elegantly functionalized peptides through decarboxylative alkylation,^21^ alkenylation,^22^ alkynylation,^23^ Giese coupling^24^ and borylation.^25^ Moreover, photoredox catalyzed decarboxylative arylation,^26^ thio-^27^ and selenoarylation^28^ were also reported. The generation of an α-aminyl radical intermediate **II** was involved in all these processes. However, in the case of the cyanation methodology involving a hypervalent iodine reagent, computation indicated that the radical was further oxidized to form an iminium intermediate, constituting one of the rare cases of two-electron oxidation under photoredox catalysis.^18^

Decarboxylative photoredox catalyzed C–O bonds formation has been described only in a few examples of protected amino acids, and is often associated with overoxidation to give amides.^29^ To the best of our knowledge, the generation of peptide derived *N*,*O*-acetals from free carboxylic acids has never been reported using photoredox conditions. When considering that photocatalysis can proceed under milder conditions (room temperature, visible light, weaker oxidants) and requires less technical know-how than electrochemistry, such a method would be highly useful for synthetic and medicinal chemists requiring modified peptides. Furthermore, the method would be complementary to existing one-electron approaches: functional group tolerance would be lower, but access to versatile iminium intermediates would allow extensive diversification of the products with nucleophiles, leading to different types of bond formations.

Herein, we report a photoredox catalyzed oxidative-decarboxylative coupling of small peptides (Scheme 1B). This methodology allowed the synthesis of modified peptides with *N*,*O*-acetals at the C-terminus, which served as intermediates to introduce phenols and indoles *via* Friedel–Crafts reactions. The scope of nucleophiles included alcohols and electron-rich aromatic residues present in natural amino acids such as serine, threonine, tyrosine and tryptophan, giving access to new types of peptide cross-linking for the synthesis of non-natural tri- and tetrapeptides.

## 2. Results and discussions

### 2.1. Discovery and optimization of the reaction

We started our study by exploring the oxidative decarboxylation of Cbz–Gly–Pro (**1a**) in presence of methanol as the model reaction (Table 1). The hypervalent iodine reagent acetoxybenziodoxolone (BI-OAc, **2a**) was selected as oxidant for the optimization, due to its recent success in oxidative decarboxylative reactions.^30^

**Table 1:** Optimization of the oxidative decarboxylation of dipeptides

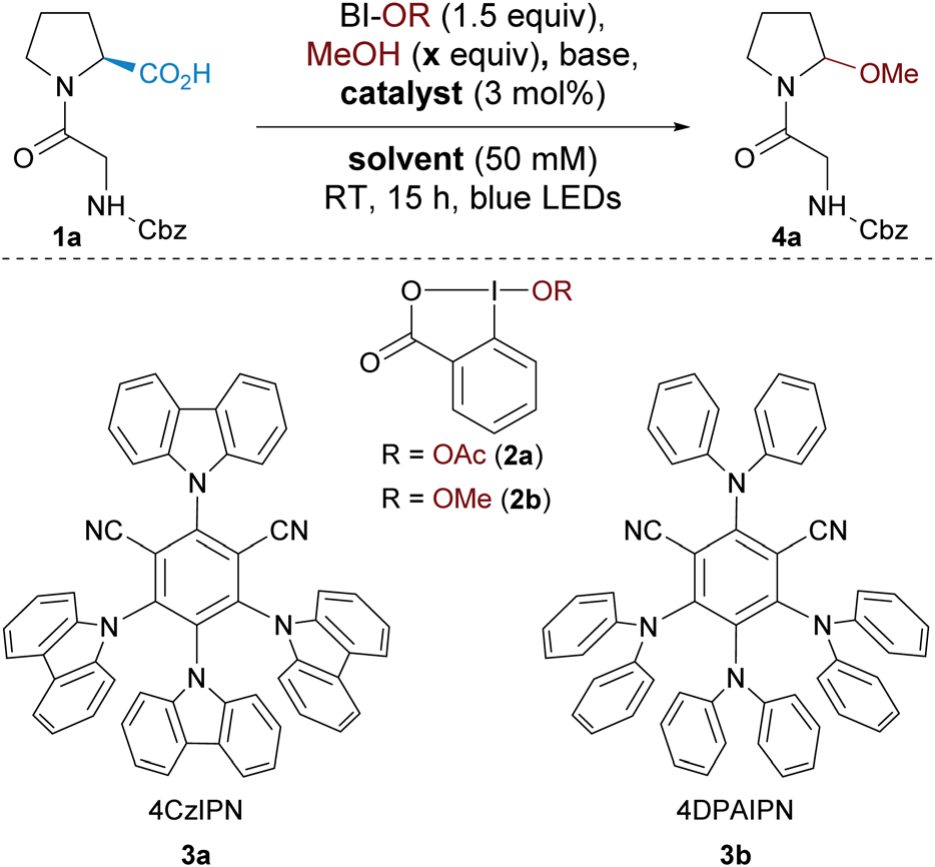					
Entry	Solvent	Catalyst	*x*	Base (2 equiv.)	HPLC yield^a^ (%)
1^b^	DMF	4CzIPN (**3a**)	50	K_2_HPO_4_	46 (18)^c^
2^b^	DMF	Ru(bpy)_3_Cl_2_	50	K_2_HPO_4_	59
3^b^	DMF	Ru(bpy)_3_Cl_2_	10	K_2_HPO_4_	78
4	DMF	Ru(bpy)_3_Cl_2_	10	K_2_HPO_4_	82
5	DMF	Ru(bpy)_3_Cl_2_	5	K_2_HPO_4_	>95
6	DMF	Ru(bpy)_3_Cl_2_	2	K_2_HPO_4_	>95
7	DMF	Eosin Y	5	K_2_HPO_4_	17
8^d^	DMF	Rhodamine B	5	K_2_HPO_4_	27
9^d^	DMF	Rose bengal	5	K_2_HPO_4_	35
10^d^	DMF	4DPAIPN (**3b**)	5	K_2_HPO_4_	45
11	MeCN	Ru(bpy)_3_Cl_2_	5	K_2_HPO_4_	>95
**12**	**MeCN**	**Ru(bpy)**_**3**_ **Cl**_**2**_	**5**	**None**	**>95 (68)** c
**13**	**DCE**	**Ru(bpy)**_**3**_ **Cl**_**2**_	**5**	**None**	**>95**
**14** e	**MeCN**	**Ru(bpy)**_**3**_ **Cl**_**2**_	**0**	**None**	**>95 (75)** c

a Ratio of integration at 214 nm by RP-HPLC.

b Concentration 10 mM.

c Isolated yield on 0.3 mmol.

d Green LEDs.

e BI-OMe (**2b**) instead of BI-OAc (**2a**).

Based on our work on the alkynylation of peptides using organic photoredox catalysts,^17^ 4CzIPN (2,4,5,6-tetra(9*H*-carbazol-9-yl)isophthalonitrile, **3a**) was selected as photocatalyst and K_2_HPO_4_ as base. To our delight the desired methanol *N*,*O*-acetal **4a** was formed with 50 equivalents of MeOH in DMF in 46% HPLC yield and 18% isolated yield (entry 1). Changing the photocatalyst to Ru(bpy)_3_Cl_2_ slightly increased the yield (entry 2).^31^ Decreasing the amount of MeOH in the reaction mixture and increasing the concentration had a beneficial impact on the yield (entries 3 and 4). Full conversion was observed with either 5 or 2 equivalents of methanol (entries 5 and 6). For practical reasons, 5 equivalents of methanol were employed for the rest of the optimization, but 2 equivalents only of alcohols will be used during the investigation of the scope of the reaction.

With those conditions in hand we decided to screen some organic dyes to accomplish a metal-free process. We thus selected organophotocatalysts with similar oxidative properties in the excited state as Ru(bpy)_3_Cl_2_ (Ru^II^*/Ru^I^: +0.77 V *vs.* SCE).^32^ However, neither Eosin Y (EY*/EY˙^−^: +0.83 V *vs.* SCE),^33^ rhodamine B (RhB*/RhB˙^−^: +0.84 V *vs.* SCE)^34^ nor rose bengal (RB*/RB˙^−^: +0.81 V *vs.* SCE)^34^ were suitable for the transformation and degradation of the dyes was observed (entries 7–9). Similarly, despite its promising redox properties, 4DPAIPN (2,4,5,6-tetrakis(diphenylamino)isophthalonitrile, **3b**) (4DPAIN*/4DPAIN˙^−^: 0.90 V *vs.* SCE)^17^ only afforded the desired product in 45% yield (entry 10). Unfortunately DMF was not a suitable solvent for further functionalization of the *N*,*O*-acetals. Based on the reported use of *N*,*O*-acetals to generate *N*-acyliminiums in MeCN,^35^ we tested this solvent and full and clean conversion was observed (entry 11). A control experiment without base was attempted and full conversion to product **4a** was also observed (entry 12). Under these conditions, *N*,*O*-acetal **4a** could be isolated in 68% yield on a 0.3 mmol scale. A similar result was obtained in DCE (entry 13). Interestingly, the reaction was also working with methoxybenziodoxolone (BI-OMe, **2b**) and the corresponding *N*,*O*-acetal **4a** was obtained in 75% isolated yield (entry 14). A slightly higher yield was obtained with **2b** but the necessity to pre-form the hypervalent iodine reagent prior to the reaction is not desirable for more complex alcohols. Finally, control experiments were carried out and only traces of the desired product were observed in the absence of light or catalyst.

### 2.2. Scope of the oxidative decarboxylation reaction

**Scheme 2:**
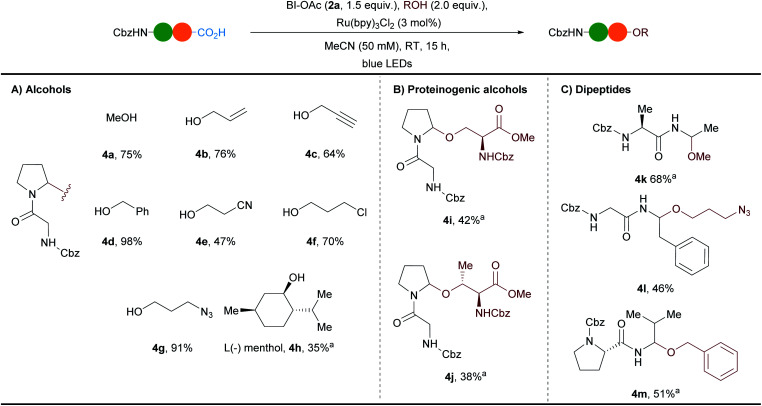
Scope of obtained *N*,*O*-acetals. Reactions performed on 0.3 mmol scale. Yields of isolated products are given. ^a^Compound obtained with a low diastereoselectivity that could not be determined exactly due to the presence of rotamers.

A robustness test with different protected amino acids present in the reaction mixture showed a moderate functional group tolerance,^36^ setting the basis for the investigation of the scope (Scheme 2). Previous works based on electrochemistry or chemical oxidation had focused mostly on the incorporation of simple alcohols as solvents.^7^ Our method was already efficient with only two equivalents of methanol, as well as allyl, propargyl and benzyl alcohols, to give *N*,*O*-acetals **4b–d** in 64–98% yield (Scheme 2A). Functional groups such as cyanide, chloride and azide were well tolerated and the corresponding *N*,*O*-acetals **4e–g** were obtained in 47–91% yield. The introduction of bioorthogonal terminal alkyne or azido groups is highly valuable as it allows to further functionalize the products. The more sterically hindered secondary alcohol (L)-menthol gave the *N*,*O*-acetal **4h** in 35% yield.

The method could be extended to the alcohol-containing amino acids serine and threonine allowing to access a new type of cross-linked peptides **4i** and **4j** in 42% and 38% yield, respectively (Scheme 2B). Finally the conditions were successfully applied to other dipeptides (Cbz–Ala–Ala (**1b**), Cbz–Gly–Phe (**1c**) and Cbz–Pro–Val (**1d**)) with various alcohols and the *N*,*O*-acetals **4k–m** were formed in 46–68% yields as mixtures of diastereoisomers in the case of **4k** and **4m** (Scheme 2C).

### 2.3. Functionalization of the *N*,*O*-acetals

**Scheme 3:**
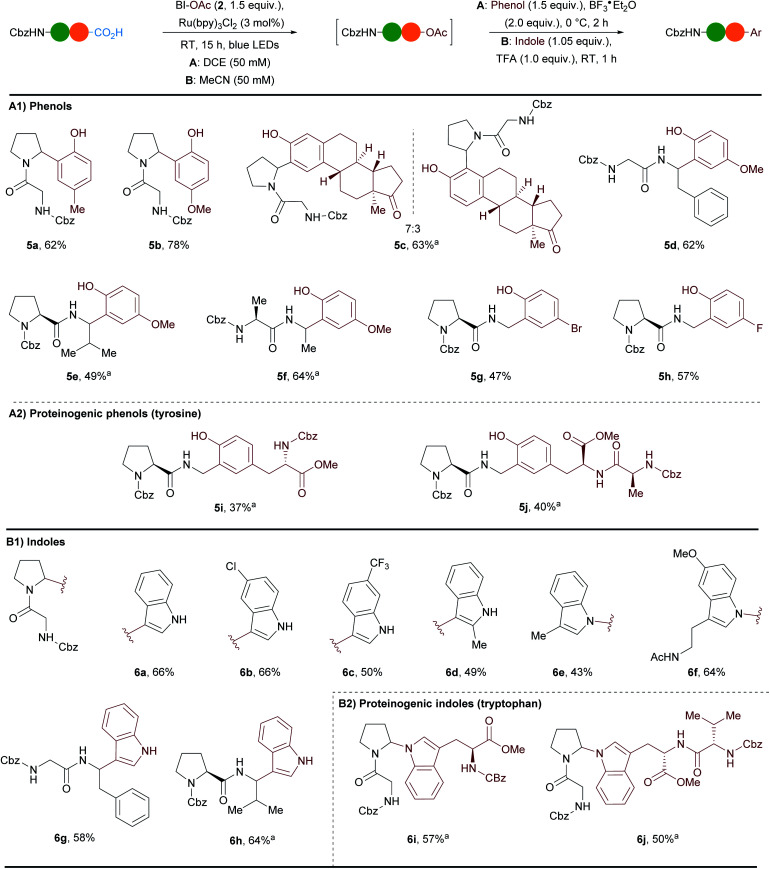
Scope of the one-pot arylation of dipeptides with phenols and indoles. Reactions performed on 0.3 mmol scale. Yields of isolated products are given. ^a^Compound obtained with a low diastereoselectivity that could not be determined exactly due to the presence of rotamers.

During our studies on the formation of *N*,*O*-acetals we observed that in absence of alcohol the reaction was still taking place and *N*,*O*Ac-acetals were generated. As we could not isolate these compounds, we wondered whether we could take advantage of their reactivity for the direct reaction with nucleophiles (Scheme 3).

Based on the report of White and coworkers,^37^ we examined the addition of *p*-cresol in presence of BF_3_·Et_2_O as Lewis acid. While only degradation was observed in MeCN, we were pleased to observe full conversion of Cbz–Gly–Pro (**1a**) to Friedel–Crafts product **5a** in DCE (Scheme 3A(1), conditions A). After the one-pot two steps procedure **5a** could be obtained in 62% yield with complete selectivity for the *ortho* position. Similarly, electron-rich *para*-methoxyphenol could be added to give **5b** in 78% yield. Estrone was also compatible with the reaction conditions: a 7 : 3 mixture of two regioisomers of **5c** was obtained in 63% yield with a preference for the position in *para* to the alkyl donating group. *Para*-methoxyphenol was successfully employed for the arylation of other dipeptides (Cbz–Gly–Phe (**1c**), Cbz–Pro–Val (**1d**) and Cbz–Ala–Ala (**1b**)) to give products **5d–f** in 49–64% yield. Cbz–Pro–Gly (**1e**) could also be arylated at the C-terminal position. Electron-withdrawing groups such as bromine and fluorine were tolerated on the phenol with this more reactive iminium and the corresponding products **5g** and **5h** were obtained in 47% and 57% yield respectively. To our delight phenols in tyrosine were compatible with our method (Scheme 3A(2)). The addition of Cbz–Tyr–OMe and Cbz–Ala–Tyr–OMe led to the formation of unprecedented unnatural tri- and tetrapeptides **5i** and **5j** in 37% and 40% yield respectively.

We were then interested in adding indoles, which are also proteinogenic nucleophiles present in tryptophan. Wang and coworkers recently reported an asymmetric Friedel–Crafts reaction between indoles and *N*-acyl iminiums generated *in situ* by decarboxylation of RAE derived from amino acids.^38^ However, this transformation was not extended to peptides and prefunctionalization of the carboxylic acids was needed. Inspired by conditions reported by our group,^35^ a one-pot two steps arylation procedure directly from free carboxylic acids was developed using a slight excess of indoles and one equivalent of TFA (Scheme 3B(1), conditions B). Using Cbz–Gly–Pro (**1a**) as model substrate several indoles reacted preferentially at the C3-position. 1*H*-indole afforded **6a** in 66% yield. A chlorine substituent in C6 position and a trifluoromethyl substituent in C7 position were tolerated and led to the formation of the desired products **6b** and **6c** in 66% and 50% yield respectively. 2-Methylindole could be introduced in 49% yield onto dipeptide **1a**. When C3-substituted indoles were employed, N-addition was the major outcome. For instance, 3-methylindole led to the formation of **6e** in 43% yield. C2-addition was also detected in the crude mixture (ratio N/C2-addition 2 : 1), but the corresponding product could not be isolated in pure form. Interestingly, melatonin, which is a hormone helping to regulate the sleep–wake cycle and which can be used for short-term treatment of insomnia, was compatible with the reaction conditions. Product **6f** was obtained in 64% yield. Cbz–Gly–Phe (**1c**) and Cbz–Pro–Val (**1d**) could also be arylated with 1*H*-indole and the corresponding products **6g** and **6h** were obtained in 58% and 64% yields respectively. The tryptophan indoles of Cbz–Trp–OMe and Cbz–Val–Trp–OMe reacted *via* C–N bond formation to give the unprecedented unnatural tri- and tetrapeptides **6i** and **6j** in 57% and 50% yield respectively (Scheme 3B(2)).

**Scheme 4:**
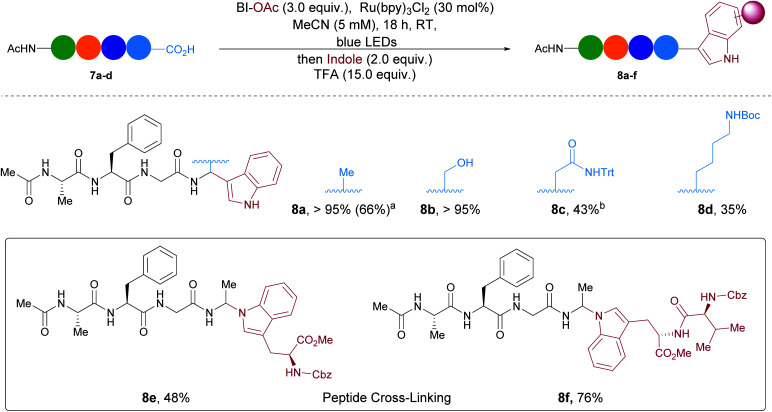
Preliminary results towards arylation of tetrapeptides. Reactions performed on 1 μmol scale. Relative HPLC ratios of the area of the product over remaining starting material and peptide side-products at 214 nm are given. Average of 3 independent trials. ^a^Calibrated yield, see SI for details. ^b^Compound **8c** could not be fully separated from a side product by HPLC.

Finally, we investigate the extension of our methodology to larger peptides (Scheme 4). Ac–Ala–Phe–Gly–Ala (**7a**) was selected as model substrate. By increasing the amount of oxidant and photocatalyst, indole could be added successfully to **7a**. The desired modified tetrapeptide **8a** was the only product observed by HPLC and was obtained in 66% yield (See ESI† for more details). Serine was also tolerated at the C-terminus and full conversion towards **8b** was observed. A protected amide from asparagine and a protected amine from lysine were moderately tolerated leading to products **8c** and **8d**. Moreover, more complex unnatural peptides could be obtained by reaction with proteinogenic indoles from Cbz–Trp–OMe and Cbz–Val–Trp–OMe. For instance **8e** and **8f** were formed in moderate to good HPLC ratios. Protected aspartic acid, histidine and arginine were also submitted to the reaction conditions and led to the formation of arylated peptides but with lower HPLC ratios (<25%, see SI for more details).^39^ Unfortunately, the current conditions for phenols were not compatible with tetramers and would require further optimization .

## 3. Conclusion

In summary, we have developed a photoredox-catalyzed oxidative decarboxylative strategy towards the introduction of diverse functional groups on peptides. Under the developed conditions, valuable alcohols were successfully introduced leading to structurally diverse *N*,*O*-acetals. Moreover, the *N*,*O*-acetals were also employed as key reactive intermediates for arylation with phenols and indoles. Bioconjugation of bioactive compounds such as estrone or melatonin with dipeptides was possible. Additionally, serine, threonine, tyrosine and tryptophan derivatives could be used as nucleophilic partners to give new types of cross-linked peptides. As a proof of concept for the arylation of larger peptides, indole and two tryptophan derivatives were successfully added to several tetrapeptides.

## Conflicts of interest

There are no conflicts to declare.

## Supplementary Material

SC-012-D0SC06180H-s001
