# Supplementary material for: Small peptide diversification through photoredox-catalyzed oxidative C-terminal modification
Source: Chem Sci. 2021 Jan 7;12(7):2467–73. doi: 10.1039/d0sc06180h (PMC8179259; doi:10.1039/d0sc06180h)

Electronic Supplementary Material (ESI) for Chemical Science.
This journal is © The Royal Society of Chemistry 2020

Table of Contents

1. General methods 2
2. Preparation of reagents and catalysts 4
3. Peptide synthesis 7
4. Optimization 8
5. Scope on dipeptides 10
6. Scope on Tetrapeptides 31
7. References 40
8. NMR spectra 41

1. General methods

All reactions were carried out in oven dried glassware under an atmosphere of nitrogen, unless for the oxidative decarboxylation and if stated otherwise. For flash chromatography, distilled technical grade solvents were used. THF, CH_3CN, toluene and CH_2Cl_2 were dried by passage over activated alumina under nitrogen atmosphere (H_2O content < 10 ppm, Karl-Fischer titration). All chemicals were purchased from Acros, Aldrich, Fluka, VWR, TCI, Merck or Bachem and used as such unless stated otherwise. All dipeptides starting materials were commercially available and used as received. Chromatographic purification was performed as flash chromatography using Macherey-Nagel silica 40-63, 60 Å, using the solvents indicated as eluent with 0.1-0.5 bar pressure. TLC was performed on Merck silica gel 60 F254 TLC aluminum or glass plates and visualized with UV light and $KMnO_4$ stain. ^1H-NMR spectra were recorded on a Brucker DPX-400 400 MHz spectrometer in chloroform-d, DMSO-d^6 or acetonitrile-d^3, all signals are reported in ppm with the internal chloroform signal at 7.26 ppm, the internal DMSO signal at 2.50 ppm or the internal acetonitrile signal at 1.94 ppm as standard. The data is being reported as (s = singlet, d = doublet, t= triplet, q = quadruplet, qi = quintet, m = multiplet or unresolved, br = broad signal, app = apparent, coupling constant(s) in Hz, integration, interpretation).^{13}C-NMR spectra were recorded with ^1H-decoupling on a Brucker DPX-400 100 MHz spectrometer in chloroform-d, DMSO-d^6 or acetonitrile-d^3, all signals are reported in ppm with the internal chloroform signal at 77.0 ppm, the internal DMSO signal at 39.5 ppm or the internal acetonitrile signals at 1.32 and 118.26 ppm as standard. Infrared spectra were recorded on a JASCO FT-IR B4100 spectrophotometer with an ATR PRO410-S and a ZnSe prisma and are reported as cm-1 (w = weak, m = medium, s = strong, br = broad).

High resolution mass spectrometric measurements were performed by the mass spectrometry service of ISIC at the EPFL on a MICROMASS (ESI) Q-TOF Ultima API. MS-MS analyses were performed on a LTQ Orbitrap FTMS instrument (LTQ Orbitrap Elite FTMS, Thermo Scientific, Bremen, Germany) operated in the positive mode coupled with a robotic chip-based nano-ESI source (TriVersa Nanomate, Advion Biosciences, Ithaca, NY, U.S.A.). A standard data acquisition and instrument control system was utilized (Thermo Scientific) whereas the ion source was controlled by Chipsoft 8.3.1 software (Advion BioScience). Samples were loaded onto a 96-well plate (Eppendorf, Hamburg, Germany) within an injection volume of 5µl. The experimental conditions for the ionization voltage was +1.4kV and the gas pressure was set at 0.30 psi. The temperature of ion transfer capillary was 275 °C, tube voltages. FTMS spectra were obtained in the 80-1000 *m/z* range in the reduce profile mode with a resolution set to 120,000. In all spectra one microscan was acquired with a maximum injection time value of 1000ms. Typical CID experiments were carried out using Normalized collision energy values of 26-28 and 5 Da of isolation width.

Photoredox catalyzed reactions were performed in test tubes (5 and 10 mL), which were hold using a rack for test tubes placed at the center of a crystallization flask. On this flask were attached the blue LEDs (RUBAN LED 5MÈTRES - 60LED/M - 3528 BLEU - IP65 with Transformateur pour Ruban LED 24W/2A/12V, bought directly on RubanLED.com). The distance between the LEDs and the test tubes was approximatively 2 cm for the test tubes

and 5 cm for the Schlenk flasks. Long irradiation resulted in temperature increasing up to 37°C during overnight reactions.

Tetramers peptides were synthesized by solid phase peptide synthesis using a Multipep RSi Intavis. Crude products were purified by preparative RP-HPLC on an Agilent 1260 HPLC system with a G2260A 1260 Prep ALS Autosampler, a G1361a 1260 Prep Pump, a G1365C 1260 MWD detector and a G1364B 1260 FC-PS collector, coupled with a Waters XBridge semi-preparative C18 column (19 x 150 mm, 5 µm). Water (solvent A) and water:acetonitrile 5:95 (solvent B), each containing 0.1% TFA, were used as the mobile phase at a flow rate of 20 mL.min-1. The following method was used: 100% A to 100% B in 20 minutes.

RP-HPLC-MS measurements were performed on an Agilent 1290 Infinity HPLC system with a G4226a 1290 Autosampler, a G4220A 1290 Bin Pump and a G4212A 1290 DAD detector, connected to a 6130 Quadrupole LC/MS MS, coupled with a Waters XBridge C18 column (250 x 4.6 mm, 5 µm). Water:acetonitrile 95:5 (solvent A) and water:acetonitrile 5:95 (solvent B), each containing 0.1% formic acid, were used as the mobile phase at a flow rate of 0.6 mL/min-1. The gradient was programmed as follows: 100% A to 100% B in 20 minutes then isocratic for 5 minutes. The column temperature was set up to 25 °C. Low resolution mass spectrometric measurements were acquired using the following parameters: positive electrospray electrospray ionization (ESI), temperature of drying gas = 350 °C, flow rate of drying gas = 12 L. min-1, pressure of nebulizer gas = 60 psi, capillary voltage = 2500 V and fragmentor voltage = 70 V.

2. Preparation of reagents and catalysts

1-Hydroxy-1,2-benziodoxol-3-(1*H*)-one (10)

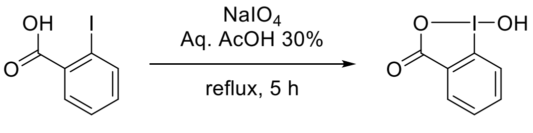

9 **10**

Following a reported procedure,[1] $NaIO_4$ (40.5 g, 189 mmol, 1.05 equiv) and 2-iodobenzoic acid (**9**) (44.8 g, 180 mmol, 1.0 equiv) were suspended in 30% (v:v) aq. AcOH (350 mL). The mixture was vigorously stirred and refluxed for 5 h. The reaction mixture was then diluted with cold water (250 mL) and allowed to cool to rt, protecting it from light. After 1 h, the crude product was collected by filtration, washed on the filter with ice water (3 x 150 mL) and acetone (3 x 150 mL), and air-dried in the dark overnight to afford 1-Hydroxy-1,2-benziodoxol-3-(1*H*)-one (**10**) (44.3 g, 168 mmol, 93%) as a white solid.

^{1}H NMR (400 MHz, DMSO-d_6) *δ* 8.02 (dd, *J* = 7.7, 1.4 Hz, 1H, Ar*H*), 7.97 (m, 1H, Ar*H*), 7.85 (dd, *J* = 8.2, 0.7 Hz, 1H, Ar*H*), 7.71 (td, *J* = 7.6, 1.2 Hz, 1H, Ar*H*). ^{13}C NMR (100 MHz, DMSO-d_6) δ 167.7, 134.5, 131.5, 131.1, 130.4, 126.3, 120.4. The values of the NMR spectra are in accordance with reported literature data.[1]

1-Acetoxy-1,2-benziodoxol-3-(1H)-one (BI-OAc, 2)

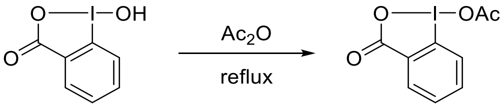

10 **2**

Following a reported procedure,[2] 1-hydroxy-1,2-benziodoxol-3-(1H)-one (**10**, 10.3 g, 39.1 mmol, 1.00 equiv) was suspended in acetic anhydride (35 mL) and heated to reflux for 30 minutes. The resulting clear, slightly yellow solution was slowly let to warm up to room temperature and then cooled to 0 °C for 30 minutes. The white suspension was filtered and the filtrate was again cooled to 0 °C for 30 minutes. The suspension was once again filtered and the combined two batches of solid product were washed with hexane (2 x 20 mL) and dried in vacuo affording **2** (10.8 g, 35.3 mmol, 90%) as a white solid.

^{1}H NMR (400 MHz, Chloroform-*d*) δ 8.24 (dd, 1H, *J* = 7.6, 1.6 Hz, Ar*H*), 8.00 (dd, 1H, *J* = 8.3, 1.0 Hz, Ar*H*), 7.92 (ddd, 1H, *J* = 8.4, 7.2, 1.6 Hz, Ar*H*), 7.71 (td, 1H, *J* = 7.3, 1.1 Hz, Ar*H*), 2.25 (s, 3H, COCH_3). ^{13}C NMR (100 MHz, Chloroform-*d*) δ 176.5, 168.2, 136.2, 133.3, 131.4, 129.4, 129.1, 118.4, 20.4. The values of the NMR spectra are in accordance with reported literature data.[2]

1-Metoxy-1,2-benziodoxol-3-(1H)-one (BI-OMe, 2b)

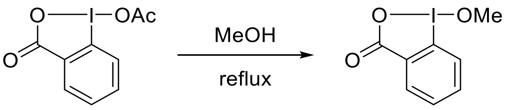

2 **11**

Following a reported procedure,[3] BI-OAc (**2a**, 1.0 g, 3.3 mmol, 1.0 equiv) was refluxed in MeOH (10 mL) for 15 min until a clear, colorless solution was obtained. The mixture was cooled to room temperature and then to -20°C. The precipitate was filtered, washed with a minimal amount of MeOH, and dried under vacuum. BI-OMe **2b**(0.69 g, 2.5 mmol, 76%) was obtained as white crystals.

^{1}H NMR (400 MHz, Chloroform-*d*) δ 8.27 (dd, *J* = 7.6, 1.6 Hz, 1H, Ar*H*), 7.90 (ddd, *J* = 8.5, 7.2, 1.6 Hz, 1H, Ar*H*), 7.76 (dd, *J* = 8.3, 1.0 Hz, 1H, Ar*H*), 7.69 (td, *J* = 7.4, 1.0 Hz, 1H, Ar*H*), 4.27 (s, 3H, O*Me*). ^{13}C NMR (101 MHz, Chloroform-*d*) δ 168.1, 135.2, 133.0, 131.1, 130.7, 126.0, 118.6, 62.4. The values of the NMR spectra are in accordance with reported literature data.[3]

Preparation of catalysts

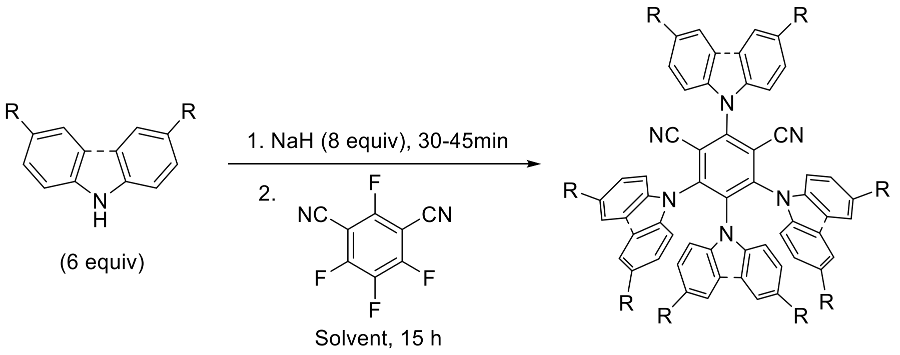

General procedure 1:

Sodium hydride (60% suspension in mineral oil, 8.0 equiv) was added slowly to a stirred solution of substituted-carbazole (5.0 equiv) in dry THF (0.05 M) under a nitrogen atmosphere at RT. After 30 min, 2,4,5,6-tetrafluoroisophthalonitrile (1.0 mmol, 1.0 equiv) was added. After stirring at RT for 15 h, 2 mL water was added to the reaction mixture to quench the excess of NaH. The resulting mixture was then concentrated under reduced pressure. The crude product was purified by recrystallization from hexane/CH_2Cl_2 then filtered. The brown liquid filtrate was concentrated and recrystallized as before. The combined solids were then purified by column chromatography on silica gel with DCM/Hexane.

General procedure 2:

Sodium hydride (60% suspension in mineral oil, 8.0 equiv) was added slowly to a stirred solution of substituted-diphenylamine (6.0 equiv) in dry DMF (0.1 M) under a nitrogen atmosphere at RT. After 45 min - 1 h, 2,4,5,6-tetrafluoroisophthalonitrile (1.0 equiv) was added. After stirring at RT for 15 h, water and ice were added to the reaction mixture to quench the excess of NaH. The precipitate was filtered and purified by recrystallization from pentane/CH_2Cl_2 then filtered. The brown liquid filtrate was concentrated and recrystallized as before. The combined solids were then purified by column chromatography on silica gel with DCM/Hexane.

2,4,5,6-Tetra(9*H*-carbazol-9-yl)isophthalonitrile (4CzIPN, 3a)

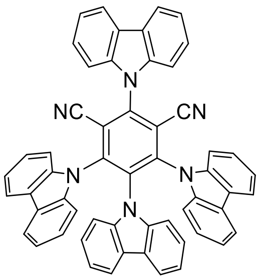

Following the general procedure 1 and starting from 9H-carbazole **12** (1.67 g, 10.0 mmol, 5.00 equiv), sodium hydride (0.60 g, 15 mmol, 7.5 equiv) and 2,4,5,6-tetrafluoroisophthalonitrile **13** (0.40 g, 2.0 mmol) in 40 mL of THF. Recrystallization (Hexanes/CH_2Cl_2 (1:1, 90 mL)) afforded the crude product as a yellow powder. Column chromatography afforded 2,4,5,6-tetra(9H-carbazol-9-yl)isophthalonitrile (**3a**) as a bright yellow crystalline solid (1.14 g, 1.45 mmol, 73 % yield).

Rf (Hexane/DCM 1/1) = 0.29. (yellow spot on TLC). ^{1}H NMR (400 MHz, Chloroform-*d*) δ 8.2 (d, *J* = 7.7 Hz, 2H, Ar*H*), 7.8 – 7.6 (m, 8H, Ar*H*), 7.5 (ddd, *J* = 8.0, 6.6, 1.6 Hz, 2H, Ar*H*), 7.3 (d, *J* = 7.5 Hz, 2H, Ar*H*), 7.2 (dd, *J* = 8.4, 1.5 Hz, 4H, Ar*H*), 7.2 – 7.0 (m, 8H, Ar*H*), 6.8 (t, *J* = 7.8 Hz, 4H, Ar*H*), 6.6 (td, *J* = 7.6, 1.2 Hz, 2H, Ar*H*).^{13}C NMR (101 MHz, Chloroform-*d*) δ 145.2, 144.6, 140.0, 138.2, 136.9, 134.7, 127.0, 125.8, 124.9, 124.7, 124.5, 123.8, 122.4, 121.9, 121.4, 121.0, 120.4, 119.6, 116.3, 111.6, 109.9, 109.5, 109.4. The values of the NMR spectra are in accordance with reported literature data.[4]

2,4,5,6-Tetrakis(diphenylamino)isophthalonitrile (4DPAIPN, 3b)

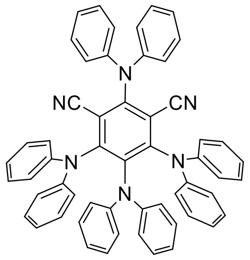

Following the general procedure 2 and starting from diphenylamine **14** (1.01 g, 6.00 mmol, 6.0 equiv), sodium hydride (320 mg, 8.00 mmol, 8.0 equiv) and 2,4,5,6-tetrafluoroisophthalonitrile **13** (200 mg, 1.00 mmol) in 10 mL of DMF. The deprotonation was performed at 50°C for 1 h, followed by stirring at the same temperature for 4 h. Recrystallization (CH_2Cl_2/pentane (1:2)) gave 2,4,5,6-tetrakis(diphenylamino)isophthalonitrile (**3b**) as a yellow-orange crystalline solid (400 mg, 0.502 mmol, 50 % yield).

Rf (pentane/DCM 1:1): 0.3. (yellow spot on TLC). ^{1}H NMR (400 MHz, Chloroform-*d*) δ 7.32 – 7.22 (m, 4H, Ar*H*), 7.12 – 7.05 (m, 12H, Ar*H*), 7.07 – 6.98 (m, 2H, Ar*H*), 6.96 – 6.84 (m, 8H, Ar*H*), 6.73 – 6.63 (m, 10H, Ar*H*), 6.56 (d, *J* = 7.4 Hz, 4H, Ar*H*). ^{13}C NMR (101 MHz, Chloroform-*d*) δ 154.2, 151.7, 145.5, 144.6, 143.1, 140.3, 129.4, 128.6, 127.5, 124.2, 123.9, 122.9, 122.6, 122.6, 121.1, 113.1, 113.0. IR (ν_{max}, cm^{-1}) 3065 (w), 3040 (w), 2361 (w), 1586 (m), 1535 (m), 1497 (s), 1415 (s), 1275 (m), 1244 (m), 1028 (w), 907 (m), 742 (s), 698 (s). HRMS (ESI/QTOF) m/z: [M + H]$^+$ Calcd for $C_{56}H_{41}N_6^+$ 797.3387; Found 797.3375. The values of the NMR spectra are in accordance with reported literature data.[5]

3. Peptide synthesis

The used dipeptides were commercially available. All peptide tetramers were synthesized by solid phase peptide synthesis using a 2-chlorotrityl chloride resin (1.0-1.6 mmol/g, 100-200 mesh). The first amino acid was loaded on the resin by incubation of the Fmoc-protected monomer (3 equiv of the number of active sites on the resin), DIPEA (4 equiv) in dichloromethane for 2 h. A cycle consisted first of the deprotection, achieved by stirring for 20 min with a 20% solution of piperidine in DMF, twice. Then the resin was washed with DMF (7x). Double couplings were performed by adding the Fmoc-protected monomer (4 equiv), HBTU (4 equiv), HOBt (4 equiv), NMM (4 equiv) and stirring for 45 min. Capping was carried out at the end of each cycle, followed by a DMF wash (7x). Acetylation of the N-terminal was achieved by incubating the resin with an Acetic Anhydride/DIPEA/DMF 10/15/75 solution for 30 min, twice. Cleavage of peptides with no protecting groups on the side-chains was performed by stirring the resin in a 20% solution of HFIP in dichloromethane for 30 min. In the presence of protecting groups, a TFA/water/triisopropylsilane 95/2.5/2.5 was used instead and the stirring time increased to 2 h. The cleavage mixture was poured into cold diethyl ether and precipitated peptides were recovered. The crude peptides were purified by preparative RP-HPLC using a gradient water-95% acetonitrile in 20 min. Pure peptides were analyzed by RP-HPLC and HRMS.

4. Optimization

4.1. Optimization of the oxidative decarboxylation

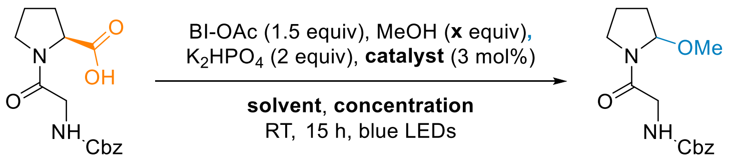

Degassed solvent was added in a 10 mL test tube containing a teflon coated stirring bar, Z-Gly-Pro (**1a**) (31 mg, 0.10 mmol, 1.0 equiv), R-BX (**2**) (0.15 mmol, 1.5 equiv), the base and the catalyst under a nitrogen atmosphere. The reaction mixture was irradiated using blue light LEDs at RT.

Procedure for HPLC yields:

The reaction was monitored by dilution of 50 μL of the crude with 950 μL of acetonitrile. The yield was estimated by the absorbance of product in comparison to the overall absorbance of product, unreacted starting material and side-products if any.

Procedure for isolated yields:

The crude mixture was diluted with 10 mL of sat. $NaHCO_3$ and extracted with diethyl ether (3 x 50 mL). The combined organic layers were washed with brine (3 x 20 mL), dried over $MgSO_4$, filtered and concentrated under vacuum. The crude product was purified by preparative TLC (DCM/ethyl acetate 7:3).

Table S1. Optimization of the oxidative decarboxylation on dipeptides

Entry	Solvent	Concentration (mM)	Catalyst	Base (equiv)	Alcohol (equiv)	HPLC yield (%)[a]
1	DMF	10	4CzIPN (**3a**)	K_2HPO_4 (2)	MeOH (50)	46
2	DMF	10	$Ru(bpy)_3Cl_2 \cdot 6H_2O$	K_2HPO_4 (2)	MeOH (50)	59
3	DMF	10	$Ru(bpy)_3Cl_2 \cdot 6H_2O$	K_2HPO_4 (2)	MeOH (10)	78
4	DMF	50	$Ru(bpy)_3Cl_2 \cdot 6H_2O$	K_2HPO_4 (2)	MeOH (10)	82
5	DMF	50	$Ru(bpy)_3Cl_2 \cdot 6H_2O$	K_2HPO_4 (2)	MeOH (5)	>95
6	DMF	50	$Ru(bpy)_3Cl_2 \cdot 6H_2O$	K_2HPO_4 (2)	MeOH (2)	>95
7	DMF	50	Eosin Y	K_2HPO_4 (2)	MeOH (5)	17
8[b]	DMF	50	Rhodamine B	K_2HPO_4 (2)	MeOH (5)	27
9[b]	DMF	50	Rose Bengal	K_2HPO_4 (2)	MeOH (5)	35
10[b]	DMF	50	4DPAIPN (**3b**)	K_2HPO_4 (2)	MeOH (5)	45
11	MeCN	50	$Ru(bpy)_3Cl_2 \cdot 6H_2O$	K_2HPO_4 (2)	MeOH (5)	>95
12	MeCN	50	$Ru(bpy)_3Cl_2 \cdot 6H_2O$	-	MeOH (5)	>95 (68)[c]
13	DCE	50	$Ru(bpy)_3Cl_2 \cdot 6H_2O$	-	MeOH (5)	>95

[a] Ratio of integration at 214 nm by RP-HPLC, [b] green LEDs, [c] isolated yield.

Control experiments were carried out and only traces of the desired product were observed in the absence of light or catalyst.

4.2. Robustness experiments

Degassed MeCN (2 mL) was added in a 5 mL test tube containing Cbz-Gly-Pro (**1a**) (31 mg, 0.10 mmol, 1.0 equiv), the protected amino acid (0.1 mmol, 1 equiv), BI-OMe (**2b**) (42 mg, 0.45 mmol, 1.5 equiv) and $Ru(bpy)_3Cl_2 \cdot 6H_2O$ (2.3 mg, 3.00 µmol, 3 mol%) under a nitrogen atmosphere. The reaction mixture was irradiated using blue light LEDs for 15 h at RT.

The reaction was monitored by dilution of 50 µL of the crude with 950 µL of acetonitrile. The yield was estimated by the absorbance of product in comparison to the overall absorbance of product, unreacted starting material and side-products if any.

Table S2. Robustness experiments

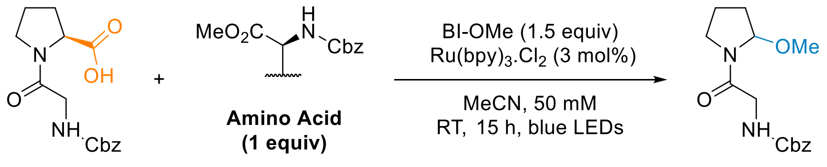

Entry	Amino acid (1 equiv)	HPLC yield (%)
1	Cbz-Met-OMe	15
2	Cbz-Ser-OMe	90[a]
3	Cbz-His-OMe	25
4	Cbz-Arg-OMe	>95
5	Cbz-Tyr-OMe	<5
6	Cbz-Trp-OMe	<5
7	Cbz-Gln-OMe	36
8	Cbz-Lys-OMe	<5
9	Cbz-Asp-OMe	>95
10	Cbz-Cys-OMe	62

[a] + 10% of Serine addition on Z-Gly-Pro (**x**).

5. Scope on dipeptides

General procedure 1 for the oxidative decarboxylation of dipeptides

Degassed MeCN (6 mL) was added in a 10 mL test tube containing the corresponding dipeptide (0.30 mmol, 1.0 equiv), BI-OMe (**2b**) (125 mg, 0.450 mmol, 1.50 equiv) and $Ru(bpy)_3Cl_2 \cdot 6H_2O$ (6.8 mg, 9.0 μmol, 3 mol%) under a nitrogen atmosphere. The reaction mixture was irradiated using blue light LEDs for 15 h at RT.

A solution obtained by dilution of 50 μL of the crude with 950 μL of acetonitrile was injected into the HPLC to monitor the conversion of the starting material.

The crude mixture was diluted with 10 mL of sat. $NaHCO_3$ and extracted with diethyl ether (3 x 30 mL). The combined organic layers were washed with brine (10 mL), dried over $MgSO_4$, filtered and concentrated under vacuum. The crude product was purified by column chromatography DCM to DCM/ethyl acetate on triethylamine deactivated silica (5 vol% in DCM).

General procedure 2 for the oxidative decarboxylation of dipeptides

Degassed MeCN (6 mL) was added in a 10 mL test tube containing the corresponding dipeptide (0.30 mmol, 1.0 equiv), BI-OAc (**2a**) (138 mg, 0.450 mmol, 1.50 equiv), the alcohol (0.60 mmol, 2.0 equiv) and $Ru(bpy)_3Cl_2 \cdot 6H_2O$ (6.8 mg, 9.0 μmol, 3 mol%) under a nitrogen atmosphere. The reaction mixture was irradiated using blue light LEDs for 15 h at RT.

A solution obtained by dilution of 50 μL of the crude with 950 μL of acetonitrile was injected into the HPLC to monitor the conversion of the starting material.

The crude mixture was diluted with 10 mL of sat. $NaHCO_3$ and extracted with diethyl ether (3 x 30 mL). The combined organic layers were washed with brine (10 mL), dried over $MgSO_4$, filtered and concentrated under vacuum. The crude product was purified by column chromatography DCM to DCM/ethyl acetate on triethylamine deactivated silica (5 vol% in DCM).

General procedure 3 for the decarboxylative arylation of dipeptides

Degassed DCE (6 mL) was added in a 10 mL test tube containing the corresponding dipeptide (0.30 mmol, 1.0 equiv), BI-OAc (**2a**) (138 mg, 0.450 mmol, 1.50 equiv) and $Ru(bpy)_3Cl_2 \cdot 6H_2O$ (6.8 mg, 9.0 μmol, 3 mol%) under a nitrogen atmosphere. The reaction mixture was irradiated using blue light LEDs for 15 h at RT.

A solution obtained by dilution of 50 μL of the crude with 950 μL of acetonitrile was injected into the HPLC to monitor the conversion of the starting material.

The phenol (0.45 mmol, 1.5 equiv) was added and the reaction mixture degassed by Ar bubbling before cooling at 0 °C. $BF_3.OEt_2$ (158 μL, 0.600 mmol, 2.00 equiv) was added dropwise and the mixture stirred for 2 h at 0 °C.

The crude mixture was diluted with 10 mL of sat. $NaHCO_3$ and extracted with diethyl ether (3 x 30 mL). The combined organic layers were washed with brine (10 mL), dried over $MgSO_4$, filtered and concentrated under vacuum. The crude product was purified by column chromatography DCM to DCM/ethyl acetate on triethylamine deactivated silica (5 vol% in DCM).

General procedure 4 for the decarboxylative arylation of dipeptides

Degassed MeCN (6 mL) was added in a 10 mL test tube containing the corresponding dipeptide (0.30 mmol, 1.0 equiv), BI-OAc (**2a**) (138 mg, 0.450 mmol, 1.50 equiv) and $Ru(bpy)_3Cl_2{\cdot}6H_2O$ (6.8 mg, 9.0 μmol, 3 mol%) under a nitrogen atmosphere. The reaction mixture was irradiated using blue light LEDs for 15 h at RT.

A solution obtained by dilution of 50 μL of the crude with 950 μL of acetonitrile was injected into the HPLC to monitor the conversion of the starting material.

The indole (0.306 mmol, 1.02 equiv) was added and TFA (23 μL, 0.30 mmol, 1.0 equiv) was added dropwise and the mixture stirred for 1 h at RT.

The crude mixture was diluted with 10 mL of sat. $NaHCO_3$ and extracted with diethyl ether (3 x 30 mL). The combined organic layers were washed with brine (10 mL), dried over $MgSO_4$, filtered and concentrated under vacuum. The crude product was purified by column chromatography DCM to DCM/ethyl acetate on triethylamine deactivated silica (5 vol% in DCM).

Benzyl (2-(2-methoxypyrrolidin-1-yl)-2-oxoethyl)carbamate (4a)

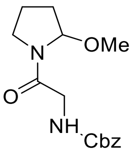

Following General Procedure 1 and starting with Cbz-Gly-Pro (**1a**) (92 mg, 0.30 mmol, 1.0 equiv), **4a** was obtained after column chromatography DCM to DCM/ethyl acetate 8:2 as a pale yellow oil (66 mg, 0.23 mmol, 75%).

Rf (DCM/ethyl acetate 7:3): 0.3. ^{1}H NMR (400 MHz, Chloroform-*d*, 1:1 mixture of rotamers (R^1/R^2)) δ 7.40-7.26 (m, 5H, Ar*H* (R^1+R^2)), 5.70 (s, 1H, N*H* (R^1+R^2)), 5.43 (d, *J* = 4.9 Hz, 0.5H, NC*H*COMe (R^1)), 5.12 (s, 2H, OC*H*$_2$Ph (R^1+R^2)), 4.96 (d, *J* = 4.5 Hz, 0.5H, NC*H*COMe (R^2)), 4.18-4.05 (m, 1H, NC(O)C*H*$_2$NHCbz (R^1)), 4.05-3.91 (m, 1H, NC(O)C*H*$_2$NHCbz (R^2)), 3.67 (ddd, *J* = 11.3, 8.4, 2.3 Hz, 0.5H, C(O)NC*H*$_2$ (R^1)), 3.58-3.48 (m, 0.5H, C(O)NC*H*$_2$ (R^2)), 3.38 (s, 1.5H, OC*H*$_3$ (R^1)), 3.43-3.32 (m, 1H, C(O)NC*H*$_2$ (R^1+R^2)), 3.31 (s, 1.5H, OC*H*$_3$ (R^2)), 2.24-1.66 (m, 4H, NCH$_2$C*H*$_2$C*H*$_2$CH (R^1+R^2)). ^{13}C NMR (101 MHz, Chloroform-*d,* mixture of rotamers, signals not fully resolved) δ 168.3, 168.2, 156.2, 156.2, 136.4, 136.3, 128.4, 128.0, 127.9, 88.2, 87.5, 66.8, 66.8, 56.6, 54.2, 45.8, 44.8, 43.4, 42.9, 31.2, 30.7, 22.7, 20.7. IR (ν_{max}, cm^{-1}) 3324 (m), 2980 (m), 2886 (m), 2339 (w), 1718 (s), 1655 (s), 1520 (m), 1451 (m), 1246 (s), 1170 (m), 1055 (s), 914 (m), 826 (w), 741 (m), 699 (m). HRMS (ESI/QTOF) m/z: $[M + Na]^+$ Calcd for $C_{15}H_{20}N_2NaO_4^+$ 315.1315; Found 315.1315.

Benzyl (2-(2-(allyloxy)pyrrolidin-1-yl)-2-oxoethyl)carbamate (4b)

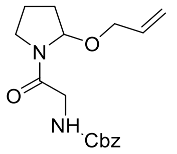

Following General Procedure 2 and starting with Cbz-Gly-Pro (**1a**) (92 mg, 0.30 mmol, 1.0 equiv) and allyl alcohol (41 μL, 0.60 mmol, 2.0 equiv), **4b** was obtained after column chromatography DCM to DCM/ethyl acetate 9:1 as a pale yellow oil (73 mg, 0.23 mmol, 76%).

Rf (DCM/ethyl acetate 7:3): 0.35. ^{1}H NMR (400 MHz, Chloroform-*d*, 6:4 mixture of rotamers (major/minor)) δ 7.42-7.27 (m, 5H, Ar*H* (major+minor)), 5.97-5.81 (m, 1H, $CH_2C\textit{H}{=}CH_2$ (major+minor)), 5.72 (br s, 1H, N*H* (major+minor)), 5.56 (d, *J* = 4.9 Hz, 0.6H, C(O)NC*H* (major)), 5.37-5.20 (m, 1.2H, $CH_2CH{=}C\textit{H}_2$ (major)), 5.17-5.06 (m, 3.2H, $CH_2CH{=}C\textit{H}_2$ (minor), $OC\textit{H}_2Ph$ (major+minor) and C(O)NC*H* (minor)), 4.22-3.86 (m, 4H, $C\textit{H}_2CH{=}CH_2$ and $C(O)C\textit{H}_2NHCbz$ (major+minor)), 3.68 (ddd, *J* = 11.3, 8.6, 2.0 Hz, 0.4H, $C(O)NC\textit{H}_2$ (minor)), 3.57-3.48 (m, 0.6H, $C(O)NC\textit{H}_2$ (major)), 3.44-3.25 (m, 1H, $C(O)NC\textit{H}_2$ (major+minor)), 2.30-1.64 (m, 4H, $NCH_2C\textit{H}_2C\textit{H}_2CH$ (major+minor)). ^{13}C NMR (101 MHz, Chloroform-*d,* mixture of rotamers, signals not fully resolved) δ 168.2, 168.1, 156.2, 156.2, 136.4, 136.4, 136.3, 134.7, 133.5, 128.4, 128.0, 127.9, 117.9, 116.6, 86.8, 86.1, 70.2, 68.0, 66.9, 66.8, 66.8, 45.9, 44.9, 43.4, 43.1, 31.7, 31.5, 22.8, 20.7. IR (ν_{max}, cm^{-1}) 3331 (m), 2982 (m), 2898 (m), 1720 (s), 1657 (s), 1538 (m), 1451 (m), 1247 (s), 1171 (m), 1052 (s), 915 (m), 740 (m). HRMS (ESI/QTOF) m/z: [M + Na]$^+$ Calcd for $C_{17}H_{22}N_2NaO_4^+$ 341.1472; Found 341.1476.

Benzyl (2-oxo-2-(2-(prop-2-yn-1-yloxy)pyrrolidin-1-yl)ethyl)carbamate (4c)

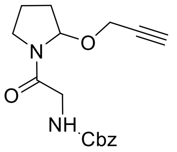

Following General Procedure 2 and starting with Cbz-Gly-Pro (**1a**) (92 mg, 0.30 mmol, 1.0 equiv) and propargyl alcohol (36 μL, 0.60 mmol, 2.0 equiv), **4c** was obtained after column chromatography DCM to DCM/ethyl acetate 10:1 as a pale yellow oil (61 mg, 0.19 mmol, 64%).

Rf (DCM/ethyl acetate 7:3): 0.33. ^{1}H NMR (400 MHz, Chloroform-*d*, 6:4 mixture of rotamers (major/minor)) *δ* 7.39-7.28 (m, 5H, Ar*H* (major+minor)), 5.69 (br s, 1H, N*H* (major+minor)), 5.66 (d, *J* = 4.9 Hz, 0.6H, C(O)NC*H* (major)), 5.31 (d, *J* = 4.1 Hz, 0.4H, C(O)NC*H*O (minor)), 5.12 (s, 2H, $OC\textit{H}_2Ph$ (major+minor)), 4.30 (qd, *J* = 15.7, 2.4 Hz, 1.2H, $OC\textit{H}_2CCH$ (major)), 4.23-4.07 (m, 1.4H, $OC\textit{H}_2CCH$ (minor) and $C(O)C\textit{H}_2NHCbz$ (major)), 4.05-3.90 (m, 1.4H, $C(O)C\textit{H}_2NHCbz$ (major+minor)), 3.71-3.63 (m, 0.4H, $C(O)NC\textit{H}_2$ (minor)), 3.53 (t, *J* = 8.8 Hz, 0.6H, $C(O)NC\textit{H}_2$ (major)), 3.36 (dq, *J* = 27.4, 9.7, 8.7 Hz, 1H, $C(O)NC\textit{H}_2$ (major+minor)), 2.58 (m, 0.4H, $OCH_2CC\textit{H}$ (minor)), 2.42 (t, *J* = 2.36 Hz, 0.6H, $OCH_2CC\textit{H}$ (major)), 2.30-1.70 (m, 4H, $NCH_2C\textit{H}_2C\textit{H}_2CH$ (major+minor)). ^{13}C NMR (101 MHz, Chloroform-*d,* mixture of rotamers, signals not fully resolved) *δ* 168.7, 168.3, 156.4, 136.6, 136.5, 128.6, 128.2, 128.1, 86.3, 86.1, 80.3, 78.7, 75.9, 73.9, 67.1, 57.1, 54.5, 46.1, 45.1, 43.6, 43.5, 32.0, 31.5, 22.9, 20.8. IR (vmax, cm^{-1}) 3416 (w), 3299 (m), 2973 (w), 2889 (w), 2116 (w), 1720 (s), 1662 (s), 1524 (m), 1430 (s), 1254 (s), 1172 (m), 1058 (s), 911 (m), 737 (s), 700 (m). HRMS (ESI/QTOF) m/z: [M + Na]$^+$ Calcd for $C_{17}H_{20}N_2NaO_4^+$ 339.1315; Found 339.1315.

Benzyl (2-(2-(benzyloxy)pyrrolidin-1-yl)-2-oxoethyl)carbamate (4d)

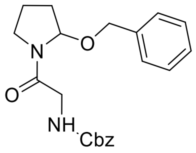

Following General Procedure 2 and starting with Cbz-Gly-Pro (**1a**) (92 mg, 0.30 mmol, 1.0 equiv) and benzyl alcohol (65 μL, 0.60 mmol, 2.0 equiv), **4d** was obtained after column chromatography DCM to DCM/ethyl acetate 20:1 as a pale yellow oil (108 mg, 0.293 mmol, 98%).

Rf (DCM/ethyl acetate 7:3): 0.41. ^{1}H NMR (400 MHz, Chloroform-*d,* 6:4 mixture of rotamers (major/minor)) δ 7.40-7.27 (m, 10H, Ar*H* (major+minor)), 5.74-5.63 (m, 1.6H, N*H* (major+minor) and C(O)NC*H* (major)), 5.20 (d, *J* = 4.1 Hz, 0.4H, C(O)NC*H* (minor)), 5.13 (d, *J* = 2.5 Hz, 2H, C(O)OCH_2Ph (major+minor)), 4.70 (br s, 0.6H, NCHOCH_2Ph (major)), 4.67 (d, *J* = 3.0 Hz, 0.6H, NCHOCH_2Ph (major)), 4.56-4.46 (m, 0.8H, NCHOCH_2Ph (minor)), 4.09 (qd, *J* = 17.0, 4.6 Hz, 0.8H, C(O)CH_2NHCbz (minor)), 3.91 (m, 1.2H, C(O)CH_2NHCbz (major)), 3.74-3.65 (m, 0.4H, C(O)NCH_2 (minor)), 3.49 (t, *J* = 8.9 Hz, 0.6H, C(O)NCH_2 (major)), 3.41 (td, *J* = 11.2, 10.5, 7.0 Hz, 0.4H, C(O)NCH_2 (minor)), 3.31 (q, *J* = 9.6 Hz, 0.6H, C(O)NCH_2 (major)), 2.34-1.67 (m, 4H, NCH$_2$CH_2CH_2CH (major+minor)). ^{13}C NMR (101 MHz, Chloroform-*d,* mixture of rotamers, signals not fully resolved) δ 168.4, 168.3, 156.4, 156.3, 149.3, 141.0, 138.7, 137.0, 136.6, 136.5, 128.8, 128.7, 128.6, 128.4, 128.3, 128.2, 128.1, 128.0, 127.8, 127.6, 127.1, 87.2, 86.5, 71.3, 69.5, 67.1, 67.0, 65.5, 46.1, 45.0, 43.6, 43.3, 32.0, 31.7, 23.0, 21.0. IR (v_{max}, cm^{-1}) 3418 (w), 3329 (w), 2979 (w), 2881 (w), 1721 (s), 1659 (s), 1524 (m), 1452 (m), 1347 (w), 1253 (m), 1172 (m), 1107 (m), 1054 (s), 914 (m), 740 (s). HRMS (ESI/QTOF) m/z: $[M + Na]^+$ Calcd for $C_{21}H_{24}N_2NaO_4^+$ 391.1628; Found 391.1633.

Benzyl (2-(2-(2-cyanoethoxy)pyrrolidin-1-yl)-2-oxoethyl)carbamate (4e)

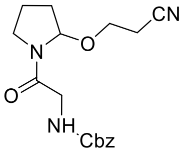

Following General Procedure 2 and starting with Cbz-Gly-Pro (**1a**) (92 mg, 0.30 mmol, 1.0 equiv) and 3-hydroxypropionitrile (41 μL, 0.60 mmol, 2.0 equiv), **4e** was obtained after column chromatography DCM to DCM/ethyl acetate 10:1 as a pale yellow oil (47 mg, 0.14 mmol, 47%).

Rf (DCM/ethyl acetate 7:3): 0.24. ^{1}H NMR (400 MHz, Chloroform-*d,* 8:2 mixture of rotamers (major/minor)) δ 7.39-7.28 (m, 5H, Ar*H* (major+minor)), 5.63 (br s, 1H (major+minor)), 5.55 (d, *J* = 4.9 Hz, 0.8H, C(O)NC*H* (major)), 5.12 (m, 2.2H, OCH_2Ph (major+minor) and C(O)NC*H* (minor)), 4.10 (qd, *J* = 16.9, 4.8 Hz, 0.4H, C(O)CH_2NHCbz (minor)), 4.01-3.91 (m, 1.6H, C(O)CH_2NHCbz (major)), 3.83 (tq, *J* = 7.4, 4.0 Hz, 1.6H, OCH_2CH$_2$CN (major)), 3.69 (dq, *J* = 12.9, 5.3, 3.9 Hz, 0.4H, OCH_2CH$_2$CN (minor)), 3.65-3.61 (m, 0.2H, C(O)NCH_2 (minor)), 3.56 (t, *J* = 9.0 Hz, 0.8H, C(O)NCH_2 (major)), 3.45-3.38 (m, 0.2H, C(O)NCH_2 (minor)), 3.34 (q, *J* = 9.8 Hz, 0.8H, C(O)NCH_2 (major)), 2.63 (t, *J* = 6.4 Hz, 0.4H, OCH$_2$CH_2CN (minor)), 2.55 (m, 1.6H, OCH$_2$CH_2CN (major)), 2.31-1.68 (m, 4H, NCH$_2$CH_2CH_2CHO (major+minor)). ^{13}C NMR (101 MHz, Chloroform-*d,* mixture of rotamers, signals not fully resolved) δ 169.0, 168.3, 156.5, 156.4, 136.5, 128.7, 128.3, 128.2, 118.1, 117.5, 87.5, 86.7, 67.1, 63.9, 61.5, 46.1, 45.9, 45.3, 43.6, 43.2, 32.0, 31.7, 23.0, 20.9, 19.3, 19.2. IR (v_{max}, cm^{-1}) 3407 (w), 3329 (w), 2959 (w),

2887 (w), 2251 (w), 1720 (s), 1660 (s), 1525 (m), 1429 (s), 1336 (m), 1254 (s), 1172 (m), 1085 (s), 1060 (s), 914 (m), 739 (s). HRMS (ESI/QTOF) m/z: $[M + Na]^+$ Calcd for $C_{17}H_{21}N_3NaO_4^+$ 354.1424; Found 354.1422.

Benzyl (2-(2-(3-chloropropoxy)pyrrolidin-1-yl)-2-oxoethyl)carbamate (4f)

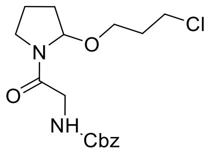

Following General Procedure 2 and starting with Cbz-Gly-Pro (**1a**) (92 mg, 0.30 mmol, 1.0 equiv) and 3-chloropropan-1-ol (25 µL, 0.60 mmol, 2.0 equiv), **4f** was obtained after column chromatography DCM to DCM/ethyl acetate 10:1 as a pale yellow oil (74 mg, 0.21 mmol, 70%).

Rf (DCM/ethyl acetate 7:3): 0.29. ^{1}H NMR (400 MHz, Chloroform-*d,* 6:4 mixture of rotamers (major/minor)) δ 7.38-7.28 (m, 5H, Ar*H* (major+minor)), 5.75-5.63 (m, 1H, N*H* (major+minor)), 5.51 (d, *J* = 4.86 Hz, 0.6H, C(O)NC*H* (major)), 5.12 (s, 2H, OCH_2Ph (major+minor)), 5.07 (d, *J* = 4.2 Hz, 0.4H C(O)NC*H* (minor)), 4.18-4.01 (m, 1H, C(O)CH_2NHCbz (major+minor)), 3.96 (dt, *J* = 17.4, 4.5 Hz, 1H, C(O)CH_2NHCbz (major+minor)), 3.72-3.49 (m, 5H, $OCH_2CH_2CH_2Cl$, $OCH_2CH_2CH_2Cl$ and C(O)NCH_2, (major+minor)), 3.34 (dq, *J* = 26.5, 9.7, 8.6 Hz, 1H, C(O)NCH_2 (major+minor)), 2.33-1.55 (m, 6H, $NCH_2CH_2CH_2CH$ and $OCH_2CH_2CH_2Cl$ (major+minor)). ^{13}CNMR (101 MHz, Chloroform-*d,* mixture of rotamers, signals not fully resolved) δ 168.4, 168.3, 156.4, 136.5, 128.6, 128.2, 128.1, 87.2, 86.5, 67.0, 65.2, 62.9, 46.1, 45.1, 43.6, 43.2, 42.0, 41.7, 32.7, 32.3, 31.7, 31.6, 23.0, 21.0. IR (ν_{max}, cm^{-1}) 3415 (w), 3319 (w), 2959 (m), 2882 (w), 1721 (s), 1660 (s), 1524 (m), 1430 (s), 1246 (m), 1171 (m), 1059 (s), 996 (m), 911 (m), 738 (s), 701 (m). HRMS (ESI/QTOF) m/z: $[M + Na]^+$ Calcd for $C_{17}H_{23}ClN_2NaO_4^+$ 377.1239; Found 377.1232.

Benzyl (2-(2-(3-azidopropoxy)pyrrolidin-1-yl)-2-oxoethyl)carbamate (4g)

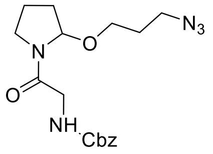

Following General Procedure 2 and starting with Cbz-Gly-Pro (**1a**) (92 mg, 0.30 mmol, 1.0 equiv) and 3-azidopropan-1-ol (600 µL, 1M in DCM, 0.60 mmol, 2.0 equiv), **4g** was obtained after column chromatography DCM to DCM/ethyl acetate 10:1 as a pale yellow oil (99 mg, 0.27 mmol, 91%).

Rf (DCM/ethyl acetate 7:3): 0.38. ^{1}H NMR (400 MHz, Chloroform-*d,* 6:4 mixture of rotamers (major/minor)) δ 7.38-7.28 (m, 5H, Ar*H* (major+minor)), 5.75-5.63 (m, 1H, N*H* (major+minor)), 5.50 (d, *J* = 4.9 Hz, 0.6H, C(O)NC*H* (major)), 5.12 (s, 2H, OCH_2Ph (major+minor)), 5.05 (d, *J* = 4.2 Hz, 0.4H, C(O)NC*HO* (minor)), 4.16-4.01 (m, 0.8H, C(O)CH_2NHCbz (minor)), 4.01-3.91 (m, 1.2H, C(O)CH_2NHCbz (major)), 3.76 (t, *J* = 4.7 Hz, 0.8H, $OCH_2CH_2CH_2N_3$ (minor)), 3.65 (qd, *J* = 6.1, 5.1, 1.8 Hz, 1.2H, $OCH_2CH_2CH_2N_3$ (major)), 3.56-3.26 (m, 4H, $OCH_2CH_2CH_2N_3$ and (CO)NCH_2 (major+minor)), 2.24-1.66 (m, 6H, $NCH_2CH_2CH_2CHO$ and $OCH_2CH_2CH_2N_3$ (major+minor)). ^{13}C NMR (101 MHz, Chloroform-*d,* mixture of rotamers, signals not fully resolved) δ 168.4, 168.2, 156.4, 136.5, 128.6, 128.2, 128.1, 87.3, 86.6, 67.0, 65.7, 63.4, 60.1,

48.6, 48.5, 48.4, 46.0, 45.1, 43.6, 43.2, 31.8, 31.6, 31.5, 29.4, 29.2, 23.0, 21.0. IR (v_{max}, cm^{-1}) 3418 (w), 3318 (w), 2952 (m), 2881 (m), 2097 (s), 1721 (s), 1661 (s), 1523 (m), 1452 (s), 1256 (s), 1172 (m), 1056 (s), 912 (m), 739 (s). HRMS (ESI/QTOF) m/z: $[M + Na]^+$ Calcd for $C_{17}H_{23}N_5NaO_4^+$ 384.1642; Found 384.1646.

Benzyl (2-(2-(((1R,2S,5R)-2-isopropyl-5-methylcyclohexyl)oxy)pyrrolidin-1-yl)-2-oxoethyl)carbamate (4h)

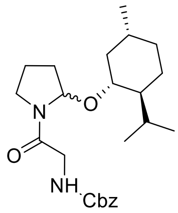

Following General Procedure 2 and starting with Cbz-Gly-Pro (**1a**) (92 mg, 0.30 mmol, 1.0 equiv) and (1R,2S,5R)-(-)-menthol (94 mg, 0.60 mmol, 2.0 equiv), **4h** was obtained after column chromatography DCM to DCM/ethyl acetate 15:1 as a pale yellow oil as an unresolved mixture of diastereoisomers (44 mg, 0.11 mmol, 35%).

Rf (DCM/ethyl acetate 7:3): 0.63. ^1H NMR (400 MHz, Chloroform-*d*, unresolved mixture of diastereoisomers and rotamers) δ 7.39-7.28 (m, 5H, Ar*H*), 5.79-5.71 (m, 0.7H, N*H* and C(O)NC*H*), 5.66 (br s, 0.4H, N*H*), 5.58 (m, 0.5H, N*H* and C(O)NC*H*), 5.26 (d, *J* = 4.16 Hz, 0.4H, C(O)NC*H*), 5.19-5.07 (m, 2H, OCH_2Ph), 4.22-3.82 (m, 2H, $C(O)CH_2NHCbz$), 3.69 (t, *J* = 9.3 Hz, 0.3H, $C(O)NCH_2$), 3.54 (t, *J* = 9.3 Hz, 0.3H, $C(O)NCH_2$), 3.51-3.19 (m, 2H, $C(O)NCH_2$ and NCHOC*H*), 3.12 (tt, *J* = 11.5, 5.7 Hz, 0.3H, $C(O)NCH_2$), 2.32-1.83 (m, 6H, $NCH_2CH_2CH_2CH$ and *H*menthol), 1.79-1.61 (m, 4H, $NCH_2CH_2CH_2CH$ and *H*menthol), 1.43-1.07 (m, 3H, *H*menthol), 1.06-0.61 (m, 13H, *H*menthol). ^{13}C NMR (101 MHz, Chloroform-*d,* mixture of diastereoisomers and rotamers, signals not fully resolved) δ 168.3, 168.0, 167.7, 156.5, 156.4, 156.3, 136.6, 136.5, 128.6, 128.2, 128.1, 128.0, 85.7, 83.2, 82.8, 77.8, 75.7, 74.9, 67.0, 66.9, 48.8, 48.3, 48.1, 46.0, 45.0, 44.7, 43.7, 43.5, 43.4, 41.8, 41.7, 40.8, 39.7, 34.7, 34.6, 34.4, 32.5, 31.8, 31.7, 31.6, 31.5, 31.4, 25.7, 25.5, 25.2, 25.1, 23.3, 23.2, 23.1, 22.9, 22.5, 22.4, 21.4, 21.3, 21.1, 20.8, 16.2, 16.1, 15.9. IR (v_{max}, cm^{-1}) 3417 (w), 3315 (w), 2954 (m), 2871 (m), 1724 (s), 1661 (s), 1523 (m), 1428 (s), 1242 (m), 1173 (m), 1095 (m), 1049 (s), 915 (m), 736 (s). HRMS (ESI/QTOF) m/z: $[M + Na]^+$ Calcd for $C_{24}H_{36}N_2NaO_4^+$ 439.2567; Found 439.2567.

(2S)-Methyl 2-(((benzyloxy)carbonyl)amino)-3-((1-(2-(((benzyloxy)carbonyl)amino)acetyl)pyrrolidin-2-yl)oxy)propanoate (4i)

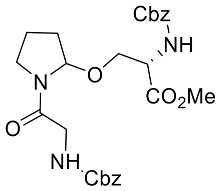

Following General Procedure 2 and starting with Cbz-Gly-Pro (**1a**) (92 mg, 0.30 mmol, 1.0 equiv) and Cbz-Ser-OMe (114 mg, 0.450 mmol, 1.50 equiv), **4i** was obtained after column chromatography DCM to DCM/ethyl acetate 1:1 as a pale yellow oil and a mixture of diastereoisomers (65 mg, 0.13 mmol, 42%).

Rf (DCM/ethyl acetate 7:3): 0.3. ^{1}H NMR (400 MHz, Chloroform-*d*, mixture of rotamers of diastereoisomers)[1] δ 7.40-7.25 (m, 10H, Ar*H*), 6.05 (d, 0.3H, N*H* Ser), 5.93 (d, *J* = 8.4 Hz, 0.1H, N*H* Ser), 5.72 (br s, 1H, N*H* Gly), 5.65 (s, 0.4H, N*H* Ser), 5.47 (d, *J* = 4.8 Hz, 0.3H, C(O)NC*H* Pro), 5.41 (d, *J* = 4.9 Hz, 0.4H, C(O)NC*H* Pro), 5.12 (s, 4H, OCH_2Ph Gly+Ser), 5.09-5.05 (m, 0.3H, C(O)NC*H* Pro), 4.59-4.49 (m, 0.3H, NHC*H* Ser), 4.51-4.41 (m, 0.7H, NHC*H* Ser), 4.12-3.78 (m, 4H, OCH_2 Ser and C(O)CH_2NHCbz Gly), 3.77-3.62 (m, 3H, CO_2CH_3 Ser), 3.59-3.19 (m, 2H, C(O)NCH_2 Pro), 2.19-1.61 (m, 4H, $NCH_2CH_2CH_2CH$ Pro). ^{13}C NMR (101 MHz, Chloroform-*d,* mixture of rotamers of diastereoisomers, signals not fully resolved) δ 170.8, 170.7, 170.2, 168.5, 168.2, 156.2, 155.9, 136.3, 136.3, 136.2, 128.5, 128.4, 128.2, 128.1, 128.1, 128.0, 128.0, 127.9, 87.6, 87.2, 87.0, 86.7, 68.9, 68.6, 67.1, 67.0, 66.9, 66.3, 66.2, 54.6, 54.4, 54.3, 54.0, 52.8, 52.7, 52.5, 52.4, 45.9, 45.1, 44.9, 43.4, 43.4, 42.9, 42.8, 33.7, 31.5, 31.4, 31.1, 31.1, 22.7, 22.7, 20.7, 20.6, 20.4. IR (ν_{max}, cm^{-1}) 3324 (m), 2979 (m), 2885 (w), 1718 (s), 1658 (s), 1518 (m), 1436 (m), 1242 (m), 1054 (s), 914 (m), 738 (s), 699 (m). HRMS (ESI/QTOF) m/z: [M + Na]$^+$ Calcd for $C_{26}H_{31}N_3NaO_8^+$ 536.2003; Found 536.2014.

(2S,3R)-methyl 2-(((benzyloxy)carbonyl)amino)-3-((1-(2-(((benzyloxy)carbonyl)amino) acetyl)pyrrolidin-2-yl)oxy)butanoate (4j)

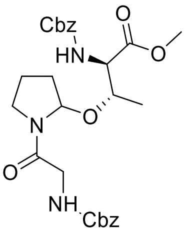

Following General Procedure 2 and starting with Cbz-Gly-Pro (**1a**) (92 mg, 0.30 mmol, 1.0 equiv) and Cbz-Thr-OMe (160 mg, 0.450 mmol, 1.50 equiv), **4j** was obtained after column chromatography DCM to DCM/ethyl acetate 1:1 as a pale yellow oil and a mixture of diastereoisomers (60 mg, 0.11 mmol, 38%).

Rf (DCM/ethyl acetate 7:3): 0.36. ^{1}H NMR (400 MHz, Chloroform-*d*, mixture of rotamers of diastereoisomers)[1] δ 7.40-7.28 (m, 10H, Ar*H*), 5.67 (d, *J* = 14.7 Hz, 1H, N*H* Gly), 5.60 (d, *J* = 9.4 Hz, 0.5H, N*H* Thr), 5.55 (d, *J* = 4.7 Hz, 0.6H, C(O)NC*H* Pro), 5.54-5.48 (m, 0.25H, N*H* Thr), 5.43 (d, *J* = 9.7 Hz, 0.25H, N*H* Thr), 5.40 (d, *J* = 4.7 Hz, 0.4H, C(O)NC*H* Pro), 5.17-5.06 (m, 4H, OCH_2Ph Gly and Ser), 4.54-4.37 (m, 0.5H, OC*H* and NHC*H* Thr), 4.37-4.22 (m, 1.2H, OC*H* and NHC*H* Thr), 4.22-4.11 (m, 0.3H, OC*H* and NHC*H* Thr), 3.96 (m, 2H, C(O)CH_2NHCbz Gly), 3.78-3.58 (m, 3H, OCH_3 Thr), 3.47 (dq, *J* = 16.4, 9.5, 8.9 Hz, 0.5H, C(O)NCH_2 Pro), 3.40-3.19 (m, 1.5H, C(O)NCH_2 Pro), 2.15-1.68 (m, 4H, $NCH_2CH_2CH_2CH$ Pro), 1.39-1.16 (m, 3H, OCHCH_3 Thr). ^{13}C NMR (101 MHz, Chloroform-*d*, mixture of diastereoisomers and rotamers, signals not fully resolved) δ 171.6, 171.4, 168.7, 168.0, 156.8, 156.7, 156.3, 136.5, 136.4, 136.3, 128.7, 128.6, 128.3, 128.2, 128.1, 86.7, 84.4, 83.9, 74.7, 72.3, 71.3, 67.4, 67.3, 67.2, 67.1, 59.1, 59.0, 52.7, 52.5, 52.4, 46.1, 45.7, 45.0, 44.8, 43.6, 43.5, 43.2, 32.2, 32.1, 31.7, 31.3, 22.9, 22.8, 20.5, 20.4, 17.8, 16.9, 16.0. IR (ν_{max}, cm^{-1}) 3365 (w), 2979 (w), 2890 (w), 2099 (w), 1719 (s), 1663 (m), 1523 (m), 1436 (m), 1318 (m), 1256 (m), 1173 (m), 1065 (s), 1002 (m), 911 (m), 739 (s). HRMS (ESI/QTOF) m/z: [M + Na]$^+$ Calcd for $C_{27}H_{33}N_3NaO_8^+$ 550.2160; Found 550.2161.

Benzyl ((2S)-1-((1-methoxyethyl)amino)-1-oxopropan-2-yl)carbamate (4k)

[1] Due to the complexity of the mixture, signals were not attributed to each rotamer and diastereoisomer.

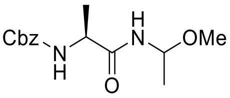

Following General Procedure 1 and starting with Cbz-Ala-Ala (**1b**) (88 mg, 0.30 mmol, 1.0 equiv), **4k** was obtained after column chromatography DCM to DCM/ethyl acetate 8:2 as a white amorphous solid (57 mg, 0.20 mmol, 68%) and as a mixture of unresolved diastereoisomers.

Rf (DCM/ethyl acetate 7:3): 0.25. ^{1}H NMR (400 MHz, Chloroform-*d*, mixture of diastereomers and rotamers) δ ^{1}H NMR (400 MHz, $CDCl_3$) δ 7.40-7.28 (m, 5H, Ar*H*), 6.30 (br s, 1H, N*H*), 5.33 (s, 1H, N*H*Cbz), 5.24 (dqd, *J* = 9.2, 5.9, 3.3 Hz, 1H, C(O)NC*H*COMe), 5.12 (s, 2H, OC*H*$_2$Ph), 4.22 (q, *J* = 6.8 Hz, 1H, CbzNC*H*Me), 3.30 (s, 1H, OC*H*$_3$), 3.27 (s, 2H, OC*H*$_3$), 1.40 (t, *J* = 7.3 Hz, 3H, CbzNCHC*H*$_3$), 1.30 (d, *J* = 5.9 Hz, 3H, C(O)NHCHC*H*$_3$). δ ^{13}C NMR (101 MHz, Chloroform-*d,* mixture of rotamers, signals not fully resolved) δ 172.3, 156.0, 136.0, 128.6, 128.3, 128.1, 78.4, 67.2, 55.6, 50.7, 28.9, 21.5, 18.7, 18.3. IR (v_{max}, cm^{-1}) 3286 (m), 2981 (w), 1688 (s), 1651 (s), 1540 (s), 1453 (m), 1382 (w), 1324 (m), 1261 (s), 1135 (m), 1070 (m), 914 (m), 738 (s), 699 (s). HRMS (ESI/QTOF) m/z: [M + Na]$^+$ Calcd for $C_{14}H_{20}N_2NaO_4^+$ 303.1315; Found 303.1315.

Benzyl (2-((1-(3-azidopropoxy)-2-phenylethyl)amino)-2-oxoethyl)carbamate (4l)

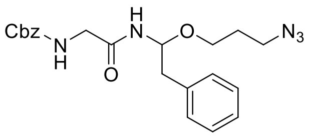

Following General Procedure 2 and starting with Cbz-Gly-Phe (**1c**) (107 mg, 0.30 mmol, 1.0 equiv) and 3-azidopropan-1-ol (600 μL, 1M in DCM, 0.60 mmol, 2.0 equiv), **4l** was obtained after column chromatography DCM to DCM/ethyl acetate 10:1 as a colourless oil (57 mg, 0.14 mmol, 46%).

Rf (DCM/ethyl acetate 7:3): 0.52. ^{1}H NMR (400 MHz, Acetonitrile-*d*$_3$, 75:25 mixture of rotamers (major/minor)) δ 7.41-7.21 (m, 10H, Ar*H* (major+minor)), 6.88 (d, *J* = 9.4 Hz, 1H, N*H* Phe (major+minor)), 6.02 (m, 0.25H, N*H* Gly (minor)), 5.90 (m, 0.75H, N*H* Gly (major)), 5.28 (dt, *J* = 9.6, 6.3 Hz, 1H, NHC*H* Phe (major+minor)), 5.09 (d, *J* = 9.8 Hz, 2H, OC*H*$_2$Ph (major+minor)), 3.81 (d, *J* = 6.1 Hz, 0.5H, NHC*H*$_2$ Gly (minor)), 3.66 (dd, *J* = 6.1, 2.8 Hz, 1.5H, NHC*H*$_2$ Gly (major)), 3.60-3.51 (m, 1H, OC*H*$_2$CH$_2$CH$_2$N$_3$ (major+minor)), 3.42-3.35 (m, 1.5H, OC*H*$_2$CH$_2$CH$_2$N$_3$ (major+minor) and OCH$_2$CH$_2$C*H*$_2$N$_3$ (minor)), 3.23 (t, *J* = 6.7 Hz, 1.5H, OCH$_2$CH$_2$C*H*$_2$N$_3$ (major)), 2.94 (dd, *J* = 13.8, 6.2 Hz, 1H, NHCHC*H*$_2$ Phe (major+minor)), 2.83 (dd, *J* = 13.7, 6.3 Hz, 1H, NHCHC*H*$_2$ Phe (major+minor)), 1.77-1.63 (m, 2H, OCH$_2$CH$_2$C*H*$_2$N$_3$ (major+minor)). ^{13}C NMR (101 MHz, Acetonitrile-*d*$_3$, mixture of rotamers, signals not fully resolved) δ 170.5, 168.2, 157.6, 138.1, 138.0, 137.5, 130.6, 129.6, 129.5, 129.2, 128.9, 128.8, 127.4, 126.4, 123.6, 113.5, 81.1, 67.4, 67.3, 65.2, 59.4, 49.1, 44.8, 41.9, 32.4, 29.6. IR (v_{max}, cm^{-1}) 3347 (m), 3064 (m), 2935 (m), 2880 (m), 2097 (s), 1708 (s), 1664 (s), 1517 (s), 1455 (m), 1257 (s), 1155 (m), 1050 (s), 909 (m), 738 (s), 699 (s). HRMS (ESI/QTOF) m/z: [M + Na]$^+$ Calcd for $C_{21}H_{25}N_5NaO_4^+$ 434.1799; Found 434.1801

Benzyl (2S)-2-((1-(benzyloxy)-2-methylpropyl)carbamoyl)pyrrolidine-1-carboxylate (4m)

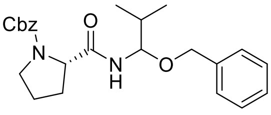

Following General Procedure 2 and starting with Cbz-Pro-Val (**1d**) (105 mg, 0.30 mmol, 1.0 equiv) and benzyl alcohol (65 μL, 0.60 mmol, 2.0 equiv), **4m** was obtained after column chromatography DCM to DCM/ethyl acetate 15:1 as a colourless oil (63 mg, 0.15 mmol, 51%).

Rf (DCM/ethyl acetate 7:3): 0.71. ^{1}H NMR (400 MHz, Acetonitrile-*d*$_3$, mixture of rotamers of diastereoisomers) δ 7.45-7.16 (m, 10H, Ar*H*), 6.97-6.70 (m, 1H, N*H*), 5.18-5.05 (m, 1.5H, OC*H*$_2$Ph Cbz), 4.99 (d, *J* = 13.3 Hz, 0.5H, OC*H*$_2$Ph Cbz), 4.91 (m, 1H, NHC*H* Val), 4.54 (m, 0.5H, OC*H*$_2$Ph Val), 4.49-4.33 (m, 1H, OC*H*$_2$Ph Val), 4.26 (m, 1.5H, OC*H*$_2$Ph Val and CbzNC*H* Pro), 3.59-3.40 (m, 2H, CbzNC*H*$_2$ Pro), 2.33-2.19 (m, 1H, CbzNCH$_2$C*H*$_2$C*H*$_2$CH Pro), 2.05-1.95 (m, 1H, CbzNCH$_2$C*H*$_2$C*H*$_2$CH Pro), 1.92-1.73 (m, 3H, CbzNCH$_2$C*H*$_2$C*H*$_2$CH Pro and NHCHC*H* Val), 0.98-0.72 (m, 6H, C*H*$_3$ Val). ^{13}C NMR (101 MHz, Acetonitrile-*d*$_3$, mixture of rotamers of diastereoisomers, signals not fully resolved) δ 174.1, 173.8, 173.7, 156.2, 155.5, 139.8, 139.7, 138.1, 129.4, 129.3, 129.2, 128.9, 128.7, 128.6, 128.4, 128.3, 84.5, 84.3, 70.1, 67.5, 67.4, 62.1, 62.0, 61.6, 61.4, 48.3, 47.8, 33.7, 32.5, 30.8, 25.3, 24.3, 18.6, 18.0, 17.9, 17.8. IR (ν_{max}, cm^{-1}) 3338 (m), 3063 (m), 2929 (m), 1672 (s), 1529 (s), 1417 (s), 1356 (s), 1231 (m), 1119 (s), 1089 (m), 1039 (m), 912 (m), 737 (s), 697 (s). HRMS (ESI/QTOF) m/z: [M + Na]$^+$ Calcd for $C_{24}H_{30}N_2NaO_4^+$ 433.2098; Found 433.2090.

Benzyl (2-(2-(2-hydroxy-5-methylphenyl)pyrrolidin-1-yl)-2-oxoethyl)carbamate (5a)

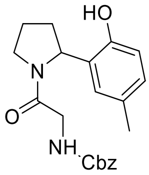

Following General Procedure 3 and starting with Cbz-Gly-Pro (**1a**) (92 mg, 0.30 mmol, 1.0 equiv) and *p*-cresol (49 mg, 0.45 mmol, 1.5 equiv), **5a** was obtained after column chromatography DCM to DCM/ethyl acetate 7:3 as a pale yellow amorphous solid (68 mg, 0.19 mmol, 62%).

Rf (DCM/ethyl acetate 7:3): 0.4. ^{1}H NMR (400 MHz, Chloroform-*d*, 7:3 mixture of rotamers (major/minor)) δ 8.94 (s, 1H, O*H* (major+minor)), 7.33 (dt, *J* = 9.1, 4.6 Hz, 5H, Ar*H* (major+minor)), 6.94 (d, *J* = 8.1 Hz, 0.7H, Ar*H* (major)), 6.89 (s, 0.7H, Ar*H* (major)), 6.82 (d, *J* = 7.6 Hz, 0.3H, Ar*H* (minor)), 6.76 (d, *J* = 8.1 Hz, 0.7H, Ar*H* (major)), 6.66 (s, 0.3H, Ar*H* (minor)), 6.51 (d, *J* = 8.0 Hz, 0.3H, Ar*H* (minor)), 5.79 (br s, 0.3H, N*H* (minor)), 5.67 (br s, 0.7H, N*H* (major)), 5.39 (dd, *J* = 7.5, 3.0 Hz, 0.7H, C(O)NC*H* (major)), 5.17 (d, *J* = 7.7 Hz, 0.3H, C(O)NC*H* (minor)), 5.11 (s, 1.4H, OC*H*$_2$Ph (major)), 5.07 (s, 0.6H, OC*H*$_2$Ph (minor)), 4.07 (td, *J* = 20.3, 18.9, 4.6 Hz, 1H, C(O)C*H*$_2$NHCbz (major+minor)), 3.92 (dd, *J* = 17.4, 4.0 Hz, 0.7H, C(O)C*H*$_2$NHCbz (major)), 3.79 (d, *J* = 6.0 Hz, 0.3H, C(O)NC*H*$_2$ (minor)), 3.73 (d, *J* = 8.1 Hz, 0.3H, C(O)NC*H*$_2$ (minor)), 3.67-3.51 (m, 1.4H, C(O)NC*H*$_2$ (major)), 3.43 (dd, *J* = 17.2, 3.3 Hz, 0.3H, C(O)C*H*$_2$NHCbz (minor)), 2.43-2.14 (m, 6H, NCH$_2$C*H*$_2$C*H*$_2$CH and C*H*$_3$ (major+minor)), 1.98-1.82 (m, 1H, NCH$_2$C*H*$_2$C*H*$_2$CH (major+minor)). ^{13}C NMR (101 MHz, Chloroform-*d*, mixture of rotamers, signals not fully resolved) δ 167.4, 156.6, 156.4, 153.1, 150.7, 136.4, 129.9, 129.6, 129.0, 128.6, 128.3, 128.1, 127.8, 127.4, 126.5, 125.9, 118.4, 115.6, 67.1, 55.9, 55.2, 47.5, 46.2, 43.6, 43.3, 34.4, 31.2, 30.5, 25.2, 21.8, 21.0, 20.9. IR (ν_{max}, cm^{-1}) 3313 (w), 2978 (m), 2899 (w), 2360 (w), 1714 (s), 1640 (s), 1510 (m), 1436 (m), 1269 (m), 1055 (m), 738 (m), 699 (m). HRMS (ESI/QTOF) m/z: [M + Na]$^+$ Calcd for $C_{21}H_{22}N_2NaO_4^+$ 389.1472; Found 389.1472.

Benzyl (2-(2-(2-hydroxy-5-methoxyphenyl)pyrrolidin-1-yl)-2-oxoethyl)carbamate (5b)

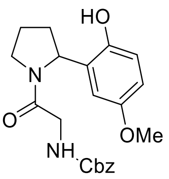

Following General Procedure 3 and starting with Cbz-Gly-Pro (**1a**) (92 mg, 0.30 mmol, 1.0 equiv) and 4-methoxyphenol (56 mg, 0.45 mmol, 1.5 equiv), **5b** was obtained after column chromatography DCM to DCM/ethyl acetate 7:3 as a pale yellow oil (90 mg, 0.23 mmol, 78%).

Rf (DCM/ethyl acetate 7:3): 0.35. ^{1}H NMR (400 MHz, Chloroform-*d*, 7:3 mixture of rotamers (major/minor)) δ 8.73 (s, 1H, O*H* (major+minor)), 7.38-7.28 (m, 5H, Ar*H* (major+minor)), 6.78 (d, *J* = 8.7 Hz, 0.7H, Ar*H* (major)), 6.68 (dd, *J* = 8.7, 2.9 Hz, 0.7H, Ar*H* (major)), 6.65 (d, *J* = 2.8 Hz, 0.7H Ar*H* (major)), 6.57-6.50 (m, 0.6H, Ar*H* (minor)), 6.45 (d, *J* = 2.1 Hz, 0.3H,Ar*H* (minor)), 5.81 (s, 0.3H, N*H* (minor)), 5.66 (s, 0.7H, N*H* (major)), 5.41-5.35 (m, 0.7H, C(O)NC*H* (major)), 5.16 (d, *J* = 7.1 Hz, 0.3H, C(O)NC*H* (minor)), 5.11 (s, 1.4H, OCH_2Ph (major)), 5.08 (s, 0.6H, OCH_2Ph (minor)), 4.16-4.00 (m, 1H, C(O)CH_2NHCbz (major+minor)), 3.93 (dd, *J* = 17.4, 4.2 Hz, 0.7H, C(O)CH_2NHCbz (major)), 3.74 (s, 2.1H, OCH_3 (major)), 3.70 (s, 0.9H, OCH_3 (minor)), 3.62 (dd, *J* = 11.1, 7.1 Hz, 1H, C(O)NCH_2 (major+minor)), 3.59-3.53 (m, 1H, C(O)NCH_2 (major+minor)), 3.46 (dd, *J* = 17.1, 3.6 Hz, 0.3H, C(O)CH_2NHCbz (minor)), 2.40-2.11 (m, 3H, NCH$_2$CH_2CH_2CH (major+minor)), 1.99-1.82 (m, 1H, NCH$_2$CH_2CH_2CH (major+minor)). ^{13}C NMR (101 MHz, Chloroform-*d*, mixture of rotamers, signals not fully resolved) δ 168.0, 167.2, 156.5, 156.3, 153.5, 153.4, 149.2, 147.0, 136.4, 129.2, 128.7, 128.6, 128.3, 128.1, 118.9, 116.3, 113.4, 112.7, 112.6, 111.8, 67.1, 56.0, 55.9, 55.4, 47.5, 46.3, 43.6, 43.3, 34.3, 31.2, 25.1, 21.7. IR (ν_{max}, cm^{-1}) 3671 (m), 3320 (m), 2987 (s), 2899 (m), 1717 (s), 1638 (s), 1507 (s), 1453 (s), 1434 (s), 1350 (m), 1286 (s), 1207 (s), 1045 (s), 815 (m), 740 (m). HRMS (ESI/QTOF) m/z: [M + H]$^+$ Calcd for $C_{21}H_{25}N_2O_5^+$ 385.1758; Found 385.1747.

and Benzyl (2-(2-((8S,9R,13R,14R)-3-hydroxy-13-methyl-17-oxo-7,8,9,11,12,13,14,15,16,17-decahydro-6H-cyclopenta[a]phenanthren-2-yl)pyrrolidin-1-yl)-2-oxoethyl) carbamate and Benzyl (2-(2-((8S,9R,13R,14R)-3-hydroxy-13-methyl-17-oxo-7,8,9,11,12,13,14,15,16,17-decahydro-6H-cyclopenta[a]phenanthren-4-yl)pyrrolidin-1-yl)-2-oxoethyl)carbamate (5c)

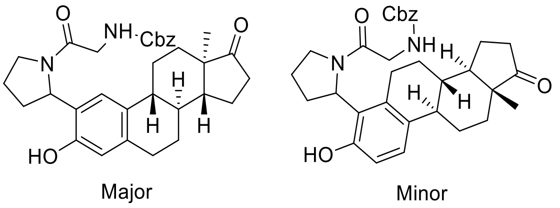

Following General Procedure 3 and starting with Cbz-Pro-Gly (**1a**) (92 mg, 0.30 mmol, 1.0 equiv) and estrone (122 mg, 0.450 mmol, 1.50 equiv), **5c** was obtained after column chromatography DCM to DCM/ethyl acetate 7:3 as a pale yellow amorphous solid (100 mg, 0.188 mmol, 63%, ratio major/minor 7:3).

Rf (DCM/ethyl acetate 7:3): 0.32.

Major: ^{1}H NMR (400 MHz, Acetonitrile-d_3, unresolved mixture of rotamers of diastereoisomers) δ 7.31 (m, 5H, Ar*H*), 7.23-7.10 (m, 0.4H, O*H*), 7.10-6.95 (m, 1H, Ar*H*), 6.89-6.73 (m, 0.6H, O*H*), 6.68-6.48 (m, 1H, Ar*H*), 5.89-5.59 (m, 1H, N*H* Gly), 5.17 (m, 1H, C(O)NC*H*), 5.01 (m, 2H, OCH_2Ph), 4.00-3.40 (m, 4H, C(O)CH_2NHCbz and C(O)NCH_2), 3.29-2.97 (m, 1H, ArCH_2 estrone), 2.90-2.60 (m, 1H ArCH_2 estrone), 2.48-2.26 (m, 3H), 2.04 (m, 5H), 1.90-1.73 (m, 2H), 1.67-1.25 (m, 7H), 0.86 (m, 3H, CH_3). ^{13}C NMR (101 MHz, Acetonitrile-d_3, mixture of

rotamers of diastereoisomers, signals not fully resolved) δ 166.8, 157.1, 153.2, 153.0, 138.2, 136.5, 132.5, 129.4, 128.8, 128.7, 127.3, 126.4, 125.7, 125.5, 114.8, 114.6, 79.3, 78.6, 67.1, 67.0, 56.8, 56.5, 51.2, 48.7, 48.5, 48.1, 47.3, 47.1, 45.3, 45.1, 44.9, 43.9, 43.8, 43.2, 39.4, 38.3, 36.4, 34.0, 32.6, 32.4, 31.4, 30.4, 29.8, 27.6, 27.4, 27.2, 27.1, 26.8, 26.6, 25.0, 24.8, 22.1, 14.3, 14.2. IR (v_{max}, cm^{-1}) 3305 (m), 2929 (m), 2867 (m), 1726 (s), 1633 (s), 1509 (s), 1451 (s), 1372 (m), 1281 (s), 1257 (s), 1056 (s), 831 (m), 741 (m), 698 (s). HRMS (nanochip-ESI/LTQ-Orbitrap) m/z: $[M + H]^+$ Calcd for $C_{32}H_{39}N_2O_5^+$ 531.2853; Found 531.2840

Minor: ^1H NMR (400 MHz, Acetonitrile-d_3, unresolved mixture of rotamers of diastereoisomers) δ 8.26-8.17 (m, 0.2H, O*H*), 8.04-7.93 (m, 0.4H, O*H*), 7.32 (m, 5H Ar*H*), 6.99-6.90 (m, 0.6H, Ar*H*), 6.82 (s, 0.4H, Ar*H*), 6.58-6.45 (m, 1H, Ar*H*), 5.89-5.59 (m, 1H, N*H* Gly), 5.27-5.09 (m, 1H, C(O)NC*H*), 5.03 (m, 2H, OCH_2Ph), 4.00-3.85 (m, 1.6H, C(O)CH_2NHCbz), 3.84-3.74 (m, 0.4H, C(O)CH_2NHCbz), 3.73-3.46 (m, 2H,C(O)NCH_2), 2.88-2.66 (m, 2H, ArCH_2 estrone), 2.47-2.25 (m, 3H), 2.13-1.97 (m, 5H), 1.87-1.72 (m, 2H), 1.67-1.33 (m, 7H), 0.88 (m, 3H, CH_3). ^{13}C NMR (101 MHz, Acetonitrile-d_3, mixture of rotamers of diastereoisomers, signals not fully resolved) δ 174.0, 168.6, 153.0, 152.0, 138.3, 137.8, 132.2, 129.4, 129.3, 128.8, 128.7, 127.3, 127.1, 124.1, 123.8, 116.3, 67.1, 56.4, 51.1, 48.7, 47.9, 47.0, 44.9, 44.8, 44.0, 43.8, 39.7, 39.4, 39.2, 36.3, 35.0, 34.5, 32.6, 32.3, 32.1, 31.1, 29.8, 29.6, 27.3, 26.9, 26.8, 25.2, 25.1, 24.5, 23.7, 22.3, 22.2, 14.3, 14.2, 11.3. IR (v_{max}, cm^{-1}) 3337 (m), 2951 (s), 2872 (m), 1733 (s), 1638 (s), 1509 (s), 1454 (s), 1426 (s), 1284 (s), 1254 (s), 1055 (s), 775 (m), 699 (m). HRMS (nanochip-ESI/LTQ-Orbitrap) m/z: $[M + H]^+$ Calcd for $C_{32}H_{39}N_2O_5^+$ 531.2853; Found 531.2838

Benzyl (2-((1-(2-hydroxy-5-methoxyphenyl)-2-phenylethyl)amino)-2-oxoethyl) carbamate (5d)

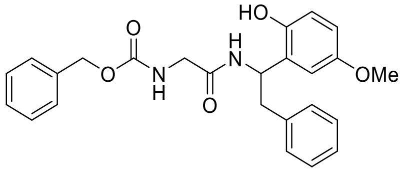

Following General Procedure 3 and starting with Cbz-Gly-Phe (**1c**) (107 mg, 0.300 mmol, 1.00 equiv) and 4-methoxyphenol (56 mg, 0.45 mmol, 1.50 equiv), **5d** was obtained after column chromatography DCM to DCM/ethyl acetate 10:1 as a colorless oil (81 mg, 0.19 mmol, 62%).

Rf (DCM/ethyl acetate 7:3): 0.31. ^1H NMR (400 MHz, Chloroform-*d,* unresolved mixture of rotamers) δ 7.98-7.49 (br s, 1H, O*H*), 7.34 (m, 5H, Ar*H*), 7.19 (dq, *J* = 14.2, 7.0 Hz, 4H, Ar*H*), 7.10 (d, *J* = 6.7 Hz, 2H, Ar*H*), 6.78 (d, *J* = 8.7 Hz, 1H, Ar*H*), 6.68 (dd, *J* = 8.7, 2.9 Hz, 1H, Ar*H*), 6.64 (s, 1H, N*H* Phe), 5.40 (s, 1H, N*H* Gly), 5.28 (q, *J* = 8.48 Hz, 1H, NHC*H*Ar), 5.09 (s, 2H, OCH_2Ph), 3.69 (d, *J* = 7.47 Hz, 5H, OCH_3 and NHCH_2), 3.20-3.05 (m, 2H, NHCHCH_2Ph). ^{13}C NMR (101 MHz, Chloroform-*d*, mixture of rotamers, signals not fully resolved) δ 169.8, 156.8, 153.5, 148.4, 137.7, 136.1, 129.2, 128.8, 128.6, 128.5, 128.3, 128.1, 126.8, 118.4, 114.0, 113.4, 67.5, 55.9, 51.1, 44. 6, 40.1. IR (v_{max}, cm^{-1}) 3402 (m), 3065 (m), 2935 (m), 2836 (w), 1707 (s), 1655 (s), 1524 (s), 1455 (m), 1350 (m), 1260 (s), 1211 (s), 1154 (s), 1032 (m), 910 (s), 735 (s). HRMS (ESI/QTOF) m/z: $[M + Na]^+$ Calcd for $C_{25}H_{26}N_2NaO_5^+$ 457.1734; Found 457.1738.

Benzyl (2S)-2-((1-(2-hydroxy-5-methoxyphenyl)-2-methylpropyl)carbamoyl)pyrrolidine-1-carboxylate (5e)

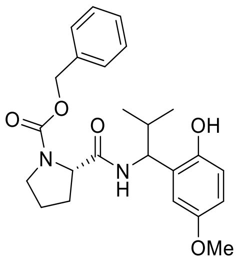

Following General Procedure 3 and starting with Cbz-Pro-Val (**1d**) (105 mg, 0.300 mmol, 1.00 equiv) and 4-methoxyphenol (56 mg, 0.45 mmol, 1.50 equiv), **5e** was obtained after column chromatography DCM to DCM/ethyl acetate 10:1 as a colorless oil (63 mg, 0.15 mmol, 49%).

Rf (DCM/ethyl acetate 7:3): 0.32. ^{1}H NMR (400 MHz, Chloroform-*d*, mixture of rotamers and diastereomers) δ 8.08-7.51 (m, 1H, O*H*), 7.33 (m, 4H, Ar*H*), 7.22 (m, 1H, Ar*H*), 7.09-6.90 (m, 1H, N*H*), 6.78 (d, *J* = 3.7 Hz, 1H, Ar*H*), 6.72-6.59 (m, 2H, Ar*H*), 5.31-4.88 (m, 2H, OCH_2Ph), 4.60 (t, *J* = 8.8 Hz, 1H, NHC*H*Ar), 4.34 (m, 1H, NC*H*C(O)), 3.74 (m, 3H, OCH_3), 3.63-3.32 (m, 2H, CH_2NCbz), 2.38-1.55 (m, 5H, NCH_2CH_2CH_2CH and NHCHC*H*), 1.09-0.69 (m, 6H, CH(CH_3)$_2$). ^{13}C NMR (101 MHz, Chloroform-*d*, mixture of diastereomers and rotamers, signals not fully resolved) δ 172.7, 172.6, 156.4, 155.6, 153.7, 153.5, 149.9, 148.9, 128.7, 128.4, 128.0, 116.2, 114.9, 113.7, 113.4, 67.7, 60.6, 55.9, 55.8, 47.6, 31.1, 24.7, 24.6, 20.4, 20.0. IR (ν_{max}, cm^{-1}) 3330 (m), 2958 (m), 2873 (m), 1685 (s), 1530 (s), 1508 (s), 1432 (s), 1356 (s), 1207 (s), 1119 (s), 1040 (m), 911 (s), 735 (s). HRMS (ESI/QTOF) m/z: [M + H]$^+$ Calcd for $C_{24}H_{31}N_2O_5^+$ 427.2227; Found 427.2229.

Benzyl ((2S)-1-((1-(2-hydroxy-5-methoxyphenyl)ethyl)amino)-1-oxopropan-2-yl) carbamate (5f)

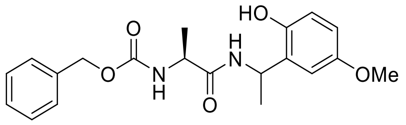

Following General Procedure 3 and starting with Cbz-Ala-Ala (**1b**) (88 mg, 0.30 mmol, 1.0 equiv) and 4-methoxyphenol (56 mg, 0.45 mmol, 1.50 equiv), **5f** was obtained after column chromatography DCM to DCM/ethyl acetate 10:1 as a colorless oil (71 mg, 0.19 mmol, 64%).

Rf (DCM/ethyl acetate 7:3): 0.34. ^{1}H NMR (400 MHz, Chloroform-*d*, mixture of rotamers and diastereomers) δ 8.23 (br s, 1H, OH), 7.41-7.27 (m, 5H, Ar*H*), 7.07-6.90 (br s, 1H, ArCHN*H*), 6.82 (dd, *J* = 8.7, 4.7 Hz, 1H, Ar*H*), 6.71 (ddd, *J* = 11.6, 6.2, 3.2 Hz, 2H, Ar*H*), 5.50-5.28 (m, 1H, CbzN*H*), 5.17 (dt, *J* = 15.0, 7.2 Hz, 1H, ArC*H*NH), 5.08 (m, 2H, OCH_2Ph), 4.17 (m, 1H, CbzNHC*H*), 3.73 (m, 3H, OCH_3), 1.49 (dd, *J* = 16.9, 6.2 Hz, 3H, ArCHCH_3), 1.37-1.27 (m, 3H, CbzNHCHCH_3). ^{13}C NMR (101 MHz, Chloroform-*d*, mixture of diastereomers and rotamers, signals not fully resolved) δ 173.0, 172.9, 156.2, 156.0, 153.4, 148.5, 135.9, 129.4, 128.6, 128.3, 128.1, 118.6, 118.5, 113.7, 112.3, 67.2, 55.8, 50.4, 50.3, 44.1, 19.6, 18.6, 18.0. IR (ν_{max}, cm^{-1}) 3327 (m), 3079 (m), 2936 (m), 2834 (m), 1705 (s), 1651 (s), 1508 (s), 1453 (s), 1258 (s), 1207 (s), 1030 (m), 910 (m), 734 (s), 699 (m). HRMS (ESI/QTOF) m/z: [M + Na]$^+$ Calcd for $C_{20}H_{24}N_2NaO_5^+$ 395.1577; Found 395.1582.

Benzyl (S)-2-((5-bromo-2-hydroxybenzyl)carbamoyl)pyrrolidine-1-carboxylate (5g)

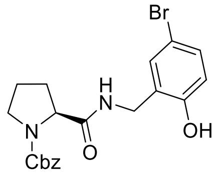

Following General Procedure 3 and starting with Cbz-Pro-Gly (**1e**) (92 mg, 0.30 mmol, 1.0 equiv) and 4-bromophenol (78 mg, 0.45 mmol, 1.5 equiv), **5g** was obtained after column chromatography DCM to DCM/ethyl acetate 10:1 as a white amorphous solid (61 mg, 0.14 mmol, 47%).

Rf (DCM/ethyl acetate 7:3): 0.5. ^{1}H NMR (400 MHz, Chloroform-*d*, 7:3 mixture of rotamers (major/minor)) δ 9.33 (br s, 1H, O*H* (major+minor)), 7.83 (br m, 0.7H, N*H* (major)), 7.44-7.27 (br m, 5H, Ar*H* (major+minor)), 7.19 (br m, 2H, Ar*H* (major+minor)), 7.04-6.91 (br m, 0.3H, N*H* (minor)), 6.80 (d, *J* = 8.6 Hz, 1H, Ar*H* (major+minor)), 5.09 (br m, 2H, OC*H*$_2$Ph (major+minor)), 4.44-4.05 (br m, 3H, NC*H*C(O), NHC*H*$_2$Ar (major+minor)), 3.46 (br m, 2H, C*H*$_2$NCbz (major+minor)), 2.13 (br m, 4H, NCH$_2$C*H*$_2$C*H*$_2$CH (major+minor)). ^{13}C NMR (101 MHz, Chloroform-*d*, mixture of rotamers, signals not fully resolved) δ 175.0, 174.4, 156.7, 155.2, 136.1, 133.4, 132.8, 128.8, 128.5, 128.1, 126.3, 120.0, 111.5, 67.8, 60.7, 60.2, 47.7, 47.4, 40.2, 28.1, 24.7, 23.9. IR (ν_{max}, cm^{-1}) 3291 (m), 3055 (m), 2933 (w), 1682 (s), 1542 (s), 1481 (s), 1419 (s), 1355 (s), 1275 (s), 1173 (s), 1092 (m), 909 (s), 732 (s). HRMS (ESI/QTOF) m/z: [M + Na]$^+$ Calcd for $C_{20}H_{21}{}^{(79)}BrN_2NaO_4^+$ 455.0577; Found 455.0579.

Benzyl (S)-2-((5-fluoro-2-hydroxybenzyl)carbamoyl)pyrrolidine-1-carboxylate (5h)

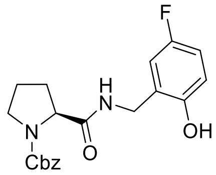

Following General Procedure 3 and starting with Cbz-Pro-Gly (**1e**) (92 mg, 0.30 mmol, 1.0 equiv) and 4-fluorophenol (50 mg, 0.45 mmol, 1.5 equiv), **5h** was obtained after column chromatography DCM to DCM/ethyl acetate 10:1 as a pale yellow oil (64 mg, 0.17 mmol, 57%).

Rf (DCM/ethyl acetate 7:3): 0.48. ^{1}H NMR (400 MHz, Chloroform-*d*, 1:1 mixture of rotamers (R^1/R^2)) δ 9.02 (br s, 1H, O*H* (R^1+R^2)), 7.82 (br m, 0.5H, N*H* (R^1)), 7.30 (br m, 5H, Ar*H* (R^1+R^2)), 7.18 (br m, 0.5H, N*H* (R^2)), 6.87 (qd, *J* = 8.8, 5.5 Hz, 2H, Ar*H* (R^1+R^2)), 6.72 (br m, 1H, Ar*H* (R^1+R^2)), 5.23-4.91 (br s, 2H, OC*H*$_2$Ph (R^1+R^2)), 4.44-4.08 (br m, 3H, NC*H*C(O), NHC*H*$_2$Ar (R^1+R^2)), 3.46 (br m, 2H, C*H*$_2$NCbz (R^1+R^2)), 2.47-1.79 (br m, 4H, NCH$_2$C*H*$_2$C*H*$_2$CH (R^1+R^2)). ^{13}C NMR (101 MHz, Chloroform-*d*, mixture of rotamers, signals not fully resolved) δ 174.3, 156.7, 156.3 (d, *J* = 236.7 Hz), 151.9 (d, *J* = 2.0 Hz), 136.2, 128.8, 128.5, 128.1, 125.2, 119.0 (d, *J* = 7.9 Hz), 116.8 (d, *J* = 23.1 Hz), 116.4 (d, *J* = 23.2 Hz), 67.8, 60.2, 59.5, 47.3, 47.0, 40.3, 28.0, 24.7, 23.9, 22.8. ^{19}F NMR (376 MHz, Chloroform-*d*) δ -124.92, -125.08. IR (ν_{max}, cm^{-1}) 3314 (m), 2925 (m), 2853 (m), 1684 (s), 1536 (m), 1508 (s), 1444 (s), 1356 (s), 1256 (s), 1188 (s), 1122 (s), 909 (s), 733 (s). HRMS (ESI/QTOF) m/z: [M + Na]$^+$ Calcd for $C_{20}H_{21}BrN_2NaO_4^+$ 455.0577; Found 455.0579.

Benzyl(S)-2-((5-((S)-2-(((benzyloxy)carbonyl)amino)-3-methoxy-3-oxopropyl)-2-hydroxy benzyl)carbamoyl)pyrrolidine-1-carboxylate (5i)

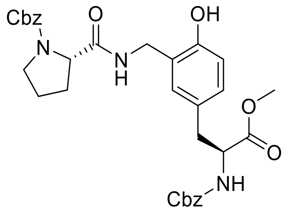

Following General Procedure 3 and starting with Cbz-Pro-Gly (**1e**) (92 mg, 0.30 mmol, 1.0 equiv) and Cbz-Tyr-OMe (148 mg, 0.450 mmol, 1.50 equiv), **5i** was obtained after column chromatography DCM to DCM/ethyl acetate 10:1 as a white amorphous solid (65 mg, 0.11 mmol, 37%).

Rf (DCM/ethyl acetate 7:3): 0.32. ^{1}H NMR (400 MHz, Chloroform-*d*, complex mixture of rotamers) δ 9.05 (br s, 1H, O*H*), 7.68 (br m, 0.5H, N*H* Gly), 7.43-7.27 (m, 10H, Ar*H*), 7.19 (br m, 0.5H, N*H* Gly), 6.91 (dd, *J* = 8.3, 2.2 Hz, 1H, Ar*H*), 6.81 (d, J = 8.2 Hz, 2H, Ar*H*), 5.10 (m, 5H, OC*H*$_2$Ph Pro+Tyr and N*H* Tyr), 4.59 (q, *J* = 5.9 Hz, 0.8H, NHC*H* Tyr), 4.46 (br s, 0.2H, NHC*H* Tyr), 4.33 (br m, 1H, NC*H*C(O) Pro), 4.25 (br m, 0.5H, NHC*H*$_2$Ar Gly), 4.14 (dt, *J* = 12.1, 6.1 Hz, 1.5H, NHC*H*$_2$Ar Gly), 3.71 (s, 3H, OC*H*$_3$ Tyr), 3.44 (br m, 2H, C*H*$_2$NCbz Pro), 2.99 (qd, *J* = 14.0, 5.9 Hz, 2H, NHCHC*H*$_2$ Tyr), 2.44-1.73 (br m, 4H, NCH$_2$C*H*$_2$C*H*$_2$CH Pro). ^{13}C NMR (101 MHz, Chloroform-*d*, mixture of rotamers, signals not fully resolved) δ 174.1, 172.2, 156.6, 155.8, 155.1, 136.4, 136.2, 131.8, 130.8, 128.7, 128.6, 128.4, 128.3, 128.2, 128.1, 127.0, 124.2, 118.2, 67.7, 67.1, 60.3, 55.1, 52.5, 47.3, 40.5, 37.4, 28.1, 24.7. IR (ν_{max}, cm^{-1}) 3317 (m), 3065 (m), 2949 (m), 1693 (s), 1533 (s), 1436 (s), 1355 (s), 1262 (s), 1213 (s), 1121 (m), 911 (s), 732 (s). HRMS (ESI/QTOF) m/z: [M + H]$^+$ Calcd for $C_{32}H_{36}N_3O_8^+$ 590.2497; Found 590.2490.

Benzyl (S)-2-((5-((S)-2-((S)-2-(((benzyloxy)carbonyl)amino)propanamido)-3-methoxy-3-oxopropyl)-2-hydroxybenzyl)carbamoyl)pyrrolidine-1-carboxylate (5j)

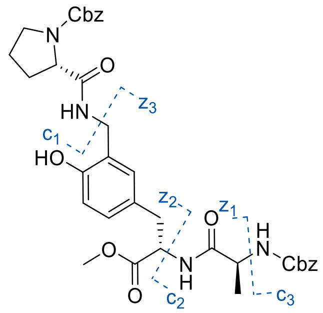

Following General Procedure 3 and starting with Cbz-Pro-Gly (**1e**) (92 mg, 0.30 mmol, 1.0 equiv) and Cbz-Ala-Tyr-OMe (180 mg, 0.450 mmol, 1.50 equiv), **5j** was obtained after column chromatography DCM to DCM/ethyl acetate 7:3 as a white amorphous solid (80 mg, 0.12 mmol, 40%).

Rf (DCM/ethyl acetate 7:3): 0.16. ^{1}H NMR (400 MHz, Acetonitrile-*d*$_3$, complex mixture of rotamers) δ 9.07 (s, 0.3H, O*H*), 8.54 (s, 0.4H, O*H*), 7.74 (s, 1H, N*H* Gly), 7.42-7.23 (m, 9H, Ar*H*), 7.16 (d, *J* = 5.8 Hz, 1H, Ar*H*), 6.99-6.83 (m, 3H, Ar*H* and N*H* Tyr), 6.71 (d, *J* = 8.1 Hz, 1H, Ar*H*), 6.18-6.06 (m, 0.7H, N*H* Ala), 5.89 (s, 0.3H, N*H* Ala), 5.19-4.87 (m, 4H, OC*H*$_2$Ph Pro+Ala), 4.57 (ddt, *J* = 10.3, 7.9, 4.0 Hz, 1H, NHC*H* Tyr), 4.31-4.01 (m, 4H, NC*H* Pro, NHC*H*$_2$ Gly and NHC*H* Ala), 3.66 (d, *J* = 3.3 Hz, 3H, OC*H*$_3$ Tyr), 3.46 (m, 2H, NC*H*$_2$ Pro), 3.07-2.96 (m, 1H, NCHC*H*$_2$ Tyr), 2.85 (m, 1H, NCHC*H*$_2$ Tyr), 2.15-2.01 (m, 1H, NCH$_2$C*H*$_2$C*H*$_2$CH Pro), 1.91-1.72 (m, 3H, NCH$_2$C*H*$_2$C*H*$_2$CH Pro), 1.20 (dd, *J* = 16.9, 7.1 Hz, 3H, C*H*$_3$ Ala). ^{13}C NMR (101 MHz, Acetonitrile-*d*$_3$, mixture of rotamers, signals not fully resolved) δ 176.2, 175.5,

173.4, 173.3, 172.8, 172.7, 172.6, 157.1, 156.9, 156.2, 155.6, 155.5, 155.0, 152.2, 138.1, 138.0, 137.9, 133.0, 132.4, 131.5, 131.1, 130.5, 130.4, 130.1, 129.5, 129.4, 128.9, 128.7, 128.6, 128.4, 125.7, 120.6, 117.4, 117.3, 67.7, 67.5, 67.3, 67.1, 61.9, 61.4, 54.6, 54.4, 52.8, 51.5, 48.2, 47.8, 40.2, 39.6, 37.0, 36.9, 32.1, 30.9, 25.1, 24.3, 18.5, 18.3. IR (v_{max}, cm^{-1}) 3317 (s), 2954 (m), 1661 (s), 1535 (s), 1448 (s), 1358 (s), 1256 (s), 1213 (s), 1120 (m), 1059 (m), 910 (s), 773 (m), 735 (s), 698 (s). HRMS (nanochip-ESI/LTQ-Orbitrap) m/z: $[M + H]^+$ Calcd for $C_{35}H_{41}N_4O_9^+$ 661.2868; Found 661.2883. MS-MS (nanochip-ESI/LTQ-Orbitrap): selected ion 661.2883. Measured c and z ions are reported in the table below

	**G**	**Y**	**A**
N-terminal	1	2	3
c	249.12	-	-
C-terminal	3	2	1
z	413.17	-	510.22

Benzyl (2-(2-(1H-indol-3-yl)pyrrolidin-1-yl)-2-oxoethyl)carbamate (6a)

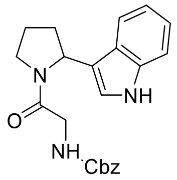

Following General Procedure 4 and starting with Cbz-Gly-Pro (**1a**) (92 mg, 0.30 mmol, 1.0 equiv) and 1H-indole (36 mg, 0.31 mmol, 1.02 equiv), **6a** was obtained after column chromatography DCM to DCM/ethyl acetate 10:1 as a pale yellow amorphous solid (75 mg, 0.20 mmol, 66%).

Rf (DCM/ethyl acetate 7:3): 0.25. ^1H NMR (400 MHz, Chloroform-*d*, 7:3 mixture of rotamers(major/minor)) δ 8.42 (s, 0.3H, N*H* indole (minor)), 8.35 (s, 0.7H, N*H* indole (major)), 7.58 (d, *J* = 7.9 Hz, 0.3H, Ar*H* (minor)), 7.50 (d, *J* = 7.9 Hz, 0.7H, Ar*H* (major)), 7.39-7.27 (m, 6H, Ar*H* (major+minor)), 7.24-7.17 (m, 1H, Ar*H* (major+minor)), 7.17-7.09 (m, 1H, Ar*H* (major+minor)), 6.88-6.80 (m, 1H, Ar*H* (major+minor)), 5.80 (s, 0.3H, N*H* Gly (minor)), 5.66 (s, 0.7H, N*H* Gly (major)), 5.59 (dd, *J* = 7.1, 2.7 Hz, 0.3H, C(O)NC*H* (minor)), 5.23-5.14 (m, 0.7H, C(O)NC*H* (major)), 5.11 (s, 0.6H, OCH_2Ph (minor)), 5.04 (s, 1.4H, OCH_2Ph (major)), 4.04 (dd, *J* = 15.8, 4.6 Hz, 1.3H, $C(O)CH_2NHCbz$ (major+minor)), 3.81 (dt, *J* = 12.0, 5.8 Hz, 0.7H, $C(O)NCH_2$ (major)), 3.69 (m, 1.7H, $C(O)CH_2NHCbz$ (major) and $C(O)NCH_2$ (major+minor)), 3.53 (q, *J* = 8.1 Hz, 0.3H, $C(O)NCH_2$ (minor)), 2.36-1.85 (m, 4H, $NCH_2CH_2CH_2CH$ (major+minor)).^{13}C NMR (101 MHz, Chloroform-*d*, mixture of rotamers, signals not fully resolved) δ 167.7, 166.7, 156.5, 156.3, 137.0, 136.6, 128.6, 128.2, 128.1, 125.3, 124.8, 122.7, 122.2, 121.4, 121.3, 120.0, 119.6, 119.0, 118.7, 116.8, 116.7, 111.8, 111.6, 67.0, 66.9, 55.0, 54.9, 46.9, 46.0, 45.9, 43.7, 43.4, 34.5, 32.1, 24.2, 22.2. IR (v_{max}, cm^{-1}) 3311 (m), 3046 (w), 2977 (m), 2876 (w), 1710 (s), 1638 (s), 1521 (m), 1455 (s), 1252 (s), 1165 (m), 1055 (m), 910 (m), 737 (s) HRMS (ESI/QTOF) m/z: $[M + Na]^+$ Calcd for $C_{22}H_{23}N_3NaO_3^+$ 400.1632; Found 400.1629.

Benzyl (2-(2-(5-chloro-1H-indol-3-yl)pyrrolidin-1-yl)-2-oxoethyl)carbamate (6b)

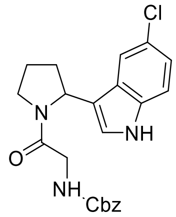

Following General Procedure 4 and starting with Cbz-Gly-Pro (**1a**) (92 mg, 0.30 mmol, 1.0 equiv) and 5-Chloro-1H-indole (46 mg, 0.31 mmol, 1.02 equiv), **6b** was obtained after column chromatography DCM to DCM/ethyl acetate 10:1 as a pale yellow oil (82 mg, 0.20 mmol, 66%).

Rf (DCM/ethyl acetate 7:3): 0.36. ^{1}H NMR (400 MHz, Chloroform-*d*, 6:4 mixture of rotamers(major/minor)) δ 8.72 (s, 0.4H, N*H* indole (minor)), 8.59 (s, 0.6H, N*H* indole (major)), 7.51 (d, *J* = 1.7 Hz, 0.4H, Ar*H* (minor)), 7.47-7.41 (m, 0.6H, Ar*H* (major)), 7.36-7.27 (m, 5H, Ar*H* (major+minor)), 7.22 (dd, *J* = 14.1, 8.7 Hz, 1H, Ar*H* (major+minor)), 7.11 (td, *J* = 8.7, 8.0, 1.7 Hz, 1H, Ar*H* (major+minor)), 6.82 (d, *J* = 1.9 Hz, 0.4H, Ar*H* (minor)), 6.73 (d, *J* = 2.4 Hz, 0.6H, Ar*H* (minor)), 5.75 (s, 0.4H, N*H* Gly (minor)), 5.68 (s, 0.6H, N*H* Gly (major)), 5.53-5.47 (m, 0.4H, C(O)NC*H* (minor)), 5.15-4.99 (m, 2.6H, C(O)NC*H* (major) and OC*H*$_2$Ph (major+minor)), 4.13-3.93 (m, 1.4H, C(O)C*H*$_2$NHCbz (major+minor)), 3.81-3.68 (m, 1.6H, C(O)NC*H*$_2$ (major+minor)), 3.64 (dd, *J* = 17.4, 4.5 Hz, 0.6H, C(O)C*H*$_2$NHCbz (major)), 3.53 (q, *J* = 7.8 Hz, 0.4H, C(O)NC*H*$_2$ (minor)), 2.35-2.16 (m, 1H, NCH$_2$C*H*$_2$C*H*$_2$CH (major+minor)), 2.06 (dtd, *J* = 17.1, 8.2, 3.7 Hz, 2H, NCH$_2$C*H*$_2$C*H*$_2$CH (major+minor)), 1.96-1.84 (m, 1H, NCH$_2$C*H*$_2$C*H*$_2$CH (major+minor)). ^{13}C NMR (101 MHz, Chloroform-*d*, mixture of rotamers, signals not fully resolved) δ 167.6, 166.9, 156.4, 156.3, 136.4, 135.3, 135.2, 128.5, 128.4, 128.1, 128.1, 128.0, 127.9, 126.1, 125.6, 125.5, 125.1, 122.9, 122.7, 122.3, 118.2, 118.0, 116.3, 116.2, 112.8, 112.6, 67.0, 66.9, 54.8, 54.6, 46.8, 46.0, 43.6, 43.3, 34.4, 32.1, 24.0, 22.1. IR (ν_{max}, cm^{-1}) 3308 (m), 2927 (m), 1710 (s), 1638 (s), 1520 (m), 1454 (s), 1254 (s), 1173 (m), 1055 (m), 909 (s), 733 (s).HRMS (ESI/QTOF) m/z: [M + Na]$^+$ Calcd for $C_{22}H_{22}ClN_3NaO_3^+$ 434.1242; Found 434.1253.

Benzyl (2-oxo-2-(2-(6-(trifluoromethyl)-1H-indol-3-yl)pyrrolidin-1-yl)ethyl)carbamate (6c)

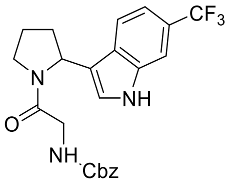

Following General Procedure 4 and starting with Cbz-Gly-Pro (**1a**) (92 mg, 0.30 mmol, 1.0 equiv) and 6-trifluoromethyl-1H-indole (57 mg, 0.31 mmol, 1.02 equiv), **6c** was obtained after column chromatography DCM to DCM/ethyl acetate 7:3 as a pale yellow oil (67 mg, 0.15 mmol, 50%).

Rf (DCM/ethyl acetate 7:3): 0.35. ^{1}H NMR (400 MHz, Acetonitrile-*d*$_3$, 1:1 mixture of rotamers, (R^1/R^2)) δ 9.69 (s, 0.5H, N*H* indole (R^1)), 9.52 (s, 0.5H, N*H* indole (R^2)), 7.79-7.73 (m, 1H, Ar*H* (R^1+R^2)), 7.73-7.66 (m, 1H, Ar*H* (R^1+R^2)), 7.39-7.26 (m, 5H, Ar*H* (R^1+R^2)), 7.26-7.21 (m, 1H, Ar*H* (R^1+R^2)), 7.19-7.09 (m, 1H, Ar*H* (R^1+R^2)), 5.84 (s, 0.5H, N*H* Gly (R^1)), 5.72 (s, 0.5H, N*H* Gly (R^2)), 5.47-5.39 (m, 0.5H, C(O)NC*H* (R^1)), 5.29 (d, *J* = 5.4 Hz, 0.5H, C(O)NC*H* (R^2)), 5.05 (s, 1H, OC*H*$_2$Ph (R^1)), 4.98 (s, 1H, OC*H*$_2$Ph (R^2)), 3.96 (d, *J* = 5.3 Hz, 1H, C(O)C*H*$_2$NHCbz (R^1+R^2)), 3.89 (dd, *J* = 17.2, 5.3 Hz, 0.5H, NC(O)C*H*$_2$NHCbz (R^1)), 3.72 (dt, *J* = 11.7, 6.3 Hz, 1H, C(O)NC*H*$_2$ (R^1+R^2)), 3.57 (m, 1H, C(O)NC*H*$_2$ (R^1+R^2)), 3.44 (dd, *J* = 17.0, 5.5 Hz, 0.5H,

C(O)C*H*2NHCbz (R2)), 2.39-2.13 (m, 2H, NCH2C*H*2C*H*2CH (R1+R2)), 2.03-1.97 (m, 1H, NCH2C*H*2C*H*2CH (R1+R2)), 1.91-1.82 (m, 1H, NCH2C*H*2C*H*2CH (R1+R2)).^{13}C NMR (101 MHz, Acetonitrile-d_3, mixture of rotamers, signals not fully resolved) δ 168.5, 167.8, 157.3 (d, J = 12.25 Hz), 138.2, 136.7, 136.4, 129.4, 129.3, 128.8, 128.7, 128.3, 127.8, 126.3, 125.9, 125.1, 124.3, 124.0, 123.8, 123.5, 120.28 (d, J = 13.93 Hz), 118.8, 116.4 (dd, J = 36.31, 3.45 Hz), 110.12 (dd, J = 28.22, 4.43 Hz), 67.1, 67.0, 55.0, 54.8, 47.4, 46.6, 44.1, 43.8, 35.5, 33.0, 30.3, 24.7, 22.8. IR (ν_{max}, cm^{-1}) 3365 (s), 3032 (m), 2930 (m), 1710 (s), 1642 (s), 1509 (s), 1454 (s), 1336 (s), 1257 (s), 1157 (s), 1111 (s), 1053 (s), 961 (m), 878 (m), 816 (s), 698 (s). HRMS (ESI/QTOF) m/z: [M + Na]$^+$ Calcd for $C_{23}H_{22}F_3N_3NaO_3^+$ 468.1505; Found 468.1513.

Benzyl (2-(2-(2-methyl-1H-indol-3-yl)pyrrolidin-1-yl)-2-oxoethyl)carbamate (6d)

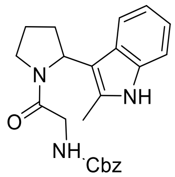

Following General Procedure 4 and starting with Cbz-Gly-Pro (**1a**) (92 mg, 0.30 mmol, 1.0 equiv) and 2-Methyl-1H-indole (40 mg, 0.31 mmol, 1.02 equiv), **6d** was obtained after column chromatography DCM to DCM/ethyl acetate 10:1 as a brown amorphous solid (58 mg, 0.15 mmol, 49%).

Rf (DCM/ethyl acetate 7:3): 0.36. ^{1}H NMR (400 MHz, Chloroform-d, 7:3 mixture of rotamers (major/minor)) δ 8.25 (s, 0.3H, N*H* indole (minor)), 8.18 (s, 0.7H, N*H* indole (major)), 7.34-7.22 (m, 7H, Ar*H* (major+minor)), 7.16-7.08 (m, 1H, Ar*H* (major+minor)), 7.04 (dd, J = 9.3, 6.6 Hz, 1H, Ar*H* (major+minor)), 5.65 (s, 1H, N*H* Gly (major+minor)), 5.32 (t, J = 6.9 Hz, 0.3H, C(O)NC*H* (minor)), 5.10-4.98 (m, 2.7H, C(O)NC*H* (major) and OC*H*2Ph (major+minor)), 4.10 (dd, J = 17.0, 4.5 Hz, 0.3H, C(O)C*H*2NHCbz (minor)), 4.00-3.90 (m, 1.7H, C(O)C*H*2NHCbz (major+minor) and C(O)NC*H*2 (major)), 3.79 (dt, J = 12.8, 7.2 Hz, 1H, C(O)NC*H*2 (major+minor)), 3.69 (td, J = 10.1, 8.0, 5.6 Hz, 0.3H, C(O)NC*H*2 (minor)), 3.30 (dd, J = 17.3, 3.2 Hz, 0.7H, C(O)C*H*2NHCbz (major)), 2.40-2.28 (m, 4H, C*H*3 and NCH2C*H*2C*H*2CH (major+minor)), 2.22-1.84 (m, 3H, NCH2C*H*2C*H*2CH (major+minor)).^{13}C NMR (101 MHz, Chloroform-d, mixture of rotamers, signals not fully resolved) δ 167.8, 166.4, 156.3, 156.2, 136.6, 135.5, 131.9, 131.3, 128.6, 128.1, 128.0, 126.2, 125.7, 121.6, 120.9, 119.9, 119.4, 118.1, 117.7, 111.6, 111.1, 110.9, 66.9, 66.8, 55.1, 54.8, 47.8, 47.1, 43.7, 43.3, 35.6, 32.7, 25.4, 23.7, 12.0, 11.8. IR (ν_{max}, cm^{-1}) 3393 (m), 3299 (m), 3060 (m), 2965 (m), 2875 (m), 1710 (s), 1637 (s), 1510 (m), 1459 (s), 1237 (m), 910 (m), 737 (s). HRMS (ESI/QTOF) m/z: [M + H]$^+$ Calcd for $C_{23}H_{26}N_3O_3^+$ 392.1969; Found 392.1977

Benzyl (2-(2-(3-methyl-1H-indol-1-yl)pyrrolidin-1-yl)-2-oxoethyl)carbamate (6e)

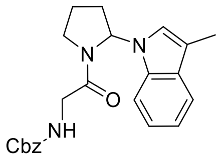

Following General Procedure 4 and starting with Cbz-Gly-Pro (**1a**) (92 mg, 0.30 mmol, 1.0 equiv) and 3-Methyl-1H-indole (40 mg, 0.31 mmol, 1.02 equiv), **6e** was obtained after column chromatography DCM to DCM/ethyl acetate 10:1 as a white amorphous solid (50 mg, 0.13 mmol, 43%).

Rf (DCM/ethyl acetate 7:3): 0.58. ^{1}H NMR (400 MHz, Chloroform-d, 6:4 mixture of rotamers (major/minor)) δ 7.55 (dd, J = 17.6, 7.8 Hz, 1H, Ar*H* (major+minor)), 7.43 (d, J = 8.2 Hz, 0.4H,

Ar*H* (minor)), 7.31 (*t*, J = 7.3 Hz, 6H, Ar*H* (major+minor)), 7.24-7.08 (m, 1.6H, Ar*H* (major+minor)), 6.76 (d, *J* = 11.0 Hz, 1H, Ar*H* (major+minor)), 6.52-6.45 (m, 0.4H, C(O)NC*H* (minor)), 6.21 (d, *J* = 5.2 Hz, 0.6H, C(O)NC*H* (major)), 5.65 (br s, 0.4H, N*H* (minor)), 5.43 (br s, 0.6H, N*H* (major)), 5.18-4.93 (m, 2H, OCH_2Ph (major+minor)), 4.13 (ddt, *J* = 13.9, 10.6, 5.0 Hz, 1H, C(O)CH_2NHCbz (major+minor)), 3.96 (ddd, *J* = 16.3, 10.9, 4.2 Hz, 1H, C(O)CH_2NHCbz (minor) and C(O)NCH_2 (major)), 3.84 (d, *J* = 10.2 Hz, 0.4H, C(O)NCH_2 (minor)), 3.73 (dt, *J* = 11.6, 8.1 Hz, 0.6H, C(O)NCH_2 (major)), 3.60 (dt, *J* = 18.4, 9.4 Hz, 0.4H, C(O)NCH_2 (minor)), 3.20 (dd, *J* = 17.4, 3.3 Hz, 0.6H,NC(O)CH_2NHCbz (major)), 2.47-2.23 (m, 4H, CH_3 and $NCH_2CH_2CH_2CH$ (major+minor)), 2.23-1.96 (m, 3H, $NCH_2CH_2CH_2CH$ (major+minor)).^{13}C NMR (101 MHz, Chloroform-*d*, mixture of rotamers, signals not fully resolved) δ 168.8, 167.8, 156.4, 156.3, 136.5, 134.9, 129.7, 129.2, 128.7, 128.6, 128.2, 128.1, 128.0, 122.6, 122.0, 121.5, 121.1, 119.9, 119.7, 119.3, 119.2, 112.8, 111.8, 109.8, 109.2, 68.7, 68.1, 67.0, 47.2, 46.1, 43.8, 43.1, 34.8, 32.3, 23.9, 21.7, 9.9. IR (ν_{max}, cm^{-1}) 3415 (m), 3053 (m), 2935 (w), 2145 (w), 1714 (s), 1662 (s), 1510 (m), 1432 (m), 1350 (m), 1193 (m), 985 (m), 910 (s), 736 (s). HRMS (ESI/QTOF) m/z: [M + Na]$^+$ Calcd for $C_{23}H_{25}N_3NaO_3^+$ 414.1788; Found 414.1797.

Benzyl (2-(2-(3-(2-acetamidoethyl)-5-methoxy-1H-indol-1-yl)pyrrolidin-1-yl)-2-oxoethyl) carbamate (6f)

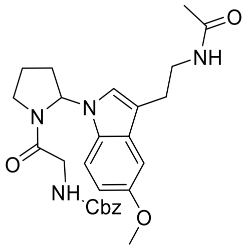

Following General Procedure 4 and starting with Cbz-Gly-Pro (**1a**) (92 mg, 0.30 mmol, 1.0 equiv) and melatonin (71 mg, 0.31 mmol, 1.02 equiv), **6f** was obtained after column chromatography DCM to ethyl acetate as a colorless oil (95 mg, 0.19 mmol, 64% yield).

Rf (DCM/ethyl acetate 7:3): 0.13. ^{1}H NMR (400 MHz, Chloroform-*d*, 7:3 mixture of rotamers (major/minor)) δ 7.38-7.27 (m, 5.3H, Ar*H* (major+minor)), 7.19 (d, *J* = 8.9 Hz, 0.7H, Ar*H* (major)), 7.03 (d, *J* = 2.3 Hz, 0.7H, Ar*H* (major)), 6.98 (d, *J* = 2.3 Hz, 0.3H, Ar*H* (minor)), 6.90 (dt, *J* = 8.8, 2.9 Hz, 1H, Ar*H* (major+minor)), 6.86 (s, 0.3H, Ar*H* (minor)), 6.82 (s, 0.7H, Ar*H* (major)), 6.40 (d, *J* = 5.3 Hz, 0.3H, C(O)NC*H* (minor)), 6.28 (s, 0.7H, AcN*H* (major)), 6.08 (dd, *J* = 6.0, 2.8 Hz, 0.7H, C(O)NC*H* (major)), 5.65 (s, 0.6H, AcN*H* and CbzN*H* (minor)), 5.48 (s, 0.7H, CbzN*H* (major)), 5.09 (s, 0.6H, OCH_2Ph (minor)), 5.00 (d, *J* = 2.8 Hz, 1.4H, OCH_2Ph (major)), 4.15-3.89 (m, 1.7H, C(O)CH_2NHCbz (major+minor) and C(O)NCH_2 (major)), 3.89-3.70 (m, 4.3H, OCH_3 (major+minor), C(O)CH_2NHCbz (minor) and C(O)NCH_2 (major+minor)), 3.54 (m, 2.3H, C(O)NCH_2 (minor) and CH_2NHAc (major+minor)), 3.17 (dd, *J* = 17.4, 5.6 Hz, 0.7H, C(O)CH_2NHCbz (major)), 2.90 (dt, *J* = 13.9, 7.0 Hz, 2H, CH_2CH$_2$NHAc (major+minor)), 2.47-1.97 (m, 4H, $NCH_2CH_2CH_2CH$ (major+minor)), 1.88 (d, *J* = 14.7 Hz, 3H, NHC(O)CH_3 (major+minor)).^{13}C NMR (101 MHz, Chloroform-*d*, mixture of rotamers, signals not fully resolved) δ 170.7, 170.3, 168.6, 168.0, 156.5, 154.6, 154.3, 136.4, 136.2, 130.9, 129.8, 129.6, 128.8, 128.6, 128.3, 128.2, 128.1, 122.9, 122.6, 114.1, 113.0, 112.3, 112.2, 110.8, 110.2, 101.4, 101.1, 69.2, 68.5, 67.1, 56.0, 47.2, 46.1, 43.8, 43.0, 40.2, 39.5, 34.7, 32.2, 25.5, 25.0, 23.9, 23.5, 23.3, 21.8. IR (ν_{max}, cm^{-1}) 3330 (m), 3033 (m), 2946 (m), 2833 (m), 1715 (s), 1654 (s), 1542 (s), 1484 (s), 1453 (s), 1220 (s), 1050 (m), 909 (s), 733 (s). HRMS (ESI/QTOF) m/z: [M + Na]$^+$ Calcd for $C_{27}H_{32}N_4NaO_5^+$ 515.2265; Found 515.2276

Benzyl (2-((1-(1H-indol-3-yl)-2-phenylethyl)amino)-2-oxoethyl)carbamate (6g)

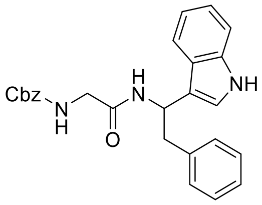

Following General Procedure 4 and starting with Cbz-Gly-Phe (**1c**) (107 mg, 0.30 mmol, 1.0 equiv) and 1H-indole (36 mg, 0.31 mmol, 1.02 equiv), **6g** was obtained after column chromatography DCM to DCM/ethyl acetate 10:1 as a red amorphous solid (74 mg, 0.17 mmol, 58%).

Rf (DCM/ethyl acetate 7:3): 0.32. ^{1}H NMR (400 MHz, Chloroform-*d*) δ 8.12-7.93 (m, 1H, N*H* indole), 7.64 (d, *J* = 7.9 Hz, 1H, Ar*H*), 7.34 (d, *J* = 7.9 Hz, 6H, Ar*H*), 7.24-7.06 (m, 7H, Ar*H*), 6.93-6.88 (m, 1H, Ar*H*), 6.45-6.19 (m, 1H, N*H* Phe), 5.59 (q, *J* = 7.2 Hz, 1H, NHC*H* Phe), 5.45-5.28 (m, 1H, N*H* Gly), 5.08 (s, 2H, OCH_2Ph), 3.74 (t, *J* = 6.9 Hz, 2H, NHCH_2 Gly), 3.26 (tt, *J* = 13.6, 6.7 Hz, 2H, NHCHCH_2 Phe). ^{13}C NMR (101 MHz, Chloroform-*d*) δ 168.1, 156.7, 138.0, 136.6, 136.2, 129.4, 128.7, 128.4, 128.3, 128.2, 126.6, 125.9, 122.6, 122.1, 120.1, 119.3, 116.0, 111.6, 67.3, 48.1, 44.8, 41.1. IR (v_{max}, cm^{-1}) 3344 (m), 3032 (m), 2946 (m), 1712 (s), 1661 (s), 1521 (s), 1455 (m), 1339 (m), 1259 (m), 1155 (m), 1075 (m), 910 (s), 740 (s), 699 (s). HRMS (ESI/QTOF) m/z: [M + Na]$^+$ Calcd for $C_{26}H_{25}N_3NaO_3^+$ 450.1788; Found 450.1792

Benzyl (2S)-2-((1-(1H-indol-3-yl)-2-methylpropyl)carbamoyl)pyrrolidine-1-carboxylate (6h)

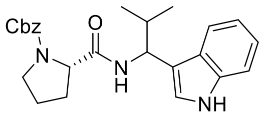

Following General Procedure 4 and starting with Cbz-Pro-Val (**1d**) (105 mg, 0.30 mmol, 1.0 equiv) and 1H-indole (**x**) (36 mg, 0.31 mmol, 1.02 equiv), **6h** was obtained after column chromatography DCM to DCM/ethyl acetate 10:1 as a red amorphous solid (81 mg, 0.19 mmol, 64%).

Rf (DCM/ethyl acetate 7:3): 0.29. ^{1}H NMR (400 MHz, Chloroform-*d*, complex mixture of diastereomers and rotamers) δ 8.22-7.85 (m, 1H, N*H* indole), 7.62-7.41 (m, 1H, Ar*H*), 7.26 (m, 5H, Ar*H*), 7.14-6.74 (m, 4H, Ar*H*), 6.24 (m, 0.4H, N*H* Val not fully resolved), 5.21-4.86 (m, 3H, OCH_2Ph and NHC*H* Val), 4.47-4.19 (m, 1H, CbzNC*H* Pro), 3.56-3.18 (m, 2H, CbzNCH_2 Pro), 2.42-1.94 (m, 3H, NHCHC*H* Val and NCH_2CH_2CH_2CH Pro), 1.89-1.63 (m, 2H, NCH_2CH_2CH_2CH Pro), 0.97-0.69 (m, 6H, CH_3 Val). ^{13}C NMR (101 MHz, Chloroform-*d*, mixture of diastereomers and rotamers, signals not fully resolved) δ 171.2, 170.7, 156.4, 155.7, 136.6, 136.5, 128.7, 128.3, 126.3, 122.1, 121.9, 119.5, 116.7, 116.2, 111.4, 67.4, 61.4, 60.8, 52.5, 47.8, 47.1, 32.8, 31.2, 28.1, 24.7, 23.6, 20.2, 19.0, 18.7. IR (v_{max}, cm^{-1}) 3314 (m), 2962 (m), 2910 (m), 1690 (s), 1660 (s), 1520 (m), 1418 (s), 1356 (s), 1210 (m), 1118 (m), 909 (s), 735 (s), 698 (m). HRMS (ESI/QTOF) m/z: [M + Na]$^+$ Calcd for $C_{25}H_{29}N_3NaO_3^+$ 442.2101; Found 442.2107.

Methyl Nα-((benzyloxy)carbonyl)-1-(1-(((benzyloxy)carbonyl)glycyl)pyrrolidin-2-yl)-L-tryptophanate (6i)

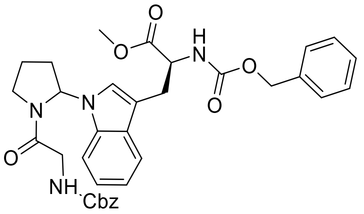

Following General Procedure 4 and starting with Cbz-Gly-Pro (**1a**) (92 mg, 0.30 mmol, 1.0 equiv) and Z-Trp-OMe (108 mg, 0.306 mmol, 1.02 equiv), **6i** was obtained after column chromatography DCM to DCM/ethyl acetate 10:1 as a white amorphous solid (105 mg, 0.171 mmol, 57% yield).

Rf (DCM/ethyl acetate 7:3): 0.58. ^{1}H NMR (400 MHz, Chloroform-*d*, complex mixture of diastereomers and rotamers) δ 7.57-7.39 (m, 2H, Ar*H*), 7.38-7.27 (m, 10H, Ar*H*), 7.21-7.06 (m, 2H, Ar*H*), 6.83-6.66 (m, 1H, Ar*H*), 6.45 (d, *J* = 6.2 Hz, 0.4H, C(O)NC*H* Pro), 6.17 (t, *J* = 5.5 Hz, 0.6H, C(O)NC*H* Pro), 5.66-5.22 (m, 2H, N*H* Gly+Trp), 5.05 (m, 4H, OCH_2Ph Gly+Trp), 4.76-4.64 (m, 1H, NHC*H* Trp), 4.18-3.86 (m, 2H, C(O)NCH_2 Pro and NHCH_2 Gly), 3.79-3.50 (m, 4H, OCH_3 Trp and C(O)NCH_2 Pro), 3.41-2.96 (m, 3H, NHCH_2 Gly and NHCHCH_2 Trp), 2.46-1.88 (m, 4H, NCH_2CH_2CH_2CH Pro).^{13}C NMR (101 MHz, Chloroform-*d*, mixture of diastereomers and rotamers, signals not fully resolved) δ 172.5, 172.4, 172.2, 168.9, 168.8, 167.8, 156.5, 156.4, 155.9, 155.8, 136.5, 136.2, 135.5, 134.7, 129.2, 128.6, 128.5, 128.2, 128.1, 122.9, 122.8, 122.4, 122.2, 120.6, 119.9, 119.8, 119.6, 119.1, 118.8, 111.3, 110.9, 110.1, 109.8, 109.4, 68.7, 68.3, 67.0, 67.0, 55.1, 54.8, 54.6, 52.6, 52.5, 47.2, 46.1, 43.8, 43.0, 34.8, 32.2, 28.3, 28.1, 23.8, 21.5. IR (ν_{max}, cm^{-1}) 3411 (m), 3280 (m), 3061 (m), 2937 (m), 1716 (s), 1667 (s), 1517 (m), 1437 (m), 1344 (m), 1211 (s), 1060 (m), 911 (m), 738 (s). HRMS (ESI/QTOF) m/z: [M + Na]$^+$ Calcd for $C_{34}H_{36}N_4NaO_7^+$ 635.2476; Found 635.2491.

Methyl Nα-(((benzyloxy)carbonyl)-L-valyl)-1-(1-(((benzyloxy)carbonyl)glycyl)pyrrolidin-2-yl)-L-tryptophanate (6j)

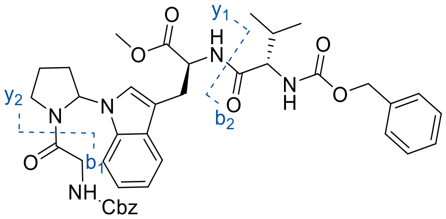

Following General Procedure 4 and starting with Cbz-Gly-Pro (**1a**) (92 mg, 0.30 mmol, 1.0 equiv) and Z-Val-Trp-OMe (138 mg, 0.306 mmol, 1.02 equiv), **6j** was obtained after column chromatography DCM to DCM/ethyl acetate 10:1 as a white amorphous solid (106 mg, 0.149 mmol, 50% yield).

Rf (DCM/ethyl acetate 7:3): 0.48. ^{1}H NMR (400 MHz, Acetonitrile-d_3, complex mixture of diastereomers and rotamers) δ 7.57-7.42 (m, 2H, Ar*H*), 7.38-7.25 (m, 10H, Ar*H*), 7.18 (dt, *J* = 8.1, 4.9 Hz, 1H, Ar*H*), 7.09 (dt, *J* = 15.1, 7.5 Hz, 2H, Ar*H*), 7.04-6.98 (m, 0.5H, N*H* Trp), 6.82 (dd, *J* = 16.7, 7.4 Hz, 0.5H, N*H* Trp), 6.40-6.25 (m, 1H, C(O)NC*H* Pro), 5.91 (d, *J* = 9.0 Hz, 2H, N*H* Gly+Val), 5.12-4.95 (m, 4H, OCH_2Ph Gly+Val), 4.77-4.64 (m, 1H, NHC*H* Trp), 4.10-3.79 (m, 4H, NHCH_2 Gly, NHCH_2 Val and 1H C(O)NCH_2 Pro), 3.68-3.50 (m, 4H, OCH_3 Trp and 1H C(O)NCH_2 Pro), 3.35-2.77 (m, 2H, NHCHCH_2 Trp), 2.48-2.19 (m, 2H, NCH_2CH_2CH_2CH Pro), 2.13-2.01 (m, 3H, NCH_2CH_2CH_2CH Pro and NHCHC*H* Val), 0.94-0.72 (m, 6H, CH_3 Val).^{13}C NMR (101 MHz, Acetonitrile-d_3, mixture of diastereomers and rotamers, signals not fully resolved) δ 173.1, 173.0, 172.9, 172.8, 172.3, 172.1, 169.6, 169.4, 169.2, 157.6, 157.4, 157.3, 157.0, 138.2, 138.0, 136.2, 135.8, 129.4, 129.3, 129.0, 128.8, 128.7,

128.6, 128.5, 125.1, 124.9, 124.7, 124.4, 123.2, 123.1, 122.8, 122.6, 122.5, 120.8, 120.7, 120.2, 120.1, 120.0, 119.7, 119.6, 119.1, 112.1, 111.8, 111.6, 111.1, 111.0, 110.7, 110.5, 110.0, 109.8, 106.9, 69.5, 69.3, 69.1, 67.2, 67.1, 66.9, 61.4, 61.1, 60.9, 60.3, 56.1, 54.6, 54.2, 53.9, 53.8, 53.5, 53.4, 52.7, 47.7, 47.6, 46.9, 46.8, 44.2, 43.5, 43.4, 35.4, 35.2, 33.4, 33.1, 33.0, 32.6, 32.2, 31.8, 31.7, 28.1, 28.0, 27.9, 27.8, 26.6, 25.7, 25.2, 24.3, 22.3, 22.2, 19.6, 19.5, 19.4, 19.2, 18.3, 18.1, 18.0, 17.8. IR (v_{max}, cm^{-1}) 3307 (m), 2963 (m), 2892 (m), 1714 (s), 1660 (s), 1524 (s), 1438 (s), 1324 (m), 1236 (s), 1055 (m), 910 (m), 735 (s). HRMS (ESI/QTOF) m/z: $[M + Na]^+$ Calcd for $C_{39}H_{45}N_5NaO_8^+$ 734.3160; Found 734.3167. MS-MS (nanochip-ESI/LTQ-Orbitrap): selected ion 734.3167. Measured b and y ions are reported in the table below

	**P**	**W**
N-terminal	1	2
b	-	-
C-terminal	2	1
y	521.28	479.23

6. Scope on Tetrapeptides

General procedure 5 for the decarboxylative arylation of Tetrapeptides

A 5 mL test tube was charged under Ar with tetrapeptide (5.0 µmol, 1.0 equiv), BIOAc (4.6 mg, 15 µmol, 3.0 equiv), $Ru(bpy)_3Cl_2$ (1.1 mg, 1.5 µmol, 0.30 equiv) and 1.0 mL of degazed MeCN. The solution was degazed by argon sparging for 5 min. 0.20 mL of the solution were placed into a sealed vial under argon. The reaction mixture was irradiated using blue light LEDs at rt overnight. Then a 41 mM solution of 1H-indole in MeCN (50 µL, 2.0 µmol, 2.0 equiv) and 2,2,2-trifluoroacetic acid (1.2 µL, 15 µmol, 15 equiv) were added. The reaction was let stirring for 1 h. At the end of the reaction, the crude was diluted with 3x the volume of MeCN and injected in RP-HPLC. The yields were determined as the ratio of Aprod/Atotal where Aprod = area in mAU of the product peak (blue arrow in HPLC traces) and Atotal = area in mAU of all peptides products (product, starting material, and side-products if present (red arrow in HPLC traces)). Reported result is an average of 3 independent trials.

(2S)-N-(2-((1-(1H-indol-3-yl)ethyl)amino)-2-oxoethyl)-2-((S)-2-acetamidopropanamido)-3-phenylpropanamide (8a)

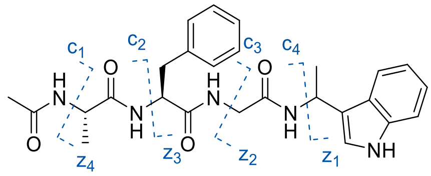

Following general procedure 5, Ac-Ala-Phe-Gly-Ala-OH (**7a**) afforded **8a** in more than 95% HPLC ratio (66% yield with calibration curve) as a mixture of diastereoisomers (retention time 11.299 min).

Reaction performed on 20 µmol scale afforded **8a** after purification by preparative RP-HPLC (gradient water-95% acetonitrile in 20 min) as a brown fluffy solid (1.7 mg, 3.6 µmol, 18%).

^{1}H NMR (400 MHz, Acetonitrile-*d*$_3$, complex mixture of diastereomers and rotamers) δ 9.21-9.01 (m, 1H, N*H* indole), 7.66-7.56 (m, 1H, Ar*H*), 7.42-7.35 (m, 1H, Ar*H*), 7.34-7.16 (m, 7H, Ar*H*+N*H* Gly), 7.13 (dddd, *J* = 8.2, 7.1, 4.7, 1.1 Hz, 1H, Ar*H*), 7.08-6.91 (m, 3H, Ar*H*+N*H* Ala+Phe), 6.80-6.64 (m, 1H, N*H* Ala), 5.41-5.29 (m, 1H, NHC*H*indole), 4.33 (dddt, *J* = 9.2, 7.1, 5.0, 2.2 Hz, 1H, NHC*H* Phe), 4.10-4.02 (m, 0.5H, AcNHC*H* Ala), 4.01-3.93 (m, 0.5H, AcNHC*H* Ala), 3.77-3.68 (m, 2H, NHCH_2 Gly), 3.17 (dt, *J* = 14.1, 5.0 Hz, 1H, NHCHCH_2Ph Phe), 2.94 (dt, *J* = 10.3, 5.3 Hz, 1H, NHCHCH_2Ph Phe), 1.84 (s, 1.5H C(O)CH_3 Ac-Ala), 1.77 (s, 1.5H, C(O)CH_3 Ac-Ala), 1.55 (dd, *J* = 6.9, 4.8 Hz, 3H, CH_3 Ala-indole), 1.15 (dd, *J* = 7.2, 1.5 Hz, 1.5H, CH_3 Ac-Ala), 1.08 (d, *J* = 7.2 Hz, 1.5H, CH_3 Ac-Ala). ^{13}C NMR (101 MHz, Acetonitrile-*d*$_3$, mixture of diastereomers and rotamers, signals not fully resolved) δ 174.3, 174.2, 172.2, 172.2, 168.8, 168.7, 138.6, 138.5, 137.7, 137.6, 130.2, 129.3, 127.6, 122.7, 122.6, 122.6, 120.1, 120.1, 120.0, 120.0, 112.3, 56.2, 56.1, 50.9, 50.9, 43.7, 43.6, 42.6, 42.4, 37.2, 37.1, 22.9, 22.7, 21.6, 21.5, 17.1, 17.0.

HRMS (nanochip-ESI/LTQ-Orbitrap) m/z: $[M + H]^+$ Calcd for $C_{26}H_{32}N_5O_4^+$ 478.2449; Found 478.2467. MS-MS (nanochip-ESI/LTQ-Orbitrap): selected ion 478.2467. Measured c and z ions are reported in the table below

	A	F	G	A*
N-terminal	1	2	3	4
c	-	-	-	335.17
C-terminal	4	3	2	1
z	-	-	-	144.08

Calibration with arylated **8a** was achieved through the preparation of several samples of different concentrations and their analysis on RP HPLC. The following linear regression was obtained y = 0,0001x - 0,0147 and R^2 = 0.998, where Y is the concentration in µmol/mL of **8a** and X the absorbance area of the peak at 214 nm.

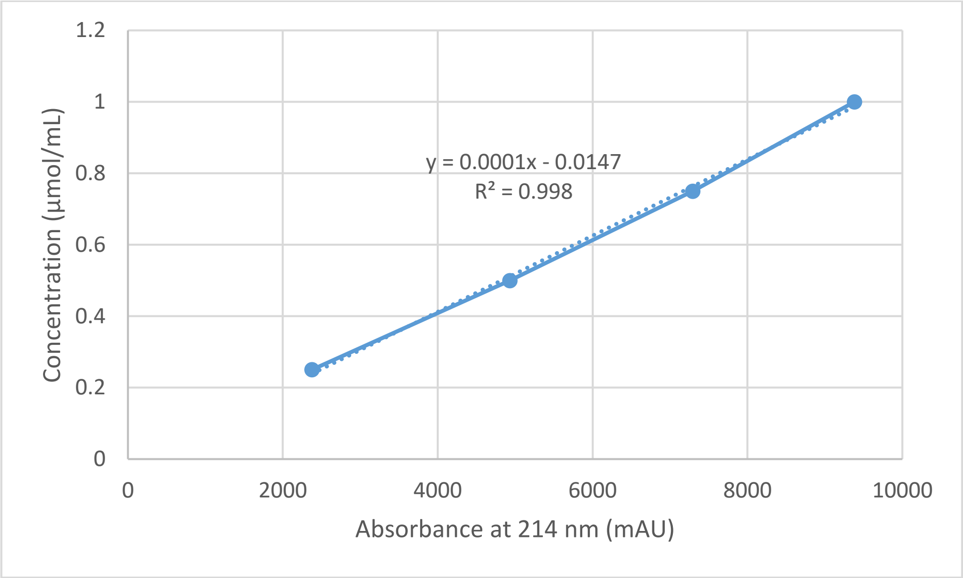

Ac-Ala-Phe-Gly-Ala-OH (**7a**)

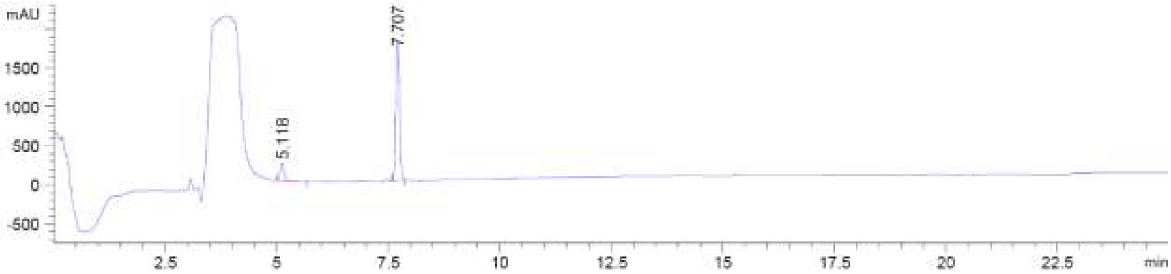

HRMS of **7a** (ESI/QTOF) m/z: $[M + Na]^+$ Calcd for $C_{19}H_{26}N_4NaO_6^+$ 429.1745; Found 429.1736.

HPLC-UV chromatogram at 214 nm

Control experiment without peptide: Iodobenzoic acid (**9**) (12.713 min) and degradation of $Ru(bpy)_3Cl_2 \cdot 6H_2O$ (5.945, 14.321, 16.218 min) are observed.

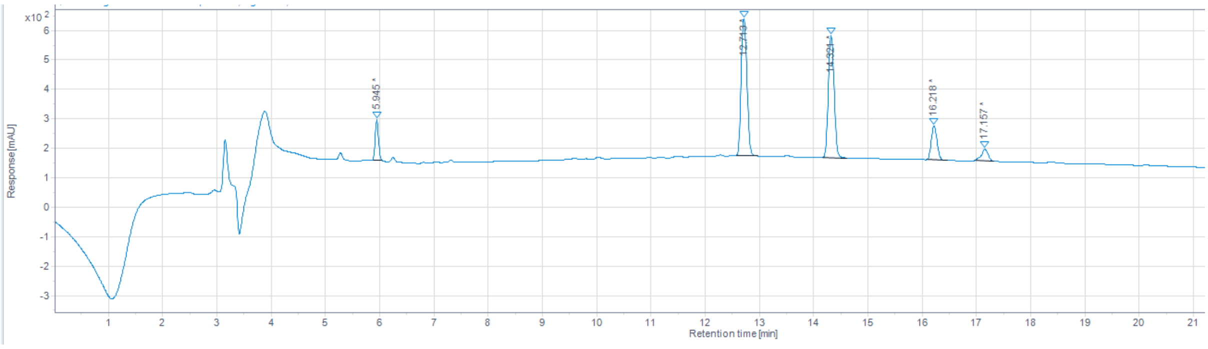

Crude reaction mixture:

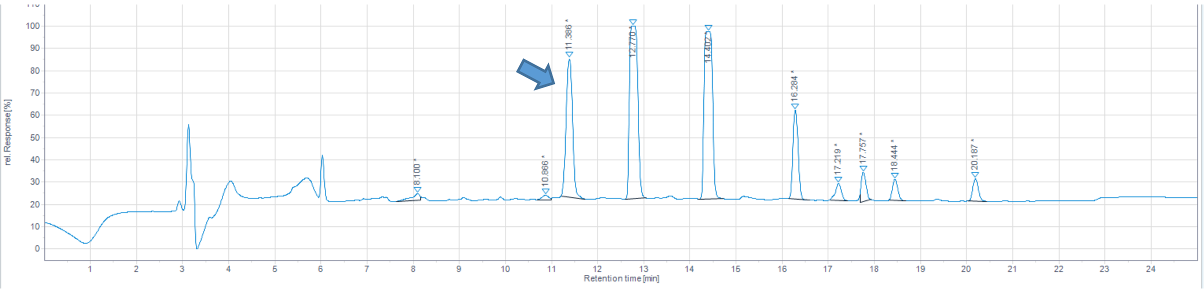

(2S)-2-((S)-2-acetamidopropanamido)-N-(2-((2-hydroxy-1-(1H-indol-3-yl)ethyl)amino)-2-oxoethyl)-3-phenylpropanamide (8b)

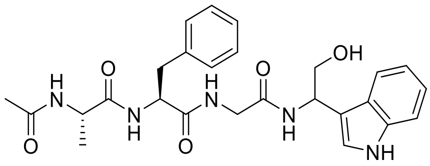

Following general procedure 5, Ac-Ala-Phe-Gly-Ser-OH (**7b**) afforded **8b** in more than 95% HPLC ratio as a mixture of diastereomers (retention time 10.186 min)

HRMS (ESI/QTOF) m/z: $[M + Na]^+$ Calcd for $C_{26}H_{31}N_5NaO_5^+$ 516,2217; Found 516,2220

Ac-Ala-Phe-Gly-Ser-OH (**7b**)

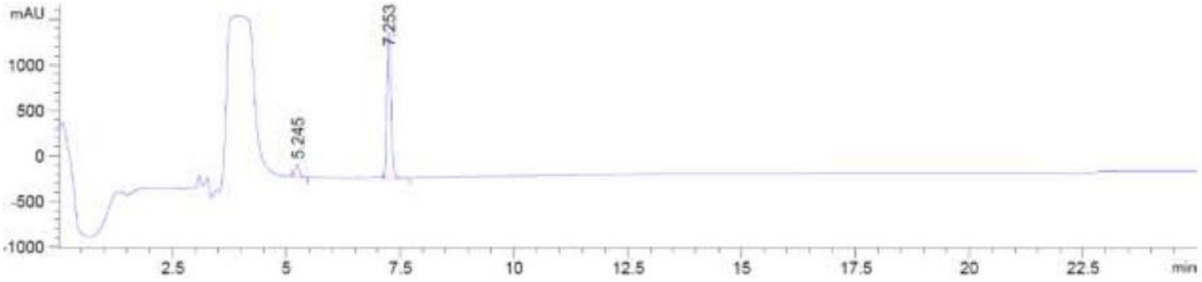

HRMS of **7b** (ESI/QTOF) m/z: $[M + Na]^+$ Calcd for $C_{19}H_{26}N_4NaO_7^+$ 445.1694; Found 445.1688.

HPLC-UV chromatogram at 214 nm of the crude reaction mixture

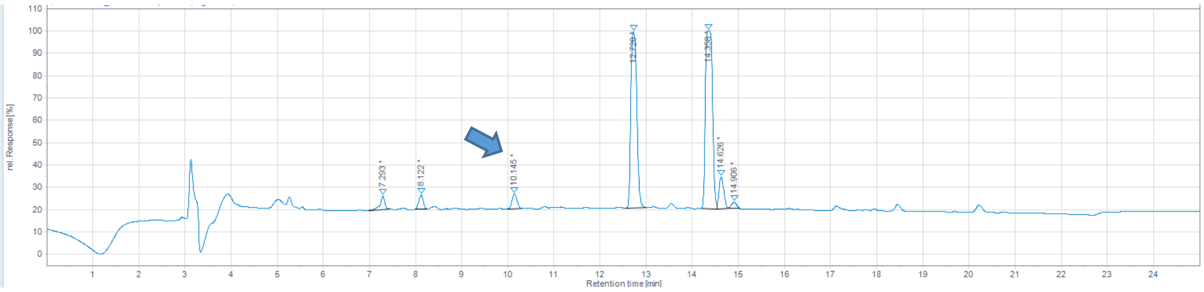

(2S)-N-(2-((1-(1H-indol-3-yl)-3-oxo-3-(tritylamino)propyl)amino)-2-oxoethyl)-2-((S)-2-acetamidopropanamido)-3-phenylpropanamide (8c)

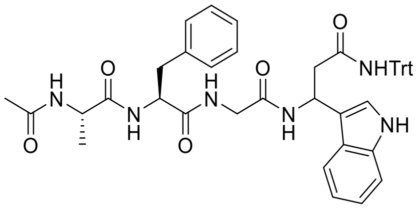

Following general procedure 5, Ac-Ala-Phe-Gly-Asn(Trt)-OH (**7c**) afforded **8c** in 43% HPLC ratio as a mixture of diastereomers (retention time 16.064 min), which could not be fully separated from a side product.

HRMS (ESI/QTOF) m/z: $[M + H]^+$ Calcd for $C_{46}H_{47}N_6O_5^+$ 763.3602; Found 763.3605.

Ac-Ala-Phe-Gly-Asn(Trt)-OH (**7c**)

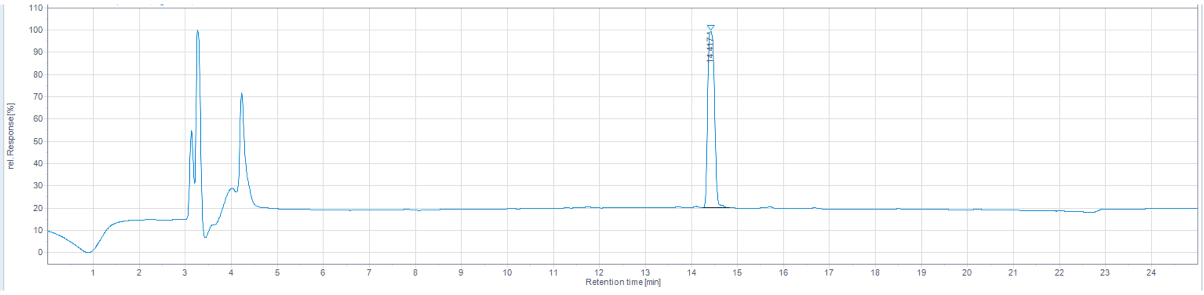

HRMS of **7c** (nanochip-ESI/LTQ-Orbitrap) m/z: $[M + H]^+$ Calcd for $C_{39}H_{42}N_5O_7^+$ 692.3079; Found 692.3075.

HPLC-UV chromatogram at 214 nm of the crude reaction mixture

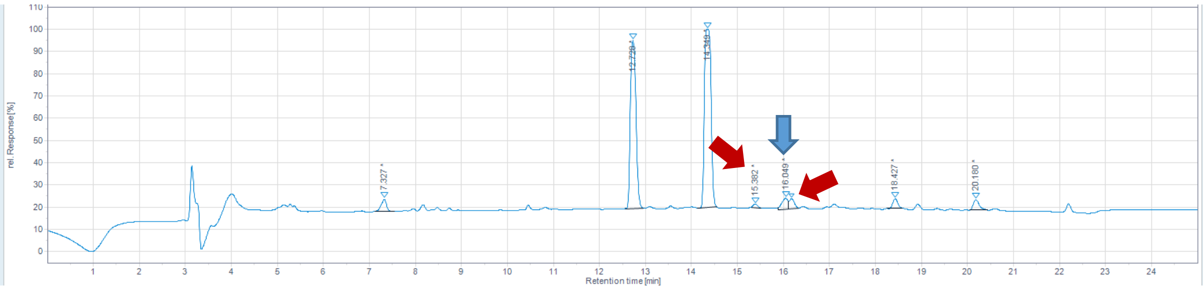

Tert-butyl ((4S,7S)-7-benzyl-13-(1H-indol-3-yl)-4-methyl-2,5,8,11-tetraoxo-3,6,9,12-tetraazaheptadecan-17-yl)carbamate (8d)

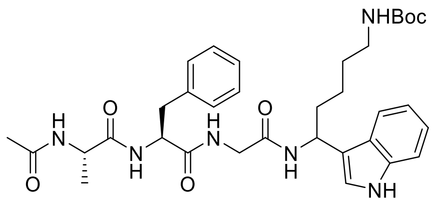

Following general procedure 5, Ac-Ala-Phe-Gly-Lys(Boc)-OH (**7d**) afforded **8d** in 35% HPLC ratio as a mixture of diastereomers (retention time 11.683 min)

HRMS (nanochip-ESI/LTQ-Orbitrap) m/z: $[M + H]^+$ Calcd for $C_{34}H_{47}N_6O_6^+$ 635.3552; Found 635.3564.

Ac-Ala-Phe-Gly-Lys(Boc)-OH (**7d**)

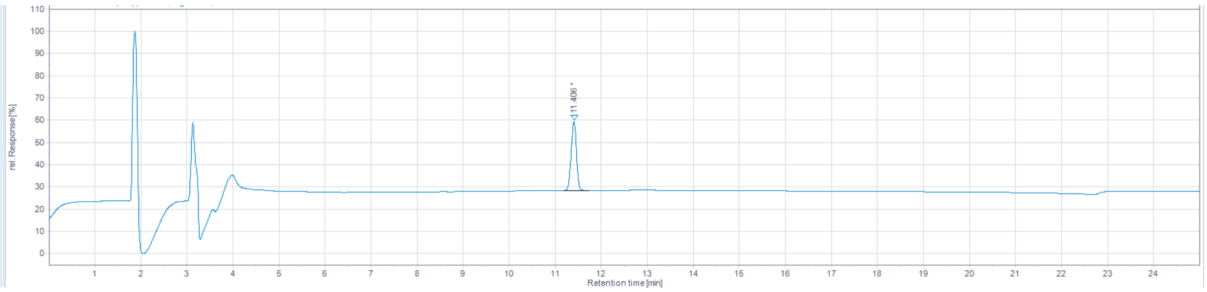

HRMS of **7d** (nanochip-ESI/LTQ-Orbitrap) m/z: $[M + H]^+$ Calcd for $C_{27}H_{42}N_5O_8^+$ 564.3028; Found 564.3027.

HPLC-UV chromatogram at 214 nm of the crude reaction mixture

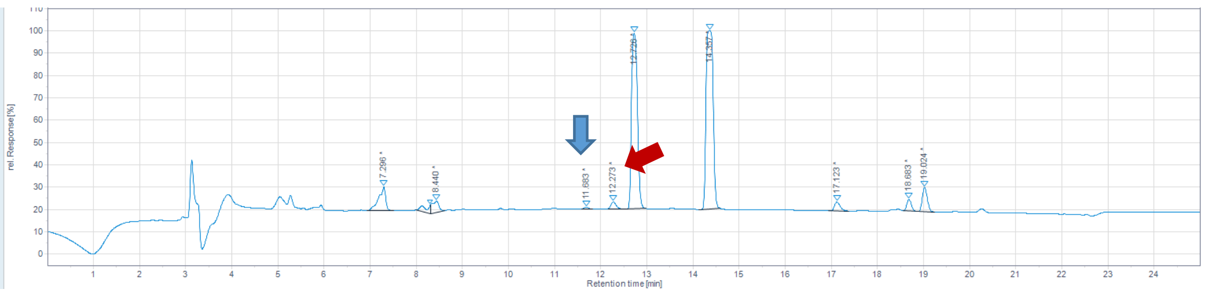

Methyl1-(1-(2-((S)-2-((S)-2-acetamidopropanamido)-3-phenylpropanamido)acetamido) ethyl)-N*α*-((benzyloxy)carbonyl)-L-tryptophanate (8e)

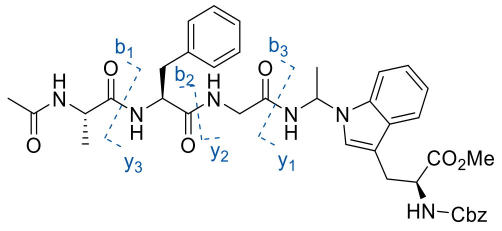

Following general procedure 5, Ac-Ala-Phe-Gly-Ala-OH (**7a**) afforded **8e** in 48% HPLC ratio as a mixture of isomers (retention time 13.873 min + 14.992 min)

HRMS (nanochip-ESI/LTQ-Orbitrap) m/z: $[M + H]^+$ Calcd for $C_{38}H_{45}N_6O_8^+$ 713.3293; Found 713.3287. MS-MS (nanochip-ESI/LTQ-Orbitrap): selected ion 713.3287. Measured b and y ions are reported in the table below

	A	F	G	A*
N-terminal	1	2	3	-
b	-	261.12	318.15	-
C-terminal	3	2	1	-
y	600.28	453.21	396.19	-

HPLC-UV chromatogram at 214 nm of the crude reaction mixture

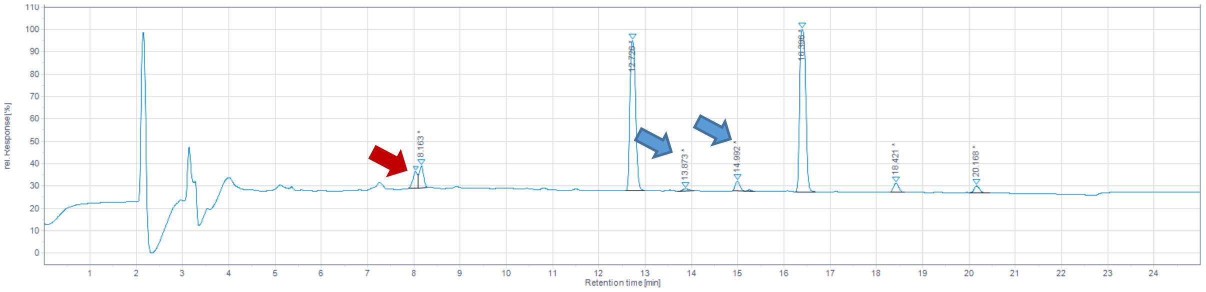

Methyl1-(1-(2-((S)-2-((S)-2-acetamidopropanamido)-3-phenylpropanamido)acetamido) ethyl)-Na-(((benzyloxy)carbonyl)-L-valyl)-L-tryptophanate (8f)

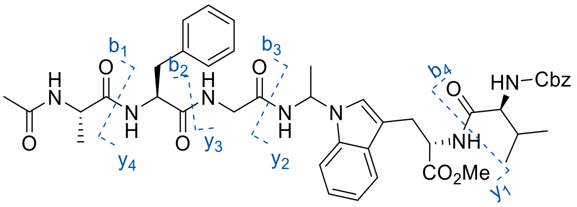

Following general procedure 5, Ac-Ala-Phe-Gly-Ala-OH (**7a**) afforded **8f** in 76% HPLC ratio as a mixture of isomers (retention time 15.302 min + 15.965 min)

HRMS (nanochip-ESI/LTQ-Orbitrap) m/z: $[M + H]^+$ Calcd for $C_{43}H_{54}N_7O_9^+$ 812.3978; Found 812.3973. MS-MS (nanochip-ESI/LTQ-Orbitrap): selected ion 812.3973. Measured b and y ions are reported in the table below

	A	F	G	A*	W*	V
N-terminal	1	2	3	-	4	-
b	-	261.12	318.15	-	-	-
C-terminal	3	2	1	-	1	-
y	-	552.28	495.26	-	579.29	-

HPLC-UV chromatogram at 214 nm of the crude reaction mixture

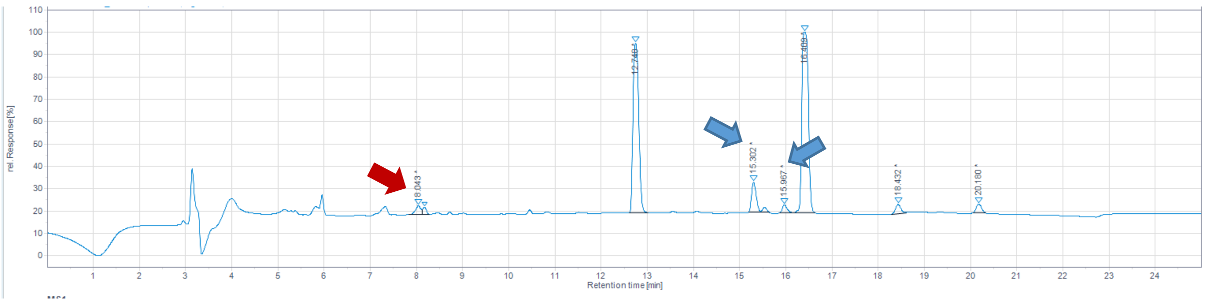

Tert-butyl (4S,7S)-7-benzyl-13-(1H-indol-3-yl)-4-methyl-2,5,8,11-tetraoxo-3,6,9,12-tetraazapentadecan-15-oate (8g)

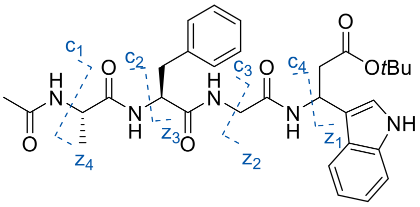

Following general procedure 5, Ac-Ala-Phe-Gly-Asp(O*t*Bu)-OH (**7e**) afforded **8g** in 26% HPLC ratio as a mixture of diastereomers (retention time 13.457 min)

HRMS (nanochip-ESI/LTQ-Orbitrap) m/z: $[M + H]^+$ Calcd for $C_{31}H_{40}N_5O_6^+$ 578.2973; Found 578.2982. MS-MS (nanochip-ESI/LTQ-Orbitrap): selected ion 578.2982. Measured c and z ions are reported in the table below

	A	F	G	A*
N-terminal	1	2	3	4
c	-	-	278.15	335.17
C-terminal	4	3	2	1
z	519.26	-	-	244.13

Ac-Ala-Phe-Gly-Asp(O*t*Bu)-OH (**7e**)

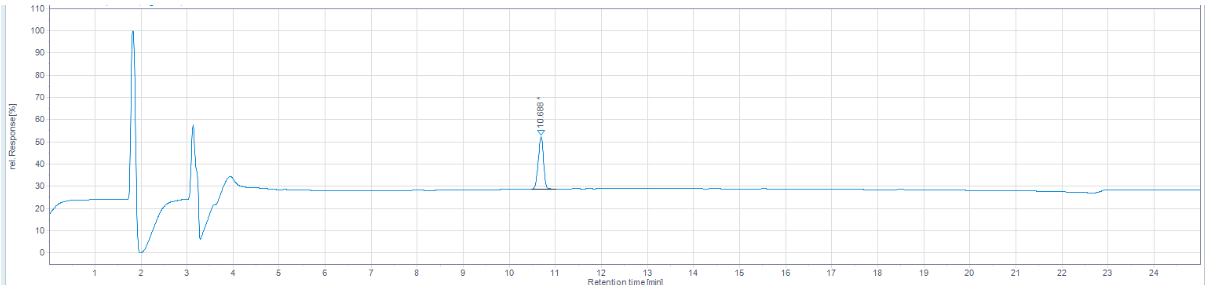

HRMS of **7e** (nanochip-ESI/LTQ-Orbitrap) m/z: $[M + H]^+$ Calcd for $C_{24}H_{35}N_4O_8^+$ 507.2449; Found 507.2448.

HPLC-UV chromatogram at 214 nm of the crude reaction mixture

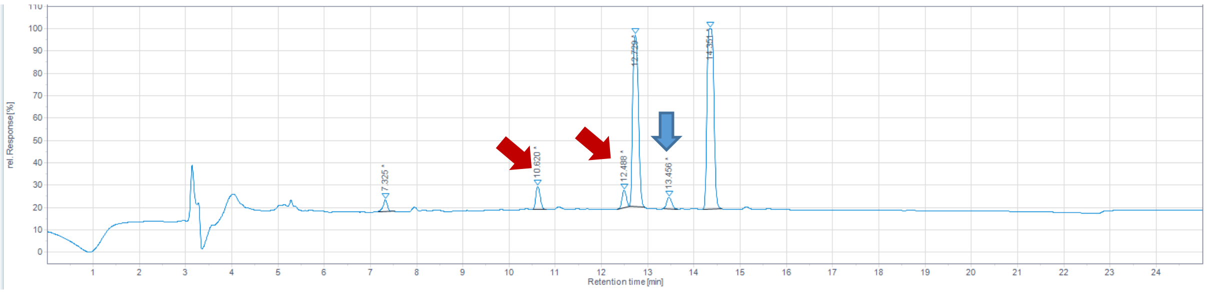

(2S)-N-(2-((1-(1H-indol-3-yl)-2-(1-trityl-1H-imidazol-4-yl)ethyl)amino)-2-oxoethyl)-2-((S)-2-acetamidopropanamido)-3-phenylpropanamide (8h)

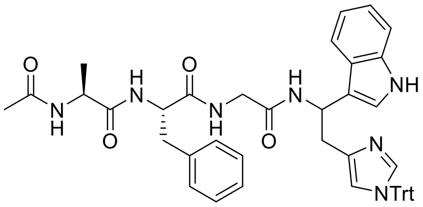

Following general procedure 5, Ac-Ala-Phe-Gly-His(Trt)-OH (**7f**) afforded **8h** in 13% HPLC ratio as a mixture of diastereomers (retention time 13.098 min)

HRMS (ESI/QTOF) m/z: $[M + H]^+$ Calcd for $C_{48}H_{48}N_7O_4^+$ 786.3762; Found 786.3744.

Ac-Ala-Phe-Gly-His(Trt)-OH (**7f**)

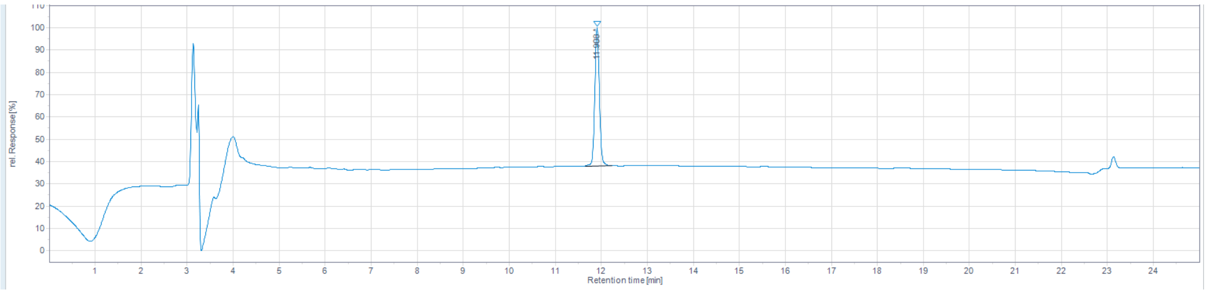

HRMS of **7f** (nanochip-ESI/LTQ-Orbitrap) m/z: $[M + H]^+$ Calcd for $C_{41}H_{43}N_6O_6^+$ 715.3239; Found 715.3237.

HPLC-UV chromatogram at 214 nm of the crude reaction mixture

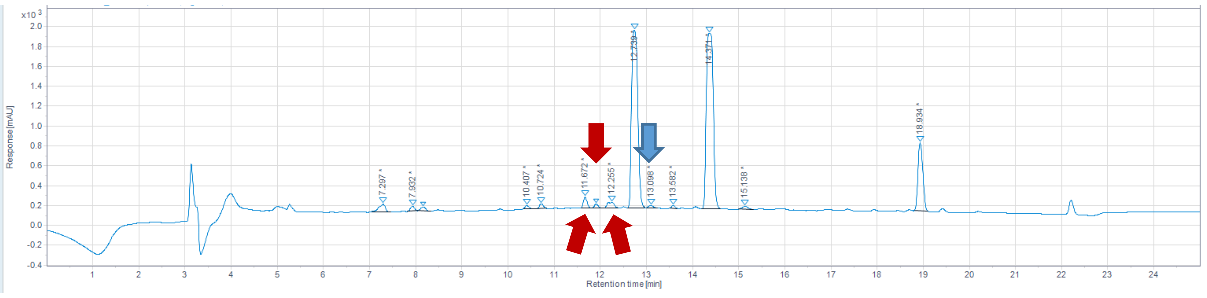

(2S)-N-(2-((1-(1H-indol-3-yl)-4-(3-((2,2,4,6,7-pentamethyl-2,3-dihydrobenzofuran-5-yl)sulfonyl)guanidino)butyl)amino)-2-oxoethyl)-2-((S)-2-acetamidopropanamido)-3-phenylpropanamide (8i)

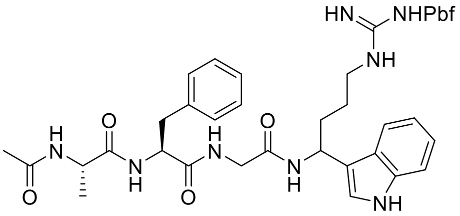

Following general procedure 5, Ac-Ala-Phe-Gly-Arg(Pbf)-OH (**7g**) afforded **8i** in 11% HPLC ratio as a mixture of diastereomers (retention time 16.150 min)

HRMS (ESI/QTOF) m/z: $[M + H_{-1}]^-$ Calcd for $C_{42}H_{53}N_8O_7S^-$ 813.3763; Found 813.3754.

Ac-Ala-Phe-Gly-Arg(Pbf)-OH (**7g**)

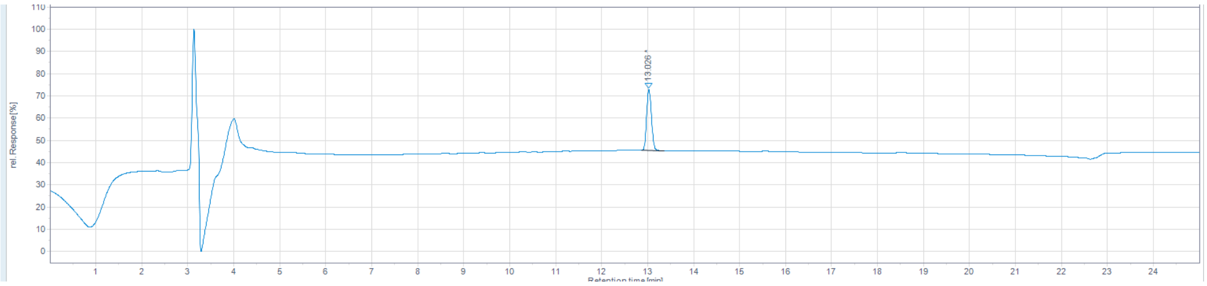

HRMS of **7g** (nanochip-ESI/LTQ-Orbitrap) m/z: $[M + H]^+$ Calcd for $C_{35}H_{50}N_7O_9S^+$ 744.3385; Found 744.3383.

HPLC-UV chromatogram at 214 nm of the crude reaction mixture

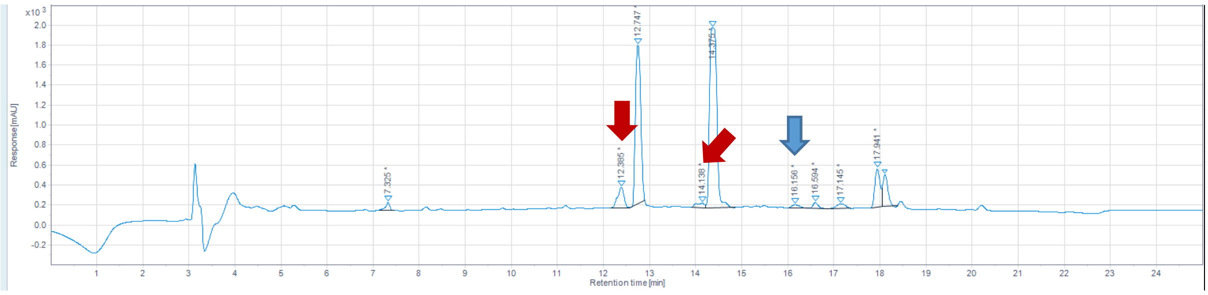

7. References

[1] J. P. Brand, C. Chevalley, R. Scopelliti, J. Waser, *Chem. Eur. J.* **2012**, *18*, 5655–5666.

[2] F. Le Vaillant, M. D. Wodrich, J. Waser, *Chem. Sci.* **2017**, *8*, 1790–1800.

[3] J. Hu, T. Lan, Y. Sun, H. Chen, J. Yao, Y. Rao, *Chem. Commun.* **2015**, *51*, 14929–14932.

[4] H. Uoyama, K. Goushi, K. Shizu, H. Nomura, C. Adachi, *Nature* **2012**, *492*, 234–238.

[5] M. Garreau, F. Le Vaillant, J. Waser, *Angew. Chem. Int. Ed.* **2019**, *58*, 8182–8186.

8. NMR spectra

^{1}H-NMR (400 MHz, Chloroform-*d*) (4a)

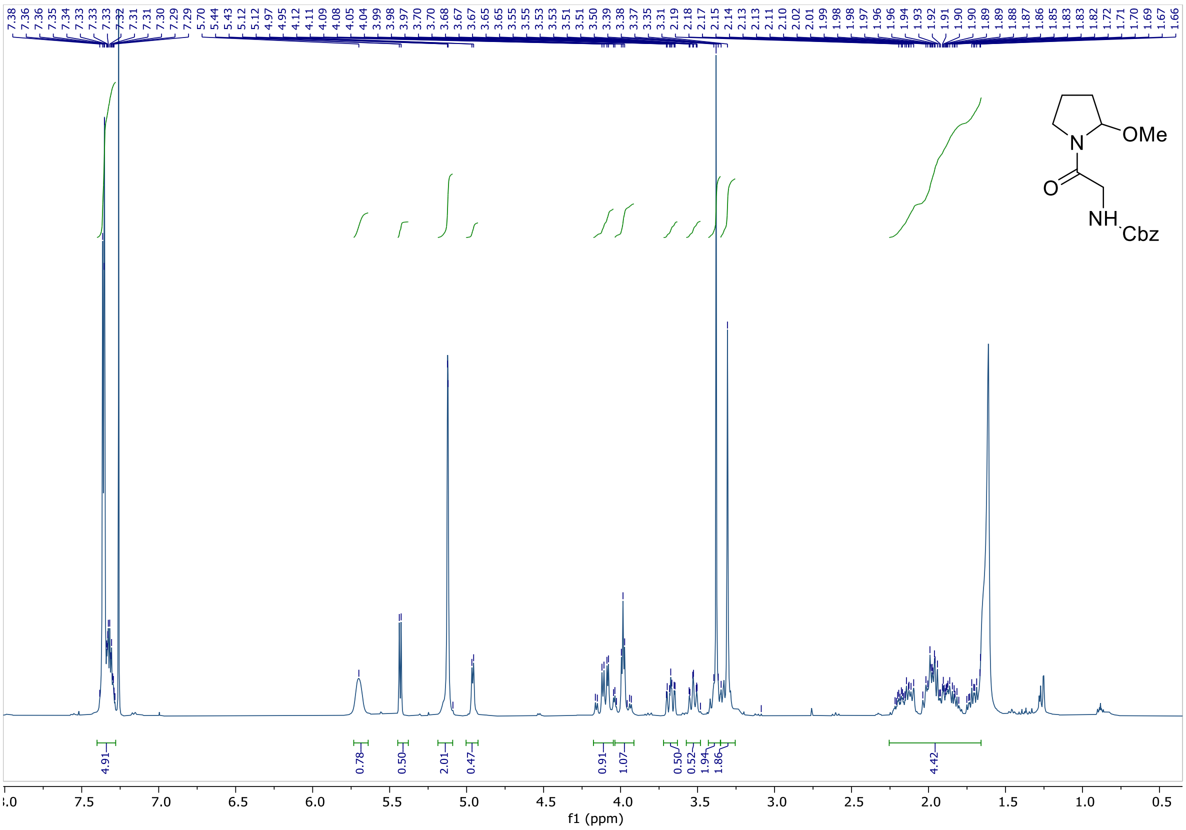

^{13}C-NMR (101 MHz, Chloroform-*d*) (4a)

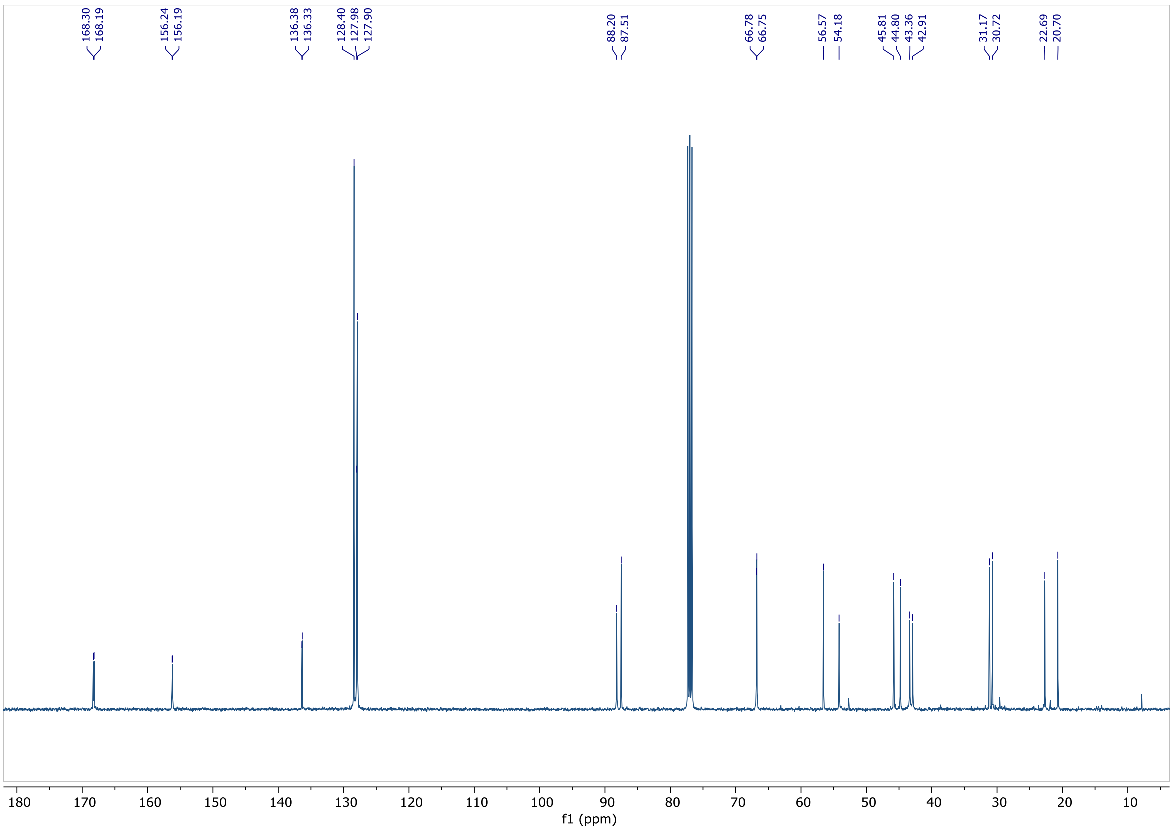

^{1}H-NMR (400 MHz, Chloroform-*d*) (4b)

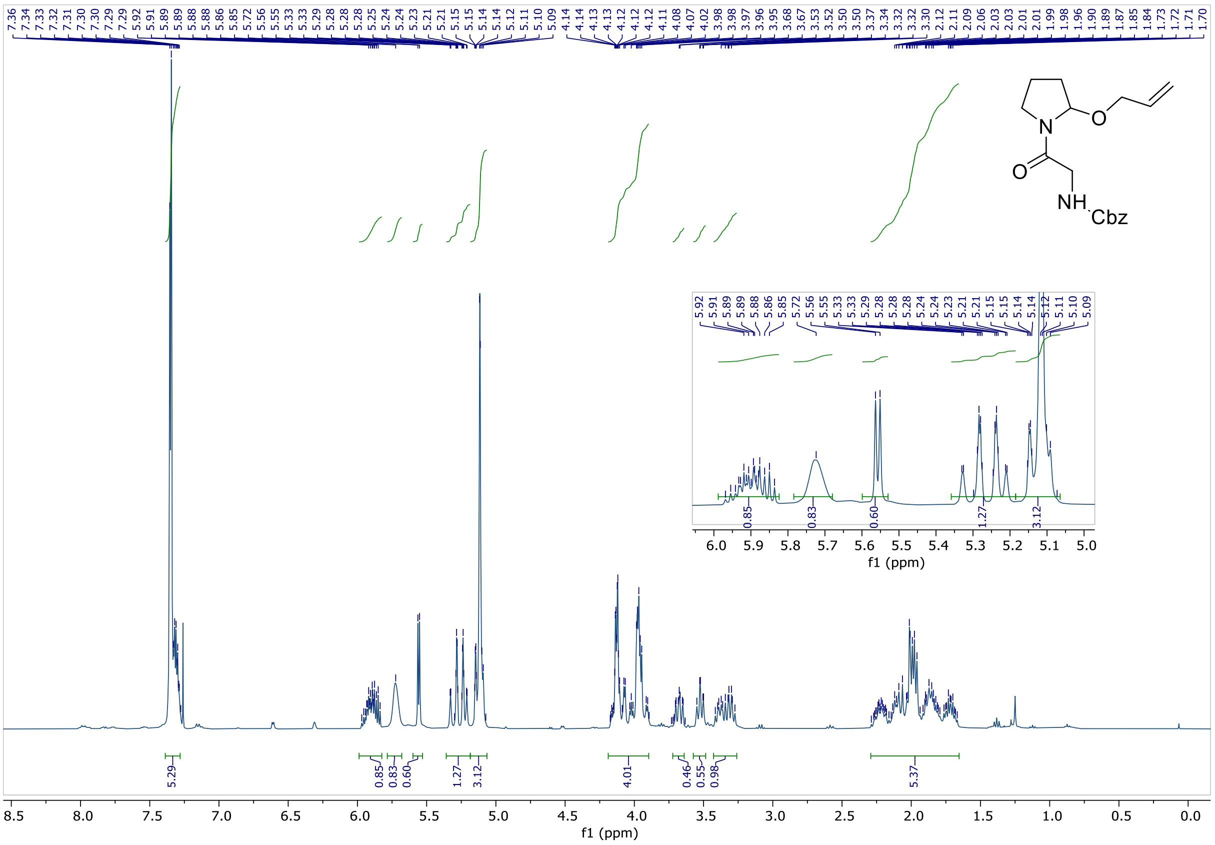

^{13}C-NMR (101 MHz, Chloroform-*d*) (4b)

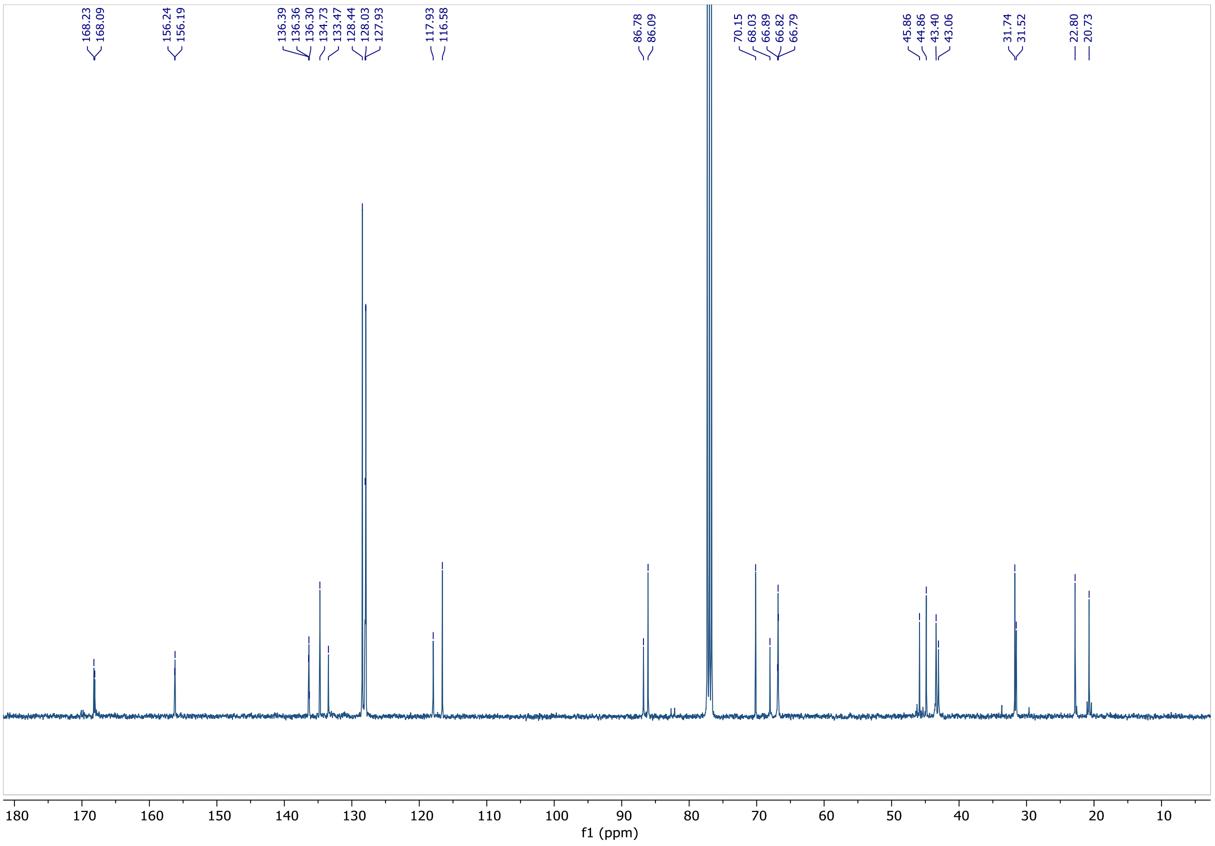

^{1}H-NMR (400 MHz, Chloroform-*d*) (4c)

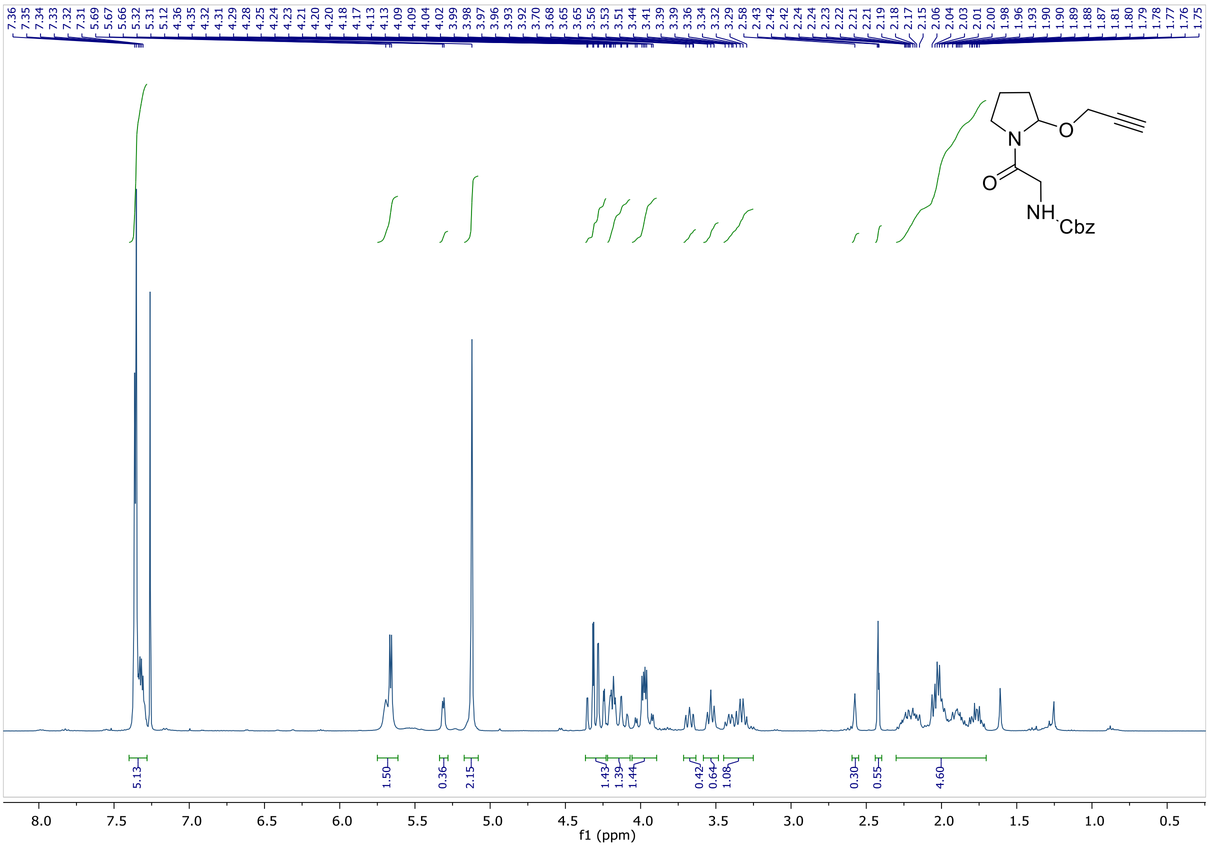

^{13}C-NMR (101 MHz, Chloroform-*d*) (4c)

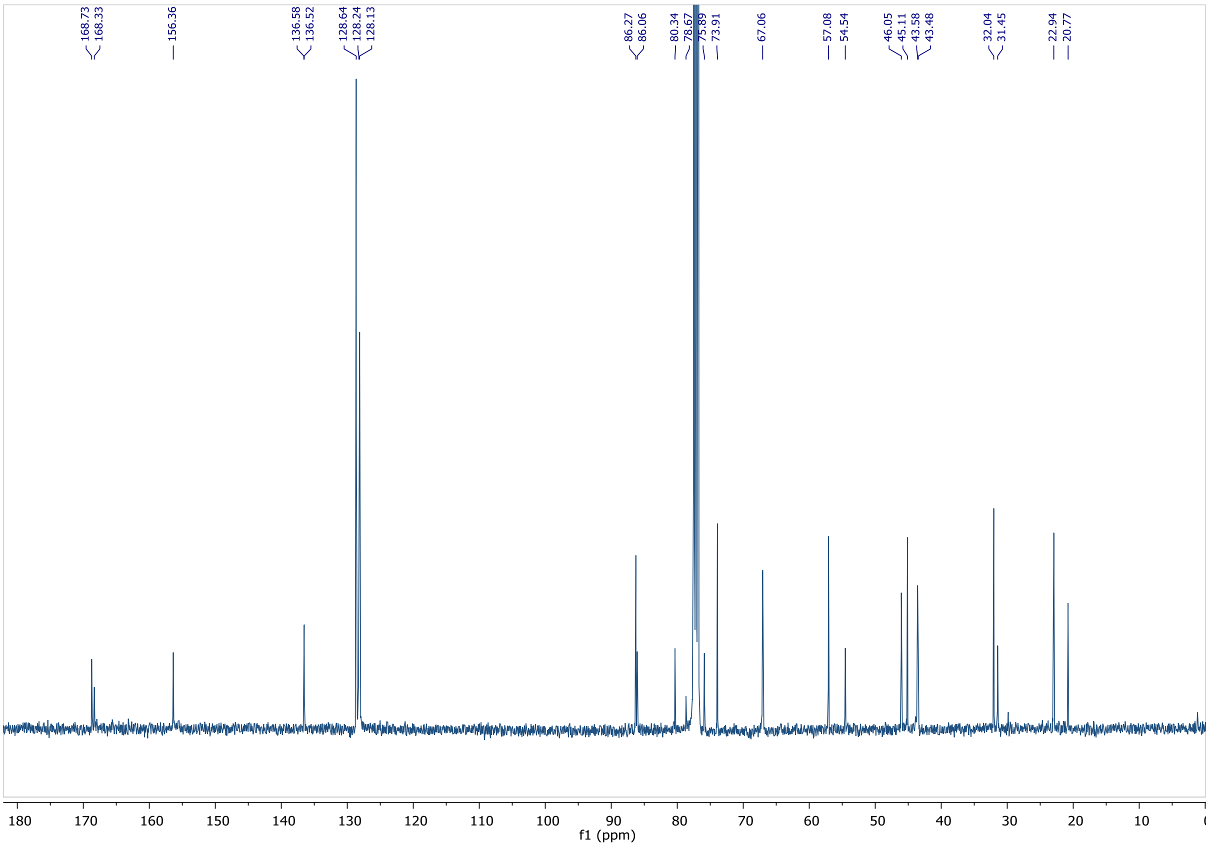

^{1}H-NMR (400 MHz, Chloroform-*d*) (4d)

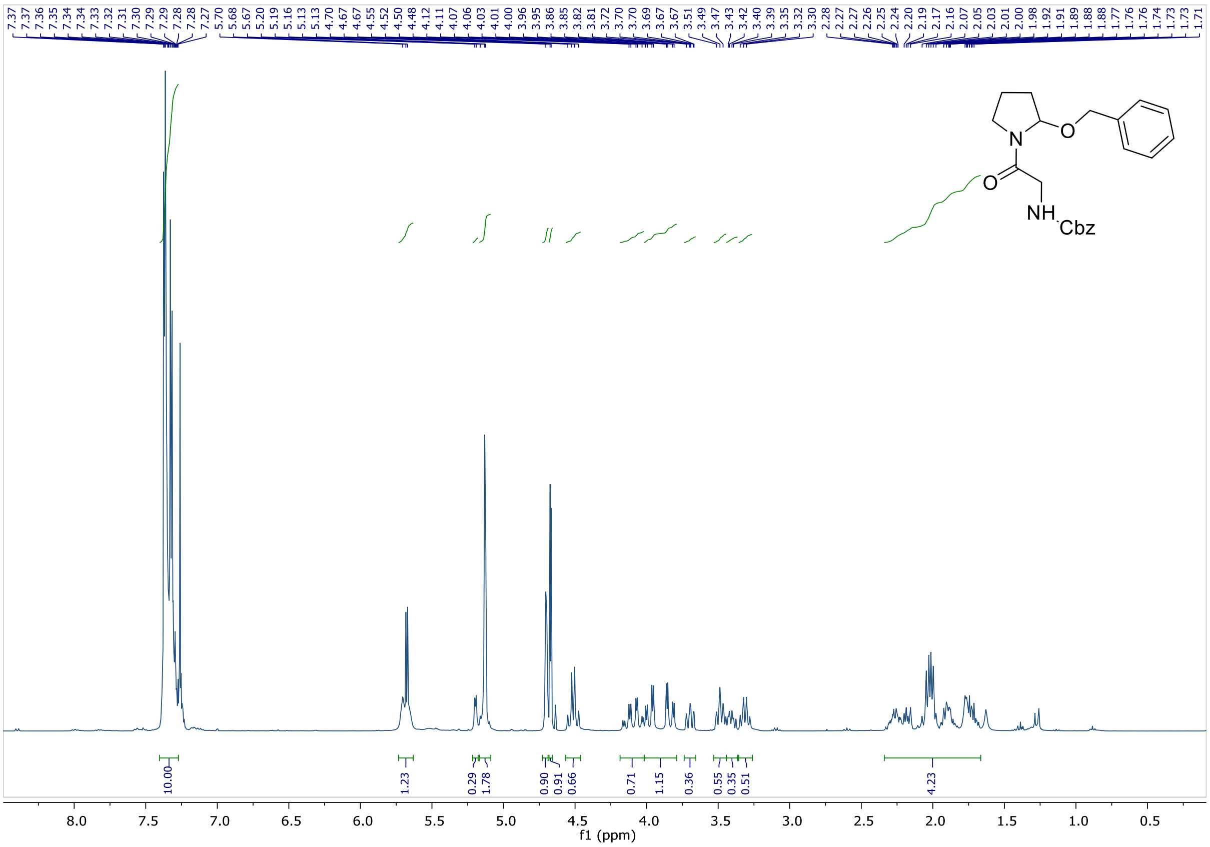

^{13}C-NMR (101 MHz, Chloroform-*d*) (4d)

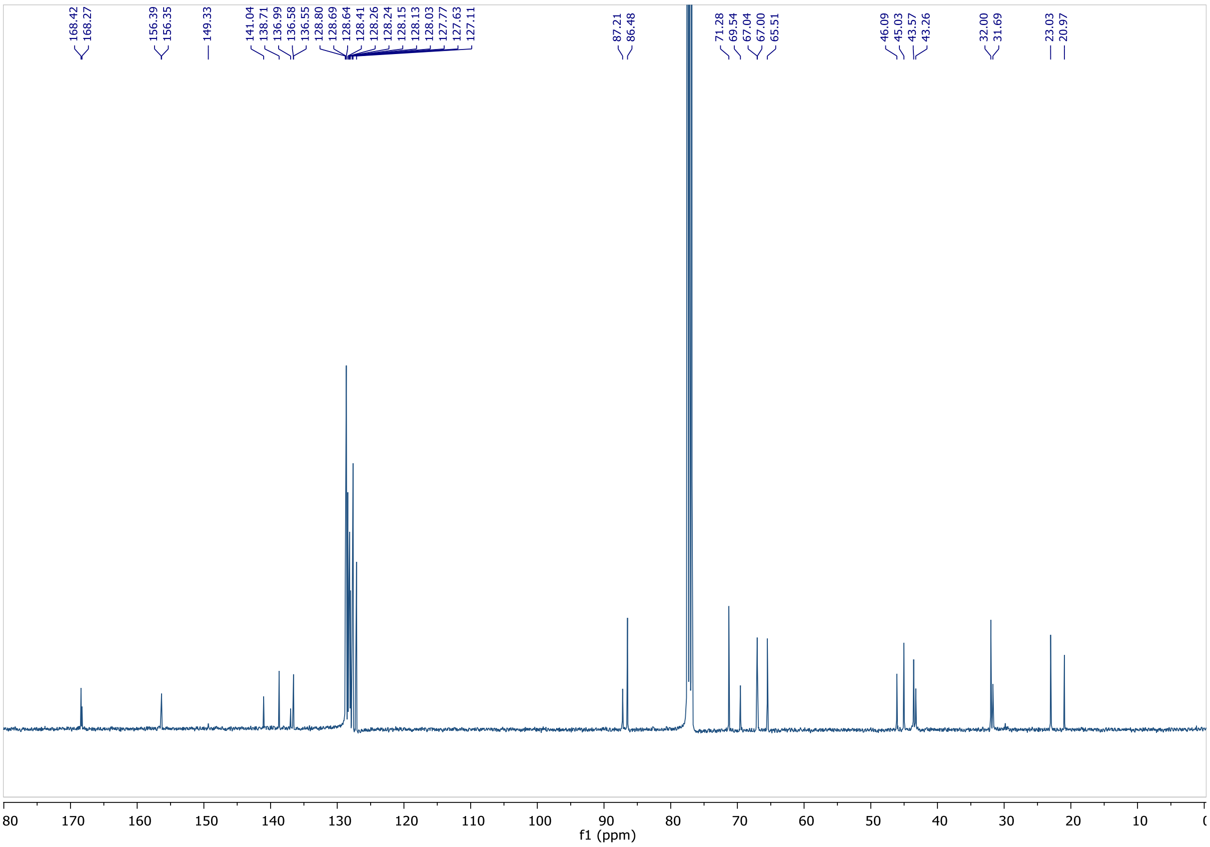

^{1}H-NMR (400 MHz, Chloroform-*d*) (4e)

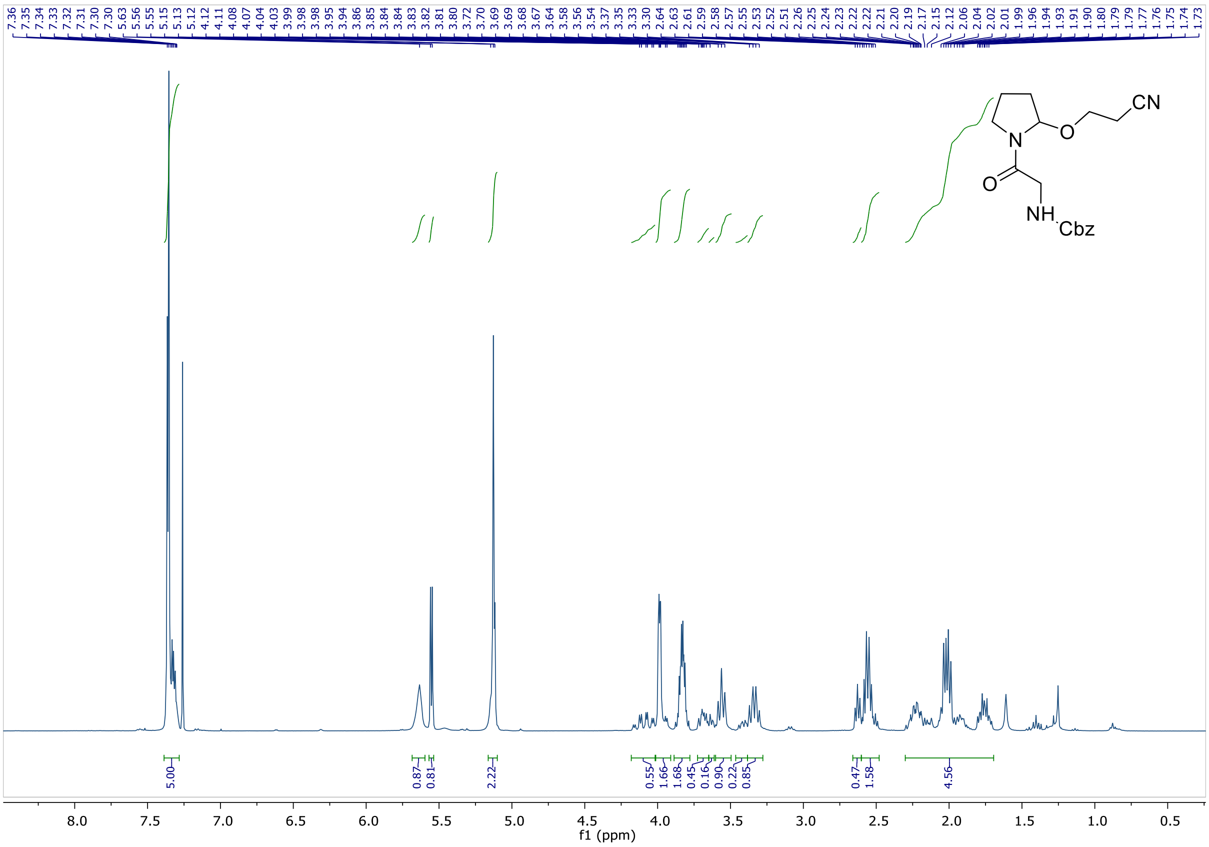

^{13}C-NMR (101 MHz, Chloroform-*d*) (4e)

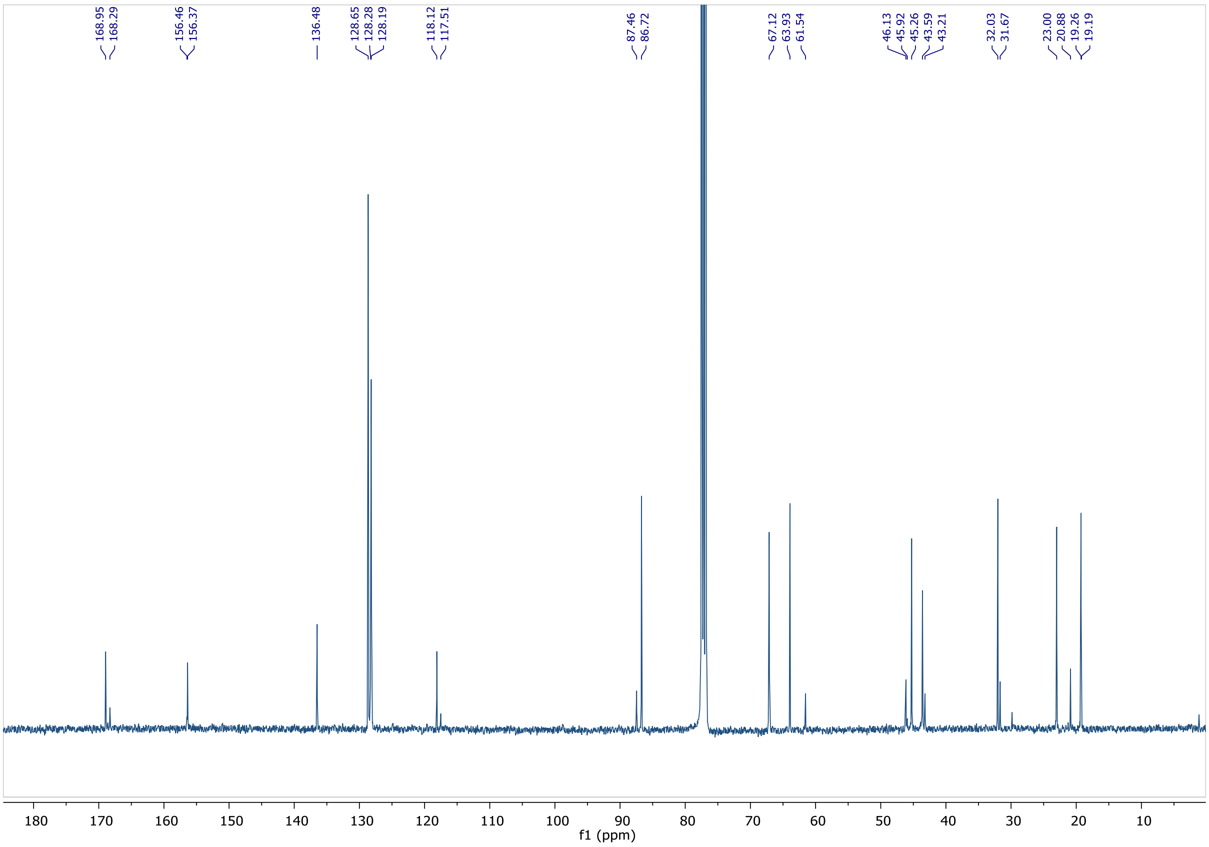

^{1}H-NMR (400 MHz, Chloroform-*d*) (4f)

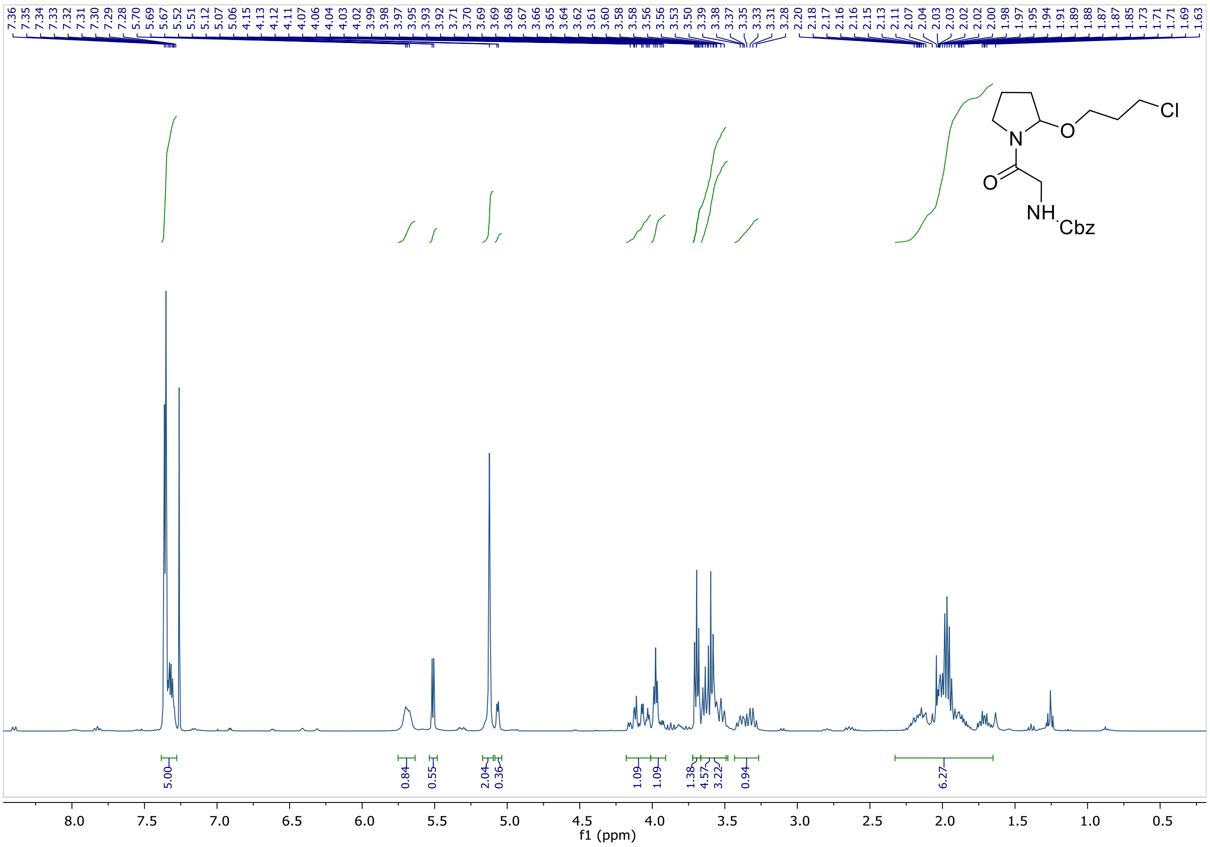

^{13}C-NMR (101 MHz, Chloroform-*d*) (4f)

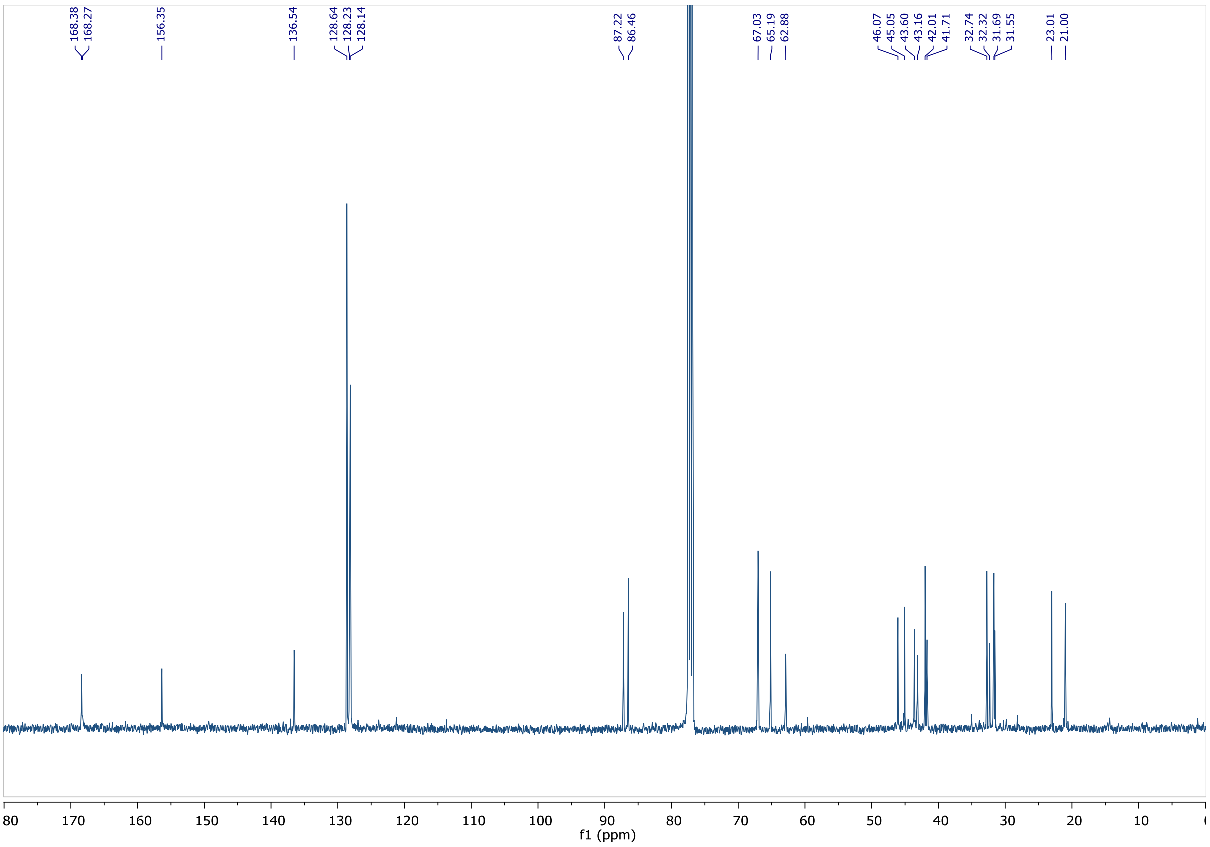

^{1}H-NMR (400 MHz, Chloroform-*d*) (4g)

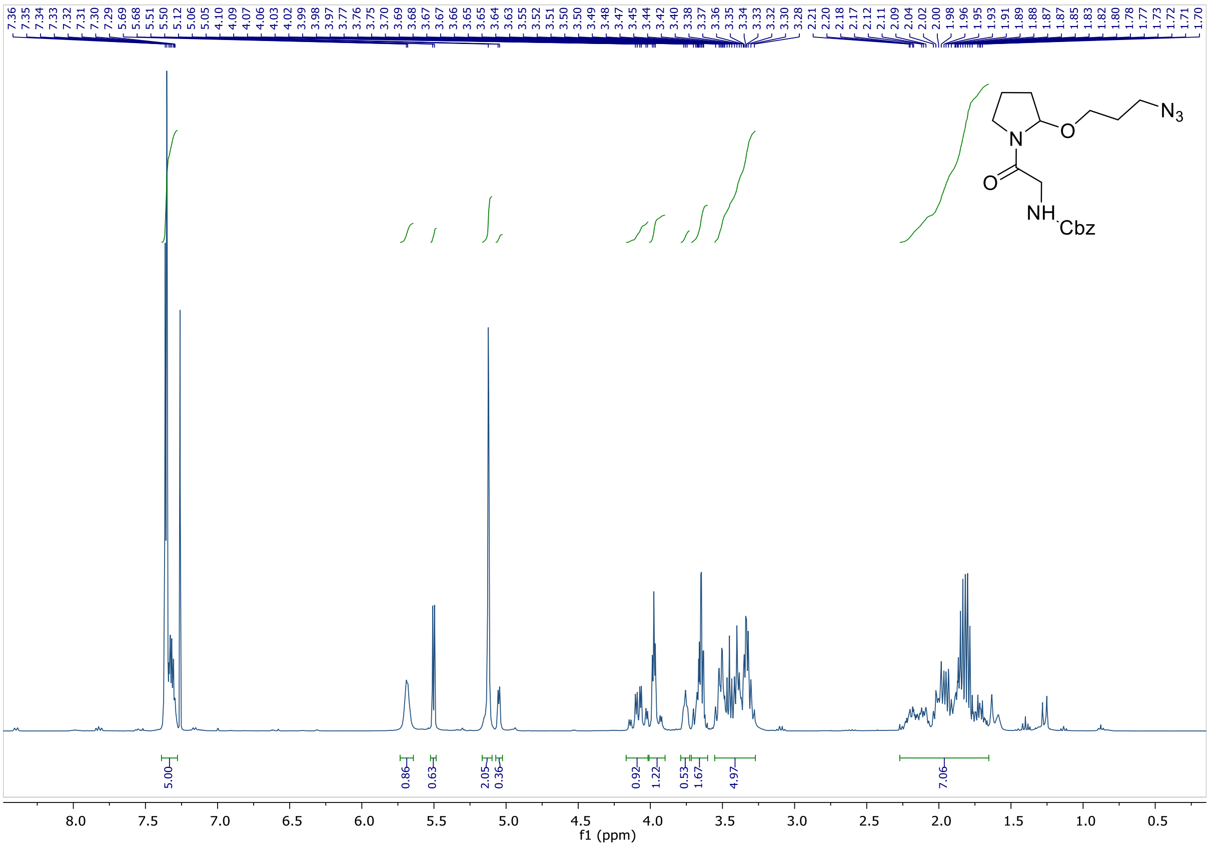

^{13}C-NMR (101 MHz, Chloroform-*d*) (4g)

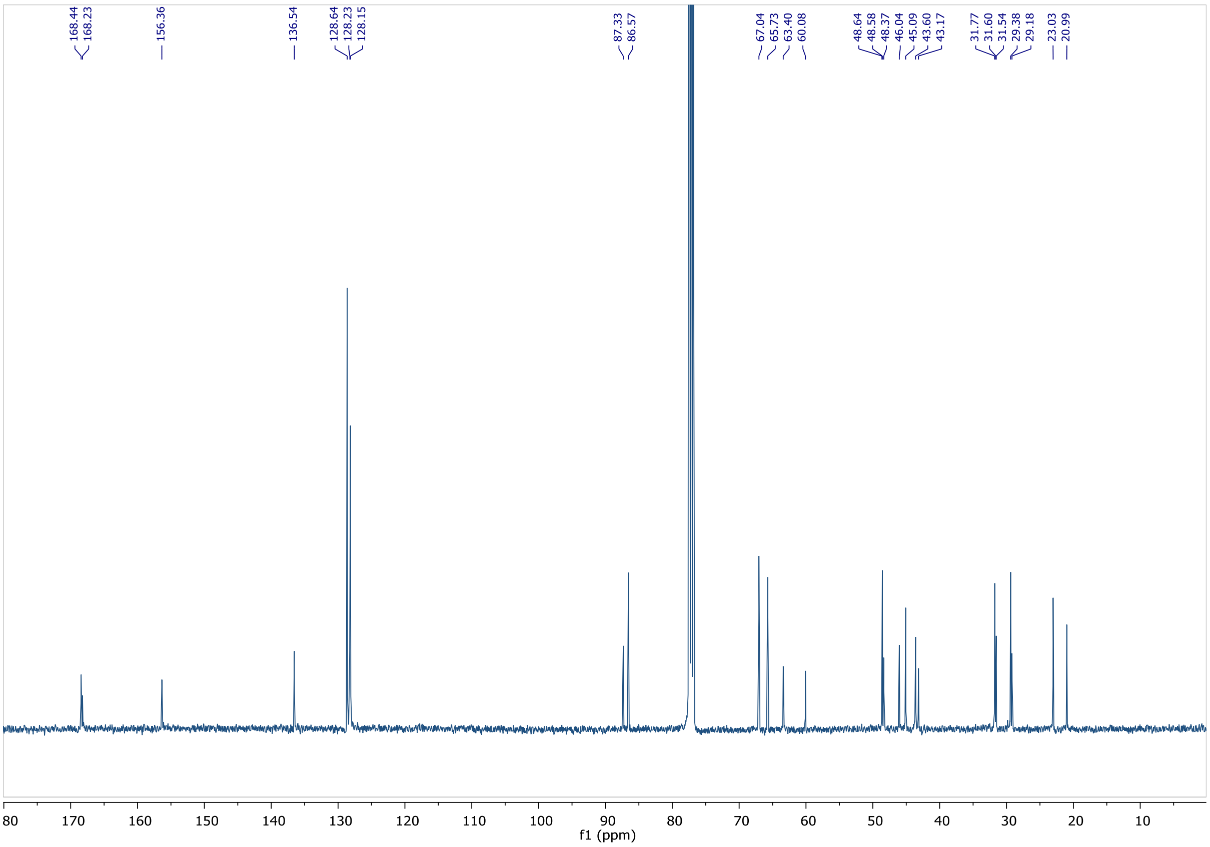

^{1}H-NMR (400 MHz, Chloroform-*d*) (4h)

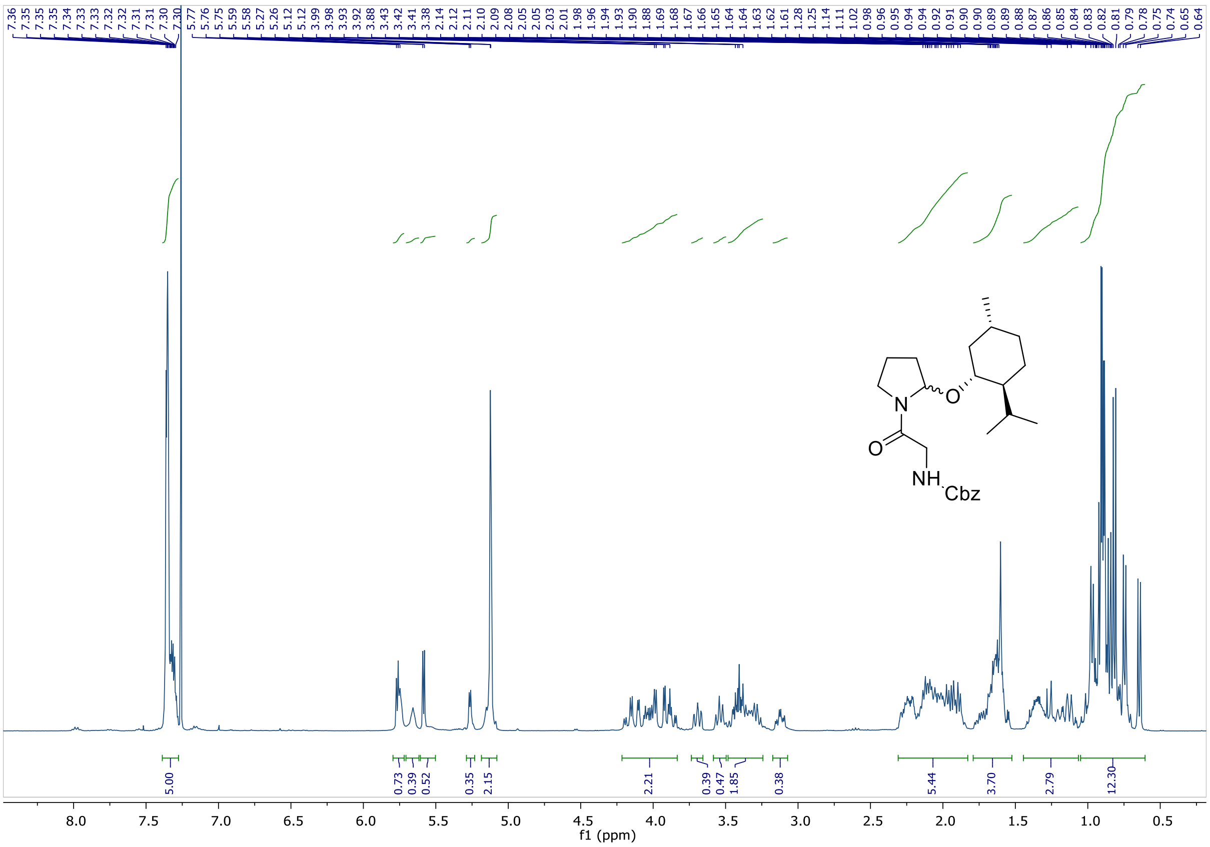

^{13}C-NMR (101 MHz, Chloroform-*d*) (4h)

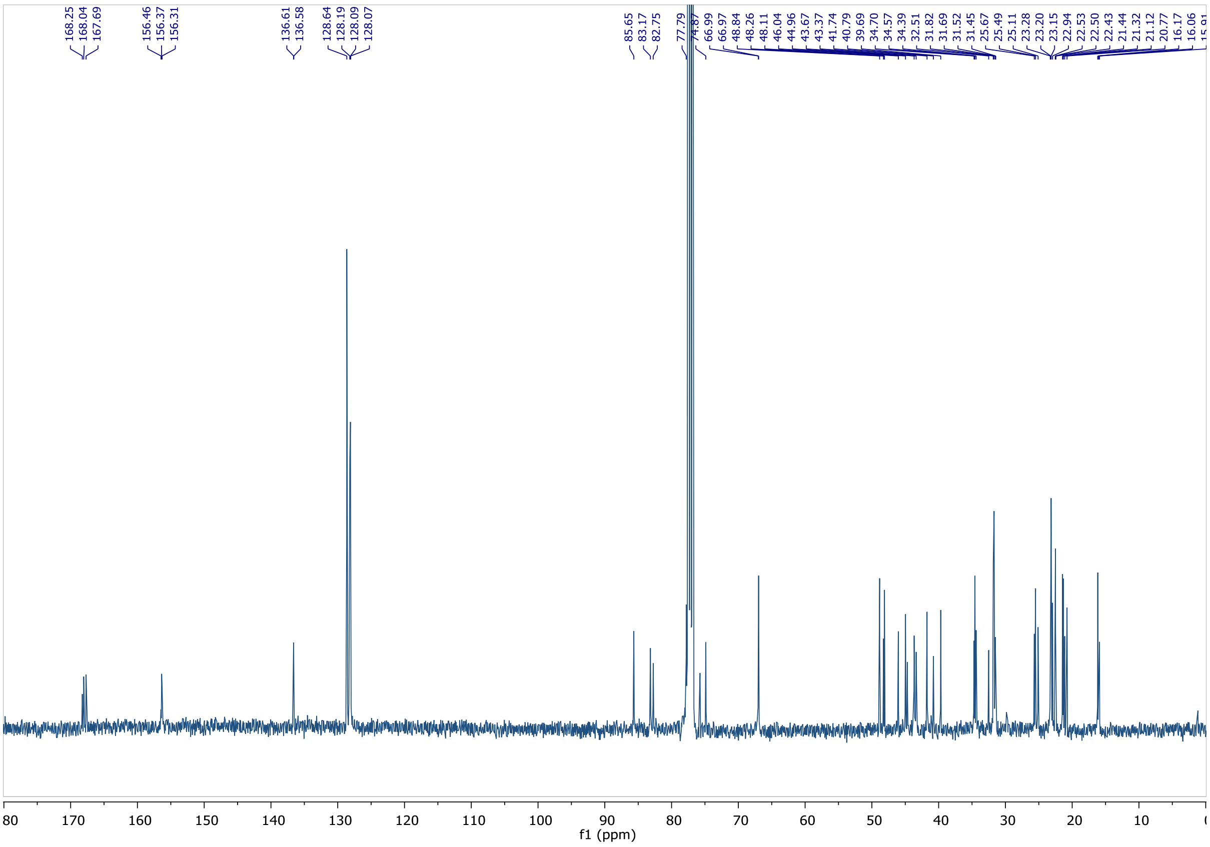

^{1}H-NMR (400 MHz, Chloroform-*d*) (4i)

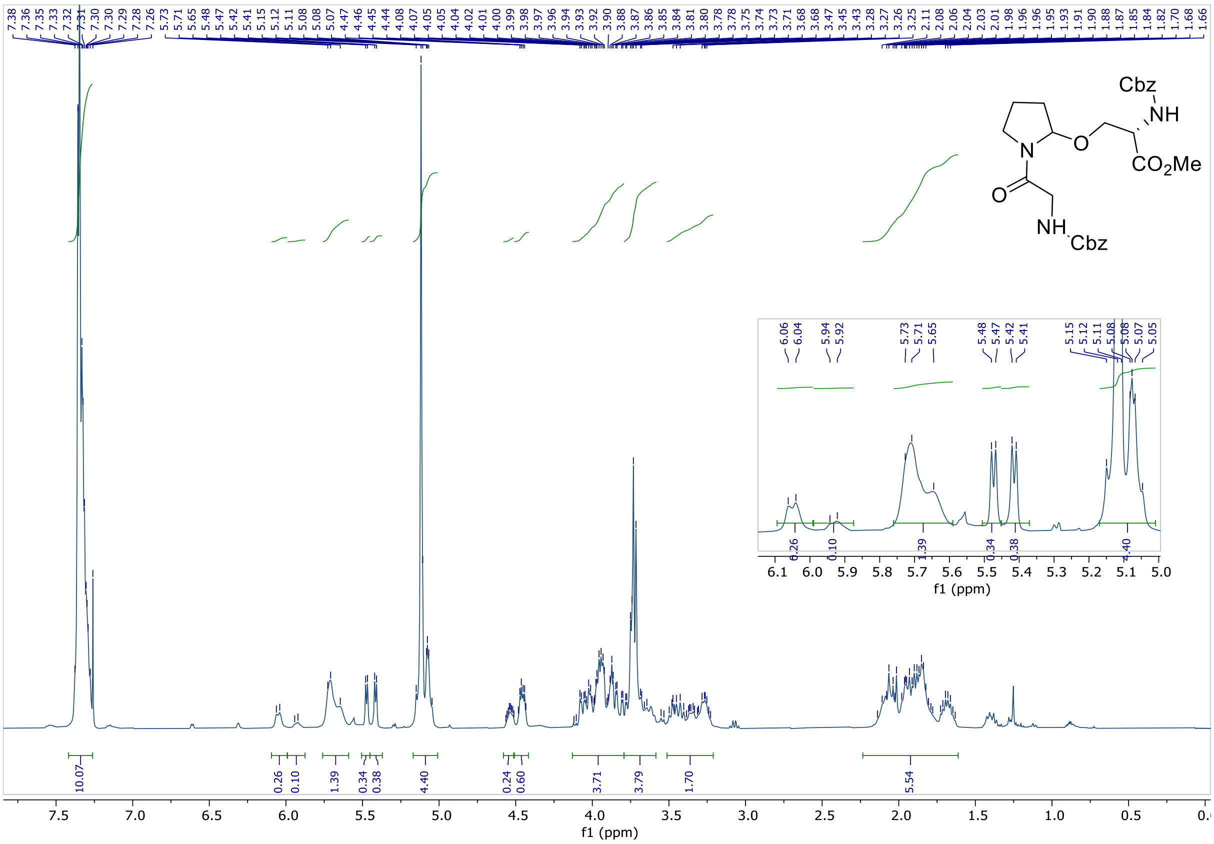

^{13}C-NMR (101 MHz, Chloroform-*d*) (4i)

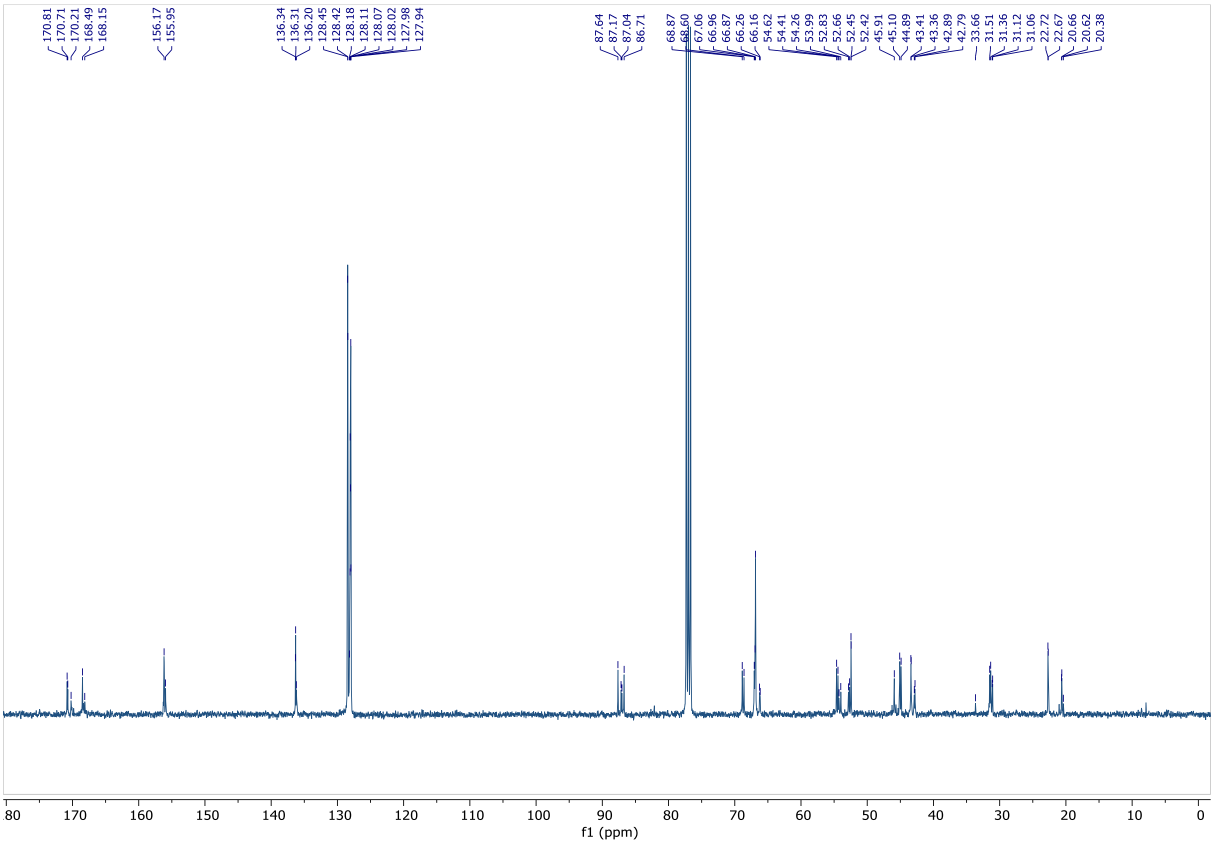

^{1}H-NMR (400 MHz, Chloroform-*d*) (4j)

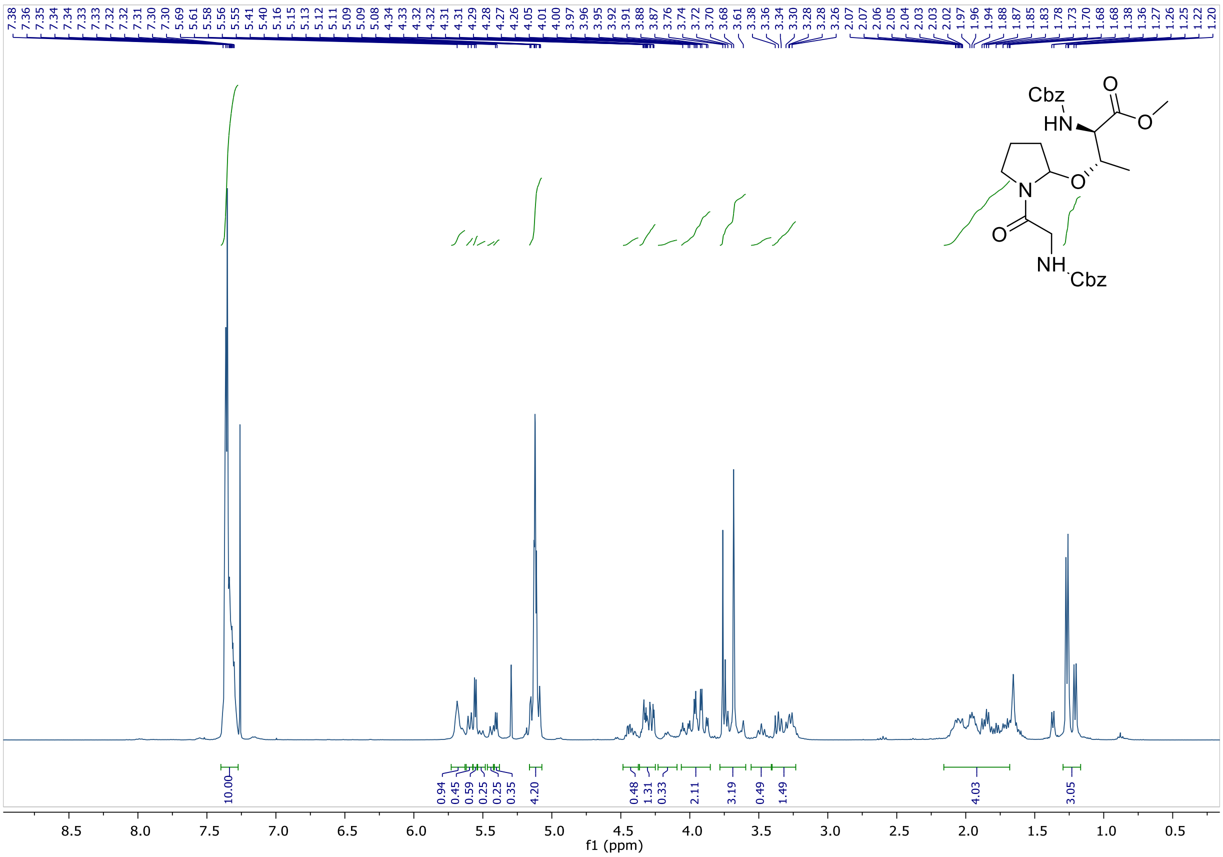

^{13}C-NMR (101 MHz, Chloroform-*d*) (4j)

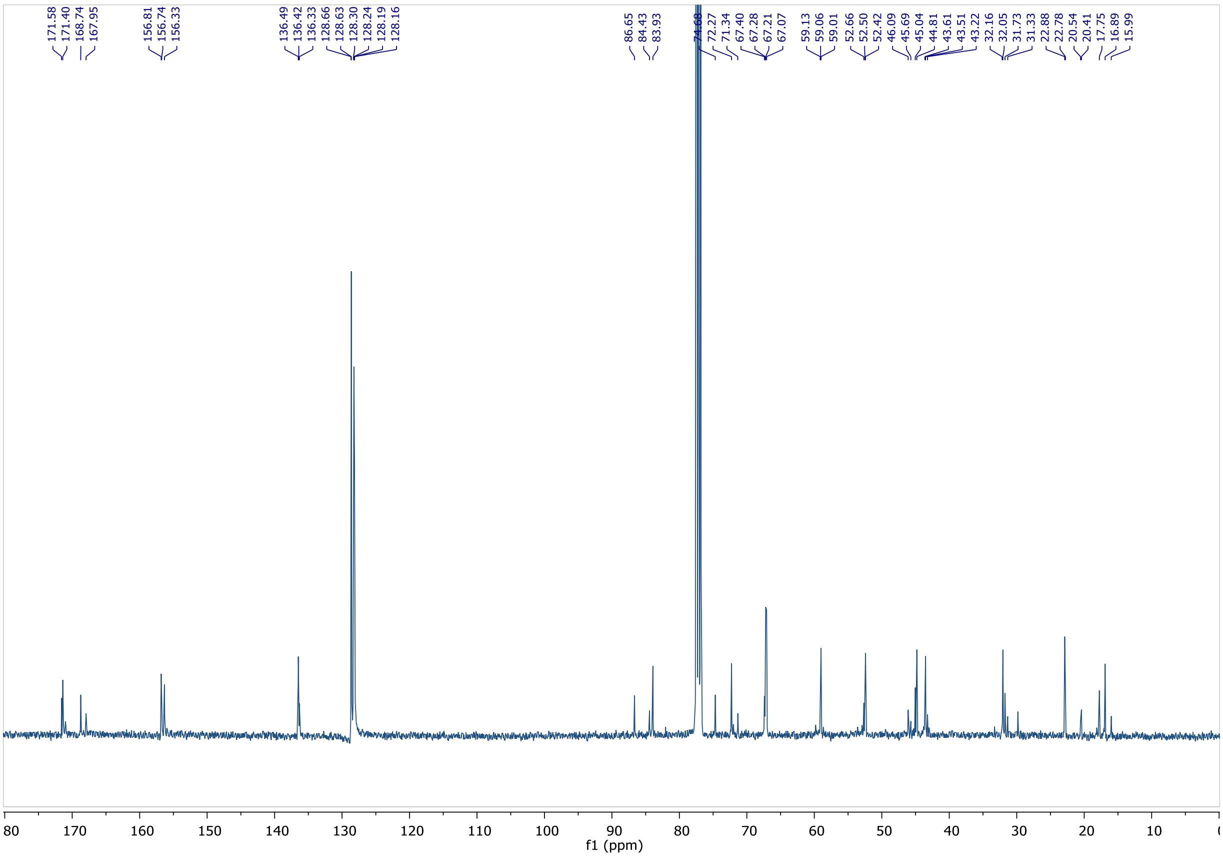

^{1}H-NMR (400 MHz, Chloroform-*d*) (4k)

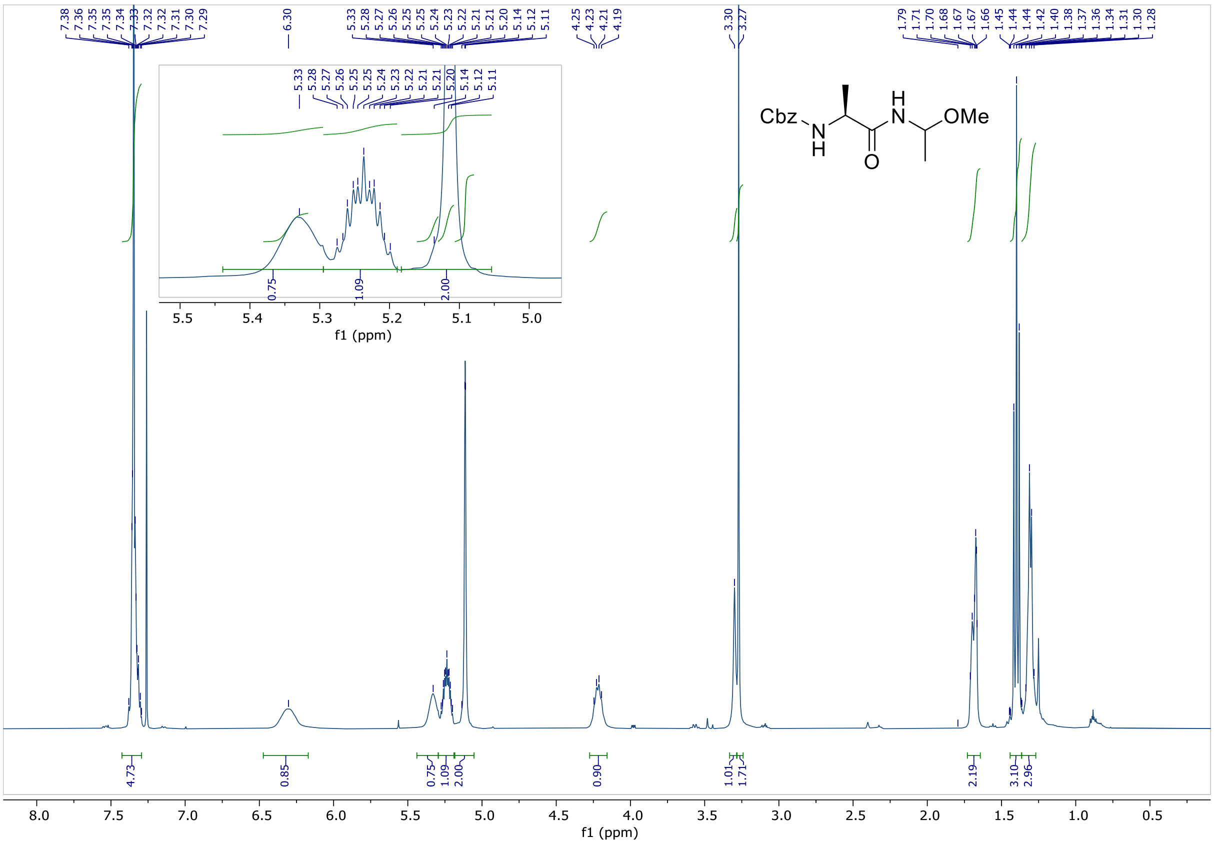

^{13}C-NMR (101 MHz, Chloroform-*d*) (4k)

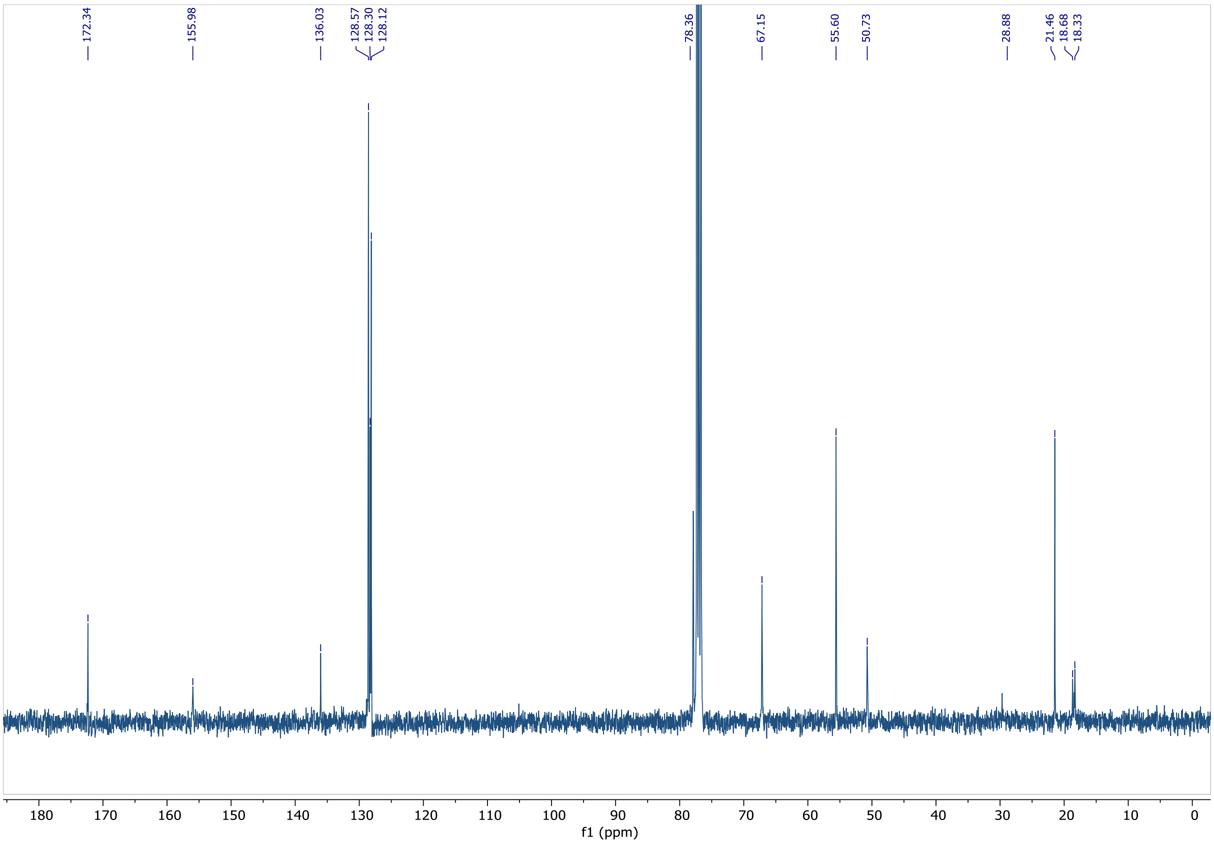

^{1}H-NMR (400 MHz, Acetonitrile-d_3) (4l)

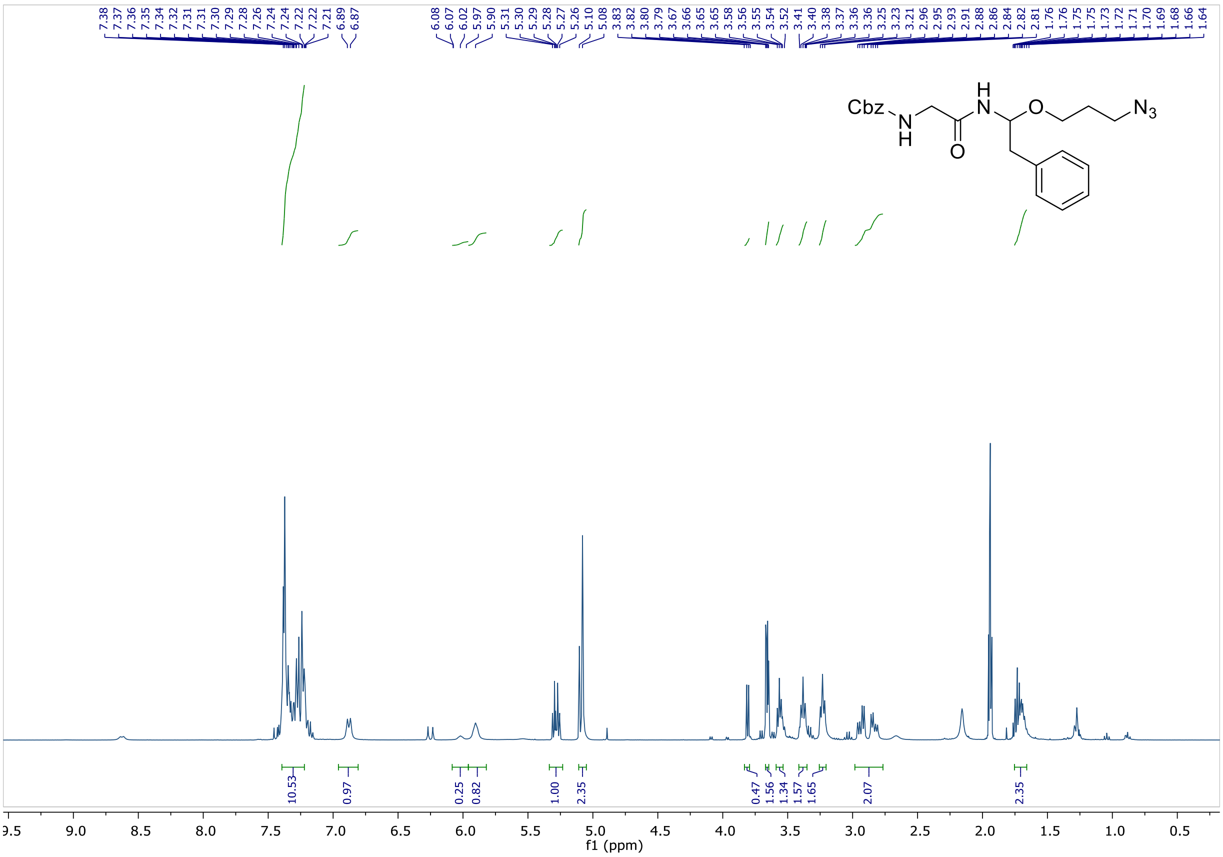

^{13}C-NMR (101 MHz, Acetonitrile-d_3) (4l)

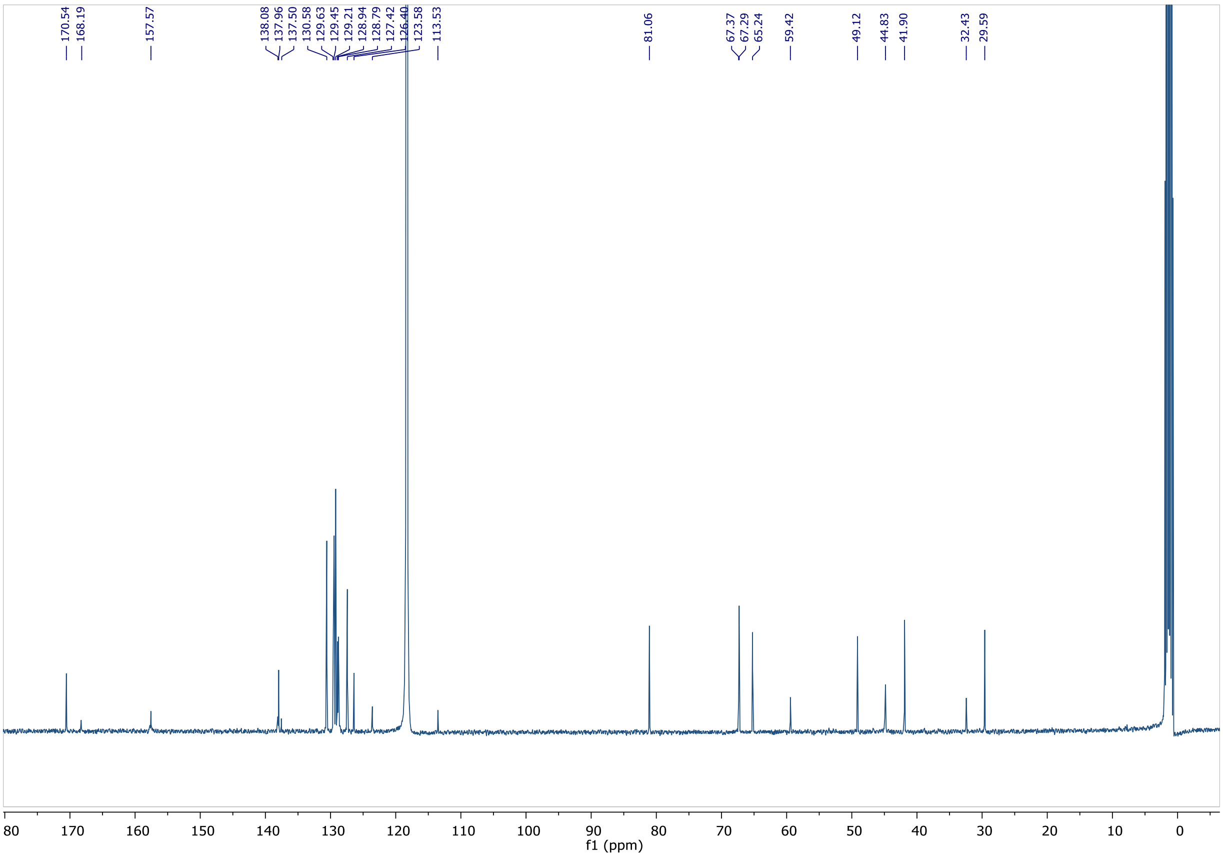

^{1}H-NMR (400 MHz, Acetonitrile-d_3) (4m)

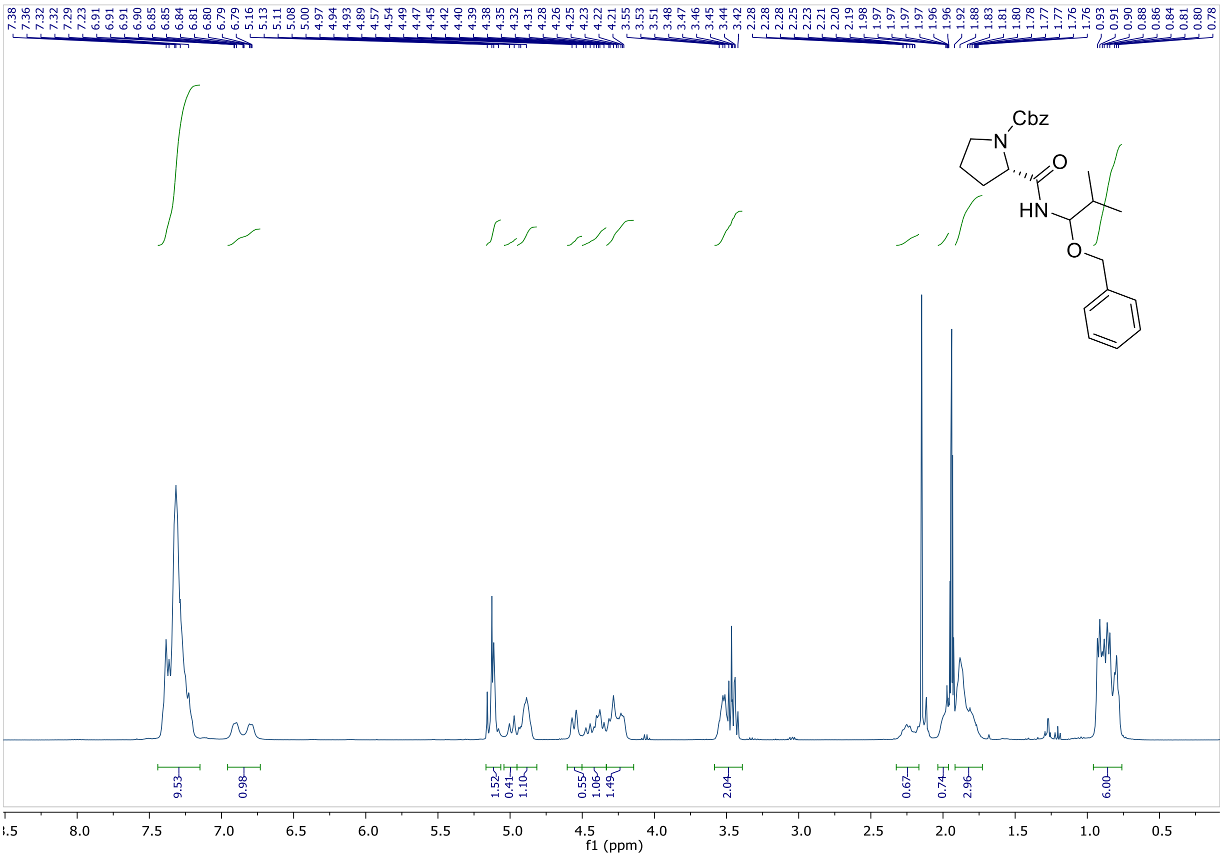

^{13}C-NMR (101 MHz, Acetonitrile-d_3) (4m)

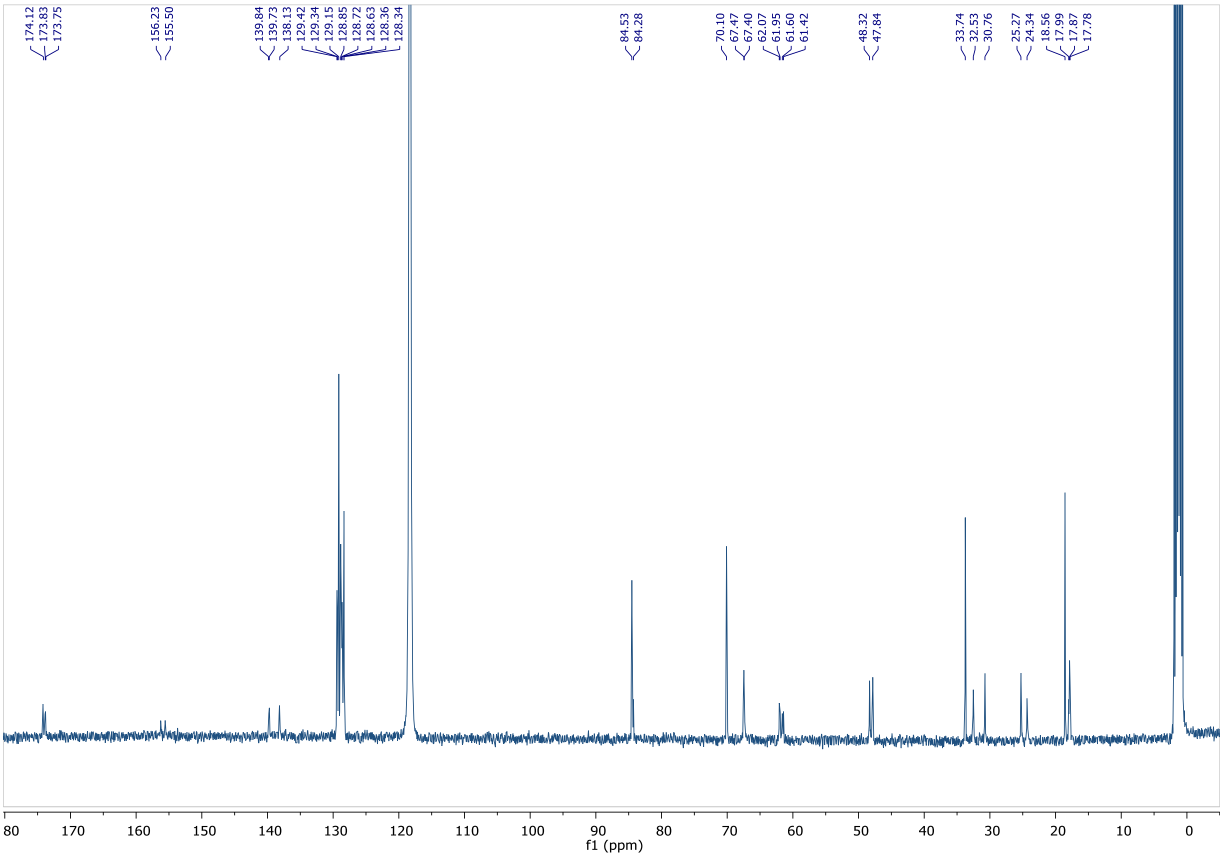

^{1}H-NMR (400 MHz, Chloroform-*d*) (5a)

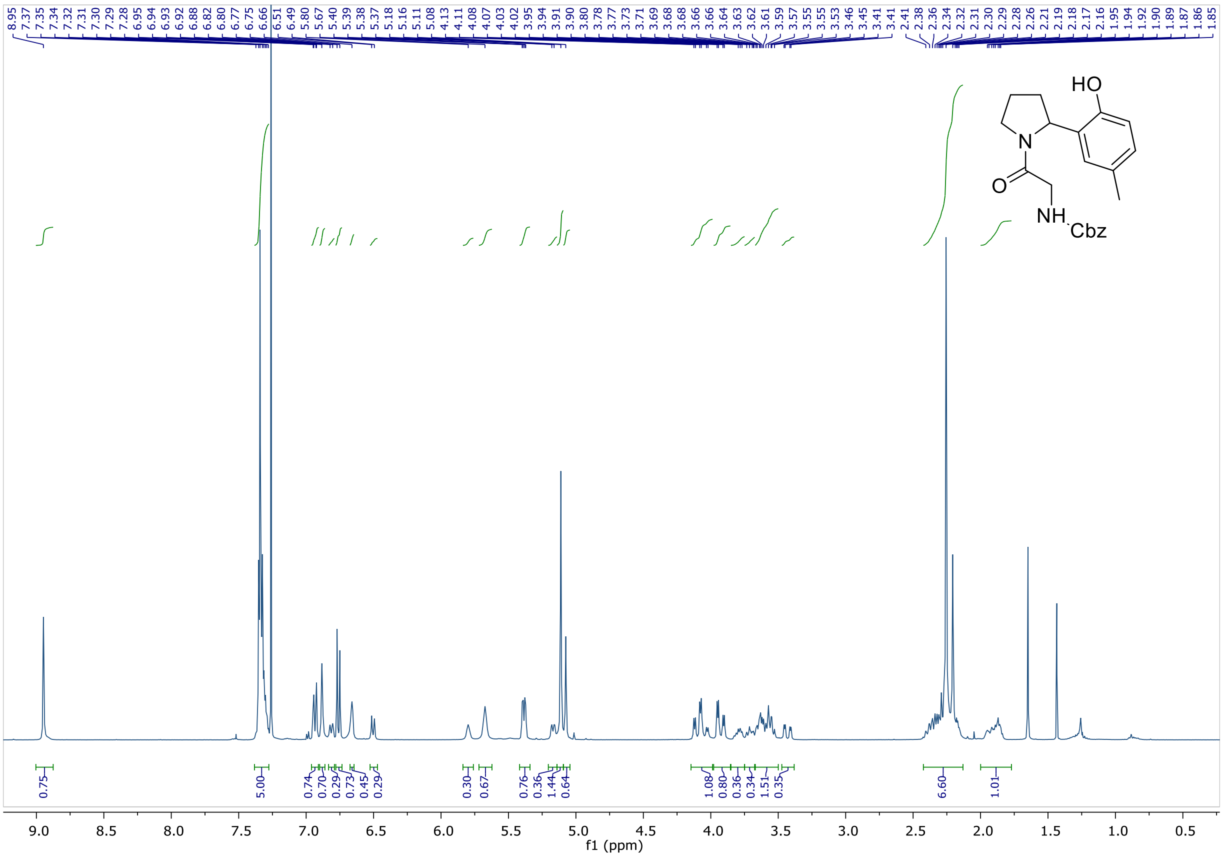

^{13}C-NMR (101 MHz, Chloroform-*d*) (5a)

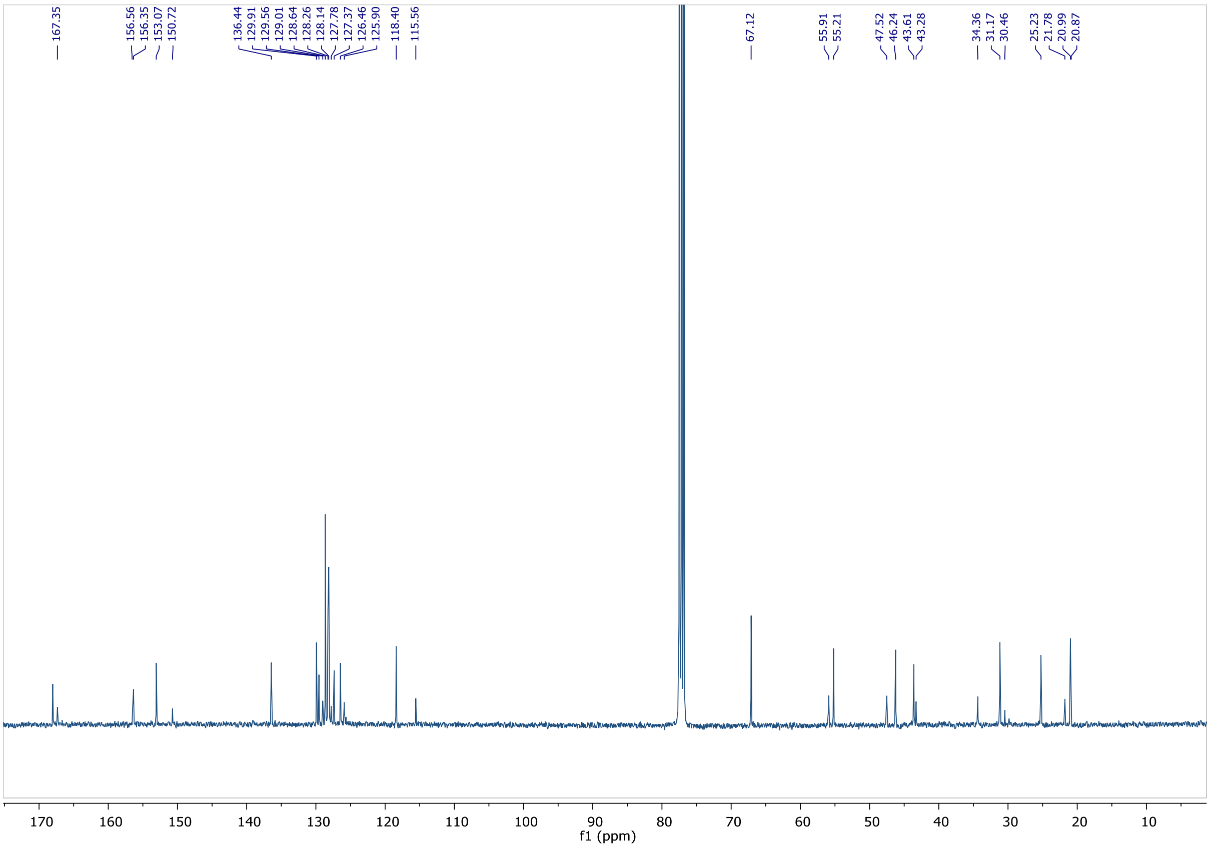

^{1}H-NMR (400 MHz, Chloroform-*d*) (5b)

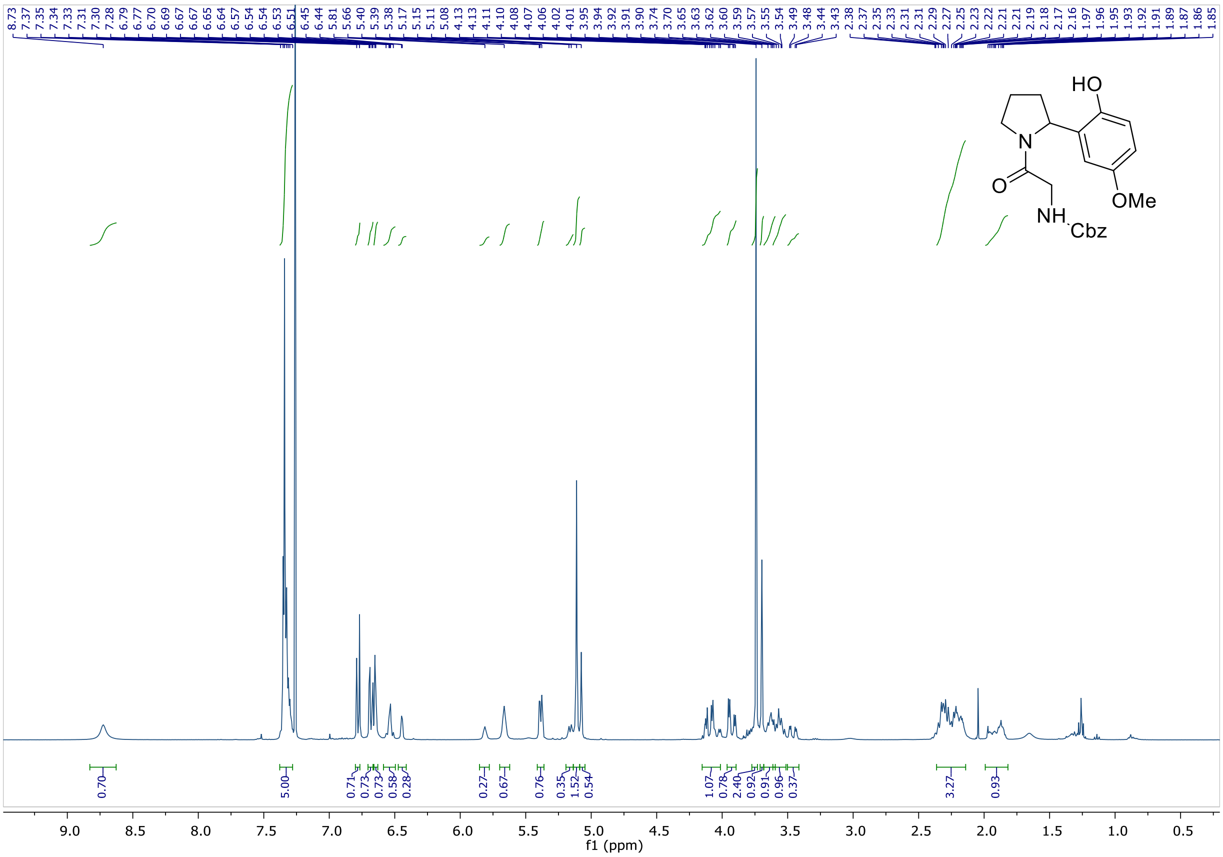

^{13}C-NMR (101 MHz, Chloroform-*d*) (5b)

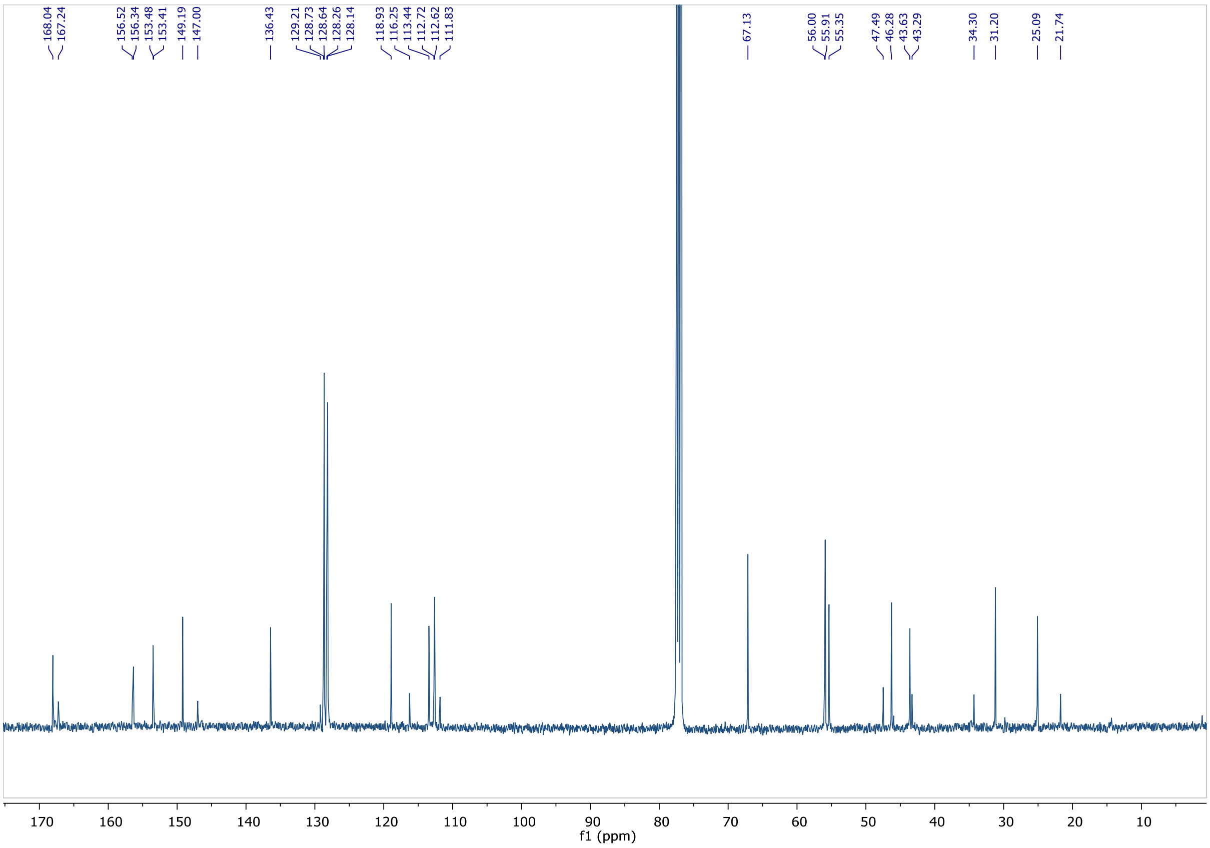

^{1}H-NMR (400 MHz, Acetonitrile-d_3) (5c major)

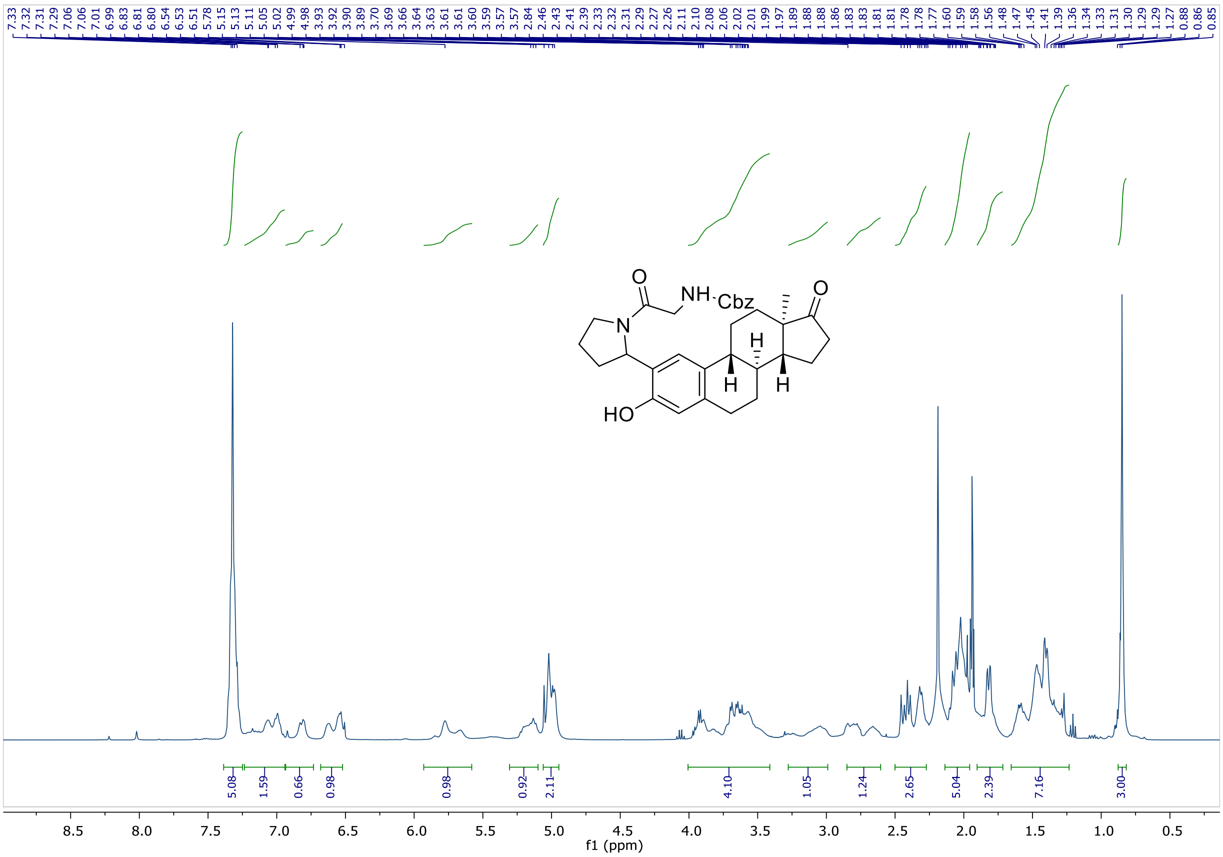

^{13}C-NMR (101 MHz, Acetonitrile-d_3) (5c major)

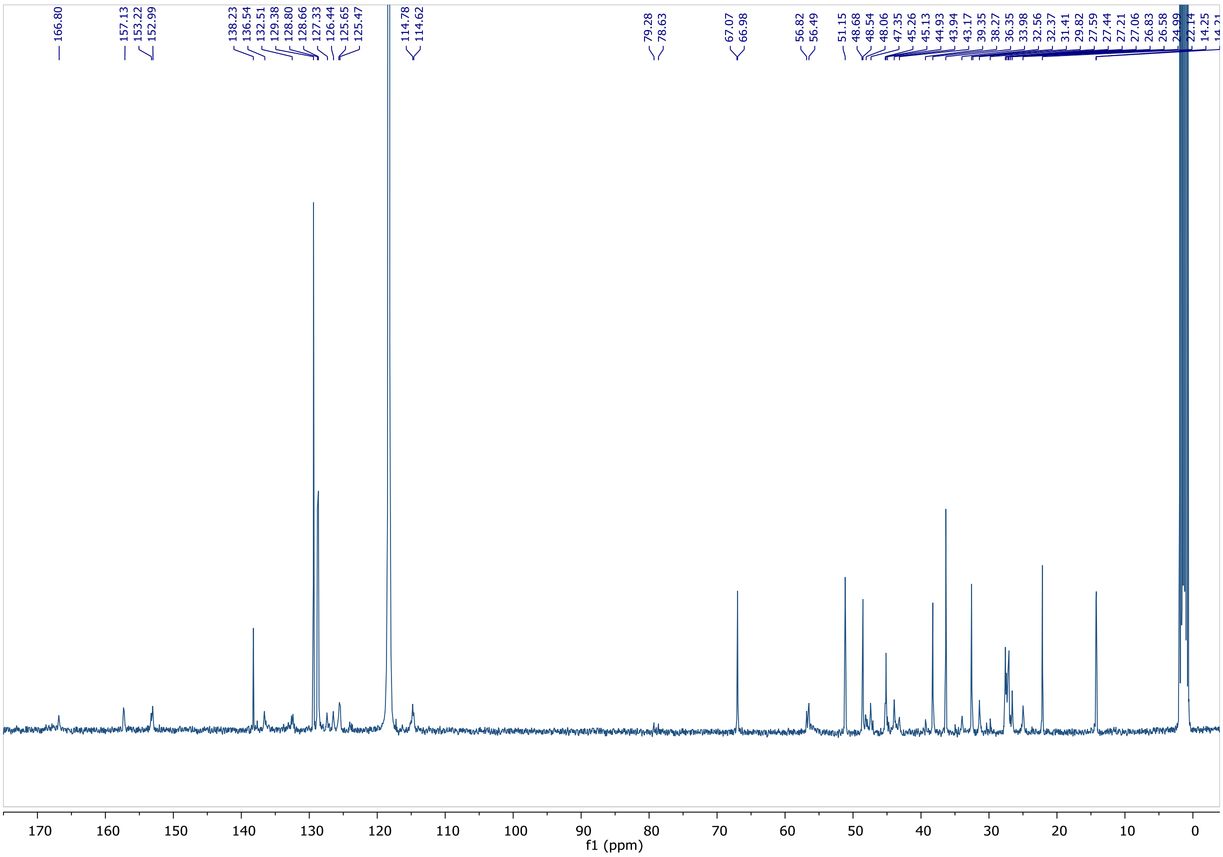

^{1}H-NMR (400 MHz, Acetonitrile-d_3) (5c minor)

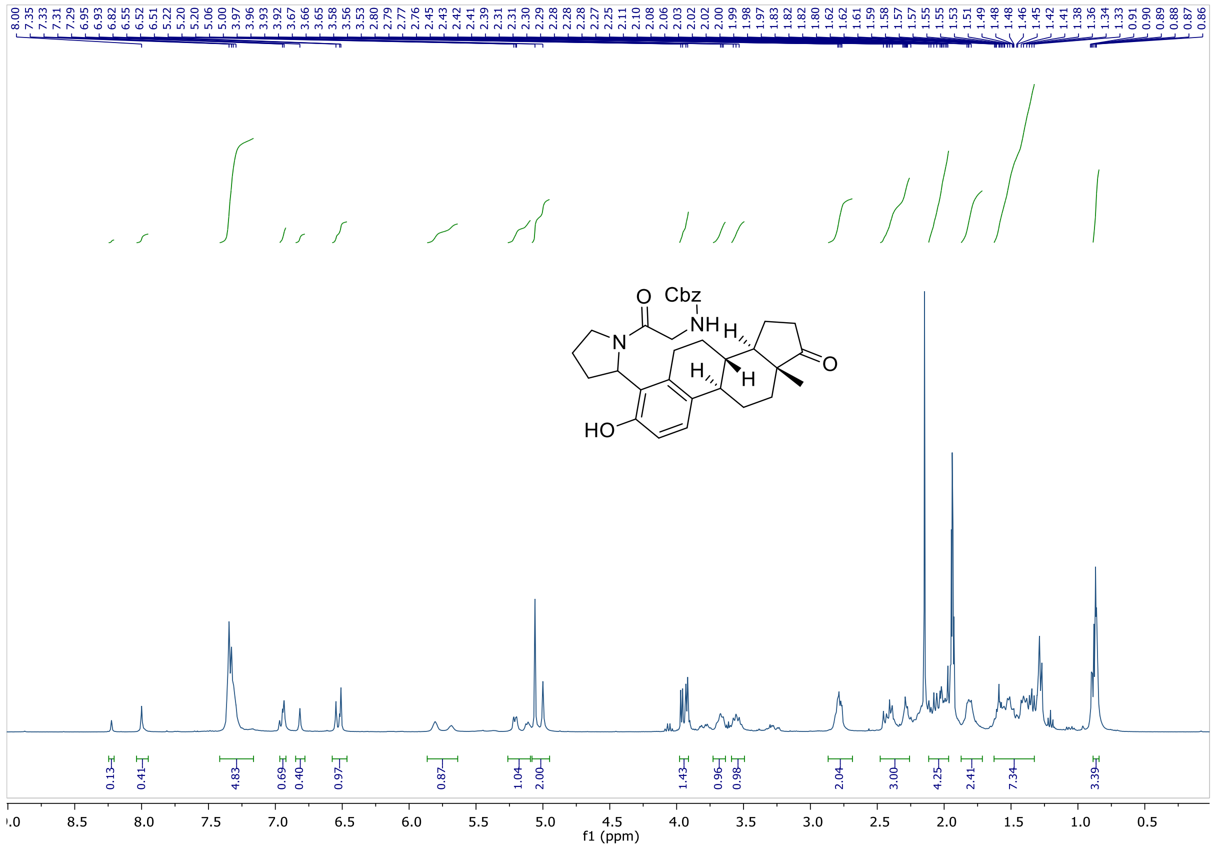

^{13}C-NMR (101 MHz, Acetonitrile-d_3) (5c minor)

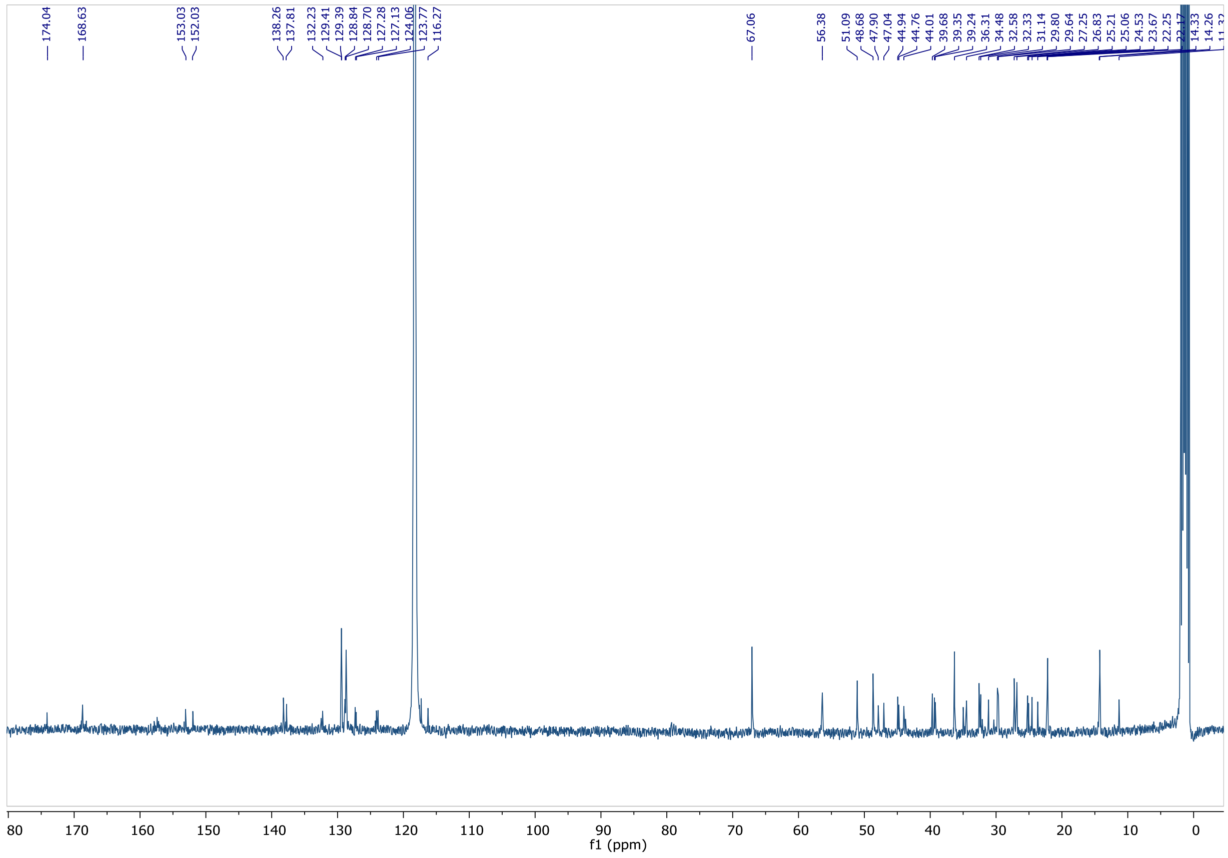

^{1}H-NMR (400 MHz, Chloroform-*d*) (5d)

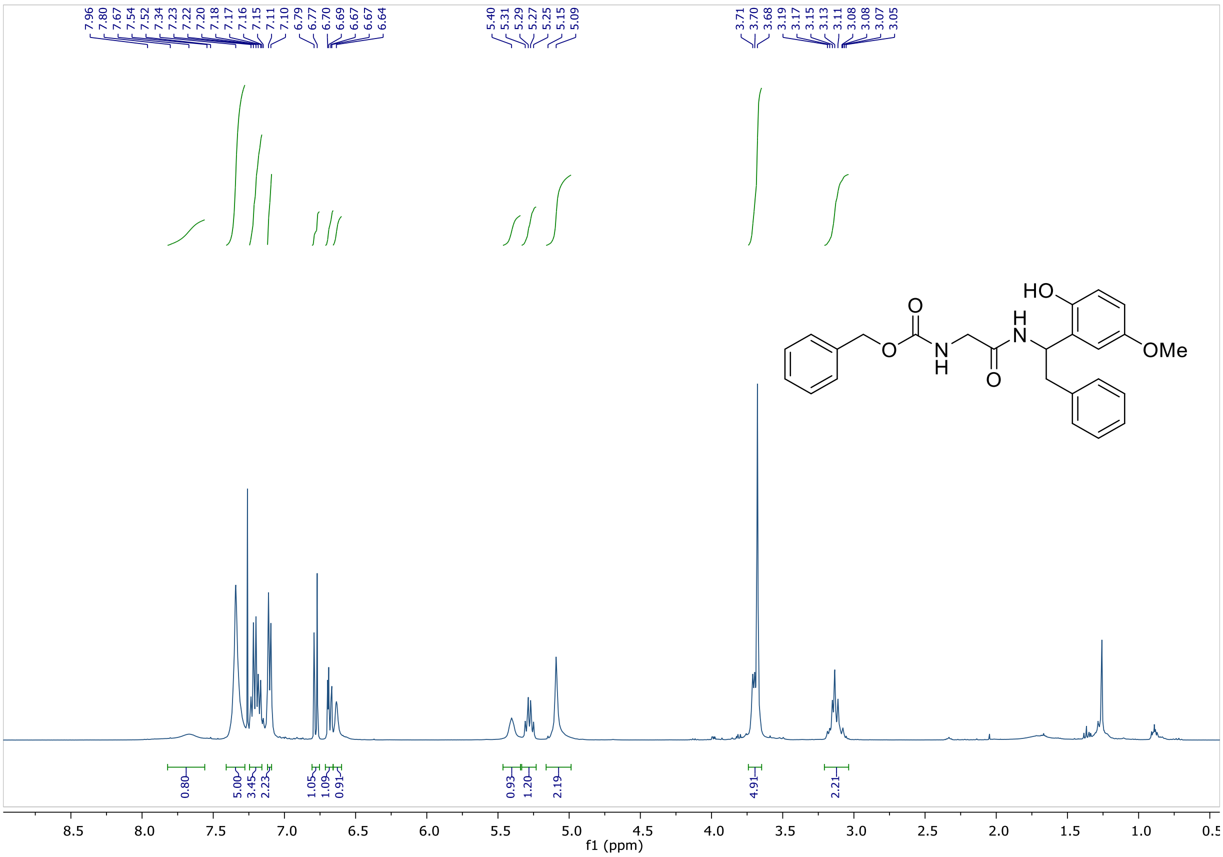

^{13}C-NMR (101 MHz, Chloroform-*d*) (5d)

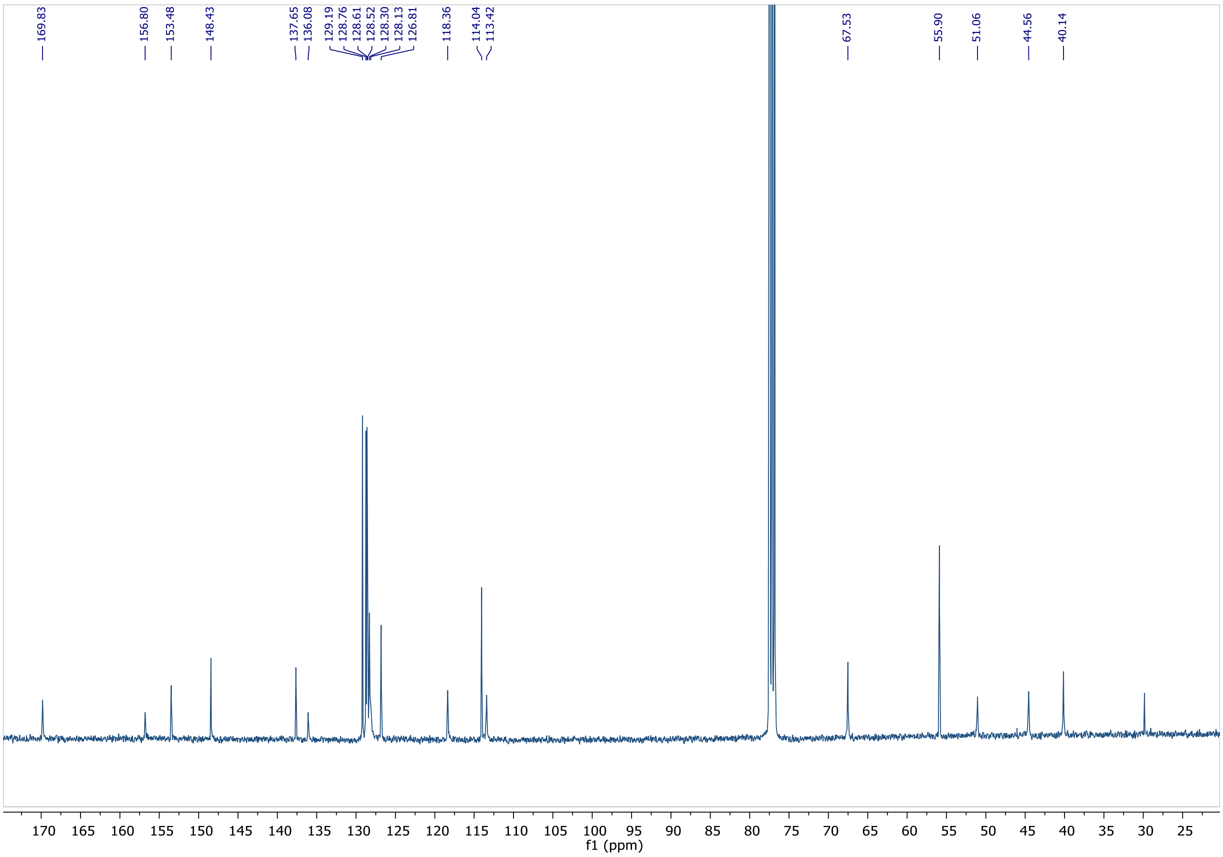

^{1}H-NMR (400 MHz, Chloroform-*d*) (5e)

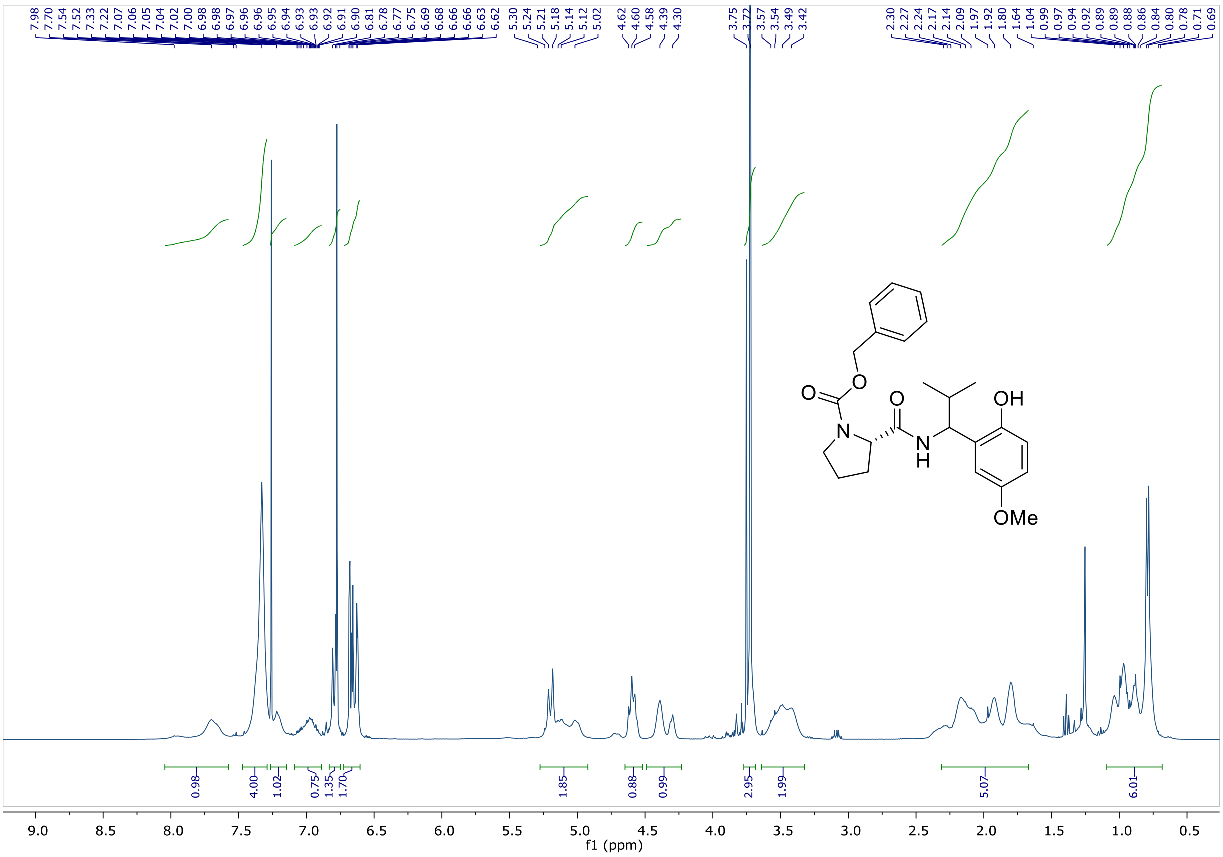

^{13}C-NMR (101 MHz, Chloroform-*d*) (5e)

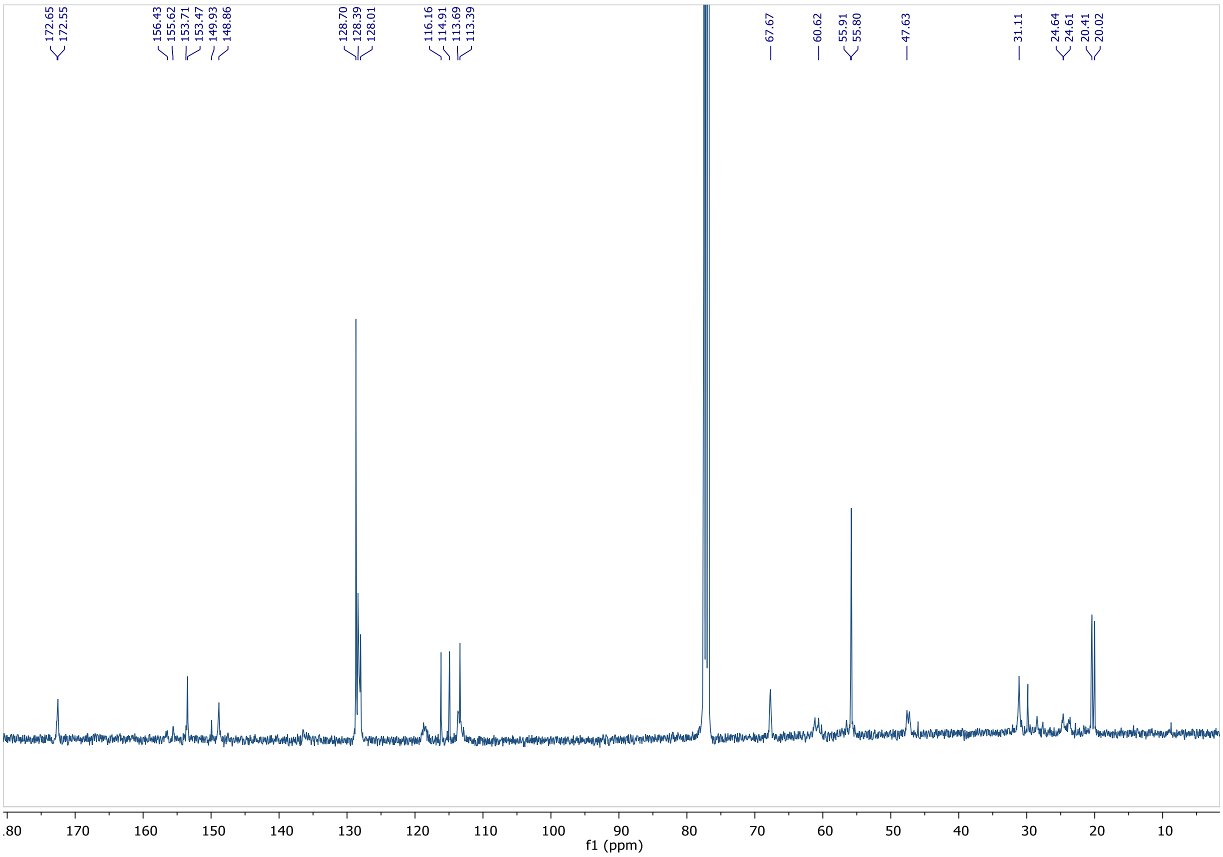

^{1}H-NMR (400 MHz, Chloroform-*d*) (5f)

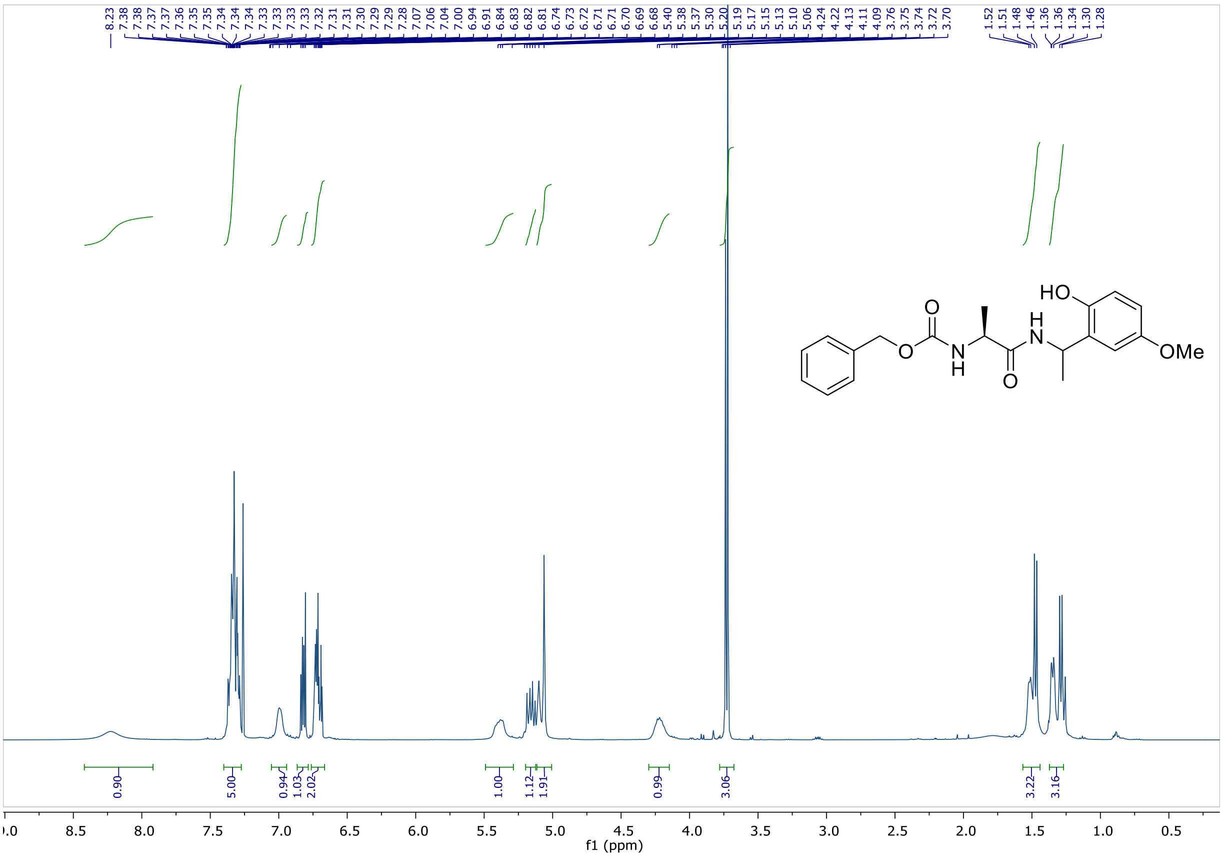

^{13}C-NMR (101 MHz, Chloroform-*d*) (5f)

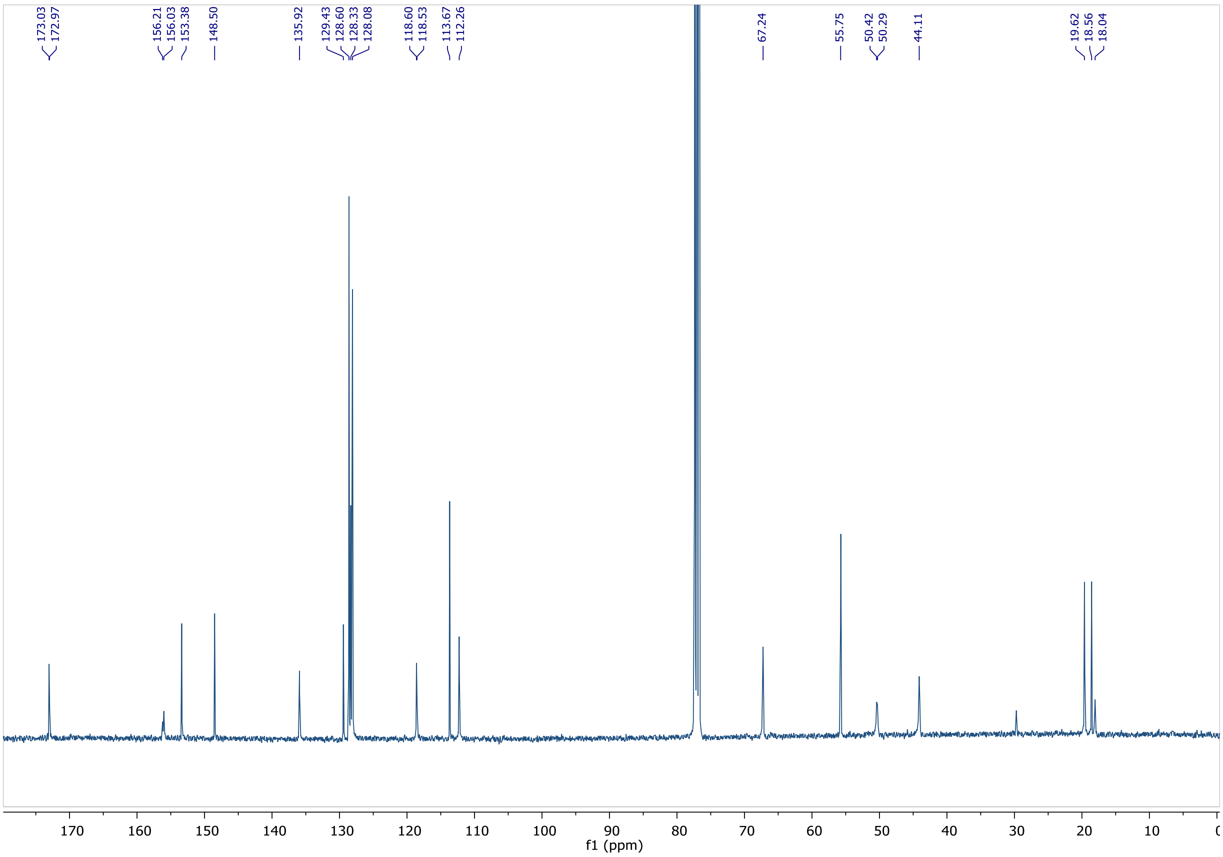

^{1}H-NMR (400 MHz, Chloroform-*d*) (5g)

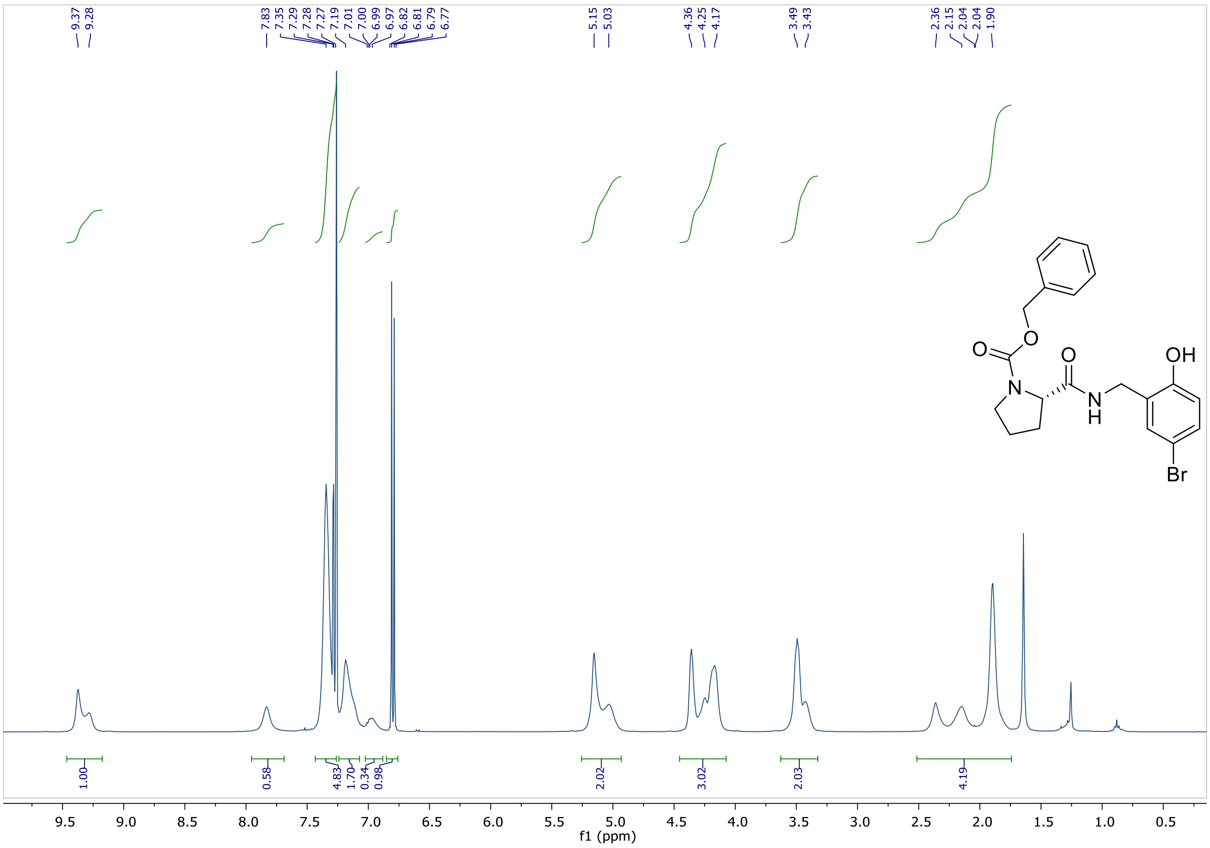

^{13}C-NMR (101 MHz, Chloroform-*d*) (5g)

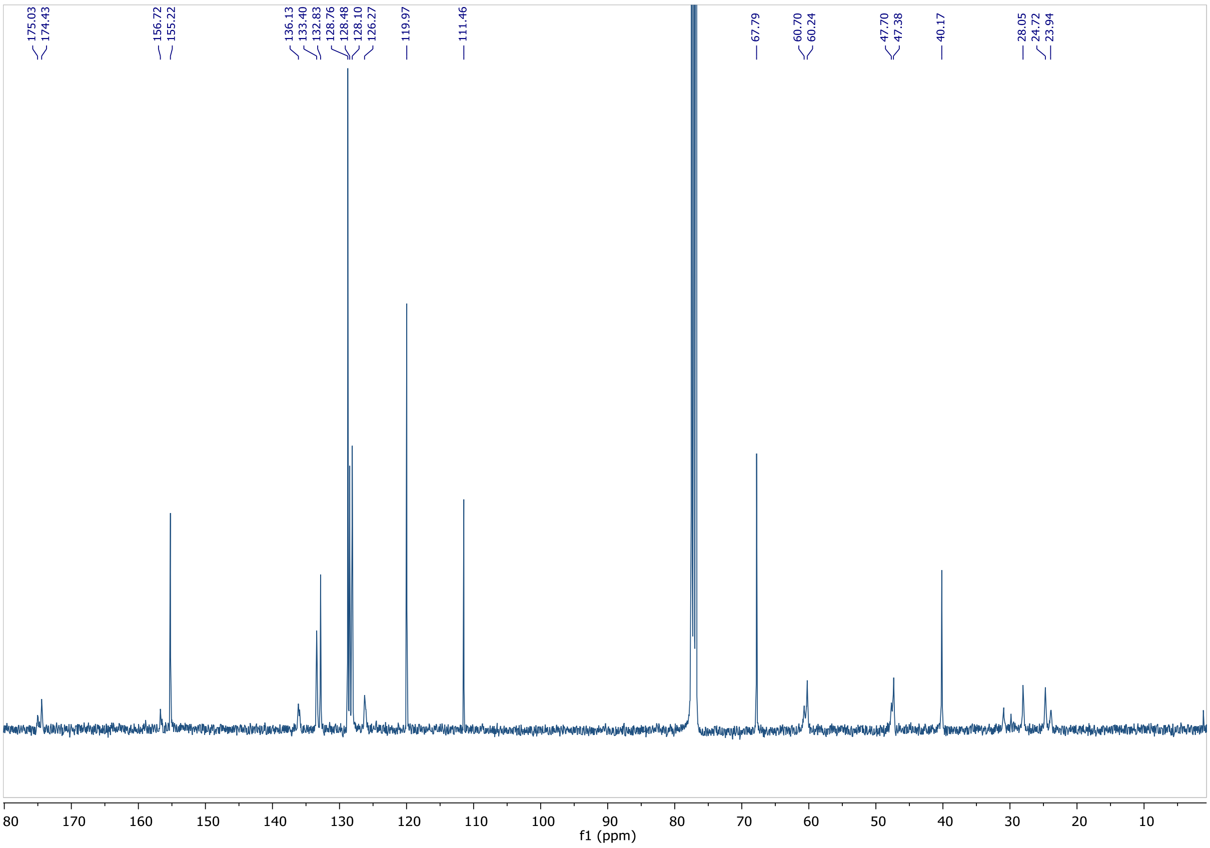

^{1}H-NMR (400 MHz, Chloroform-*d*) (5h)

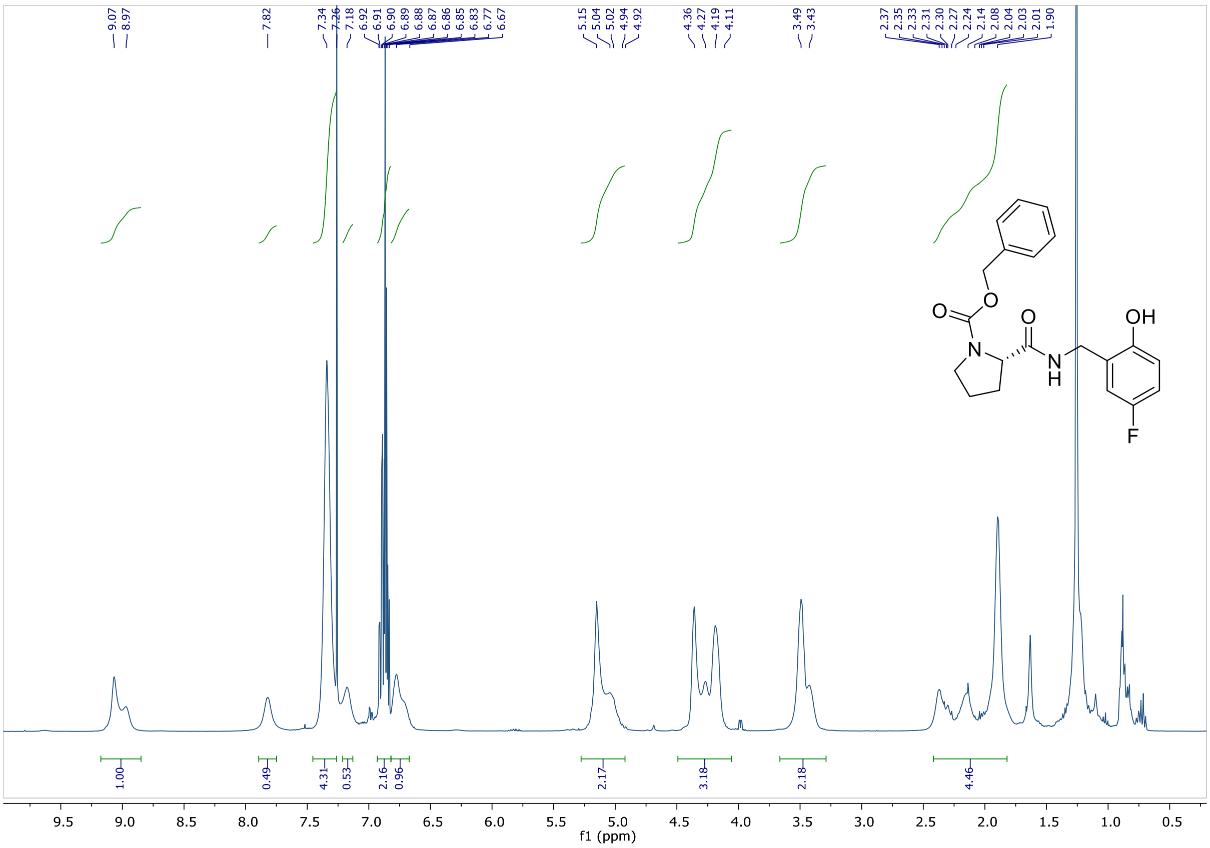

^{13}C-NMR (101 MHz, Chloroform-*d*) (5h)

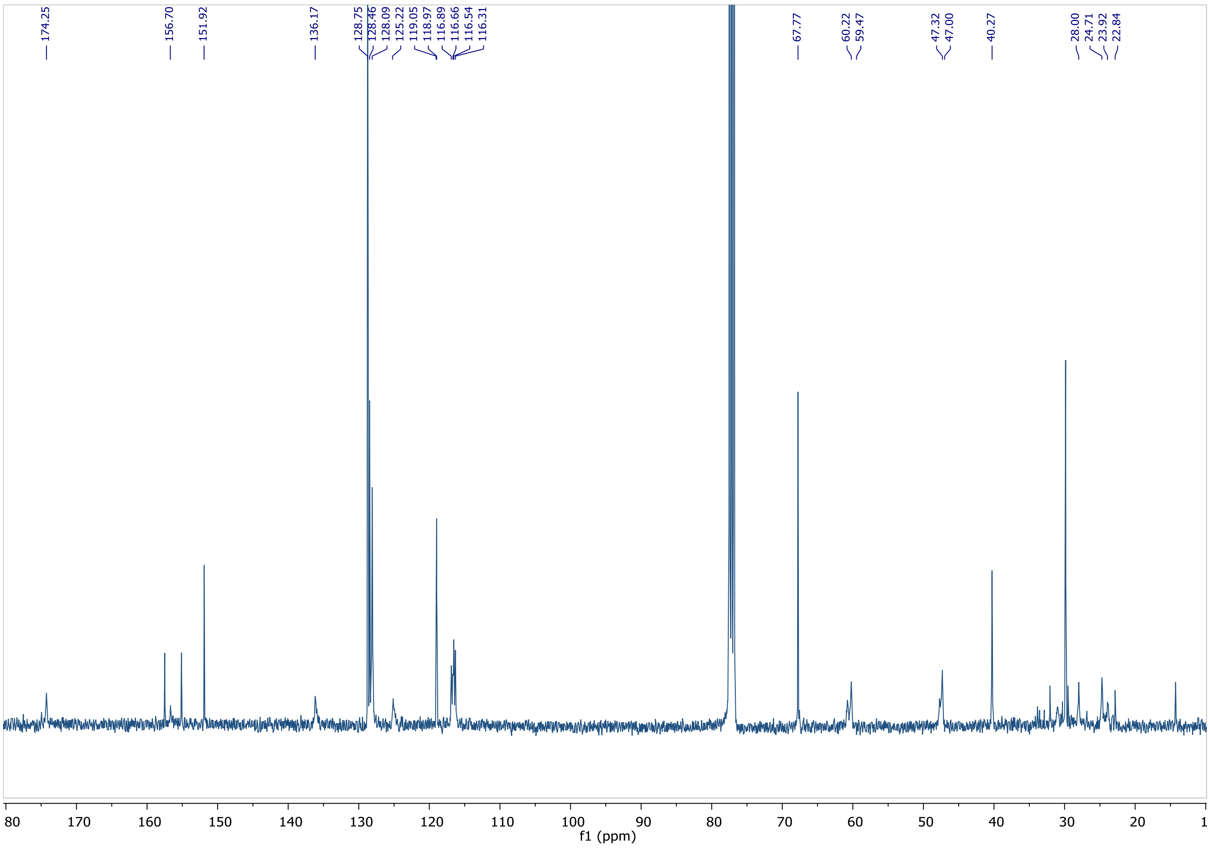

^{19}F-NMR (376 MHz, Chloroform-*d*) (5h)

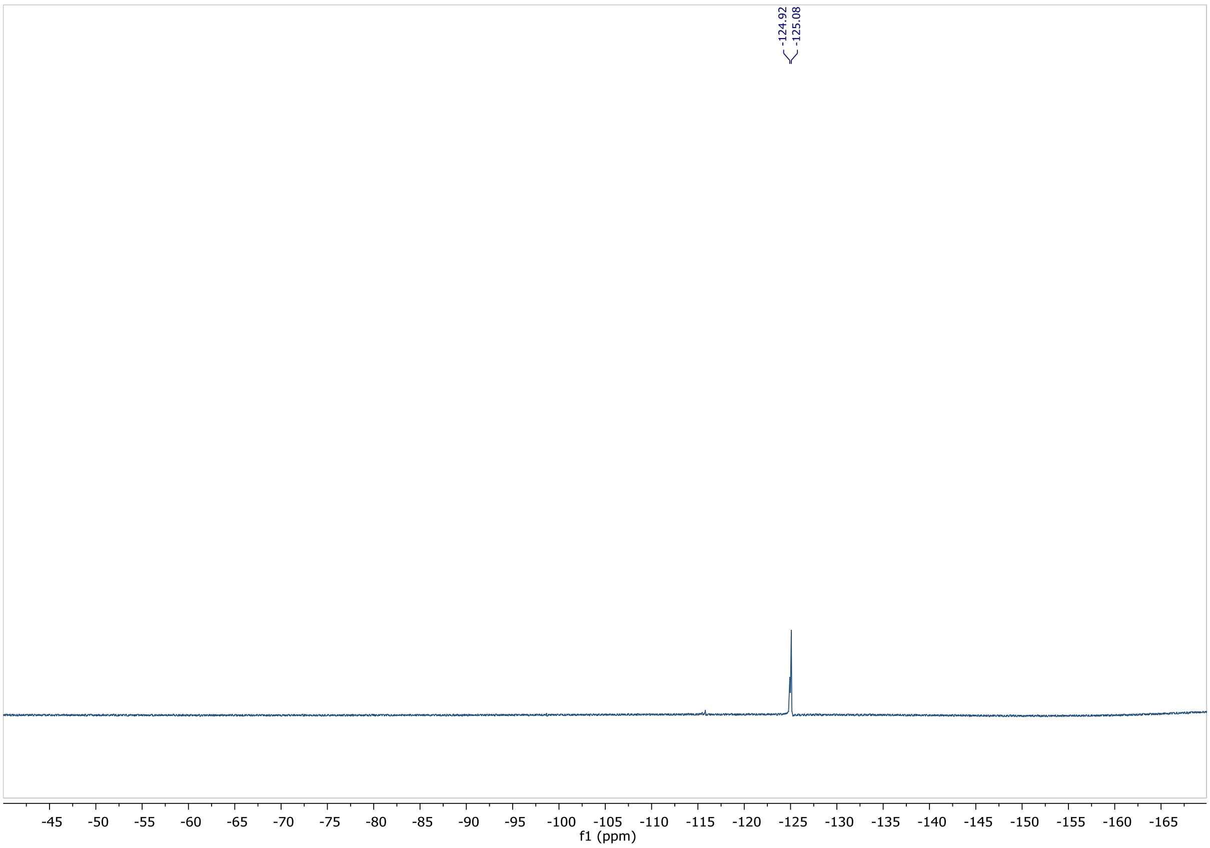

^{1}H-NMR (400 MHz, Chloroform-*d*) (5i)

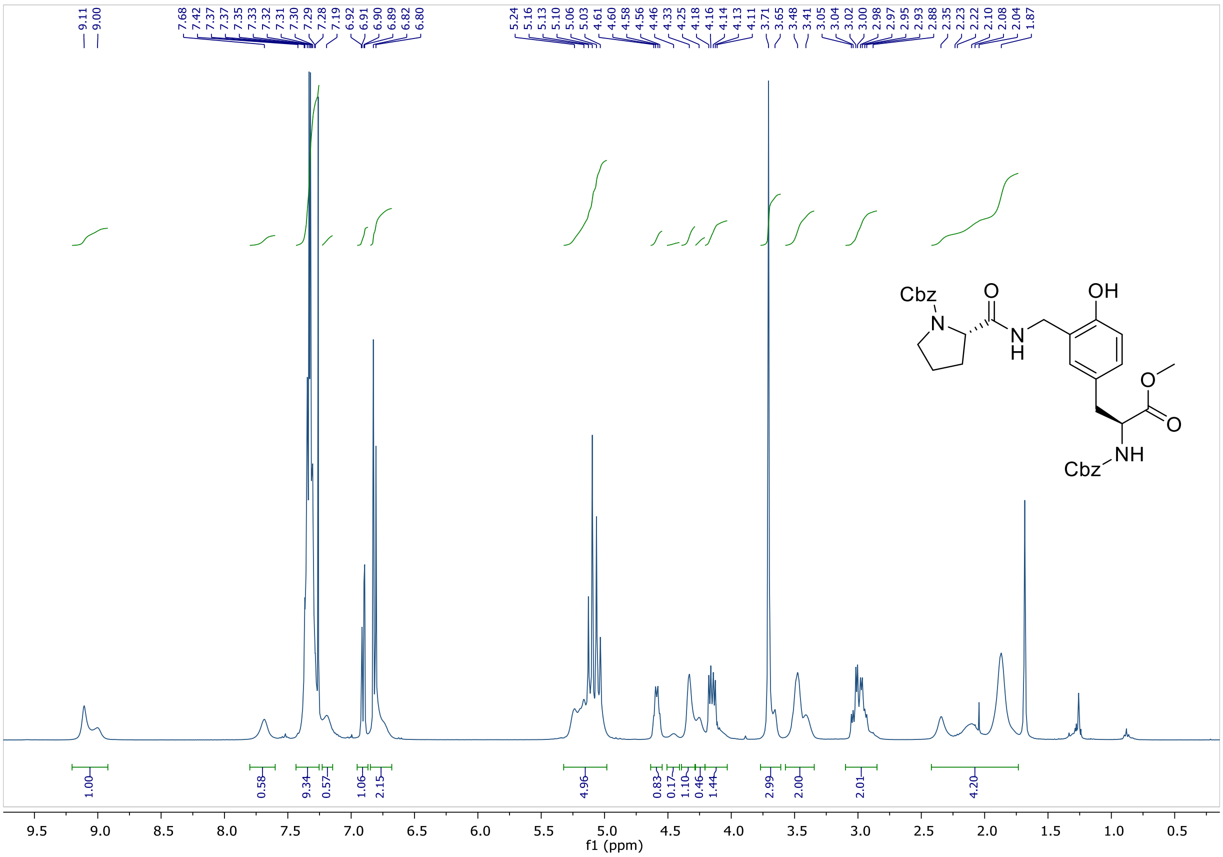

^{13}C-NMR (101 MHz, Chloroform-*d*) (5i)

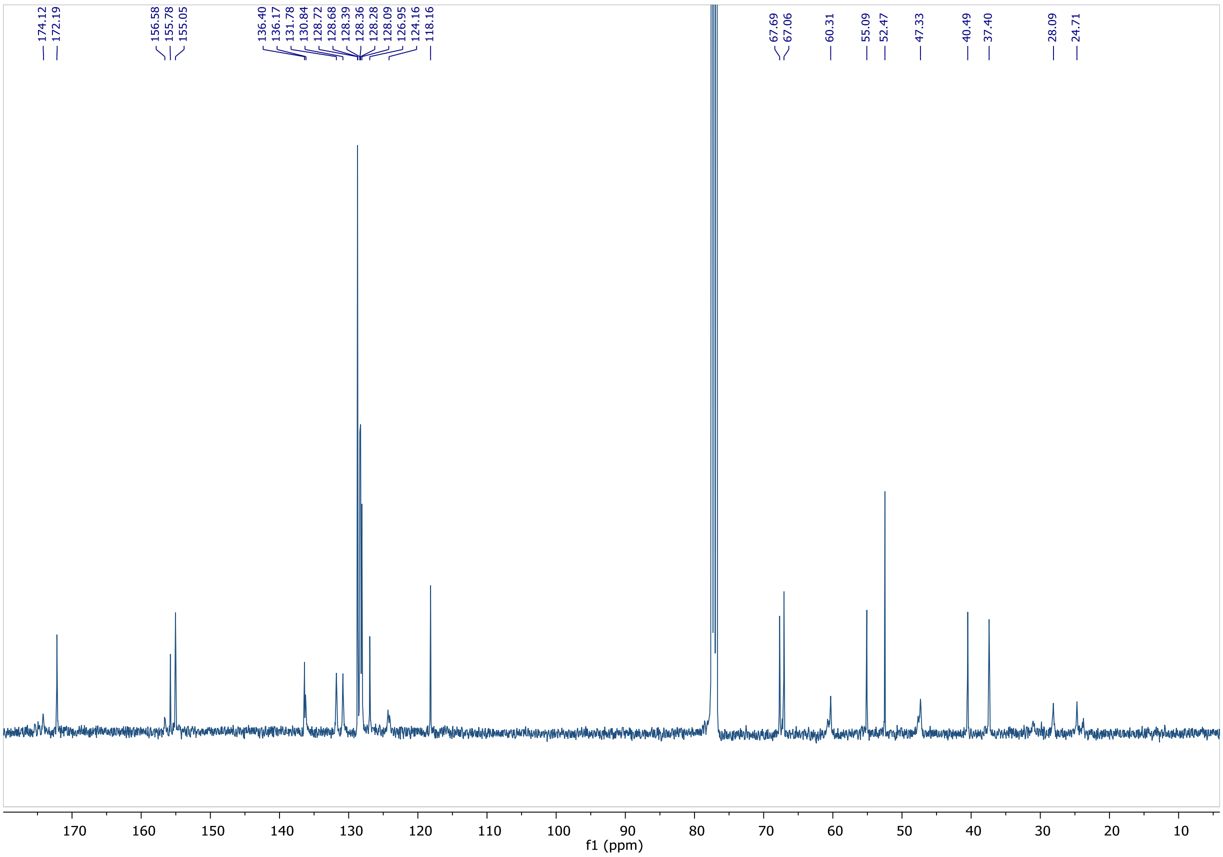

^{1}H-NMR (400 MHz, Acetonitrile-d_3) (5j)

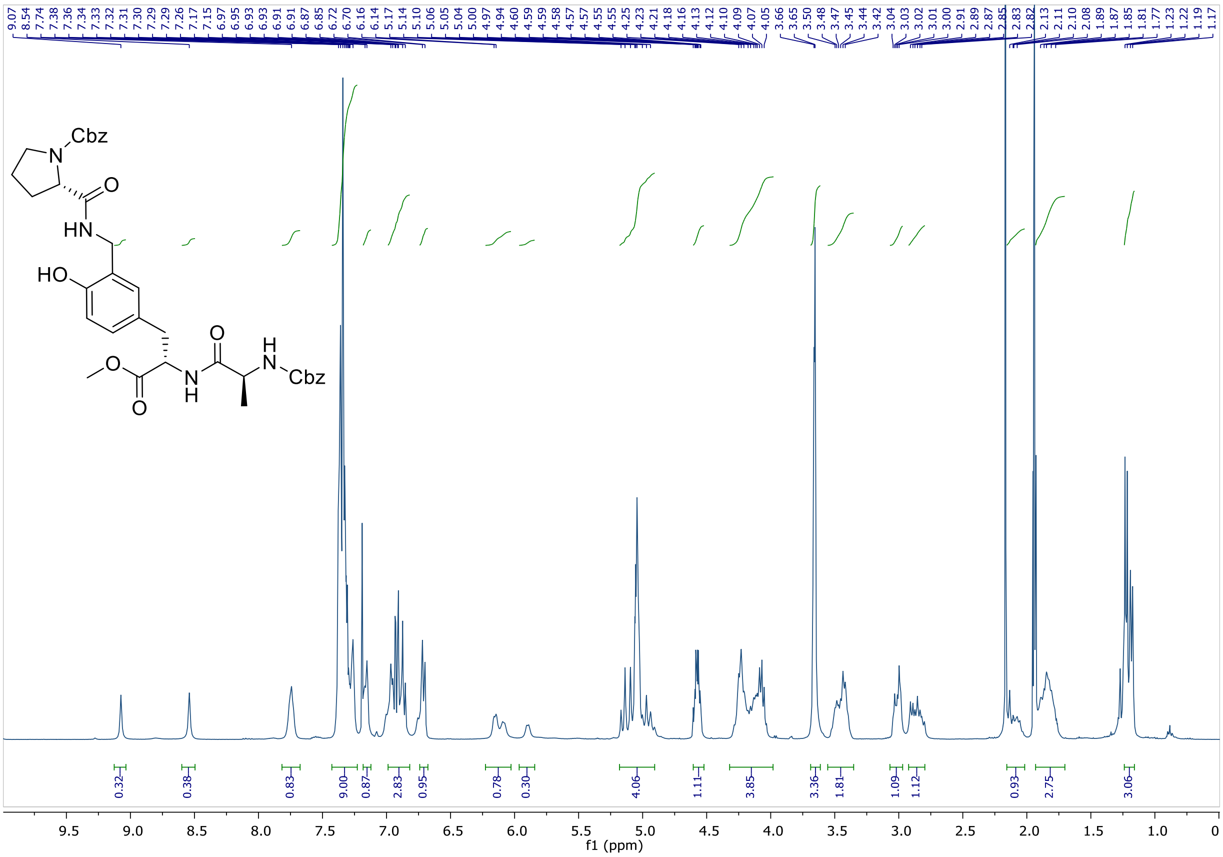

^{13}C-NMR (101 MHz, Acetonitrile-d_3) (5j)

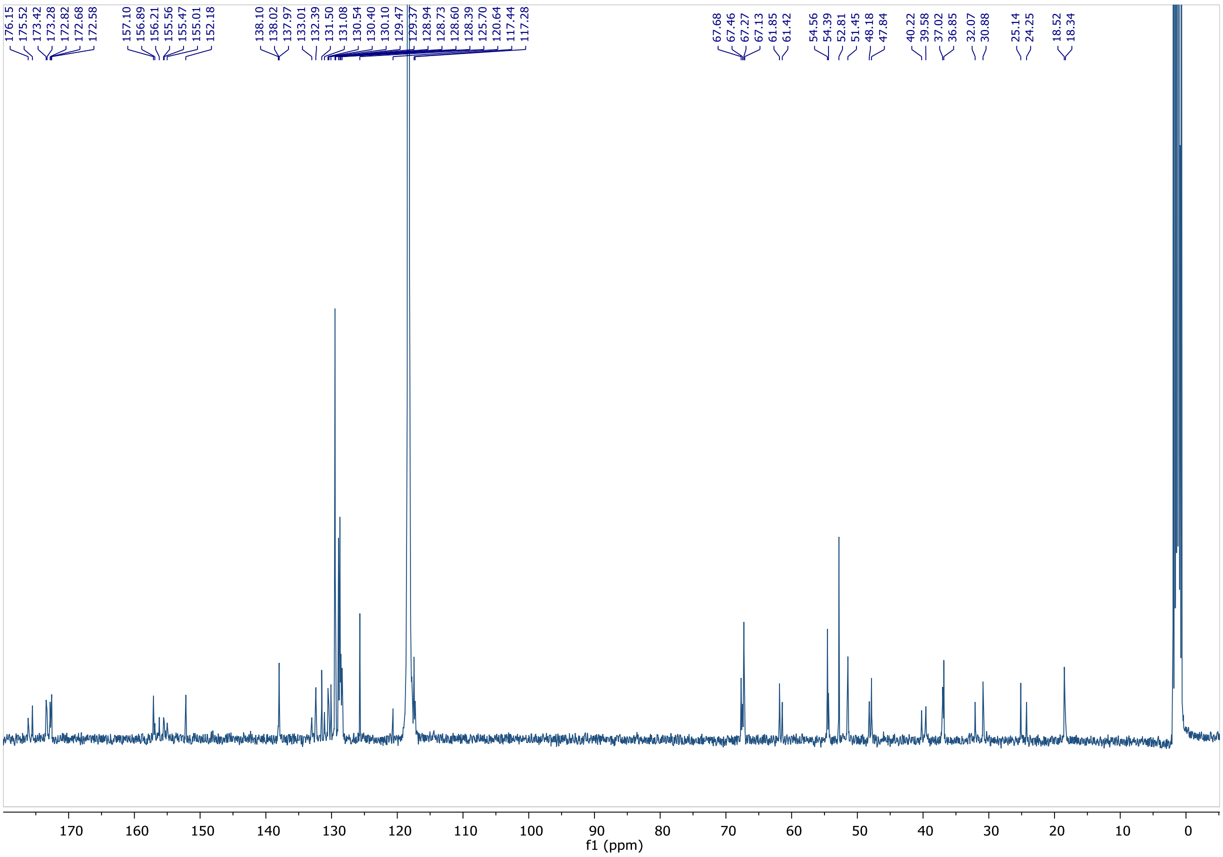

^{1}H-NMR (400 MHz, Chloroform-*d*) (6a)

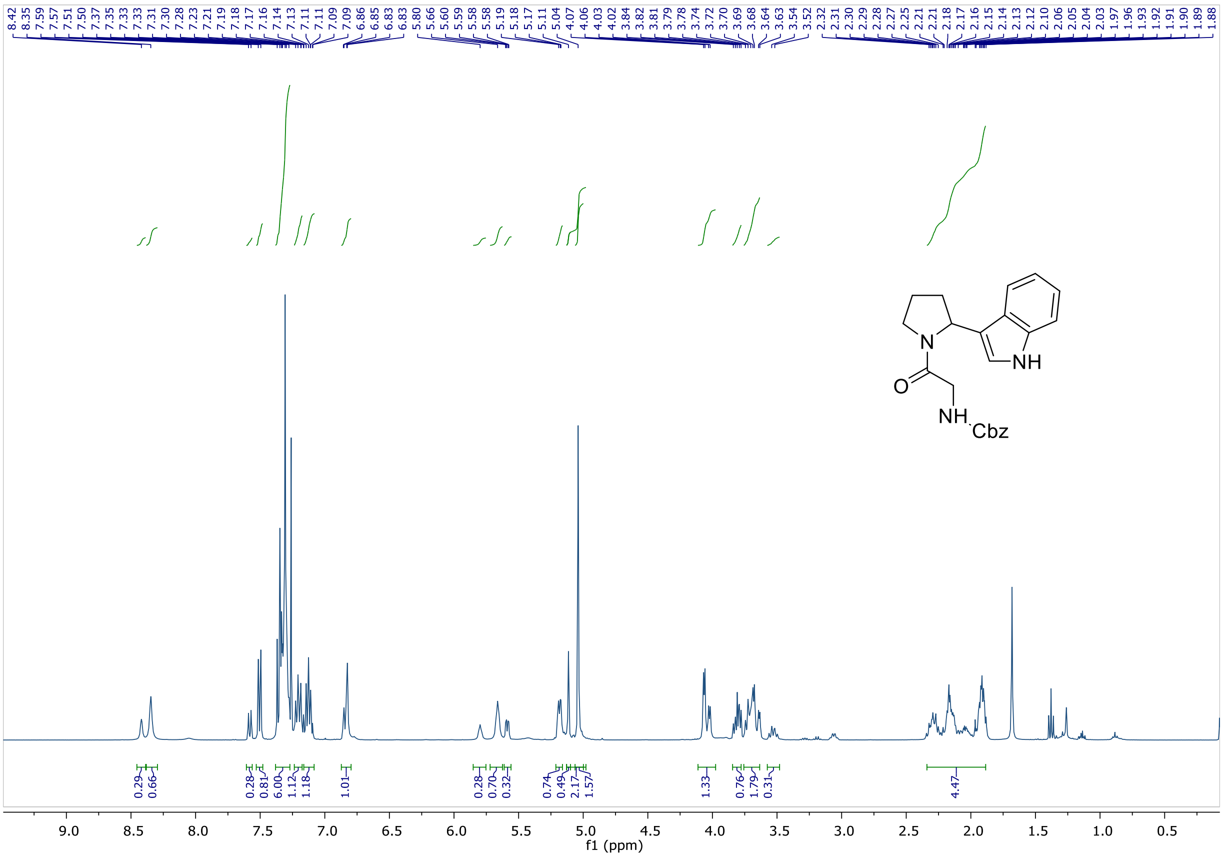

^{13}C-NMR (101 MHz, Chloroform-*d*) (6a)

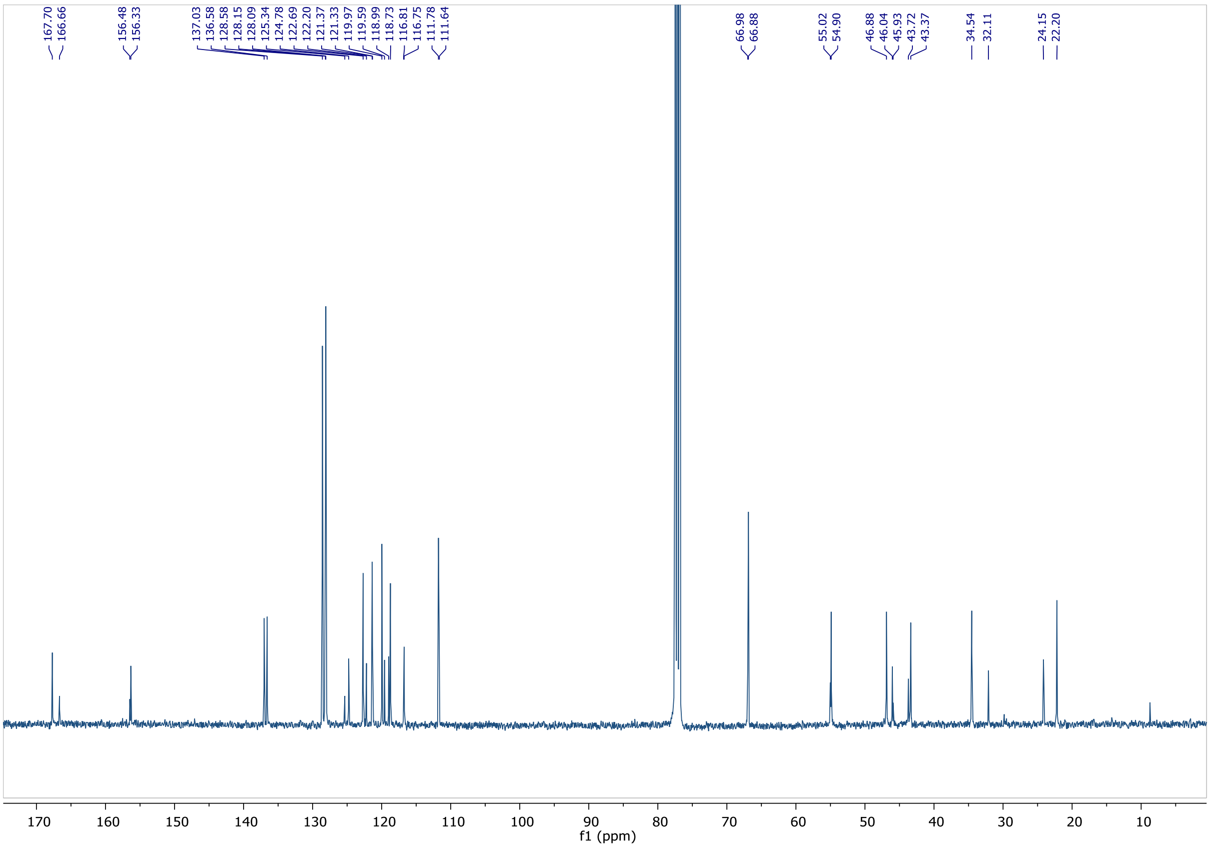

^{1}H-NMR (400 MHz, Chloroform-*d*) (6b)

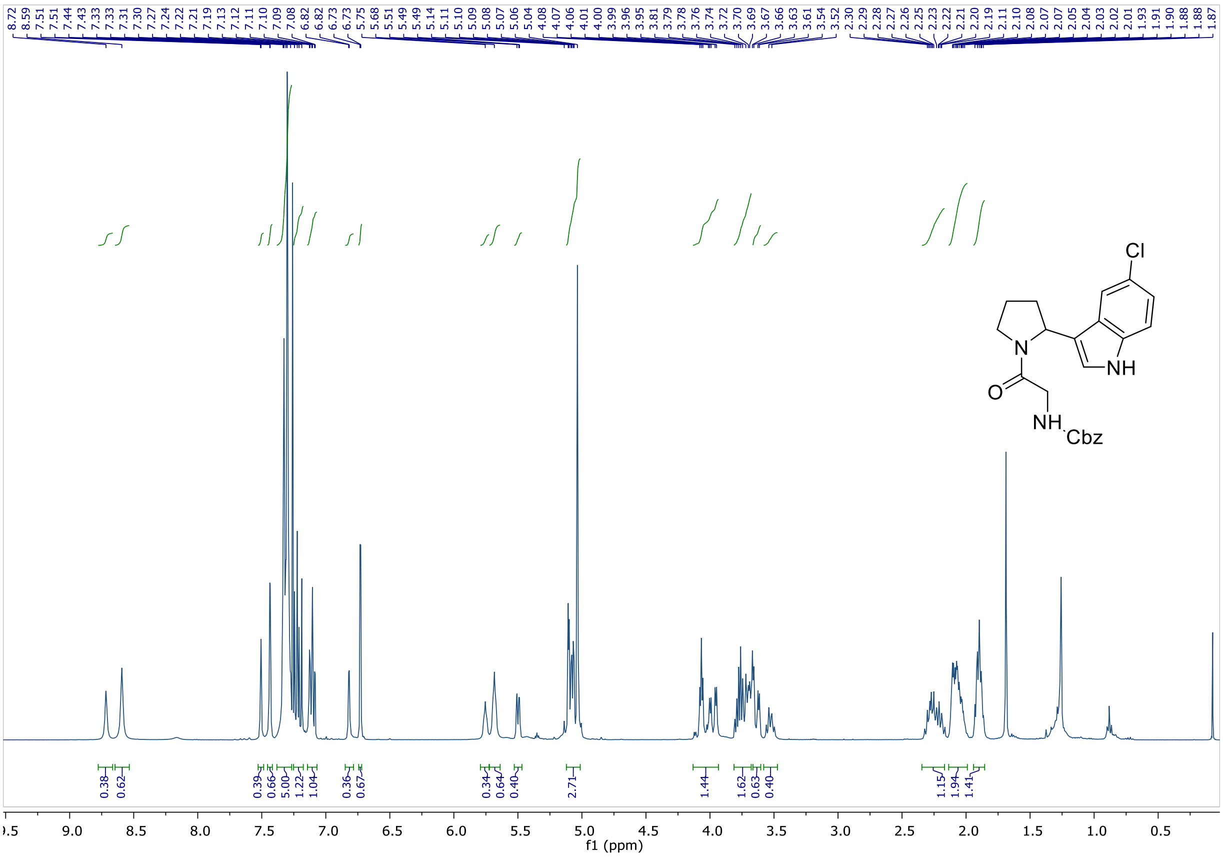

^{13}C-NMR (101 MHz, Chloroform-*d*) (6b)

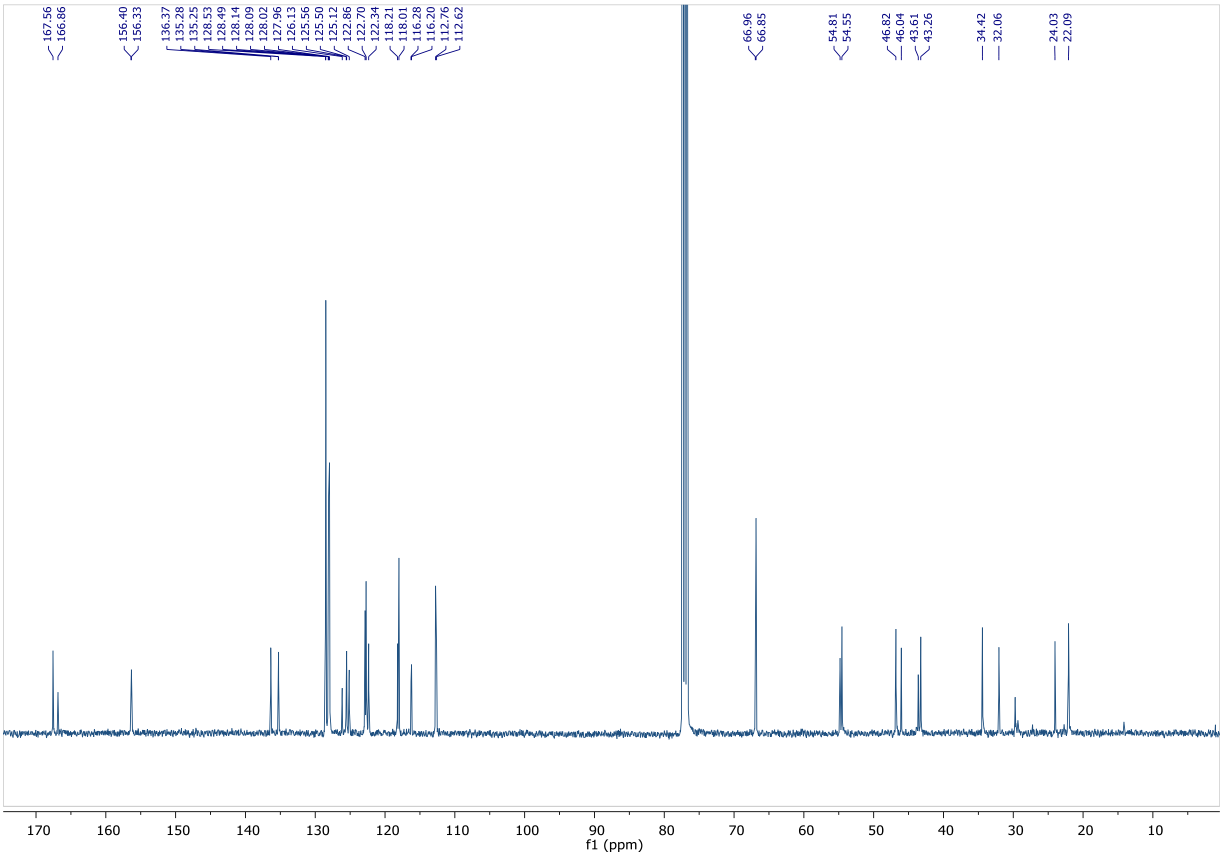

^{1}H-NMR (400 MHz, Acetonitrile-d_3) (6c)

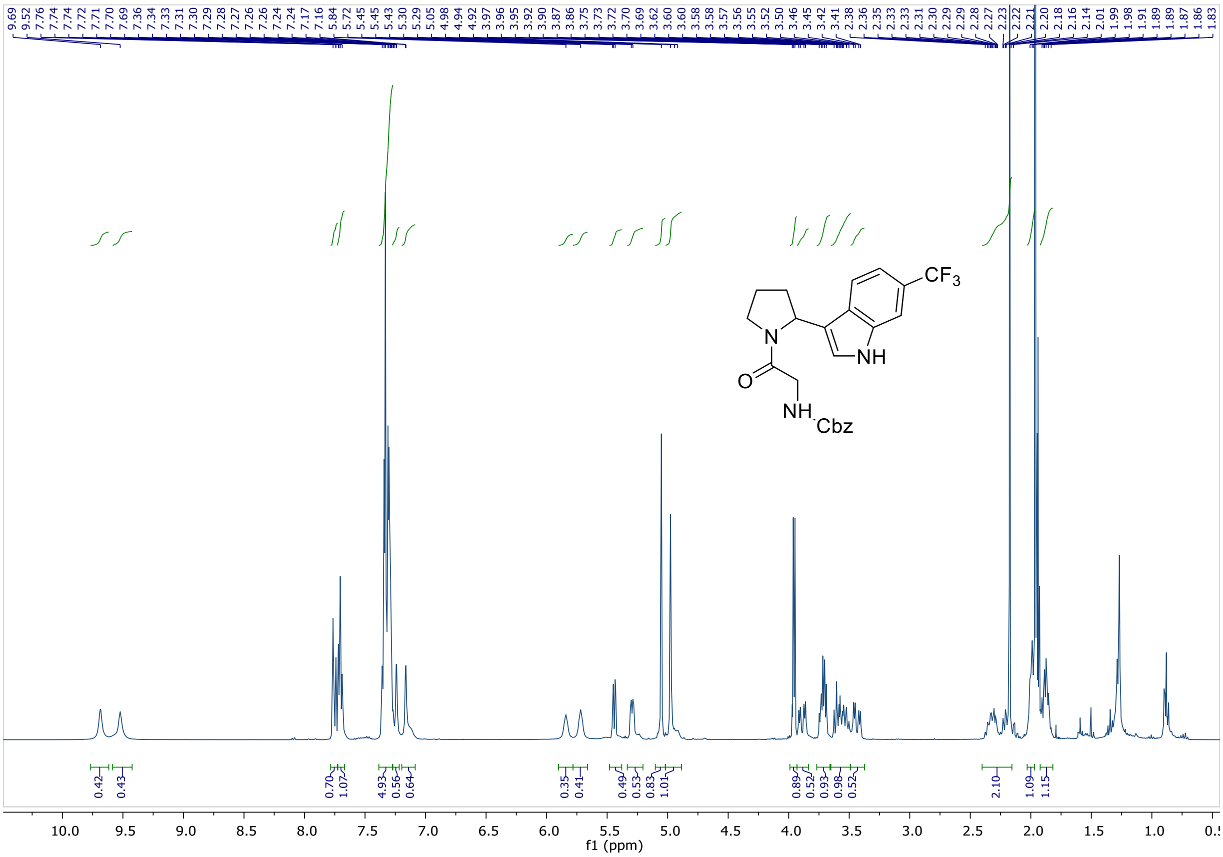

^{13}C-NMR (101 MHz, Acetonitrile-d_3) (6c)

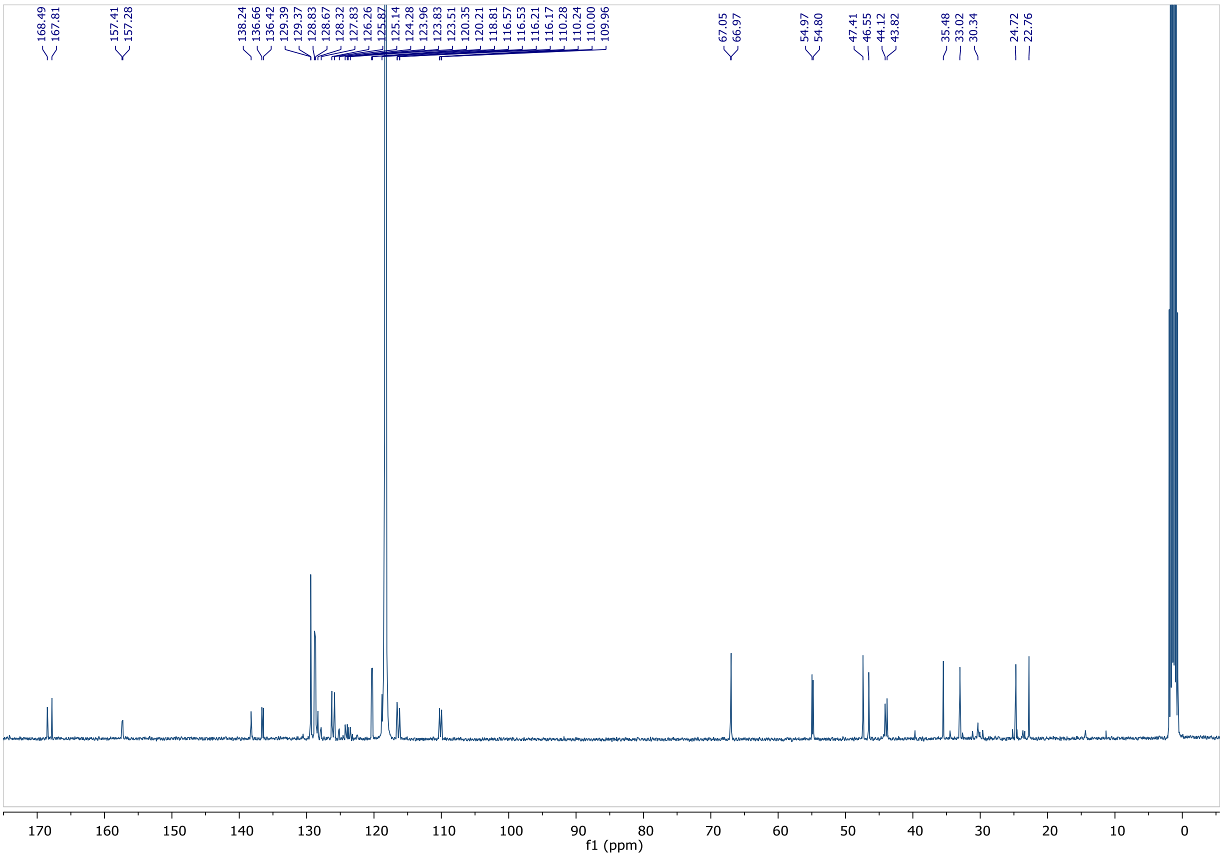

^{19}F-NMR (376 MHz, Acetonitrile-d_3) (6c)

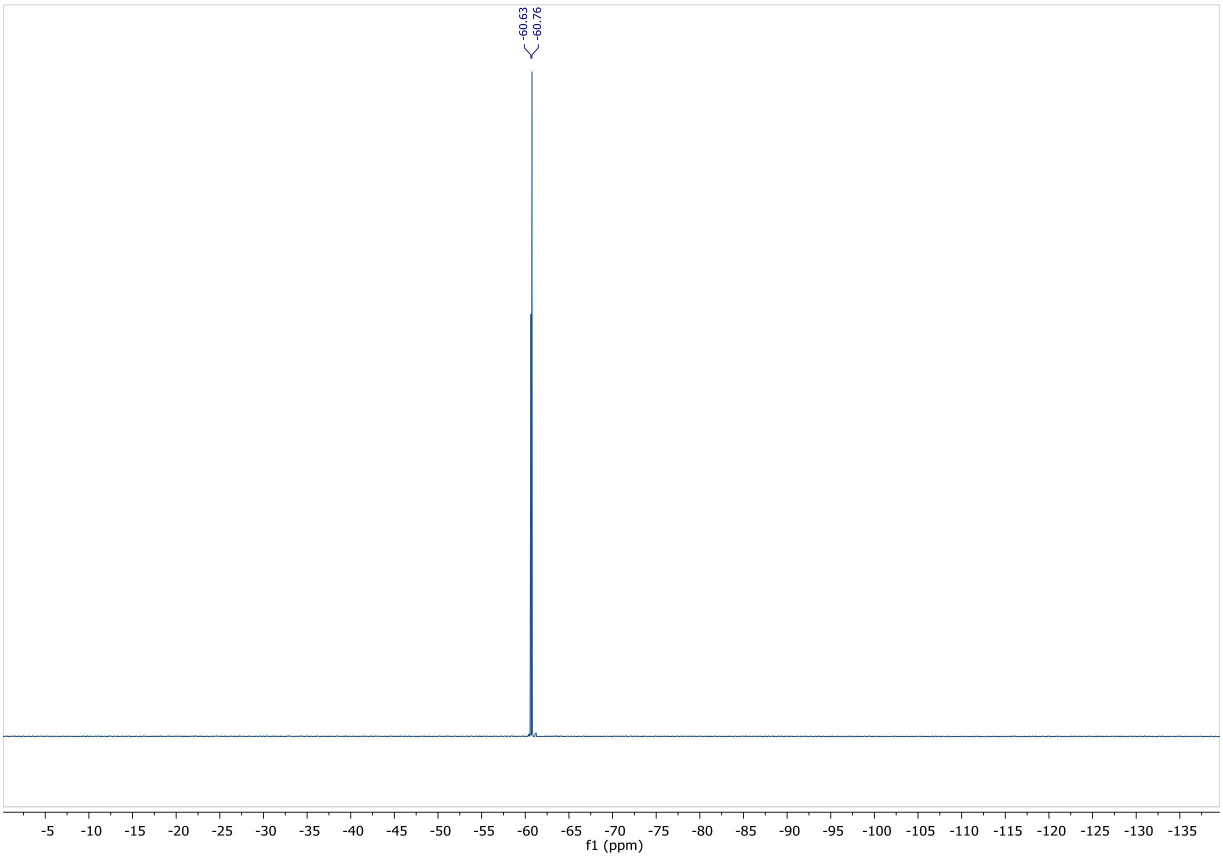

^{1}H-NMR (400 MHz, Chloroform-*d*) (6d)

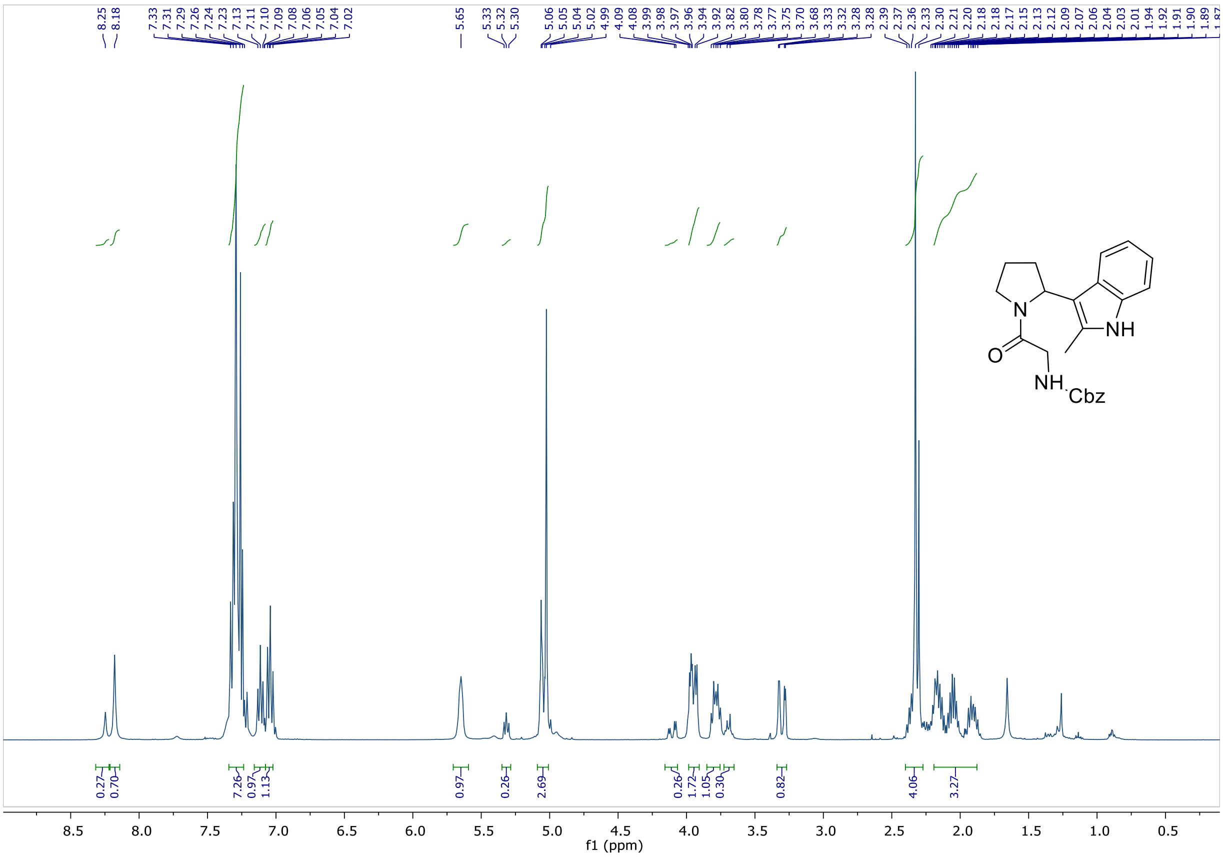

^{13}C-NMR (101 MHz, Chloroform-*d*) (6d)

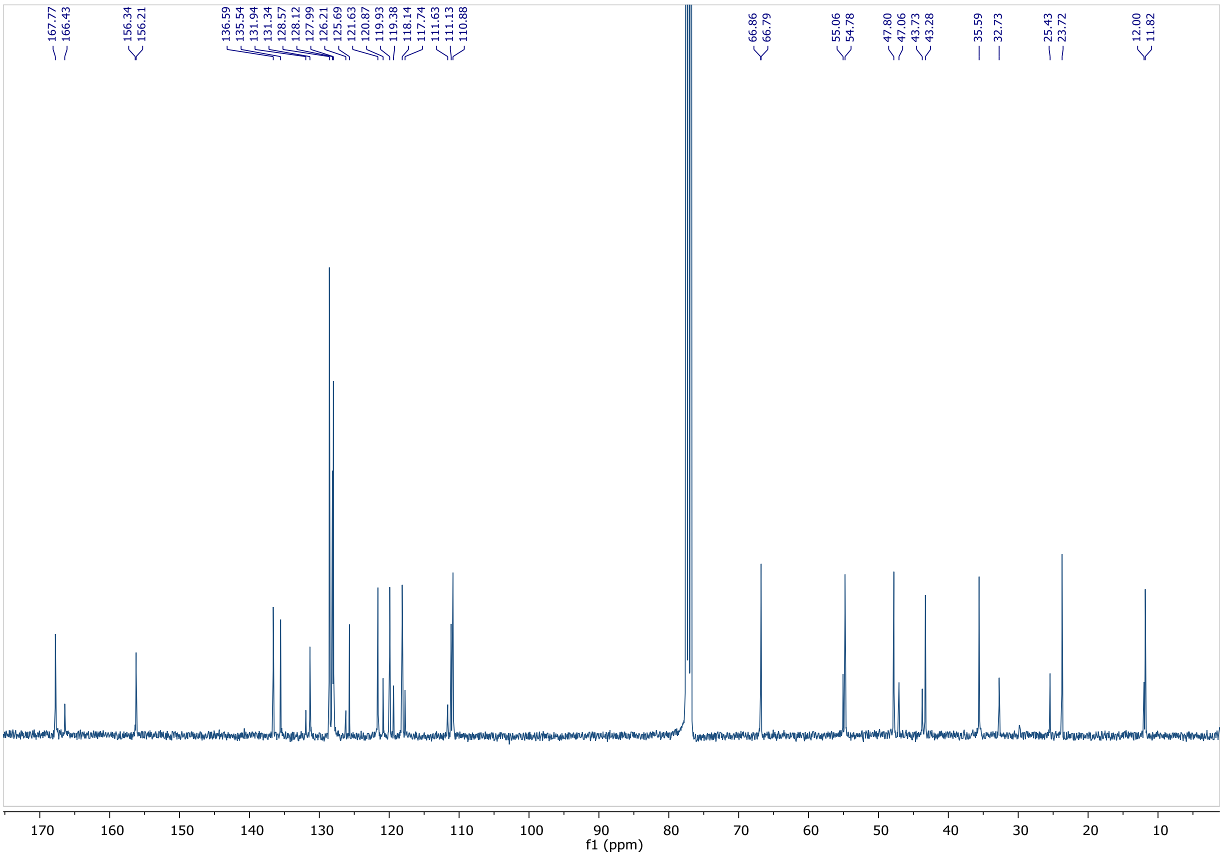

^{1}H-NMR (400 MHz, Chloroform-*d*) (6e)

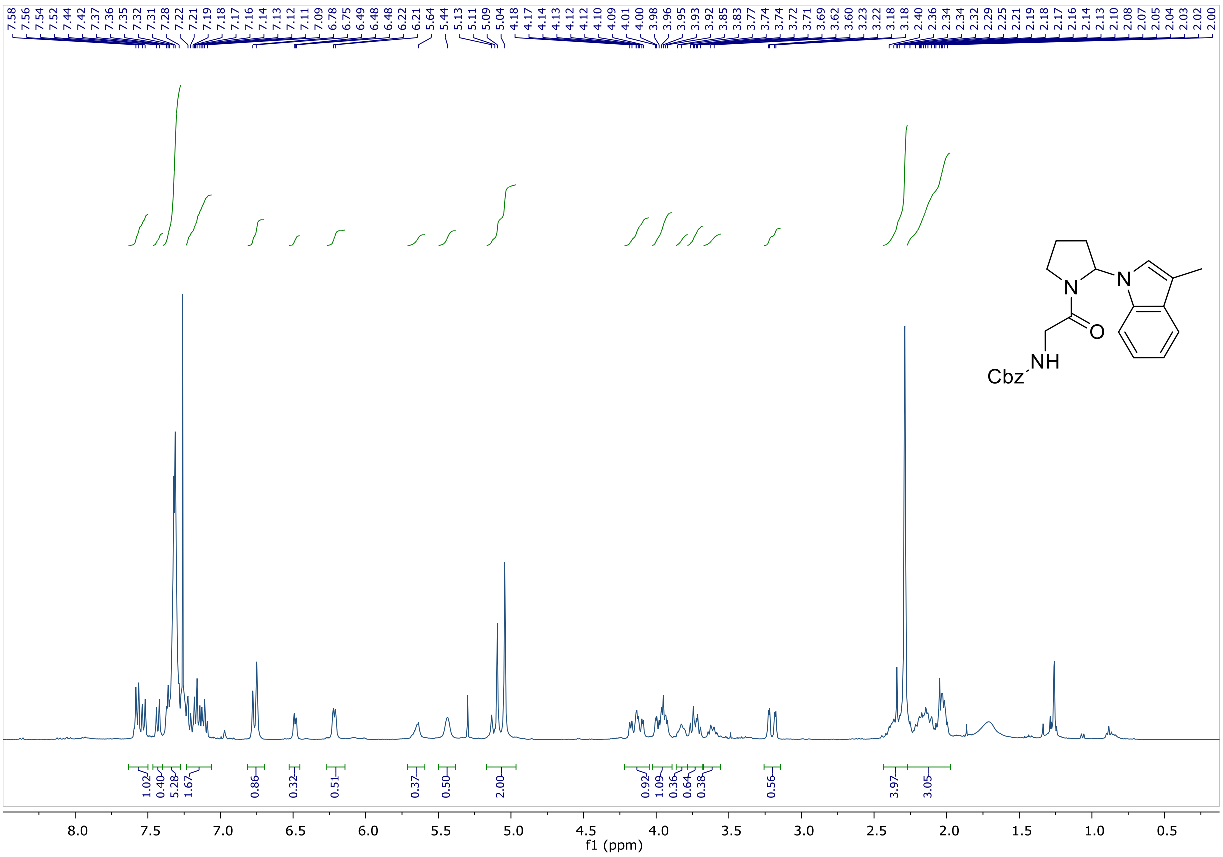

^{13}C-NMR (101 MHz, Chloroform-*d*) (6e)

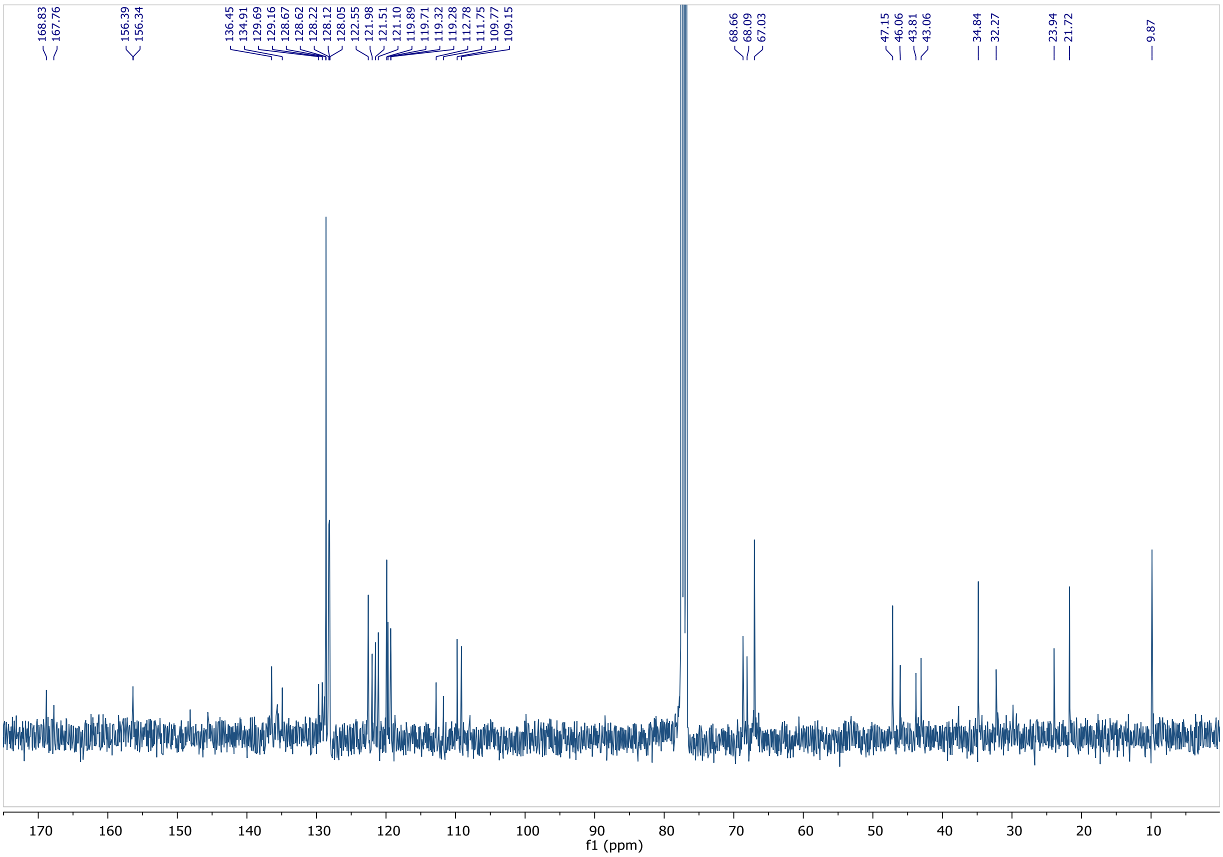

^{1}H-NMR (400 MHz, Chloroform-*d*) (6f)

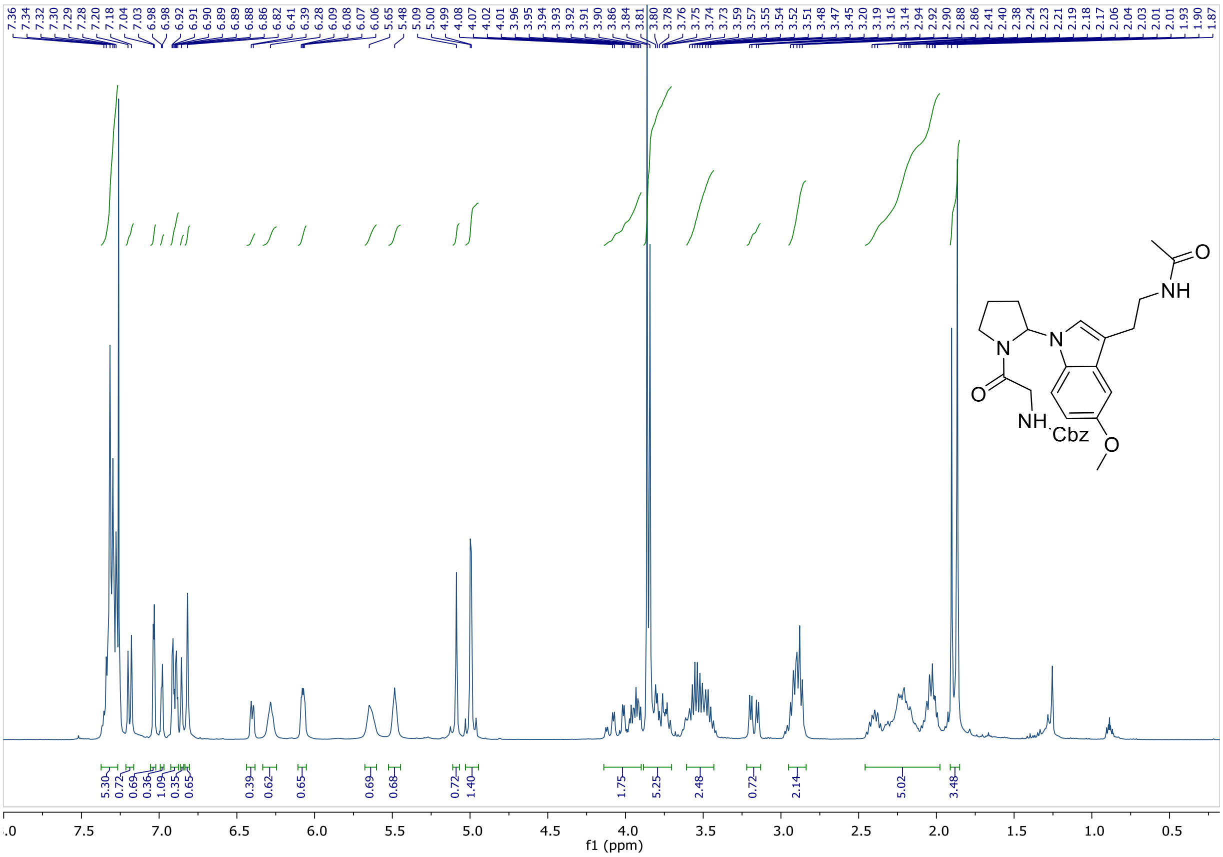

^{13}C-NMR (101 MHz, Chloroform-*d*) (6f)

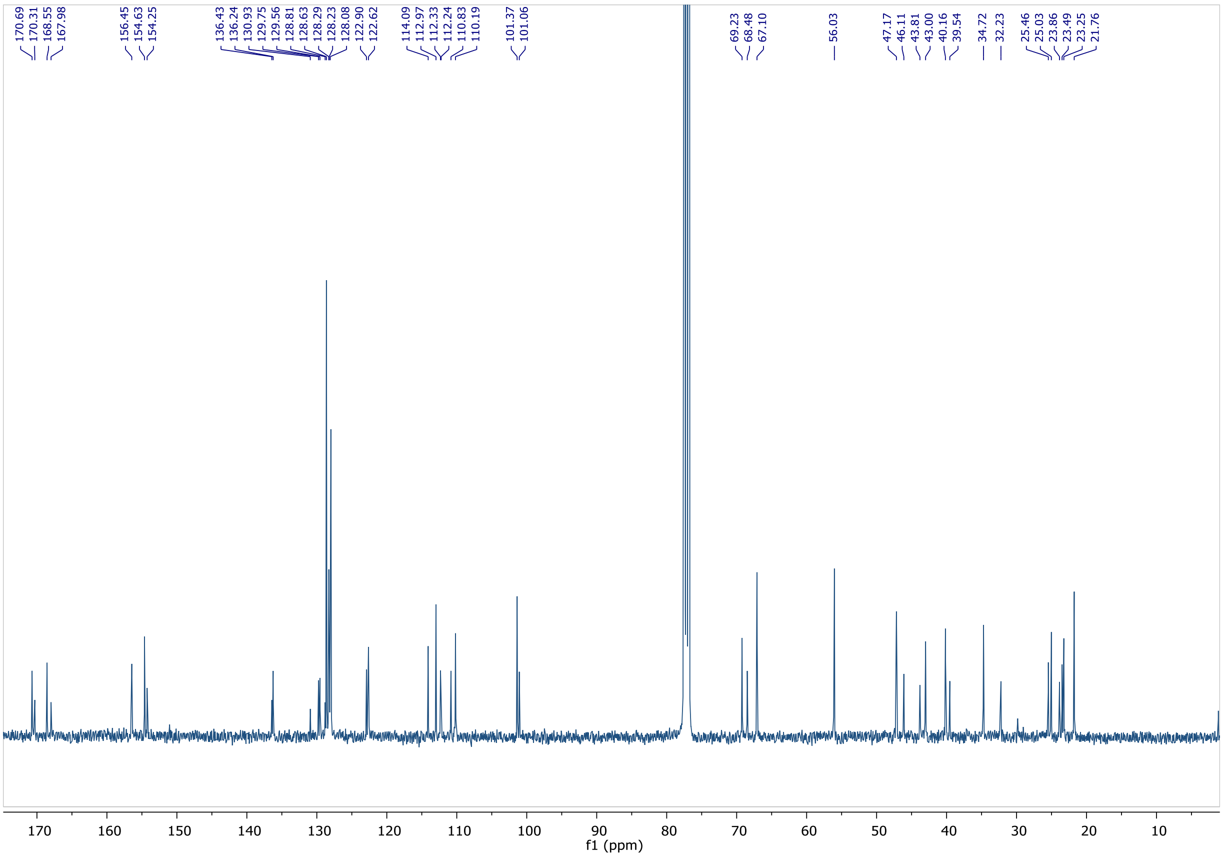

^{1}H-NMR (400 MHz, Chloroform-*d*) (6g)

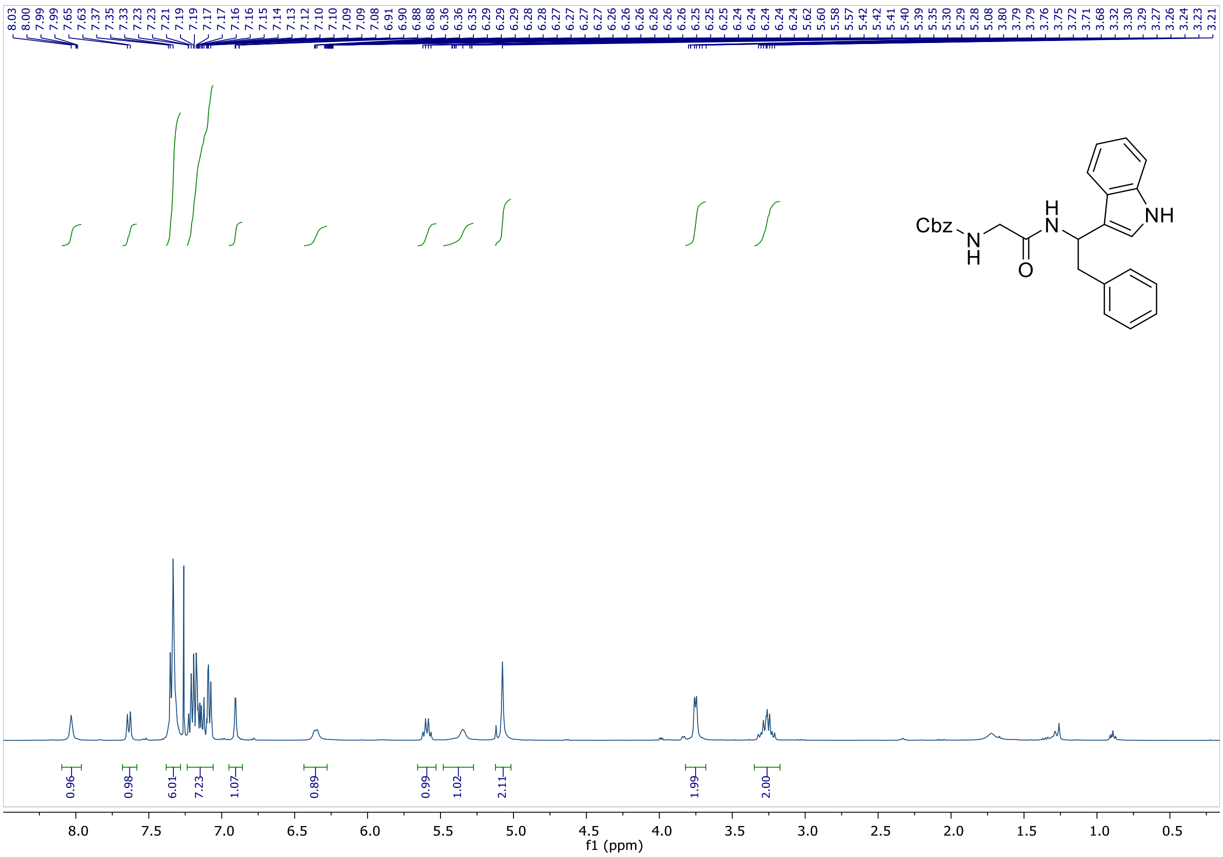

^{13}C-NMR (101 MHz, Chloroform-*d*) (6g)

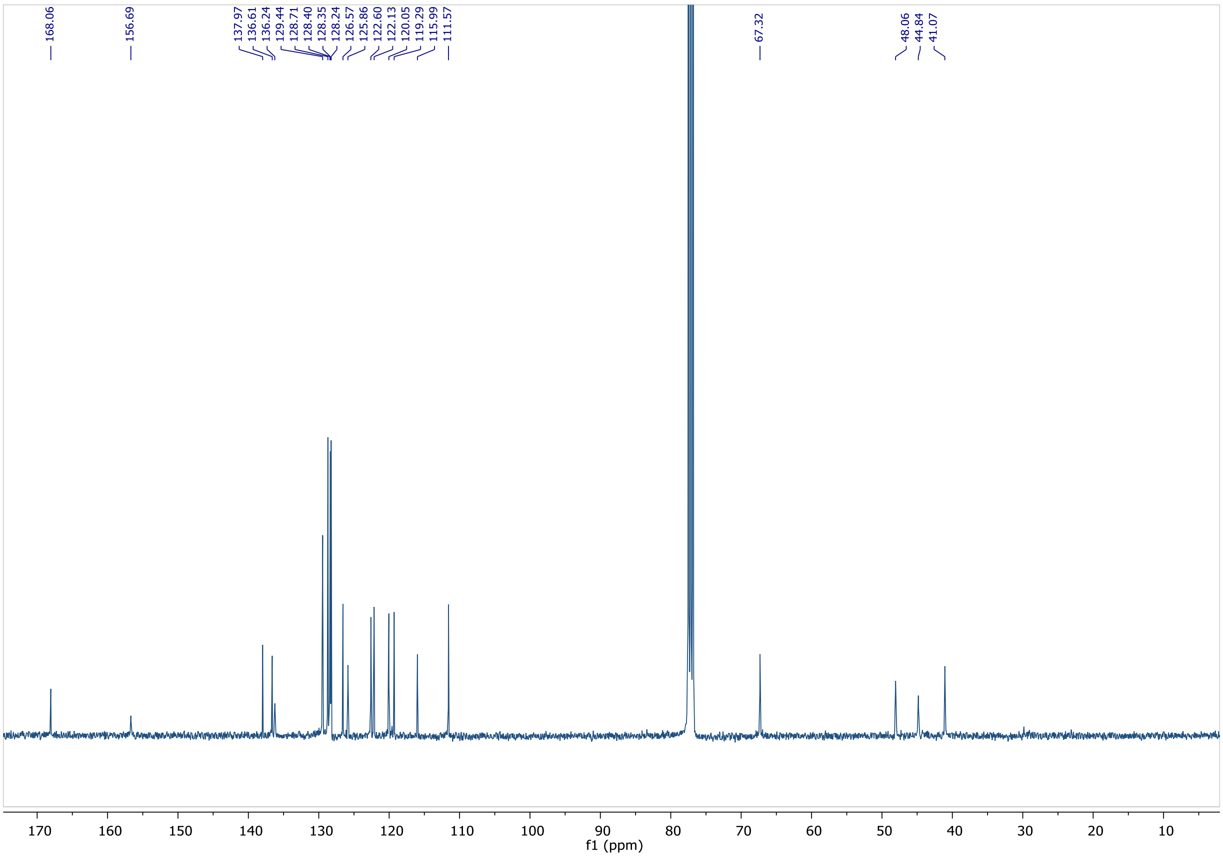

^{1}H-NMR (400 MHz, Chloroform-*d*) (6h)

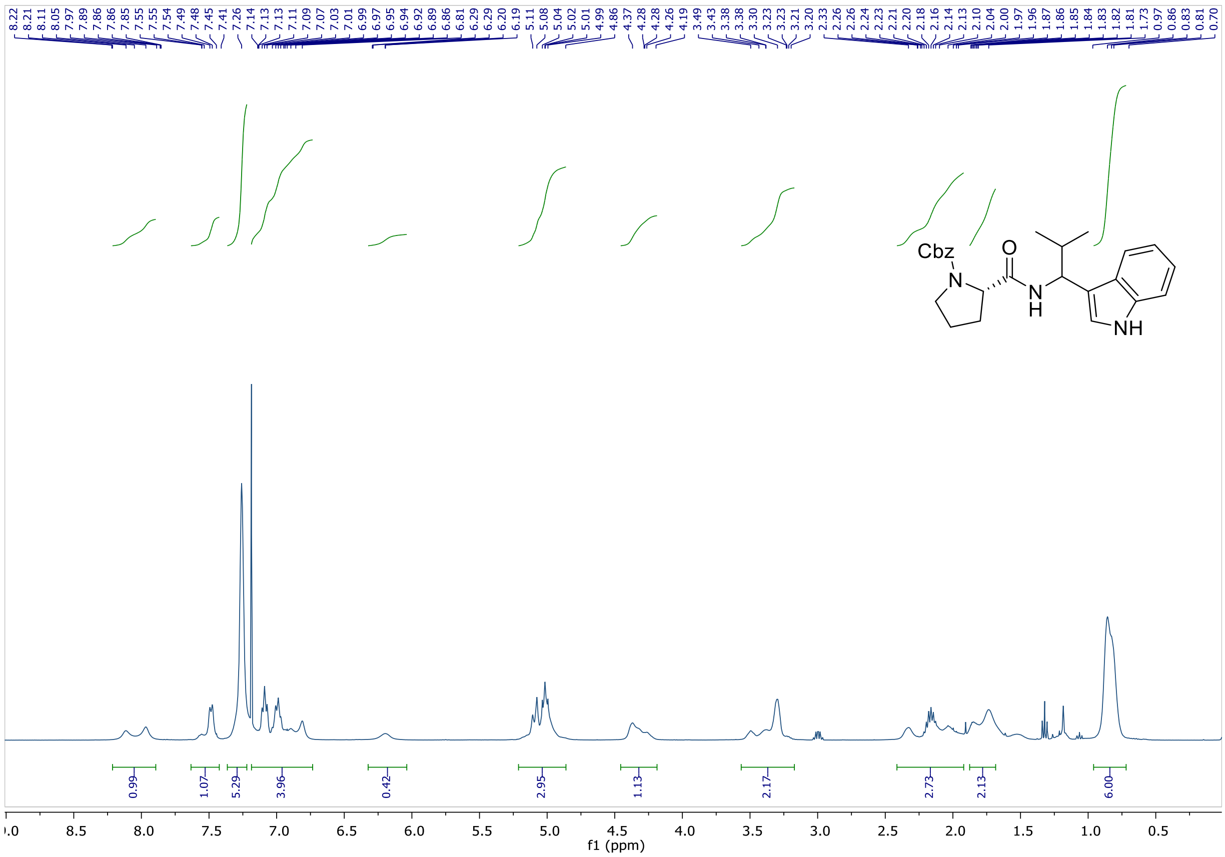

^{13}C-NMR (101 MHz, Chloroform-*d*) (6h)

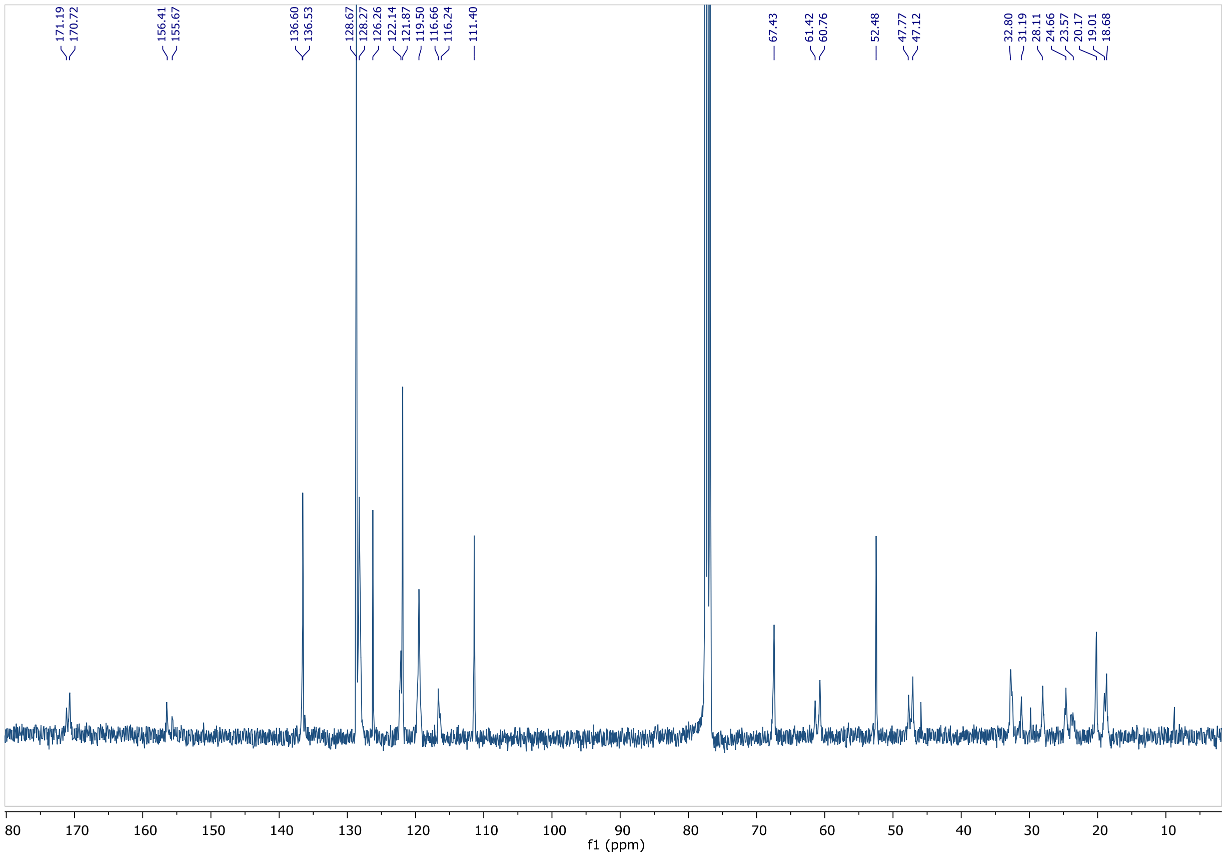

^{1}H-NMR (400 MHz, Chloroform-*d*) (6i)

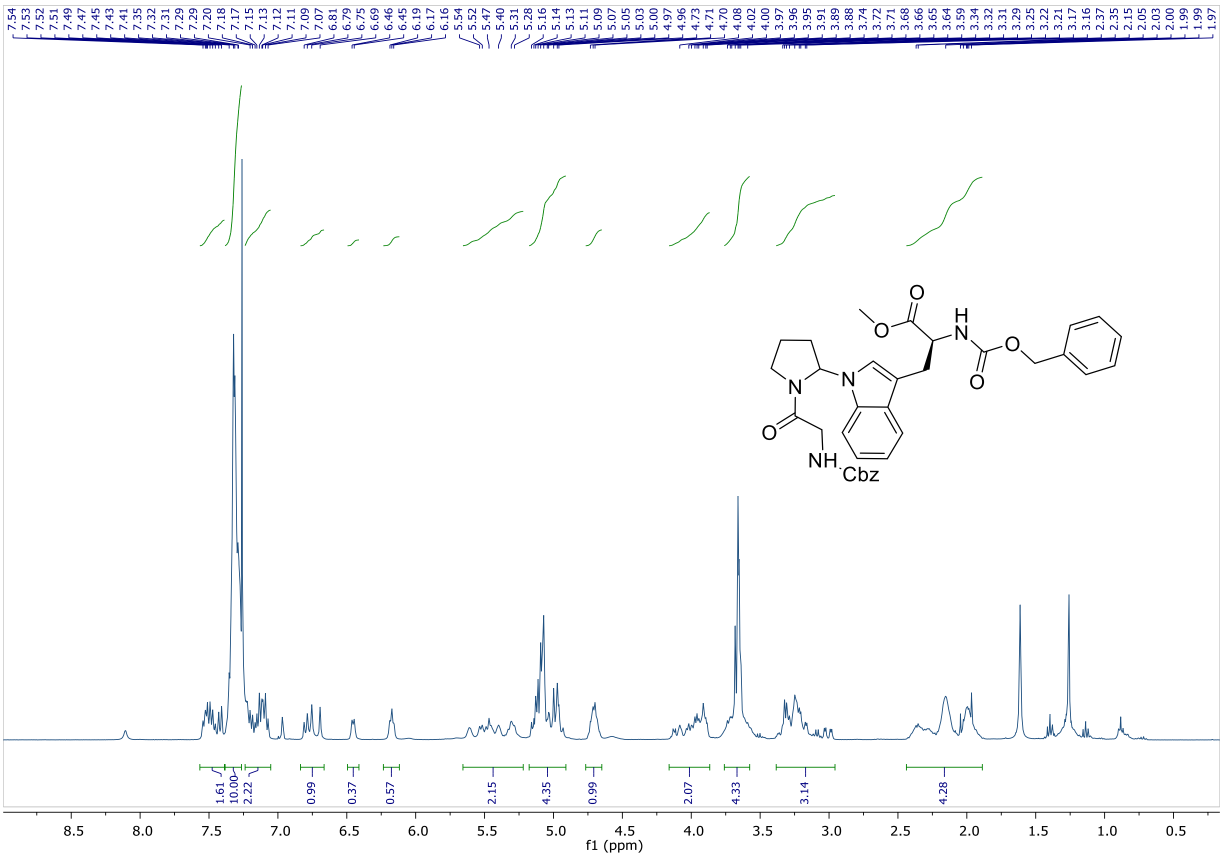

^{13}C-NMR (101 MHz, Chloroform-*d*) (6i)

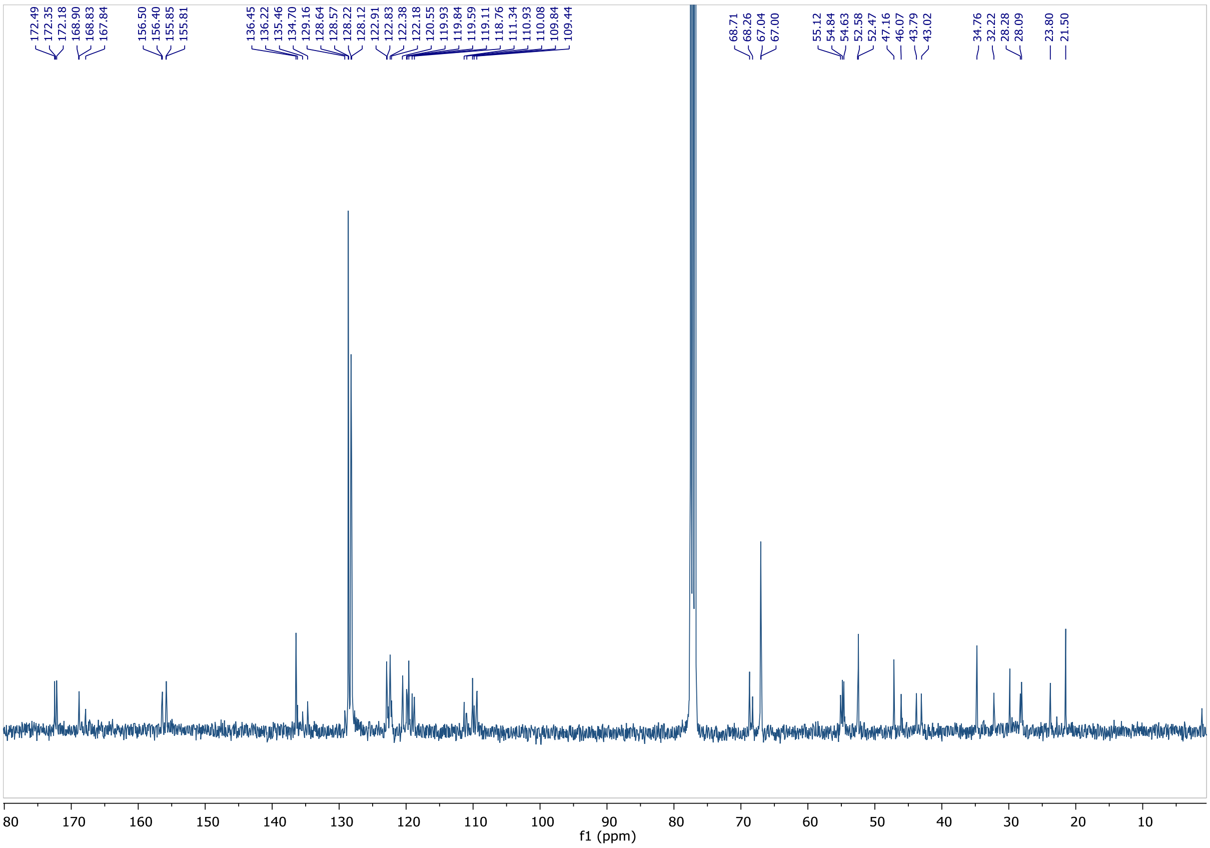

^{1}H-NMR (400 MHz, Acetonitrile-d_3) (6j)

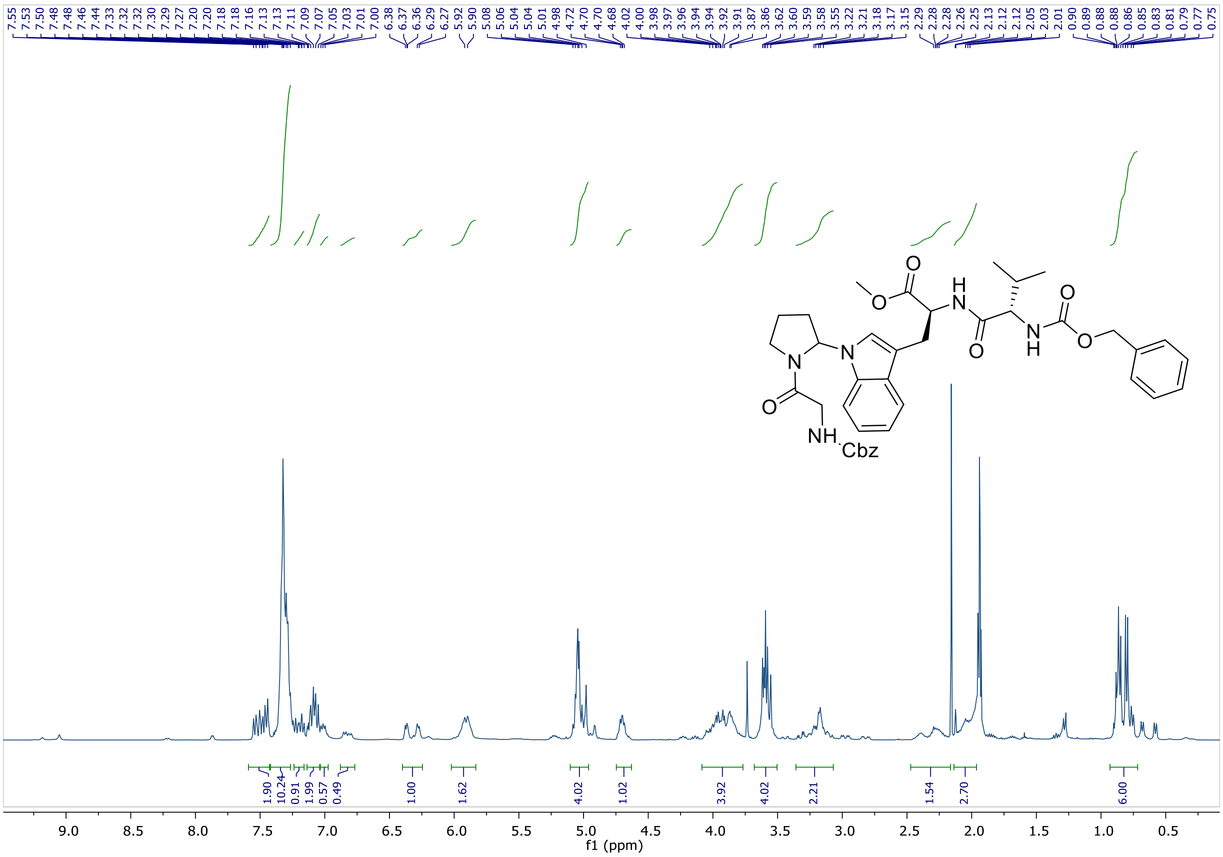

^{13}C-NMR (101 MHz, Acetonitrile-$d3$) (6j)

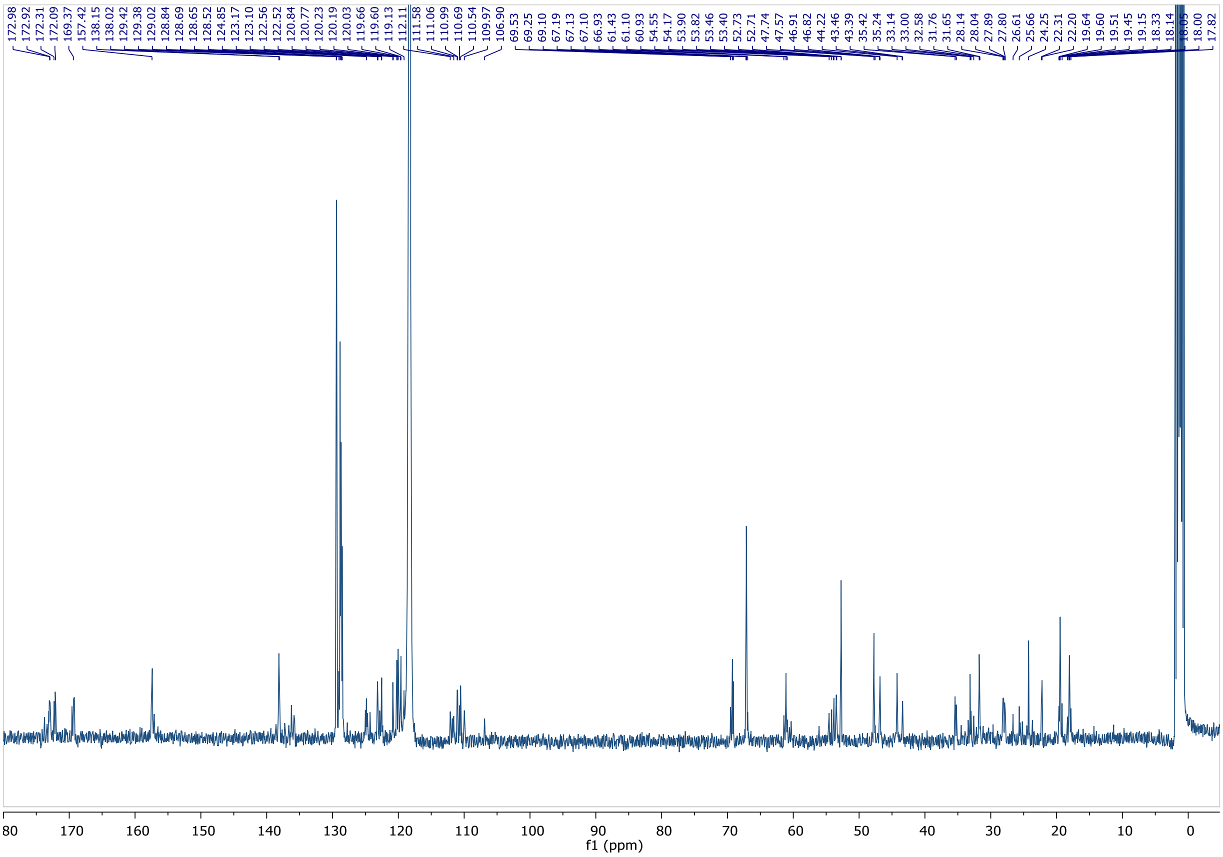

^{1}H-NMR (400 MHz, Acetonitrile-d_3) (8a)

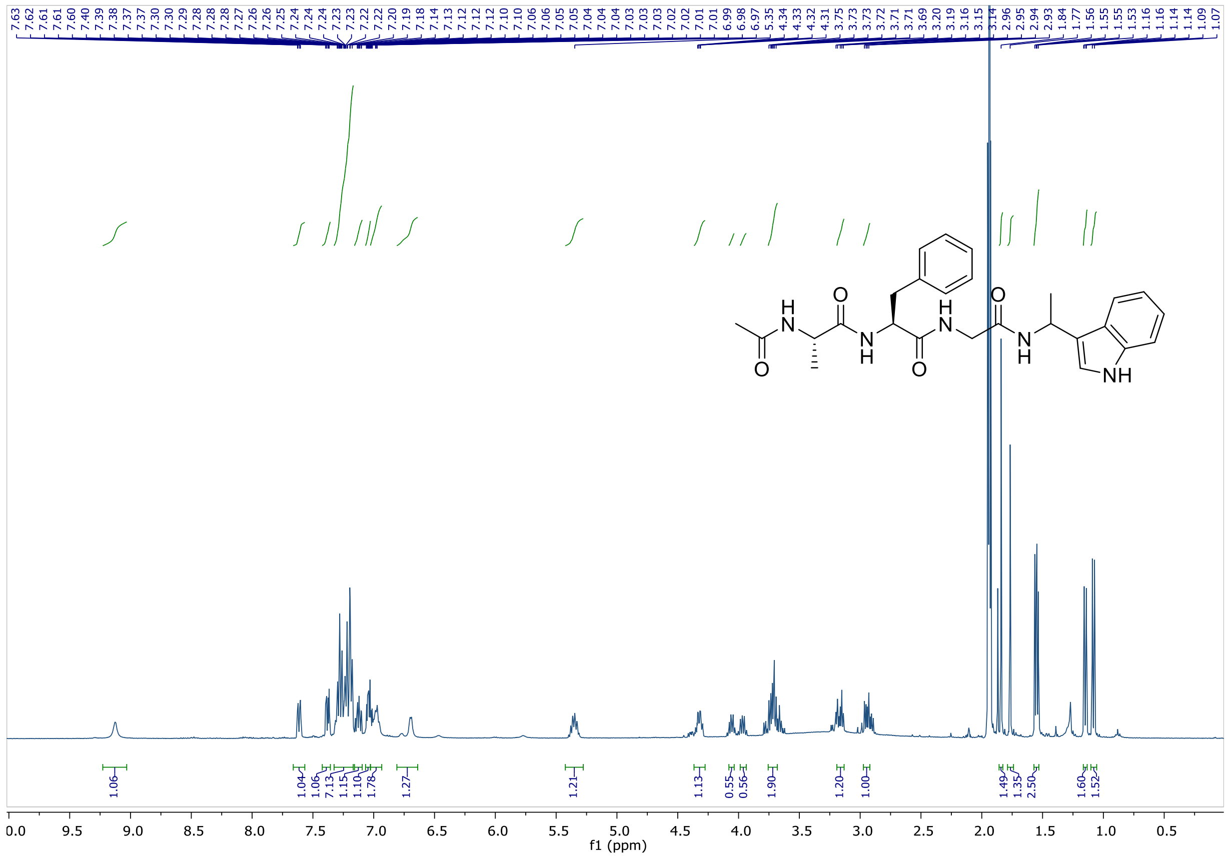

^{13}C-NMR (101 MHz, Acetonitrile-$d3$) (8a)

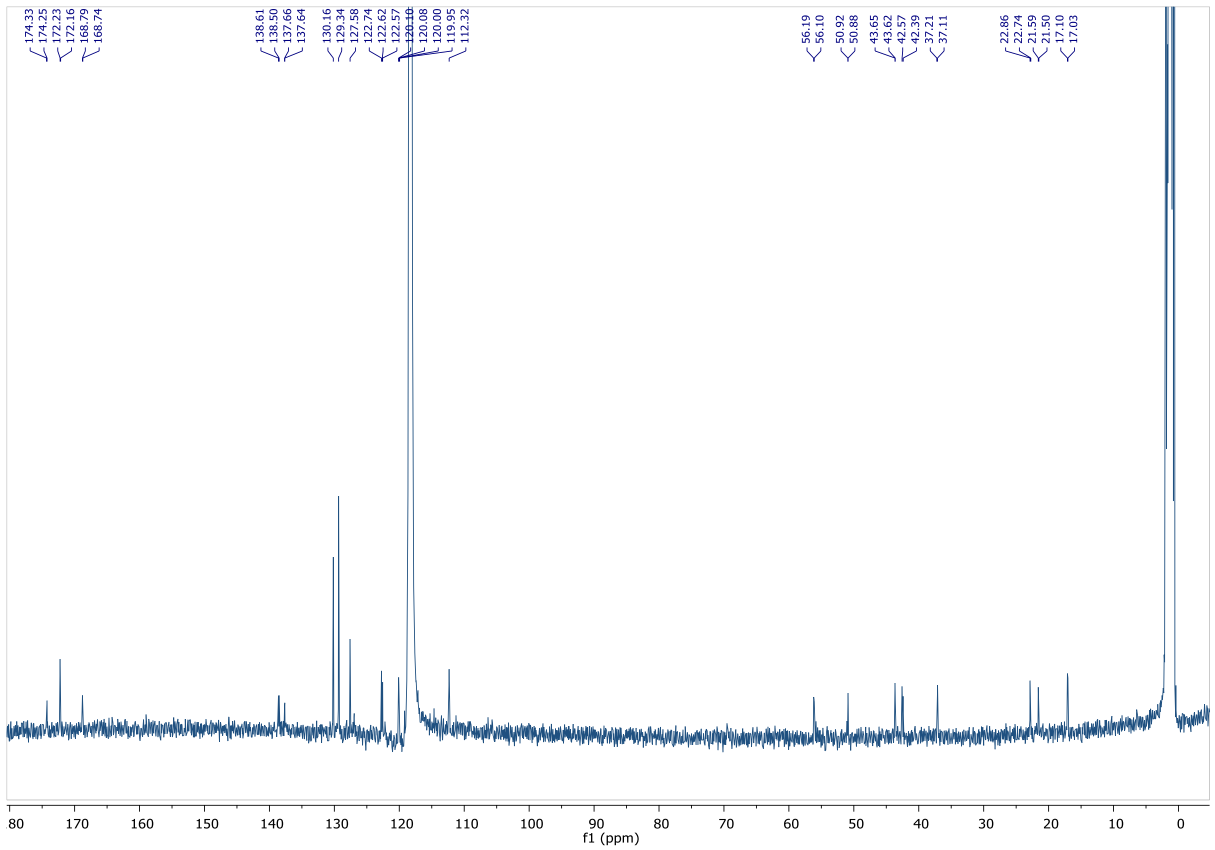

Supplement: SC-012-D0SC06180H-s001 [file SC-012-D0SC06180H-s001.pdf]
